# A novel ECG analog 4-(*S*)-(2,4,6-trimethylthiobenzyl)-epigallocatechin gallate selectively induces apoptosis of B16-F10 melanoma via activation of autophagy and ROS

**DOI:** 10.1038/srep42194

**Published:** 2017-02-10

**Authors:** Jing Xie, Ju-ping Yun, Ya-nan Yang, Fang Hua, Xiao-wei Zhang, Heng Lin, Xiao-xi Lv, Ke Li, Pei-cheng Zhang, Zhuo-wei Hu

**Affiliations:** 1 State Key Laboratory of Bioactive Substances and Functions of Natural Medicines, Institute of Materia Medica, Chinese Academy of Medical Sciences and Peking Union Medical College, Beijing 100050, People’s Republic of China; 2 Department of Pharmacy, China-Japan Friendship Hospital, Beijing 100050, People’s Republic of China; 3 Laboratory of Antiviral Research, Institute of Medicinal Biotechnology; Chinese Academy of Medical Sciences & Peking Union Medical College, Beijing 100050, People’s Republic of China

## Abstract

Autophagy-induced cancer cell death has become a novel strategy for the development of cancer therapeutic drugs. Numerous studies have indicated that green tea polyphenols induce both autophagy and apoptosis in a variety of cancer cells. Here, we synthesized a series of green tea polyphenol analogues, among which JP8 was shown to potently activate autophagy. JP8 treatment had a stronger effect on apoptosis in B16-F10 melanoma cells than that in normal AML-12 hepatocytes. JP8 selectively resulted in reactive oxygen species (ROS) accumulation in B16-F10 cells, and this effect was associated with corresponding increases in key components of the ER stress-mediated apoptosis pathway. Pharmacological inhibition of ROS by N-acetyl-L-cysteine (NAC) attenuated JP8-induced autophagy and apoptosis, indicating an upstream role of ROS in JP8-induced autophagy. An *in vivo* study showed that JP8 had significant antitumor effects in a B16-F10 xenograft mouse model. Our results indicate that JP8 is a novel anticancer candidate with both autophagy and ROS induction activities.

Autophagy is a catabolic process in which cells respond to external stresses by recycling intracellular components, including proteins, ribosomes, lipids, and even entire organelles. Studies from our laboratory and others have suggested that the expression of autophagic genes and their corresponding activities are frequently suppressed in cancer cells and that autophagy plays a critical role in carcinogenesis as a tumor suppressor^1^,^2^,^3^,^4^,^5^,^6^. Furthermore, autophagy may have a role in type II programmed cell death in cancer cells in which apoptosis is defective or difficult to induce^7^. Therefore, we hypothesized that the induction of cell death by autophagy may be used as a novel therapeutic strategy to treat cancer.

Currently, many autophagy modulators are derived from natural products, including a variety of polyphenolic compounds found in food, which are active against different cancer types^8^,^9^. Green tea has been the subject of numerous studies due to its beneficial effects on multiple diseases, including cancer, atherosclerosis, and cardiovascular diseases^10^,^11^,^12^,^13^,^14^. These healing properties are attributable to its abundant polyphenolic catechins, such as epigallocatechin-3-gallate (ECG) and epigallocatechin gallate (EGCG)^15^,^16^. However, these compounds inhibit cancer cell growth at concentrations of 150–200 *μ*M in cultured cells^17^,^18^,^19^,^20^. The relatively low potency of green tea polyphenols limits their preclinical use in cancer studies and treatment. Here, we aimed to identify novel polyphenol analogs with potent autophagy-inducing and tumor inhibition activities.

We synthesized a series of analogs based on ECG and EGCG by introducing a thioether group into ring C at C-4. At the same time, we constructed an autophagy-screening system to identify novel autophagy activators from our compound library. Among the analogs, JP8 exhibited potent autophagy-inducing activity. Subsequently, cell viability assays were performed using these compounds in several tumor cell lines and their normal counterparts. JP8 was shown to potently and preferentially induce cell death in B16-F10 melanoma cells.

Polyphenols are extensively studied for their anti-oxidant or pro-oxidant abilities. In this study, we found that JP8 selectively induced reactive oxygen species (ROS) accumulation in cancer cells. We also assessed whether this selective induction of ROS was responsible for the selective cancer cell-killing properties of JP8. In addition, the relationship between JP8-induced ROS and autophagy was also studied using the ROS scavenger N-acetyl-L-cysteine (NAC) and siRNA knockdown of ATG5, an autophagy gene. Finally, *in vivo* antitumor activity was examined, and the results showed that JP8 is a promising therapeutic candidate with both autophagy- and ROS-modulating activities.

## Results

### Chemistry

Polymeric flavan-3-ols (PFs) are important and abundant secondary metabolites found in the condensed tannins of many plants, such as green tea, *Rheum palmatum* L., *Polygonum cuspidatum, Acacia catechu*, and *Cinnamomum cassia*. Structurally, PFs are oligomers of catechins, epicatechins (EC), epigallocatechin-3-gallate (ECG), and epigallocatechin gallate (EGCG) via a C-4/C-6 or C-4/C8 bond. A literature^21^ search revealed that acid-mediated depolymerization of PFs in the presence of thiol nucleophiles leads to β-C-4 substituted flavanol derivatives, which is a simple and rapid synthetic method to obtain flavanol derivatives with a substituted group at C-4.

Our aim was to test the antitumor effects of ECG and EGCG derivatives. The purified PFs, which principally contained ECG polymers (ECGp) or EGCG polymers (EGCGp), were the key precursors for the synthesis of target compounds. To obtain ECG polymers and EGCG polymers, we performed depolymerization experiments on five natural plants (green tea, *Polygonum cuspidatum*, grape seeds, *Rhodiola crenulata*, and *Rhodiola kirilowii*) using benzyl mercaptan as the nucleophile. The results revealed that the major contents of *Rhodiola crenulata* and *Rhodiola kirilowii* were ECGp and EGCGp, respectively. The *R. crenulata* and *R. kirilowii* root extracts were further purified by a macroporous adsorption resin (HP-20) column to yield ECGp and EGCGp, respectively.

**Figure 1:**
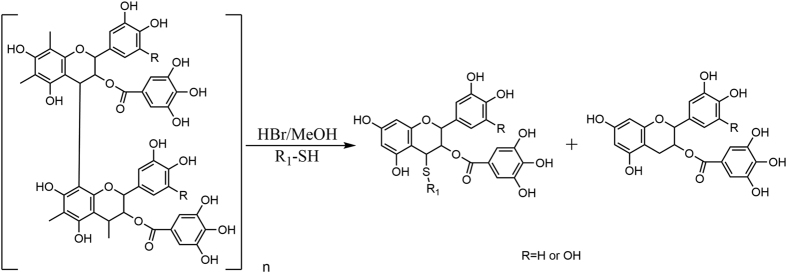
The synthetic route of flavan derivatives.

We designed and synthesized a series of *β*-C-4-substituted flavanol derivatives based on ECGp and EGCGp in a one-step reaction, as shown in Fig. 1. These compounds could be classified into four types of derivatives according to the type of substituted group at C-4, including thio-benzyl (I), thio-phenyl (II), thio-alkyl (III), and thio-carboxylic ester (IV). For types I and II, we evaluated the bioactivity of compounds **1**–**29** when the electron-donating group, electron-withdrawing group, and alkyl were linked to a benzene ring. For types III and IV, we investigated the influence of the length of substituted alkyl chain on bioactivity.

### Biological Results

#### Screening for autophagy activators

LC3 is the classic marker used to monitor autophagic activity. When autophagy is stimulated, the soluble form of LC3 (LC3-I) is converted into a lipidated form (LC3-II), which is tightly bound to the autophagosomal membranes and thus serves as an autophagic marker protein. When fused with green fluorescent protein (GFP), LC3 fluorescence can be quantified using FACS to measure autophagy. In our screening system, we detected the fluorescence of membrane-bound GFP-LC3-II after a saponin extraction procedure^22^. To examine autophagic flux, we pre-treated the cells with the lysosomotropic agent bafilomycin A1 to prevent fusion of autophagosomes with lysosomes. An autophagy activator increased GFP-LC3-II, and further increases were observed with the addition of bafilomycin A1.

CHO cells stably expressing GFP-LC3 were treated with each newly synthesized compound at 5 concentrations ranging from 1 to 50 *μ*M. The minimum effective concentration (MEC) for autophagy activation was defined as the minimum concentration of compound that caused a statistically significant increase in GFP-LC3-II fluorescence. Among the 57 newly synthesized compounds, JP8 was identified as the most potent autophagy activator, with a MEC of 5 *μ*M. JP21 was also identified as autophagy activator, although it was less potent than JP8. JP19 and JP29 displayed weak activation of autophagy (Table 1). All of these compounds showed more effective induction of autophagy than that of their parental compounds ECG and EGCG; there was no detectable activity with ECG and EGCG in our screening system, even at the highest concentration tested. Previous findings have demonstrated the autophagy-induction activity of both EGCG and ECG with the effective concentration ranging from 10 to 250 *μ*M, according to Western Blot analysis of LC3-II/I^17^,^18^,^19^. In our screening system, the fluorescence of autophagesome bound GFP-LC3-II was used as the readout, this may result in undetectable signals in ECG and EGCG treated cells due to different sensitivity compared to direct western blot analysis.

#### JP8 is identified as an autophagic flux activator using the autophagy-screening system

**Figure 2:**
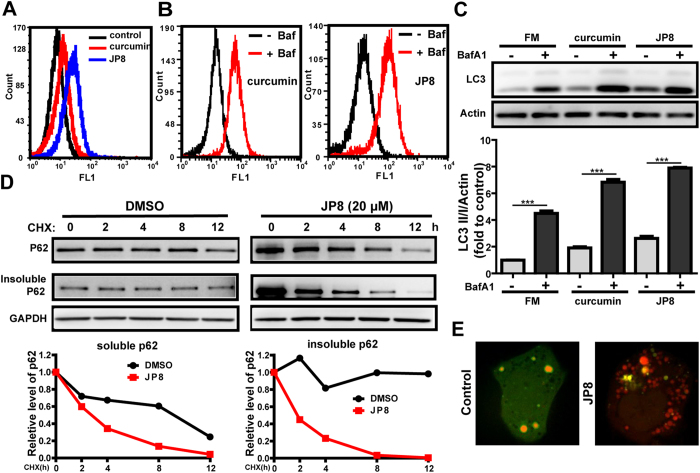
JP8 activates autophagic flux. **(A)** JP8 increases the conversion of LC3 I to LC3 II. CHO cells stably expressing GFP-LC3 were incubated with JP8 (10 *μ*M) for 12 h. Cells were washed with PBS containing 0.05% saponin and then analyzed by flow cytometry. The fluorescence intensity is directly correlated with LC3 II levels. Curcumin (20 *μ*M) was used as a positive control. **(B** and **C)** JP8 further increases LC3 II level following treatment with bafilomycin A1. Cells stably expressing GFP-LC3 were treated with JP8 for 12 h with or without the addition of bafilomycin A1. After the cells were washed with PBS containing 0.05% saponin, cells were analyzed by flow cytometry **(B)**. Wild type CHO cells were treated with JP8 for 12 h with or without the addition of bafilomycin A1. Then, the cell lysates were analyzed by immunoblotting. Images are representatives and bar graphs are quantified results of three independent experiments expressed as the mean ± SEM, ***P < 0.001 compared to the control.**(C)**. **(D)** JP8 promotes the degradation of the autophagic substrate p62. Wild type CHO cells were incubated with CHX (10 *μ*g/ml) for the indicated times after 2 h of JP8 or DMSO vehicle stimulation. Then, the cell lysates were collected for immunoblotting. GAPDH was used as the loading control. **(E)** Representative images of live cells infected with GFP-RFP-LC3 adenovirus with or without the presence of JP8 (20 *μ*M) for 12 h.

JP8 potently induced LC3 conversion, as indicated by the increase in fluorescence intensity (Fig. 2A). Administration of bafilomycin A1 further increased the JP8-induced level of LC3-II (Fig. 2B and C).

The ultimate goal of autophagy is the degradation of proteins and organelles mediated by autophagic cargo receptors, such as p62, NBR1, NDP52 and NIX, which contain an LC3-interacting region (LIR) and can therefore bind directly to LC3^23^,^24^. We evaluated the effects of JP8 on autophagy substrate degradation. JP8 promoted the degradation of both soluble and insoluble p62 (Fig. 2D), indicating an acceleration of autophagic degradation by JP8.

Recent studies have used tandem fluorescent proteins to measure autophagic flux based on the concept of lysosomal quenching of GFP^25^. GFP is a stably folded protein and is relatively resistant to lysosomal proteases. However, the low pH inside the lysosome quenches the fluorescence signal of GFP. In contrast, red fluorescent protein (RFP) exhibits more stable fluorescence in acidic compartments, and mRFP-LC3 can readily be detected in autolysosomes. In RFP-GFP-LC3-transfected cells, autophagosomes and autolysosomes are labeled with yellow (i.e., mRFP and GFP) and red (i.e., mRFP only) signals, respectively. If the autophagic flux is increased, both yellow and red puncta are increased. However, if the maturation of autophagosomes into autolysosomes is blocked, only yellow puncta are increased without a concomitant increase in red puncta^26^. Our results showed that in normal conditions, mRFP-GFP-LC3 was detected as yellow puncta, indicating a steady state of autophagy. In contrast, after treatment with JP8, increased red puncta and dynamically moving yellow spots were observed. Together, these results suggested that JP8 increases autophagic flux (Fig. 2E and Supplementary Videos).

#### JP8 preferentially induces cell death in B16 melanoma cells

**Figure 3:**
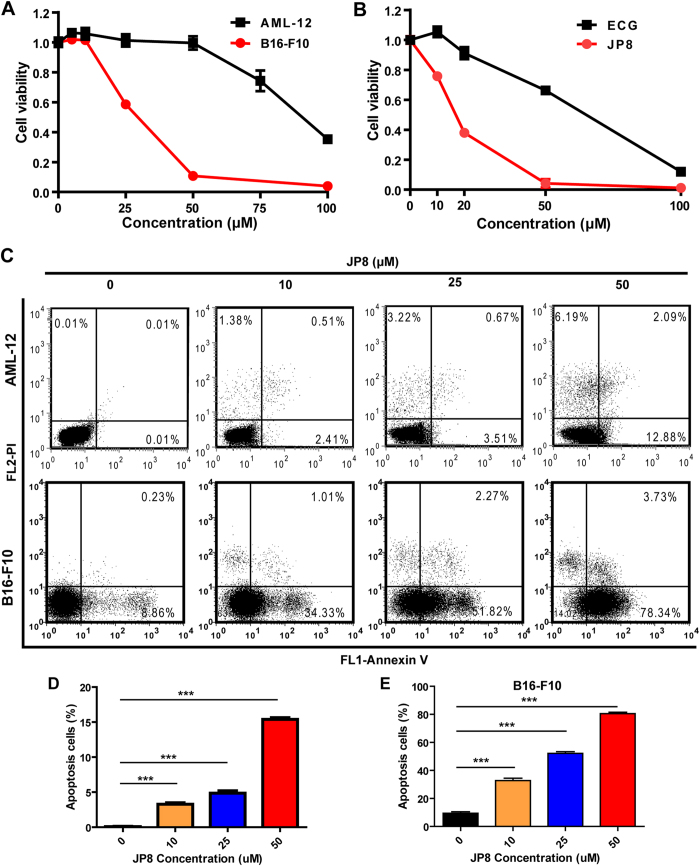
JP8 preferentially induces cell death in cancer cells. **(A)** JP8 treatment induces cell death in mouse B16-F10 melanoma cells more potently than that in normal mouse AML-12 hepatocyte cells. Cells were grown in 96-well plates and treated with JP8 at 5–100 *μ*M or DMSO (control) for 48 h. Cytotoxicity was measured using cell counting kit-8 (CCK-8) assays. **(B**) JP8 inhibits cancer cell growth more effectively than its skeleton ECG. B16-F10 cells were grown in 96-well plates and treated with JP8 or ECG at 10–100 *μ*M for 48 h. Cell viability was measured using CCK-8 assays. **(C**,**D** and **E)** JP8 treatment induces apoptosis in B16-F10 cells more potently than that in AML-12 cells. Cells were grown in 12-well plates and treated with JP8 at indicated concentration for 48 h. Apoptosis was detected by flow cytometry using annexin V and PI double staining. Dot plots are representatives and bar graphs are quantified results of three independent experiments expressed as the mean ± SEM, **P < 0.01, ***P < 0.001 compared to the control.

After identifying JP8 as an autophagy activator, we evaluated its anticancer effects on different cancer cells and its toxicity in normal cells. Exposure to JP8 reduced cell viability in B16 melanoma cells more efficiently than that in normal mouse AML-12 hepatocyte cells (Fig. 3A, Figure S171 in Supporting Information). JP8 treatment resulted in a larger proportion of apoptotic cells in B16 cells than that in AML-12 cells (Fig. 3C,D and E, Figure S172 in Supporting Information). JP8 also has some selectivity on cell growth inhibition between human skin-origin melanoma cells and normal cells (Figure S171 in Supporting Information). In addition, JP8 more effectively inhibited B16 cells than the ECG parent compound (Fig. 3B).

#### JP8 selectively induces ROS accumulation in cancer cells

**Figure 4:**
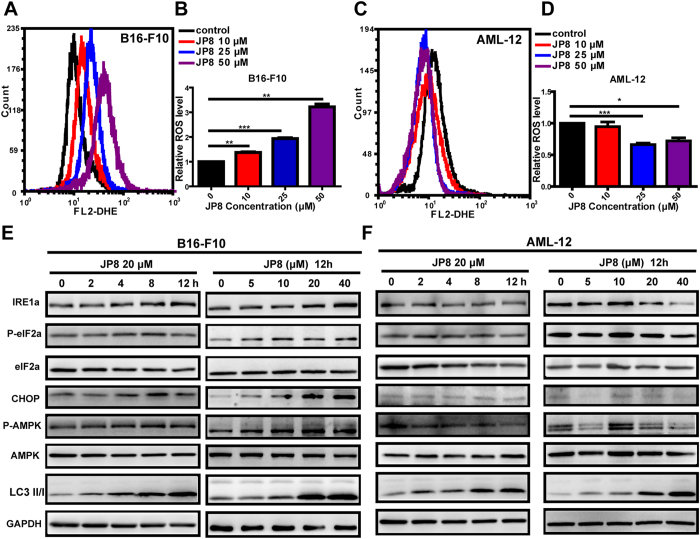
JP8 selectively induces ROS accumulation and stress response-related proteins in B16-F10 cells. JP8 treatment increases ROS in the cancer cell line B16-F10 (**A** and **B)** but not the normal cell line AML-12 **(C** and **D)**. Cells were treated with JP8 at the indicated concentrations for 24 h and stained with dihydroethidium (DHE). Intracellular ROS levels were analyzed with flow cytometry. B16-F10 **(E)** and AML-12 **(F)** cells were treated with JP8 for the indicated times and at the indicated concentrations, and then, the protein expression levels of IRE1a, p-elF2a, elF2a, CHOP, p-AMPK, AMPK, and LC3 were assessed. GAPDH was used as a loading control. Western blot results are representative of three independent experiments with identical results. Bar graphs represent the fold increase in ROS in three independent experiments expressed as the mean ± SEM, *P < 0.05, **P < 0.01, ***P < 0.001 compared to the control.

Based on the polyphenol structure of JP8, we hypothesized that JP8 may modulate ROS. We measured ROS levels in both B16 and AML-12 cells after JP8 treatment. Surprisingly, we found that JP8 treatment dramatically increased ROS accumulation in B16 cells in a dose-dependent manner (Fig. 4A,B and Figure S173 in Supporting Information). In contrast, JP8 decreased ROS levels in normal AML-12 cells (Fig. 4C,D and Figure S173 in Supporting Information). We have also observed that the EC50 (24.03 *μ*M) of JP8 on ROS induction was very close to the IC50 (26.41 *μ*M) of JP8 on cell death induction in B16-F10 cell line (Figure S174 in Supporting Information), indicating an underlying mechanism of ROS in JP8 induced cancer cell death. We also test this selectivity on other skin and hepatocyte-origin cancer and normal cell lines. In consistent with our above findings, JP8 selectively increased ROS accumulation in A375, SK-Mel-1 and HepG2 cancer cell lines while this ROS induction effects was not obviously observed in HaCaT and CCC-ESF-1 normal cell lines (Figure S175 in Supporting Information). This selective induction of ROS in cancer cells distinguishes JP8 from other small molecules that alter ROS levels, such as curcumin and resveratrol^27^,^28^,^29^,^30^. Furthermore, ECG, the precursor of JP8, did not affect the ROS levels in B16 cells (Figure S176 in Supporting Information).

We next examined the expression levels of components in the stress response pathways in JP8-treated cancer and normal cell lines. Consistent with our above findings, JP8 upregulated the expression of IRE1a, phospo-elF2a and CHOP in B16 cells in concentration- and time-dependent manners (Fig. 4E), and these changes were not observed in the AML-12 cell line (Fig. 4F). Adenosine monophosphate-activated protein kinase (AMPK) is an energy-sensing molecule and a positive modulator that activates autophagy and can be induced by polyphenols such as EGCG^31^ and resveratrol^32^. We found that JP8 treatment activated AMPK in B16 cells but not in AML-12 cells, indicating a ROS-dependent role of AMPK activation in JP8 treatment.

#### ROS-mediated cell damage is a critical mechanism for JP8-induced autophagy and cell death

**Figure 5:**
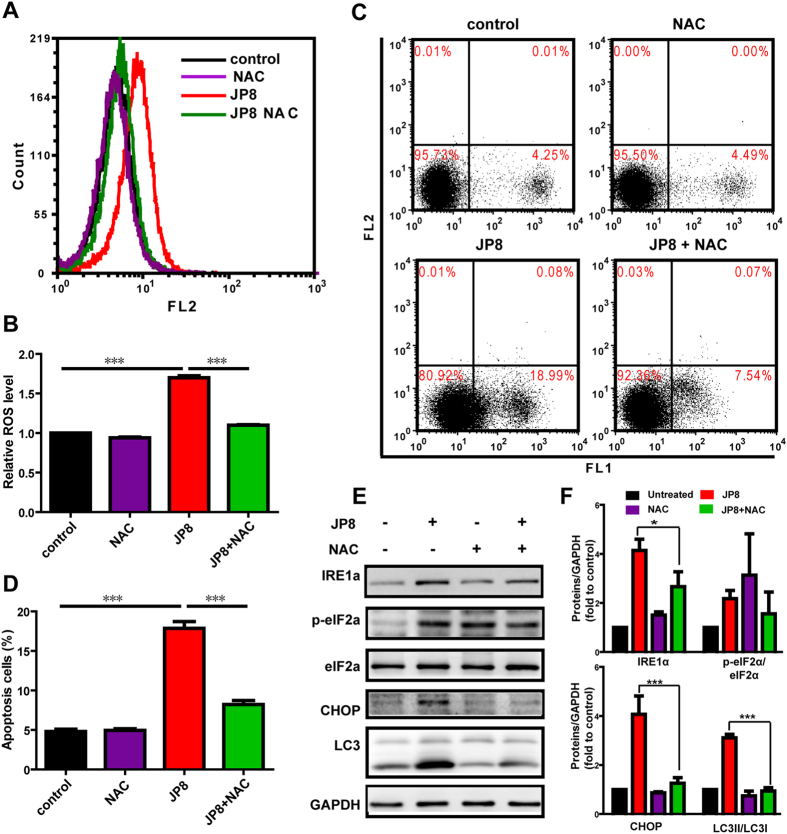
JP8 induces intracellular ROS-mediated apoptosis and autophagy. **(A**,**B)** Co-treatment with NAC abolishes JP8-induced ROS accumulation in cancer cells. B16-F10 cells were treated with 20 *μ*M JP8 for 12 h in the presence or absence of 2 mM NAC. Cells were stained with DHE and examined by flow cytometry. Bar graph represents the fold increase in ROS of three independent experiments expressed as the mean ± SEM, ***P < 0.001. **(C**,**D)** Co-treatment with NAC reduces JP8-induced apoptosis in B16-F10 cells. B16-F10 cells were treated with 20 *μ*M JP8 for 12 h in the presence or absence of 2 mM NAC. Cell apoptosis was measured by annexin V/PI assays. The dot plots are representative and the bar graph is the mean ± SEM of three independent experiments, ***P < 0.001. **(E**,**F)** Western blot analysis of ROS-related proteins and autophagy marker LC3. B16-F10 cells were treated with 20 *μ*M JP8 for 12 h in the presence or absence of 2 mM NAC. GAPDH was used as a loading control. Images are representatives and bar graphs are quantified results of three independent experiments expressed as the mean ± SEM, *P < 0.05, ***P < 0.001 compared to the JP8 treated group.

Because autophagy and ROS are both important inducers of apoptosis, and we determined that JP8 promoted ROS generation, we examined whether ROS played a role in JP8-elicited apoptosis and autophagy. We used NAC (5 mM), a ROS scavenger, to assess its effects on apoptosis and autophagy after JP8 treatment. Pretreatment with NAC substantially suppressed ROS accumulation (Fig. 5A and B) and reduced JP8-induced apoptosis in B16 cells (Fig. 5C and D). Stress-related proteins induced by JP8, such as IRE1a, p-elF2a and CHOP, were also attenuated in the NAC-treated group (Fig. 5E and F). ROS attenuation by NAC subsequently prevented JP8-induced autophagy, as indicated by the diminished conversion of LC3-I to LC3-II (Fig. 5E and F). Altogether, these findings reveal that ROS plays an important role in JP8-induced autophagy and apoptosis.

#### JP8-induced autophagy promotes the apoptotic cell death but does not affect ROS generation

**Figure 6:**
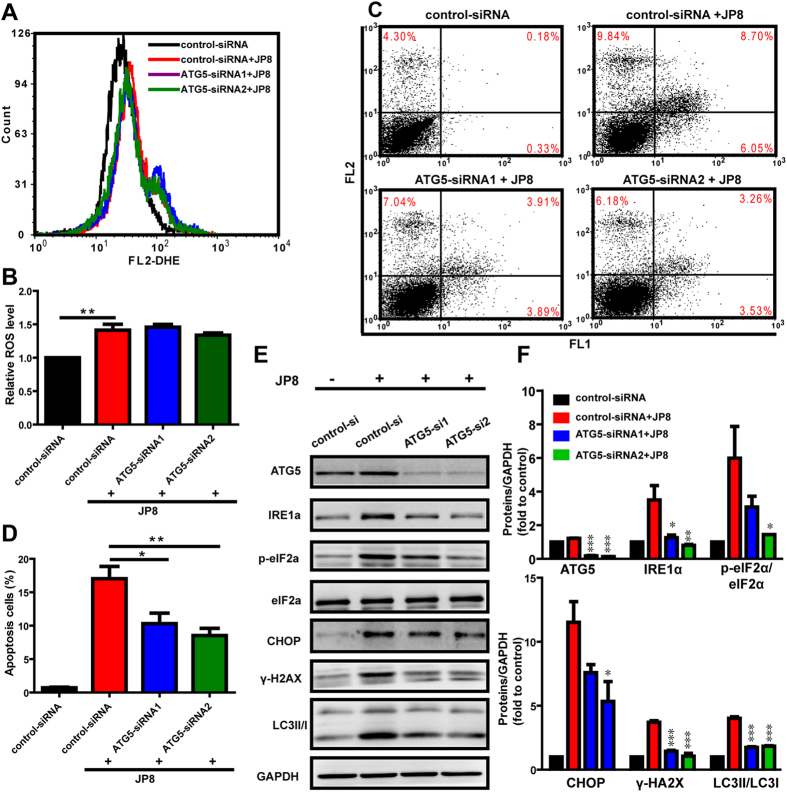
Autophagy enhances JP8-induced cell death in B16-F10 cells. **(A**,**B)** Autophagy inhibition does not affect JP8-induced ROS accumulation. Control-siRNA or ATG5-siRNA-transfected cells were treated with 20 *μ*M JP8 for 12 h. Cells were stained with DHE and analyzed by flow cytometry. Bar graph represents the fold increase in ROS of three independent experiments expressed as the mean ± SEM, **P < 0.01 compared to the control. **(C**,**D)** Autophagy inhibition reduces JP8-induced apoptosis. Control-siRNA or ATG5-siRNA-transfected cells were treated with 20 *μ*M JP8 for 12 h. Cell apoptosis was measured by annexin V/PI assays. The dot plots are representative and the bar graph is shown as the mean ± SEM of three independent experiments, *P < 0.05, **P < 0.01. **(E**,**F**) Control-siRNA or ATG5-siRNA-transfected cells were treated with 20 *μ*M JP8 for 12 h and subjected to western blot analysis using the indicated antibodies. The data are representative of three independent assays with identical results. Images are representatives and bar graphs are quantified results of three independent experiments expressed as the mean ± SEM, *P < 0.05, **P < 0.01, ***P < 0.001 compared to the control-siRNA and JP8 double treated group.

We subsequently assessed whether autophagy induction contributes to the ROS generation and cell death mediated by JP8. We assessed ROS and apoptosis levels in B16 cells with or without ATG5 depletion by siRNA. Autophagy inhibition by knockdown of ATG5 did not affect the JP8-induced increase in ROS levels (Fig. 6A and B). However, autophagy inhibition prevented JP8-induced apoptosis in B16 cells (Fig. 6C and D) and the upregulation of stress-related proteins (Fig. 6E and F). Furthermore, inhibition of autophagy by silencing of ATG5 can also attenuate JP8 induced cell death in AML-12 cells (Figure S177 in Supporting Information).

#### JP8 suppresses tumor growth *in vivo*

**Figure 7:**
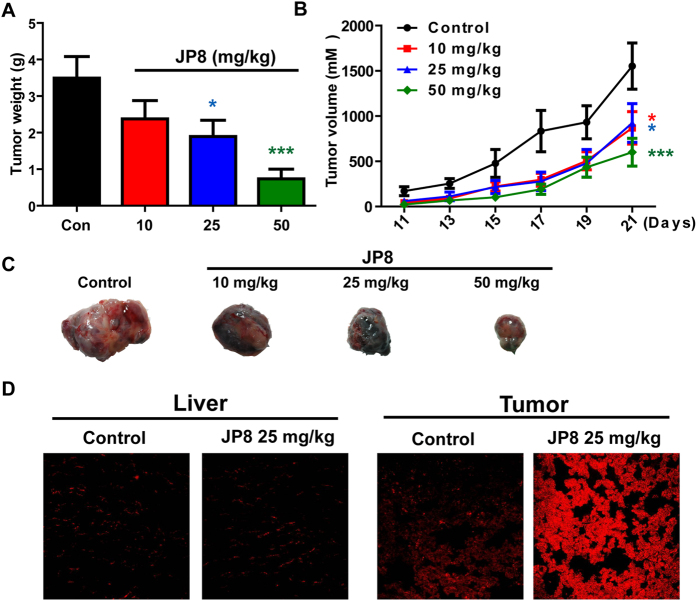
JP8 inhibits melanoma tumor growth in C57BL/6 mice. Mice were injected with B16 cells (3 × 10^5^) subcutaneously. The next day, mice were randomized and treated with vehicle or JP8 at the indicated doses for 21 days. **(A)** The tumor volume was calculated based on the width and length of tumors every other day during the whole process. **(B)** Tumor weight was measured after the mice were sacrificed on day 23. Data are presented as the mean ± SEM (n = 8). *P < 0.05, ***P < 0.001 compared to the control. **(C)** Photographs of representative tumors from each group are shown. **(D)** JP8 increases ROS accumulation in tumor tissues. Representative images of DHE-detected ROS in liver and tumor tissues from three independent assays.

We further investigated the *in vivo* antitumor efficacy of JP8 using a C57BL/6 mouse melanoma model. Tumors were established by subcutaneously implanting B16-F10 cells (3 × 10^5^ cells in 100 *μ*L of PBS) into the mice. JP8 at doses of 10, 25, and 50 mg/kg or vehicle were administered once daily intraperitoneally starting from day 3 to day 21. Treatment with JP8 at either 25 or 50 mg/kg resulted in significant suppression (P < 0.05) of tumor growth relative to that of the vehicle control group (Fig. 7A,B and C). To determine the *in vivo* relevance of our findings, we also measured ROS in both tumor and normal liver tissues. Consistent with the *in vitro* study, JP8 treatment induced ROS accumulation in tumor tissues but not in normal liver tissues (Fig. 7D).

## Discussion and Conclusion

Autophagy has important roles in major human diseases^33^,^34^; thus, identifying autophagy modulators is urgently needed. An important step towards this goal is to establish assays and screening systems that can rapidly and precisely measure autophagic flux in response to drugs. Most assays for autophagy modulators use the LC3 autophagy marker protein as a readout for autophagic activity. LC3, which is initially presented in a soluble form, becomes lipidated and closely associated with autophagosomal membranes upon autophagy induction. Thus, conversion of LC3 and its turnover can be used as a measure of autophagic activity. In our screening system, we used a strategy that combines the sensitivity of the GFP-LC3 reporter protein with the quantitative capacity of flow cytometry. To specifically monitor autophagosome-associated LC3 II, we used saponin extraction following to a protocol by Eng KE and colleagues^22^. Among the 57 novel synthesized polyphenols, JP8 was identified as a potent autophagy modulator characterized by significant accumulation of LC3 II.

Since the concept of autophagic flux was proposed, increasing numbers of researchers have recognized the distinction between autophagy induction and lysosomal inhibition^35^,^36^. To exclude the possibility that accumulation of LC3 II may be due to lysosomal inhibition, we examined three different aspects of autophagic flux. LC3 turnover was observed after the addition of bafilomycin A1, a lysosomal inhibitor. The degradation of the p62 autophagy substrate was accelerated following JP8 treatment. In addition, live cell imaging directly visualized the dynamic transition of LC3 from autophagosomes to autolysosomes. Together, these changes in LC3 and p62 indicate a dynamic autophagic flux induced by JP8. Activation of autophagic flux has been implicated in the treatment of human diseases^6^, and previous findings in our laboratory revealed that inhibition of autophagic flux may facilitate the development and progression of hepatocellular carcinoma^1^,^37^,^38^. Consistent with these hypotheses, our results showed that JP8 inhibits cancer cell growth in different cancer cell lines. Importantly, JP8 selectively killed cancer cells when added to both the B16-F10 and AML-12 cell lines. Furthermore, this anticancer property of JP8 was significantly better than that of its skeleton ECG.

**Figure 8:**
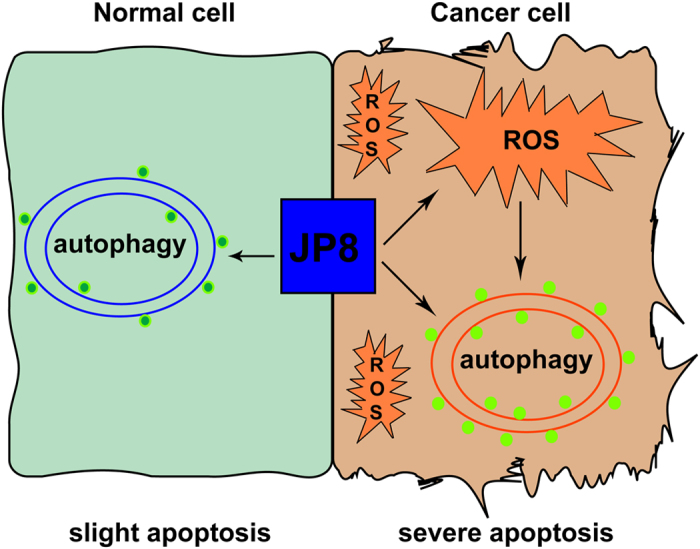
Schematic diagram illustrates the anti-melanoma mechanisms of JP8. JP8 activates autophagy in both normal and cancer cells and selectively induces ROS accumulation in cancer cells. ROS can lead to apoptosis in cancer cells; additionally, accumulation of ROS further accelerates autophagy and autophagy-induced cell death in cancer cells. Thus JP8 may exhibit selective toxicity against cancer cells and has few side effects on normal cells.

As the autophagy-inducing property of JP8 was not selective between normal and cancer cells, we hypothesized that other factors are responsible for this selectivity. Because JP8 has a polyphenol structure, and polyphenols are often related to ROS, which are either upstream of autophagy or a trigger of cell death^8^,^39^,^40^, we examined whether JP8 had pro- or anti-oxidant activity. Consistent with the above hypothesis, JP8 treatment induced selective ROS accumulation in cancer cells. Furthermore, oxidative stress-related biomarkers were also selectively induced in cancer cells in a time- and dose-dependent manner. Additionally, JP8 treatment promoted AMPK phosphorylation in cancer cells but not in normal cells. Previous reports have identified AMPK as a target of EGCG in inducing apoptosis^41^,^42^ and autophagy^31^. Our results suggested that AMPK is a link between ROS and autophagy in cancer cells. This induction of autophagy through AMPK may further aggravate JP8-induced cell death in cancer cells compared to that in normal cells. This is the first report demonstrating that the tea polyphenol analog JP8 induces selective ROS accumulation and cell growth inhibition in cancer cells. As cancer cells are characterized by an altered redox status and increased ROS compared to that of normal cells^43^,^44^,^45^,^46^, further accumulation of ROS by compounds such as JP8 may damage cancer cells while sparing normal cells (Fig. 8). This characteristic suggests a possible therapeutic strategy. Drugs that can selectively induce ROS accumulation are promising^47^,^48^, and our study supports the potential of this therapeutic strategy.

After identifying the autophagy- and ROS-inducing ability of JP8, we examined the cross-talk between these two processes and assessed whether they were both responsible for JP8-induced cell death. Pretreatment with NAC, a ROS scavenger, rescued JP8-induced cell death. Moreover, JP8 upregulated stress-related biomarkers and LC3 conversion, which were reversed upon treatment with NAC. These data indicate an upstream role of ROS in JP8-induced autophagy and cell death. In contrast, we found that inhibition of autophagy by ATG5 siRNA did not affect JP8-induced ROS accumulation, but treatment with ATG5 siRNA diminished JP8-induced cell death, albeit to a lesser extent. Stress response proteins, such as IRE1a, p-elF2a, and CHOP, were downregulated due to autophagy inhibition. These results further confirmed that autophagy acts as a downstream sensor of ROS and sequentially induces cell death. However, other signaling pathways may also contribute to JP8-induced autophagy because autophagy activation was also observed in normal cells with low ROS levels. Further in-depth studies are required to establish the mechanisms by which JP8 affect the ROS levels in cancer cells but not in normal cells.

This broad spectrum of mechanisms, including ROS and autophagy, in cancer cells may extend the therapeutic potential of JP8 in cancer treatment. Indeed, in addition to the anticancer effects observed *in vitro*, we showed that JP8 also effectively inhibited tumor growth in a B16-F10 xenograft mouse model. Moreover, the *in vivo* study further emphasized the selectivity of JP8 in inducing ROS accumulation in cancer tissues but not in normal liver tissues. Taken together, these data showed that a novel compound, JP8, exhibited antitumor effects against B16-F10 melanoma cells *in vitro* and *in vivo* via mechanisms involving both ROS and autophagy. These properties of JP8 should be further explored in the development of effective and safe anticancer agents for the treatment of melanoma.

## Experiment Section

### Chemistry

The roots of *Rhodiola crenulata* and *Rhodiola kirilowii* were collected in June 2009 from Tibet and Gansu Provinces, respectively. All chemicals were purchased from Sigma-Aldrich (St. Louis, MO, USA) and were used without further purification. The HPLC grade solvents were purchased from Thermo Fisher Scientific (Pittsburgh, PA, USA). ^1^H NMR (500 MHz) and ^13^C NMR (125 MHz) were obtained using an INOVA 500 spectrometer (Varian, Inc., Palo Alto, CA, USA). High-resolution electrospray ionization mass spectrometry (HRESIMS) was performed on an Agilent 6520 LC-Q-TOF mass spectrometer (Agilent Technologies, Waldbronn, Germany). Silica gel TLC plates (Qing Dao Marine Chemical Factory, Qingdao, China) were used to monitor the progression of the reactions. Column chromatography was performed using macroporous resin (Diaion HP-20, Mitsubishi Chemical Corp., Tokyo, Japan) and silica gel (200–400 mesh size, Qing Dao Marine Chemical Factory, Qingdao, China). The purity of the synthetic compounds was determined by HPLC analysis using an Agilent 1200 series system equipped with a DAD detector and an Apollo C18 column (250 mm × 4.6 mm, 5 *μ*m, Alltech Corp., Kentucky, USA). Preparative high-performance liquid chromatography (PHPLC) was performed using a Shimadzu LC-6AD instrument with a SPD-20A detector (Shimadzu Corp., Tokyo, Japan) and a YMC-Pack ODS-A column (250 mm × 20 mm, 5 *μ*m, YMC Corp., Kyoto, Japan).

### Preparation of ECGp and EGCGp

Air-dried roots (20 kg) of *Rhodiola crenulata* were extracted with 80% EtOH. After the solvent was evaporated under reduced pressure, the residue was resuspended in H_2_O (5000 mL) and extracted with EtOAc (3 × 5000 mL). The aqueous layer was applied to a HP-20 macroporous adsorbent resin (4000 g, dried weight) column. Successive elution from the column with H_2_O, 15% EtOH, 30% EtOH, 50% EtOH, 70% EtOH and 95% EtOH (15 L each) yielded five corresponding fractions after removing the solvents. Among them, the 50% EtOH portion was selected as the reactant (ECGp). Using the same method as that for *Rhodiola crenulata*, we obtained the 50% EtOH fraction of *Rhodiola kirilowii* (EGCGp).

### General procedures for the synthesis of compounds 1–57

ECGp/EGCGp (2.0 g) were dissolved in methanol (60 mL) and placed in a 200 mL round-bottomed flask. The reaction mixture was stirred at 60 °C and acidified with 48% HBr (2 mL). Then, the thiol reagent (1.0 g) was added. After four hours, the mixture was diluted with H_2_O (90 mL) and extracted with EtOAc (3 × 150 mL). The EtOAc layer was dried over anhydrous Na_2_SO_4_. The solvent was concentrated to a slurry, which was chromatographically separated on a silica gel and further purified by the reverse-phase PHPLC to obtain the product.

### Animals

Male C57BL/6 mice (6- to 8-week-old) were obtained from Vital River Laboratory Animal Technology (Beijing, China) and maintained in the Laboratory Animal Center at the Chinese Academy of Medical Sciences. All animal procedures were approved by the Institutional Animal Care and Use Committee of the Chinese Academy of Medical Sciences and Peking Union Medical College (Permit No. 002802). All protocols were in accordance with the approved guidelines and regulations.

### Antibodies and reagents

Anti-LC3 I/II and anti-p62 antibodies were purchased from Sigma-Aldrich (St. Louis, MO, USA). Anti-ATG5 antibodies were purchased from Medical & Biological Laboratories (Japan). Anti-GAPDH antibodies were purchased from Kangchen Bio-tech (Shanghai, China). All other antibodies were obtained from Cell Signaling. Saponin and cycloheximide (CHX) were purchased from Sigma-Aldrich (St. Louis, MO, USA). Rapamycin, bafilomycin A1 and geneticin were purchased from Calbiochem (San Diego, CA, USA). Lipofectamine transfection reagents were purchased from Thermo Fisher.

### Cell culture

B16-F10 mouse melanoma cells, AML-12 mouse hepatocytes, and 16HBE human bronchial epithelial cells were maintained in 1640 medium (Thermo Fisher) containing 10% FBS (Thermo Fisher) and 1% penicillin/streptomycin (Thermo Fisher) in a 5% CO_2_ atmosphere at 37 °C. A549 human lung carcinoma cells were maintained in Dulbecco’s modified Eagle’s medium (DMEM) (Thermo Fisher) containing 10% FBS. MDA-MB-231 human breast carcinoma cells were maintained in Leibovitz’s L-15 medium (Thermo Fisher) containing 10% FBS. HMLE human breast epithelial cells were maintained in DMEM: nutrient mixture F-12 (DMEM/F12) (Thermo Fisher) containing 10% FBS.

Chinese hamster ovary (CHO) cells stably expressing GFP-LC3 were generated by transfection with a GFP-LC3 plasmid (OriGene Technologies, Rockville, MD, USA) using Lipofectamine 2000 (Thermo Fisher). Stable clones of GFP-LC3-transfected cells were selected in 1 mg/ml geneticin (G418).

For transfection with ATG-5 siRNA (RiboBio, Beijing, China), Lipofectamine RNAiMAX (Thermo Fisher) was used according to the manufacturer’s instructions. Briefly, cells were seeded in 6-well plates. RNA and RNAiMAX were diluted in Opti-MEM and incubated for 5 min, and the transfection mix was then added to the culture media.

### Compounds screening for autophagy activators

CHO cells stably expressing GFP-LC3 were incubated with different compounds (10 *μ*M) for 12 h in 12-well plates. Rapamycin (0.1 *μ*M) was used as a positive control. Cells were harvested and washed with PBS and then with PBS containing 0.05% saponin. GFP-LC3 fluorescence was measured using a flow cytometer (Partec, Muenster, Germany) with more than 10,000 events. Data were analyzed with FCS Express 4. Each experiment was performed in duplicate, and compounds were excluded if duplicates differed by more than 10%.

### Western blot analysis

Immunoblotting was performed following standard procedures. The cells were lysed in RIPA lysis buffer (Beyotime, Nanjing, China) with a phosphatase inhibitor mixture (Thermo Fisher) and a protease inhibitor mixture (Roche Applied Science, Penzberg, Upper Bavaria, Germany). An equal amount of protein was resolved on SDS-PAGE and transferred onto polyvinylidene difluoride membranes (Merck Millipore, Darmstadt, Germany). After the membrane was blocked with 5% nonfat milk, the membrane was probed with the designated first and second antibodies, developed with a chemiluminescence substrate (Merck Millipore) and visualized by a LAS4000 Image Station (General Electric Company, Fairfield, CT, USA).

### Cell viability assay

The cell viability was determined using the Cell Counting Kit-8 (CCK-8) (Dojindo Laboratories, Kumamoto, Japan). Cells were seeded at a density of 2000 cells/well in 96-well plate and treated with JP8 at various concentrations for 48 h. Cells were then exposed to 10 μL of the CCK-8 reagent for 1 h at 37 °C. The absorbance at 450 nm was measured using a microplate reader. The viability of untreated cells was considered to be 100%.

### Apoptosis assay

Cells were seeded in 12-well plates and treated with JP8 or DMSO. After incubation, cells were harvested and washed twice with PBS. After the cells were stained with annexin V-FITC and PI (KenGEN Biotech, Nanjing, Jiangsu, China) at room temperature for 15 min, they were analyzed with a flow cytometer (Partec). Annexin V-FITC-positive cells were identified as apoptotic cells.

### DiOC6/PI double staining for apoptosis detection

Cells were stained with both DiOC6 and PI at final concentrations of 40 nM and 1 *μ*g/mL respectively. After 15 min incubation at 37 °C, stained samples were analyzed by flow cytometry. DiOC6 is in FL1 and PI is in FL2.

### Live-cell imaging for autophagic flux

mRFP-GFP-LC3 adenoviral (Hanbio, Shanghai, China)-infected B16 cells were grown at 37 °C on single thickness 8-well Lab-Tek chamber glass slides (Nunc). After a 12 h incubation with JP8 (20 *μ*M) or DMSO, imaging was performed on an UltraVIEW VoX 3D Live Cell Imaging System^49^. All image acquisition settings were kept the same during the image collection. Volocity Demo 5.4 software was used for image processing and analysis. For collecting images to prepare videos, images were taken at 5 second intervals, processed and merged using ImageJ software, and the images were exported as ‘.avi’ files with 4 frames per second.

### Measurement of intracellular ROS

Cells were treated with JP8 or vehicle control DMSO for 12 h, and ROS generation was detected with dihydroethidium (DHE) (Vigorous Biotechnology, Beijing, China). Cells were harvested and washed once with PBS. Cells were incubated with 5 *μ*M DHE at 37 °C for 15 min, washed once with PBS and immediately assessed by flow cytometry (Partec). Data were analyzed with FCS Express 4 software.

### Immunofluorescence

Frozen liver and tumor sections were incubated with 5 mM DHE (Vigorous Biotechnology, Beijing, China) at 37 °C for 30 min. After two washes with PBS, slides were sealed with cover slips. The ROS level in the tissue was measured by a Leica SP2 confocal microscope (Leica Microsystems, Wetzlar, Germany).

### *In vivo* antitumor assay

Mice were inoculated subcutaneously (s.c.) with 3 × 10^5^ viable B16F10 cells in the right flank. JP8 was dissolved in PBS containing 20% PEG400 (Sinopharm Chemical Reagent, Shanghai, China). From day 3, JP8 was i.p. administered at 3 different doses (10, 25 and 50 mg/kg). The untreated tumor control group received 100 *μ*L of PBS containing 20% PEG400. After administration of JP8, tumor size was measured daily with a caliper. Tumor volume (V) was calculated by the following formula: V = 0.5 × d2 × D; where d and D are the short and long diameters of the tumor, respectively. Animals were sacrificed at day 23. Tumors and livers were excised and then fixed or frozen for morphological evaluation or measurement of ROS content.

### Statistics

Statistical analysis was performed using GraphPad Prism 5.0 software (La Jolla, CA, USA). Data are represented as the mean ± SEM. Two-tailed Student’s t-tests were used to determine significance (*P < 0.05, **P < 0.005, ***P < 0.001, and ****P < 0.0001).

## Additional Information

**How to cite this article**: Xie, J. *et al*. A novel ECG analog 4-(S)-(2,4,6-trimethylthiobenzyl)-epigallocatechin gallate selectively induces apoptosis of B16-F10 melanoma via activation of autophagy and ROS. *Sci. Rep.* **7**, 42194; doi: 10.1038/srep42194 (2017).

**Publisher's note:** Springer Nature remains neutral with regard to jurisdictional claims in published maps and institutional affiliations.

## Supplementary Material

Supplementary Video 1

Supplementary Video 2

Supporting Information

## Figures and Tables

**Table 1:**
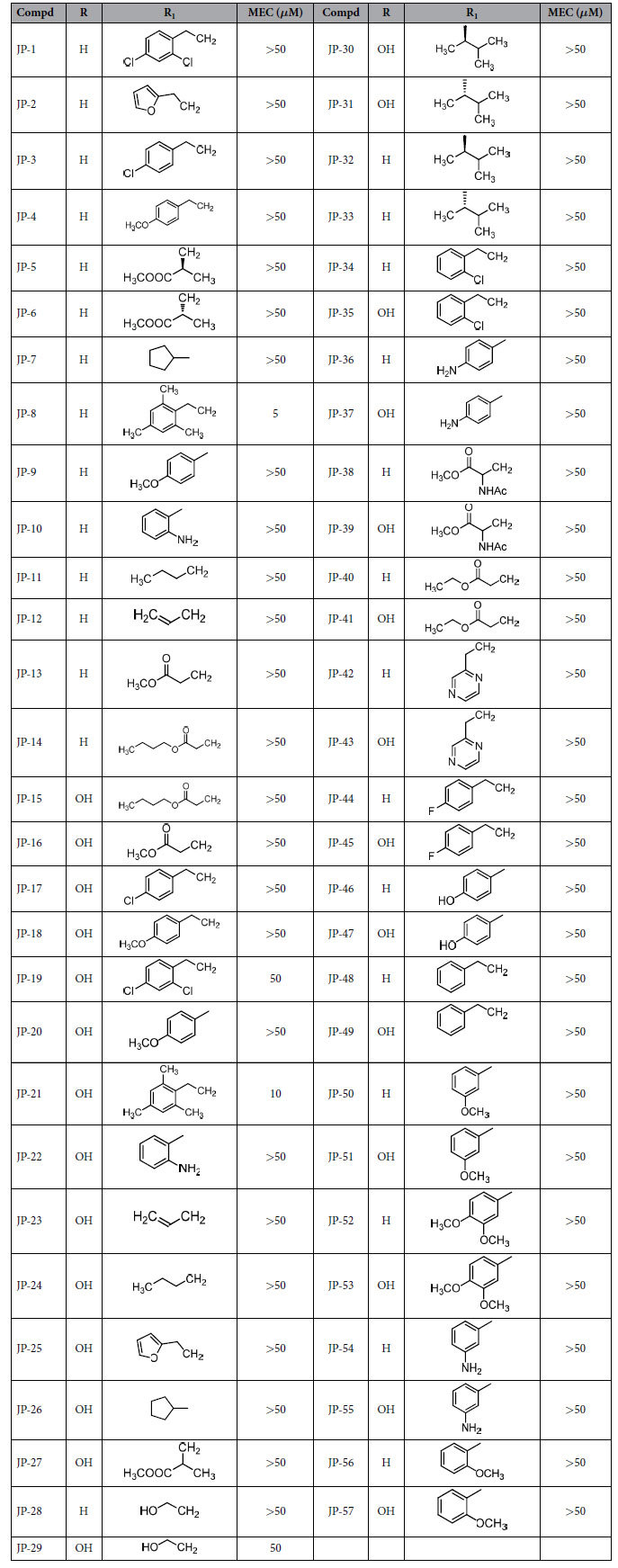
ECG and EGCG derivatives and their autophagic activation.
