# Supplementary material for: A novel ECG analog 4-(S)-(2,4,6-trimethylthiobenzyl)-epigallocatechin gallate selectively induces apoptosis of B16-F10 melanoma via activation of autophagy and ROS
Source: Sci Rep. 2017 Feb 10;7:42194. doi: 10.1038/srep42194 (PMC5301500; doi:10.1038/srep42194)
Supplement: Supporting Information [file srep42194-s3.pdf]

A novel ECG analog 4-(*S*)-(2,4,6-trimethylthiobenzyl)-epigallocatechin gallate selectively induces apoptosis of B16-F10 melanoma via activation of autophagy and ROS

Jing Xie[1,3,*], Ju-ping Yun[1,*], Ya-nan Yang[1,*], Fang Hua[1], Xiao-wei Zhang[1], Heng Lin[1], Xiao-xi Lv[1], Ke Li[2], Pei-cheng Zhang[1,#] & Zhuo-wei Hu[1,#]

[1] State Key Laboratory of Bioactive Substances and Functions of Natural Medicines, Institute of Materia Medica, Chinese Academy of Medical Sciences and Peking Union Medical College, Beijing 100050, People's Republic of China

[2] Laboratory of Antiviral Research, Institute of Medicinal Biotechonology; Chinese Academy of Medical Sciences & Peking Union Medical College, Beijing 100050, People's Republic of China

[3] Department of Pharmacy, China-Japan Friendship Hospital, Beijing 100050, People's Republic of China

*These authors contributed equally to this work.

[#]Correspondence and requests for materials should be addressed to P. C. Z. (email: pczhang@imm.ac.cn) and Z. W. H. (email: huzhuowei@imm.ac.cn)

SUPPORTING INFORMATION

1. ^{1}H NMR and ^{13}C NMR spectra of compounds 1-57.

2. The reaction yield of compounds 1-57.

3. Spectra data for 1-57.

4. Supplementary figures S170-177 and legend.

5. Video 1: Representative video of live cells infected with GFP-RFP-LC3 adenoviral without the presence of JP8 (20 μM) for 12 h.

6. Video 2: Representative video of live cells infected with GFP-RFP-LC3 adenoviral in the presence of JP8 (20 μM) for 12 h.

1. ^{1}HNMR and ^{13}CNMR spectra of compounds 1-57

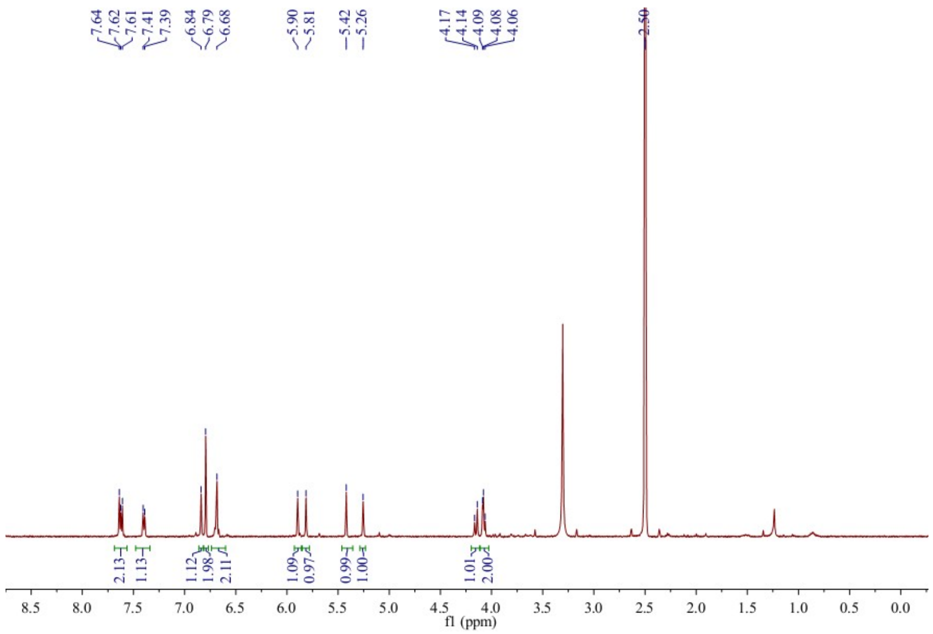

Figure S1. ^{1}H NMR of compound **JP-1** (DMSO-d_6)

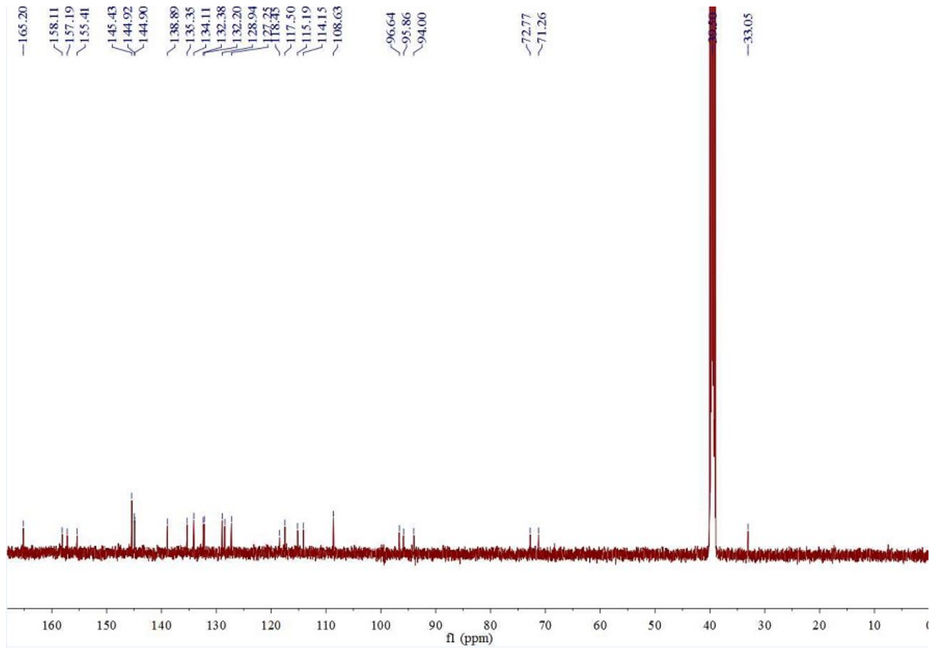

Figure S2. ^{13}C NMR of compound **JP-1** (DMSO-d_6)

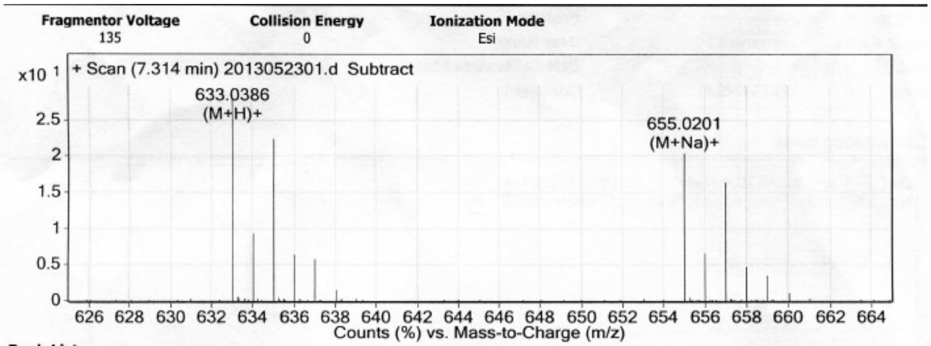

Peak List

m/z	z	Abund	Formula	Ion
158.1537	1	88421		
172.1695		83289		
277.1776	1	54292		
309.2036	1	183367		
310.2065	1	35123		
317.1724		40452		
423.1995	1	68382		
633.0386	1	51804	C29 H23 Cl2 O10 S	(M+H)+
635.0371	1	41003	C29 H23 Cl2 O10 S	(M+H)+
655.0201	1	37316	C29 H22 Cl2 Na O10 S	(M+Na)+

Figure S3. HRESIMS of compound **JP-1**

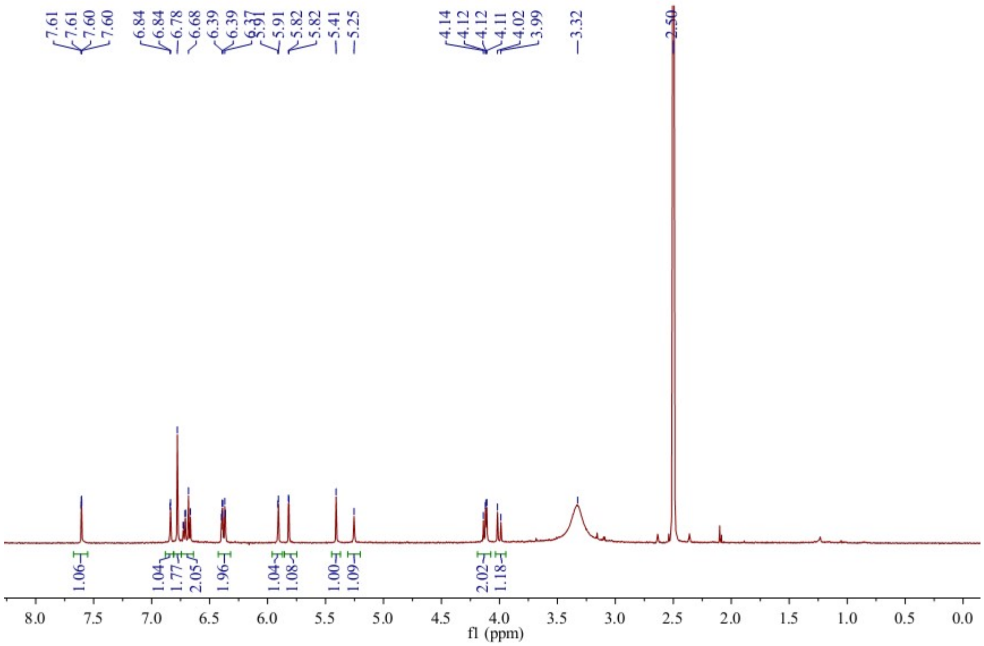

Figure S4. ^{1}H NMR of compound **JP-2** (DMSO-d_6)

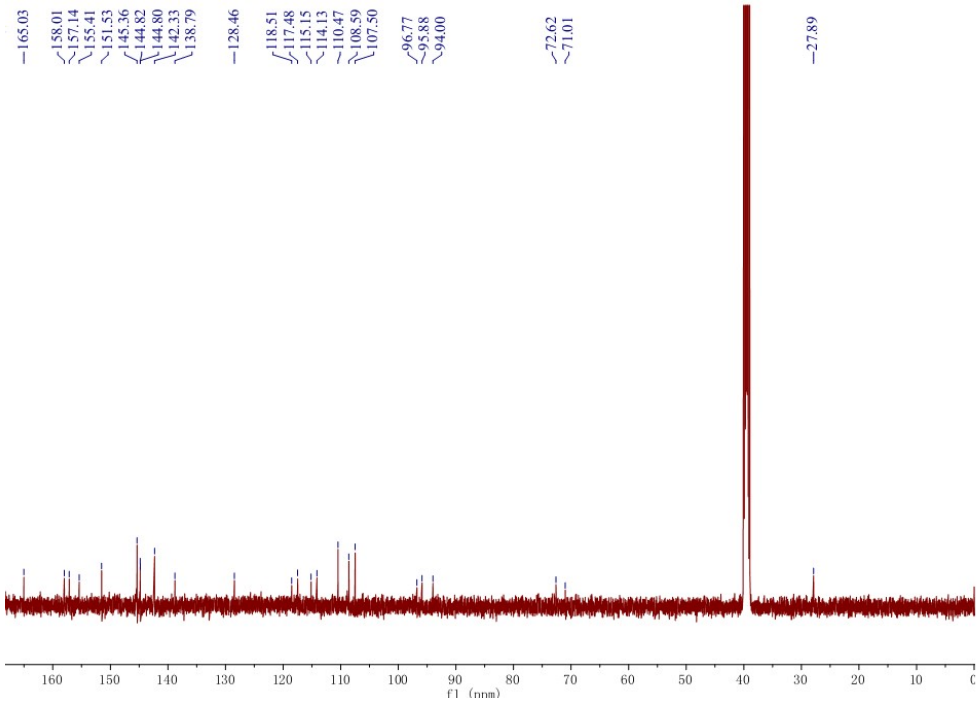

Figure S5. ^{13}C NMR of compound **JP-2** (DMSO-d_6)

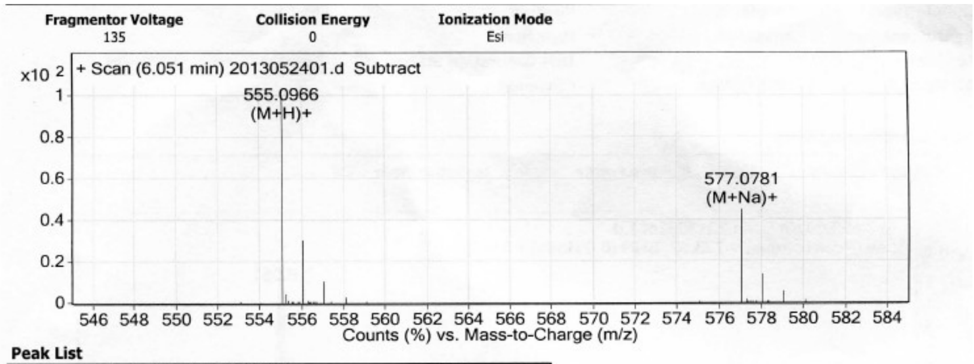

Peak List

m/z	z	Abund	Formula	Ion
181.1206		35143		
209.12		38910		
299.115		44688		
385.0751		36975		
441.0819		84956		
555.0966	1	436037	C27 H23 O11 S	(M+H)+
556.0995	1	132031	C27 H23 O11 S	(M+H)+
557.098	1	45706	C27 H23 O11 S	(M+H)+
577.0781	1	195528	C27 H22 Na O11 S	(M+Na)+
578.0812	1	58177	C27 H22 Na O11 S	(M+Na)+

Figure S6. HRESIMS of compound **JP-2**

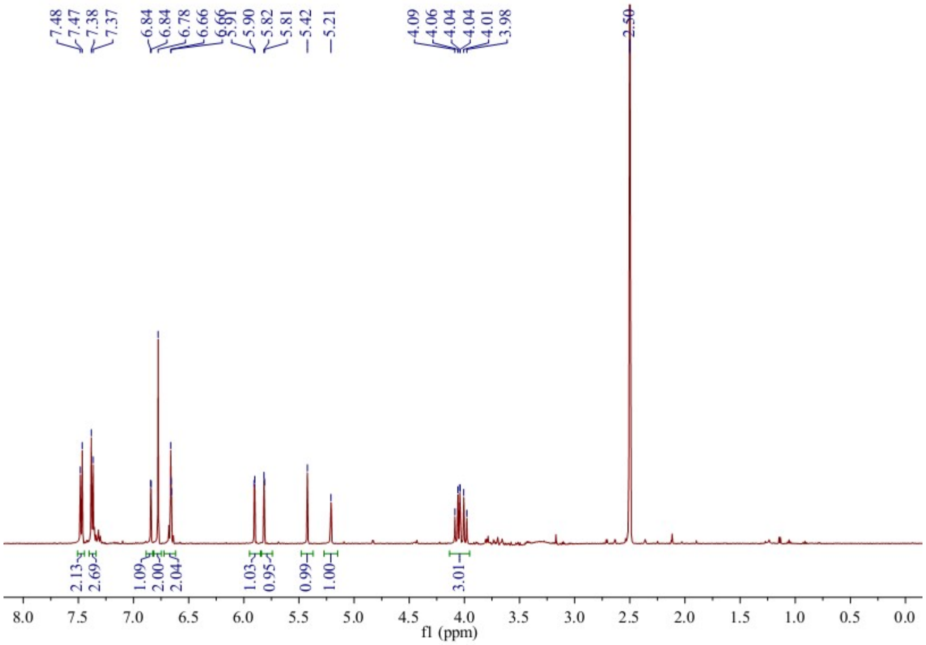

Figure S7. ^{1}H NMR of compound **JP-3** (DMSO-d_6)

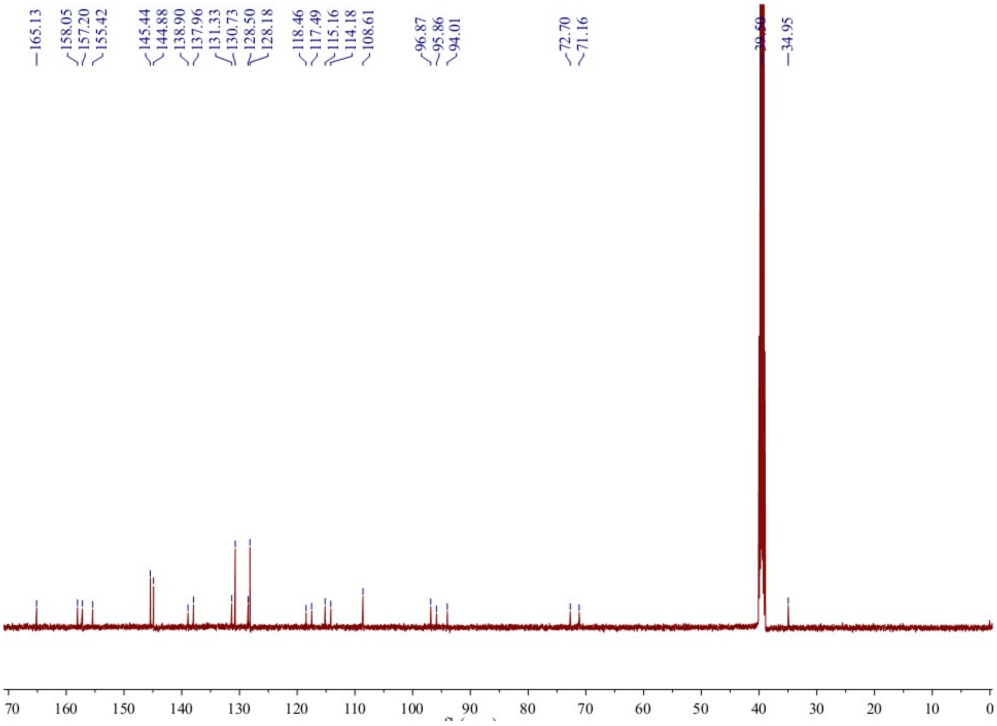

Figure S8. ^{13}C NMR of compound **JP-3** (DMSO-d_6)

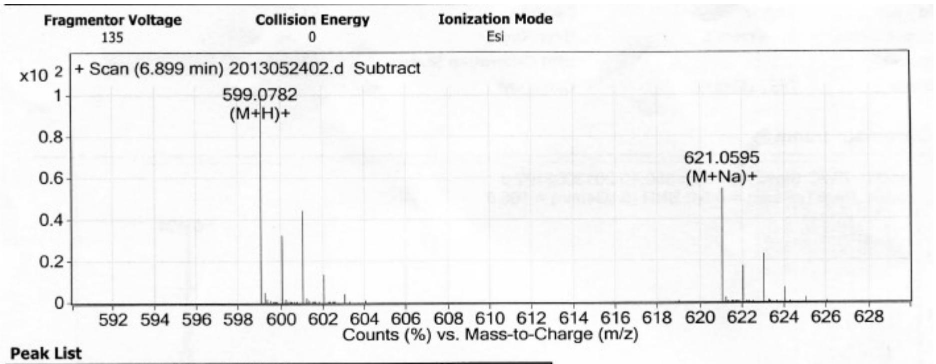

Peak List

m/z	z	Abund	Formula	Ion
158.154	1	580226		
180.1356		210784		
441.0819	1	186755		
599.0782	1	733461	C29 H24 Cl O10 S	(M+H)+
600.0811	1	237446	C29 H24 Cl O10 S	(M+H)+
601.0762	1	324592	C29 H24 Cl O10 S	(M+H)+
602.078	1	97569	C29 H24 Cl O10 S	(M+H)+
621.0595	1	401634	C29 H23 Cl Na O10 S	(M+Na)+
622.0598	1	126345	C29 H23 Cl Na O10 S	(M+Na)+
623.0571	1	170362	C29 H23 Cl Na O10 S	(M+Na)+

Figure S9. HRESIMS of compound **JP-3** (DMSO-d_6)

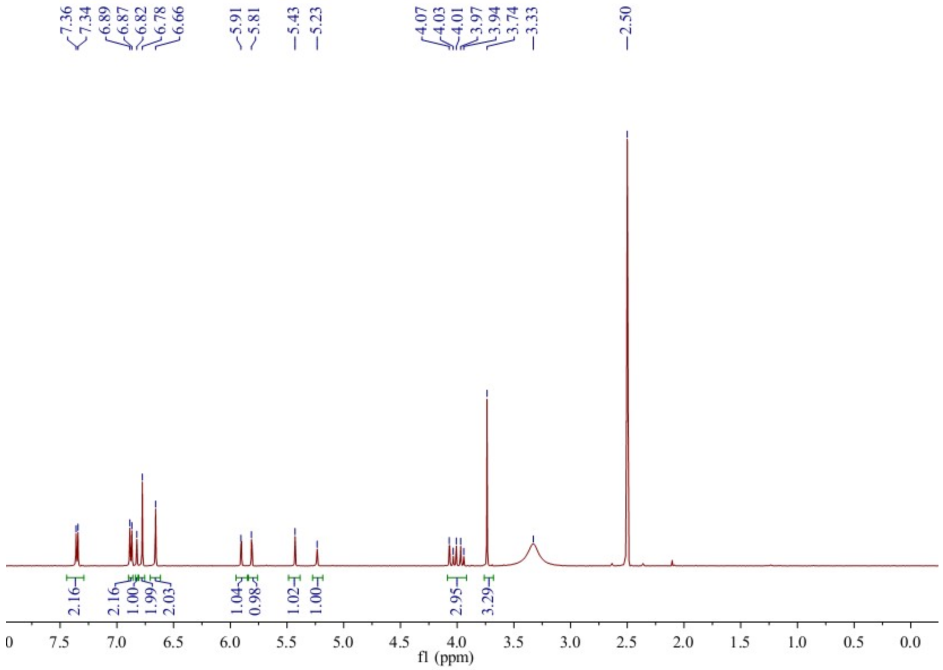

Figure S10. ^{1}H NMR of compound **JP-4** (DMSO-d_6)

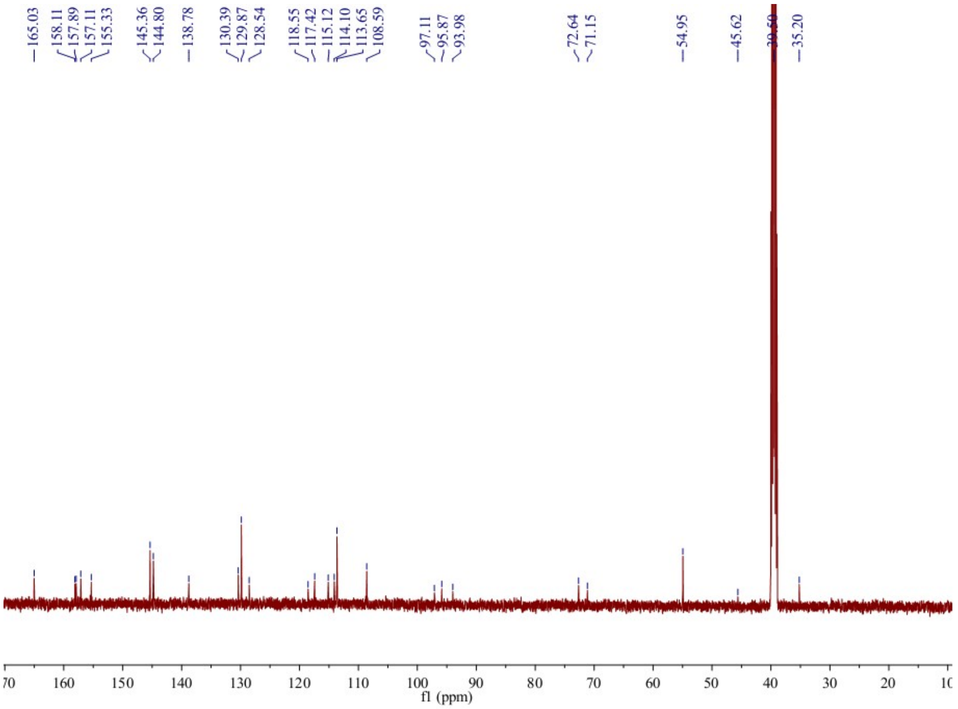

Figure S11. ^{13}C NMR of compound **JP-4** (DMSO-d_6)

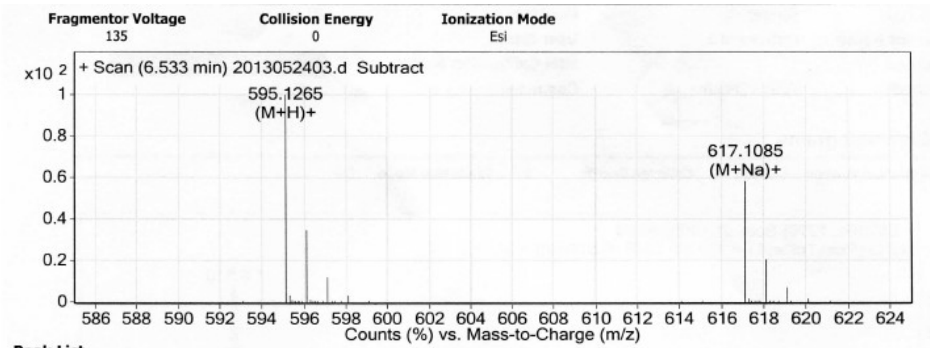

Peak List

m/z	z	Abund	Formula	Ion
116.9851		17518		
241.1406		19920		
425.1048		21770		
441.0803	1	59868		
595.1265	1	276376	C30 H27 O11 S	(M+H)+
596.1296	1	95615	C30 H27 O11 S	(M+H)+
597.1287	1	33488	C30 H27 O11 S	(M+H)+
617.1085	1	161050	C30 H26 Na O11 S	(M+Na)+
618.1113	1	56525	C30 H26 Na O11 S	(M+Na)+
619.1099	1	19414	C30 H26 Na O11 S	(M+Na)+

Figure S12. HRESIMS of compound **JP-4**

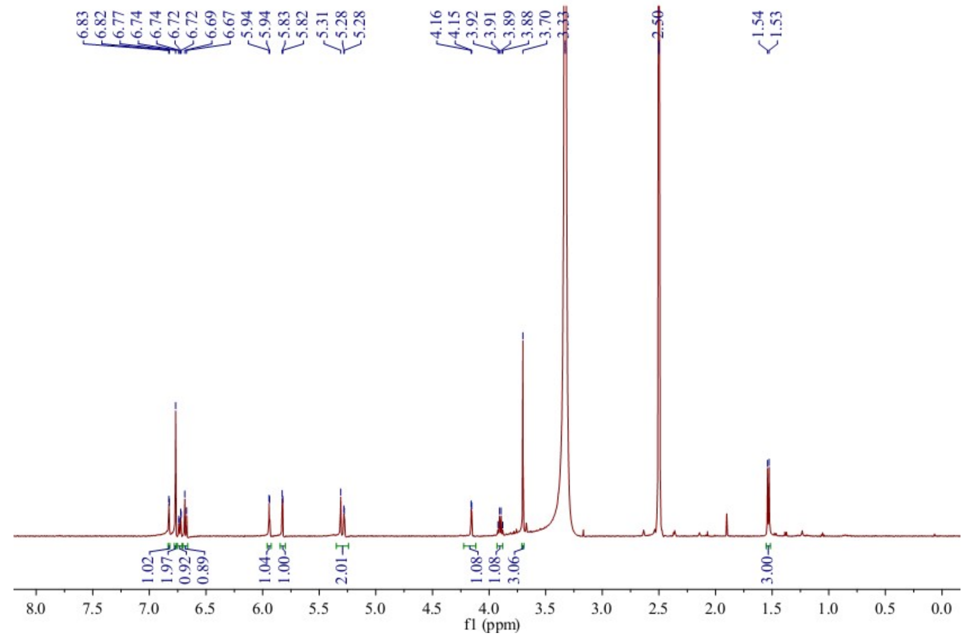

Figure S13. ^{1}H NMR of compound **JP-5** (DMSO-d_6)

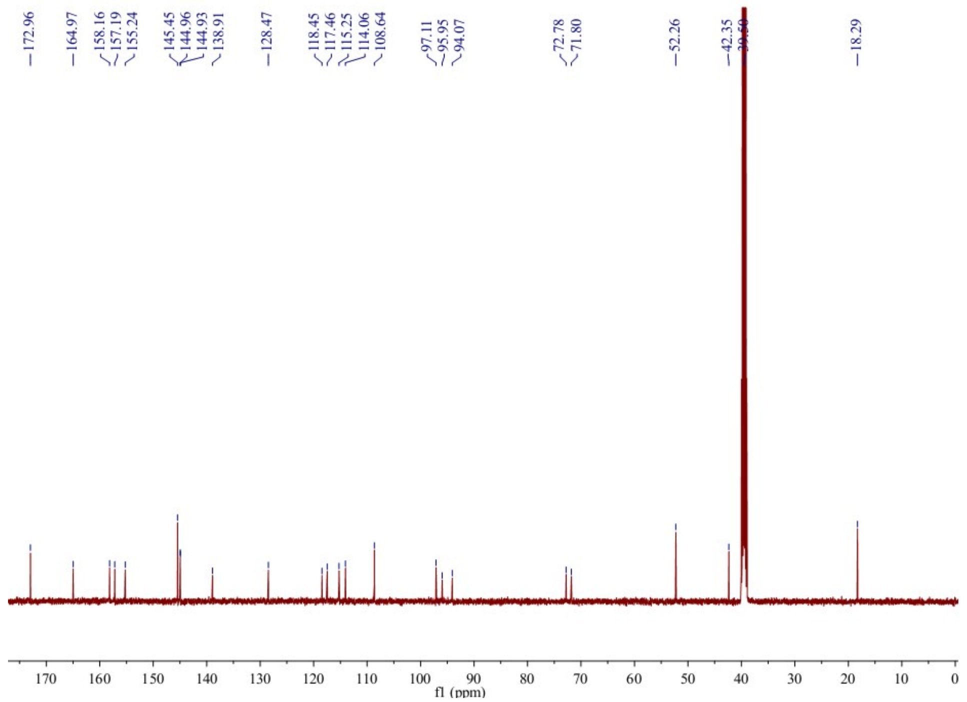

Figure S14. ^{13}C NMR of compound **JP-5** (DMSO-d_6)

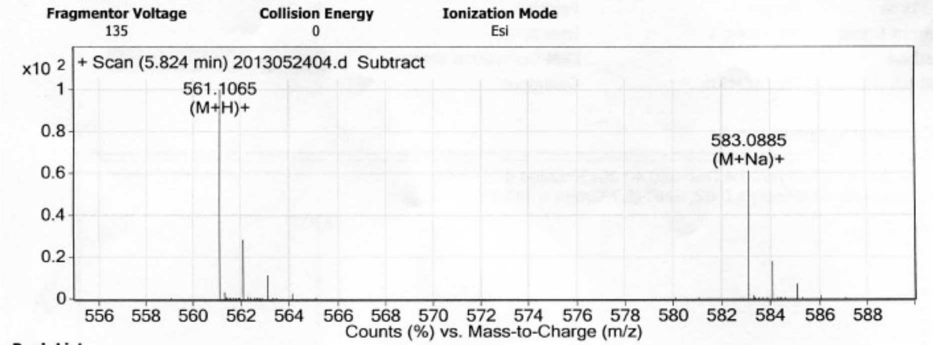

Peak List

m/z	z	Abund	Formula	Ion
211.1382		30670		
251.1627		69178		
284.9615		32497		
286.959		32638		
441.082	1	62869		
561.1065	1	233457	C26 H25 O12 S	(M+H)+
562.1102	1	66188	C26 H25 O12 S	(M+H)+
563.108	1	26554	C26 H25 O12 S	(M+H)+
583.0885	1	143278	C26 H24 Na O12 S	(M+Na)+
584.0909	1	41527	C26 H24 Na O12 S	(M+Na)+

Figure S15. HRESIMS of compound **JP-5**

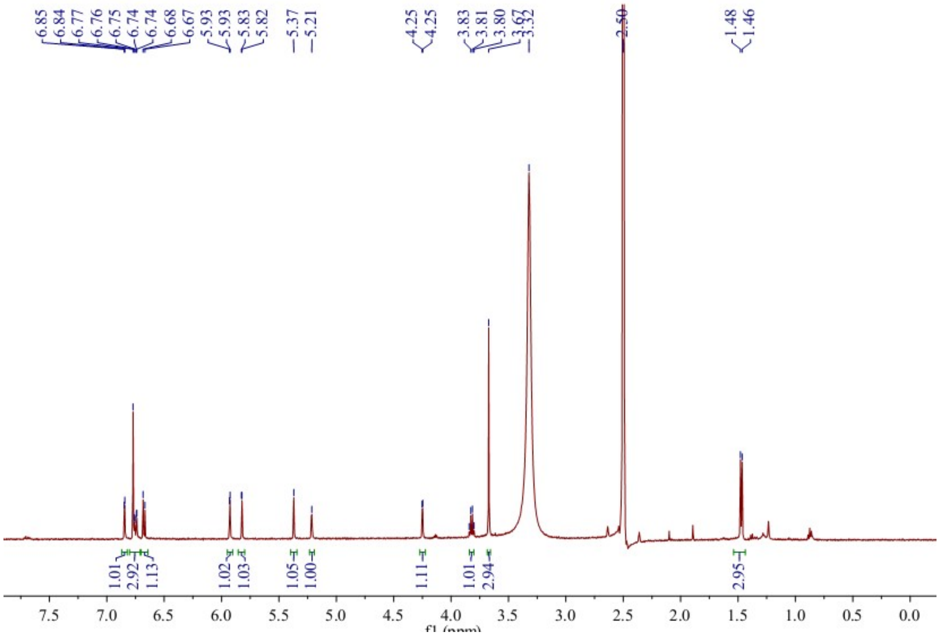

Figure S16. ^{1}H NMR of compound **JP-6** (DMSO-d_6)

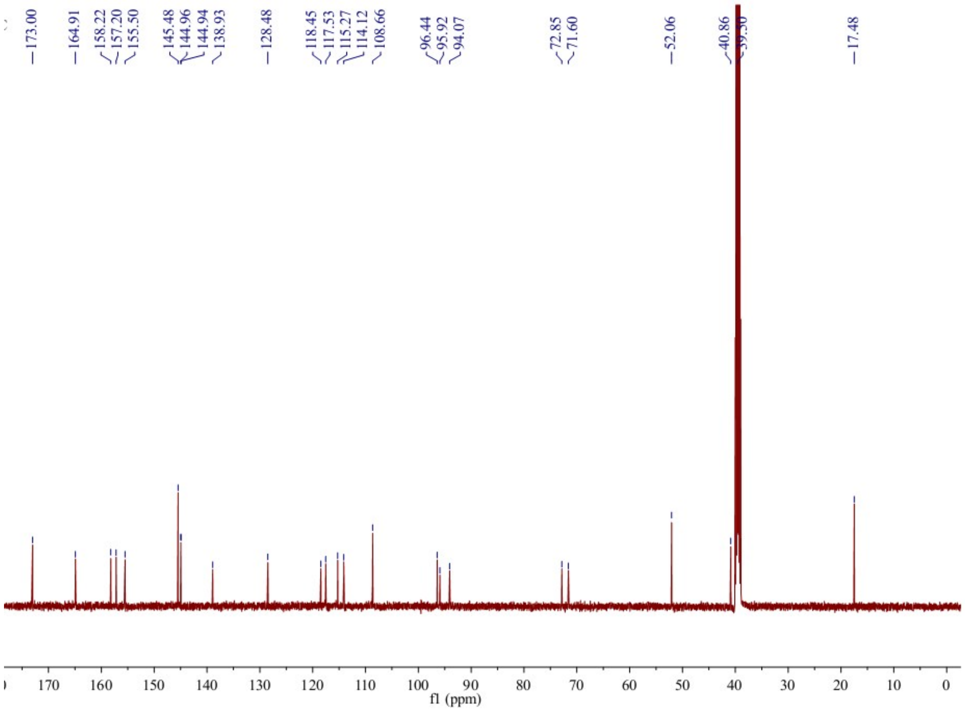

Figure S17. ^{13}C NMR of compound **JP-6** (DMSO-d_6)

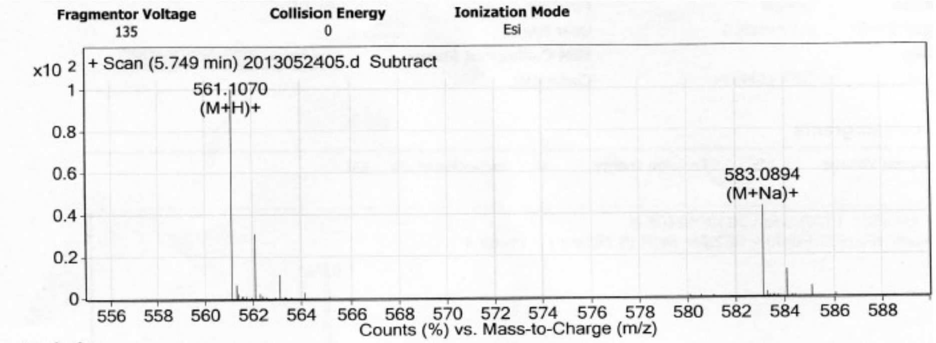

Peak List

m/z	z	Abund	Formula	Ion
441.0823		249542		
561.107	1	1276809	C26 H25 O12 S	(M+H)+
561.3023		79745		
562.1107	1	393299	C26 H25 O12 S	(M+H)+
563.109	1	147362	C26 H25 O12 S	(M+H)+
583.0894	1	550943	C26 H24 Na O12 S	(M+Na)+
584.0923	1	163296	C26 H24 Na O12 S	(M+Na)+
1143.1875	1	296772		
1144.1899	1	178845		
1145.189	1	94728		

Figure S18. HRESIMS of compound **JP-6**

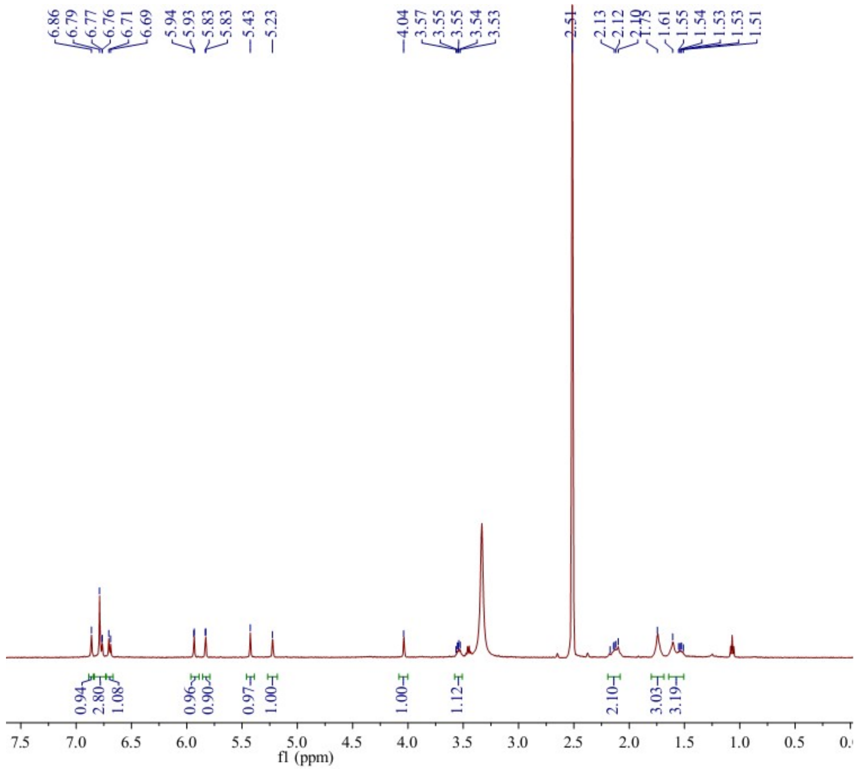

Figure S19. ^{1}H NMR of compound **JP-7** (DMSO-d_6)

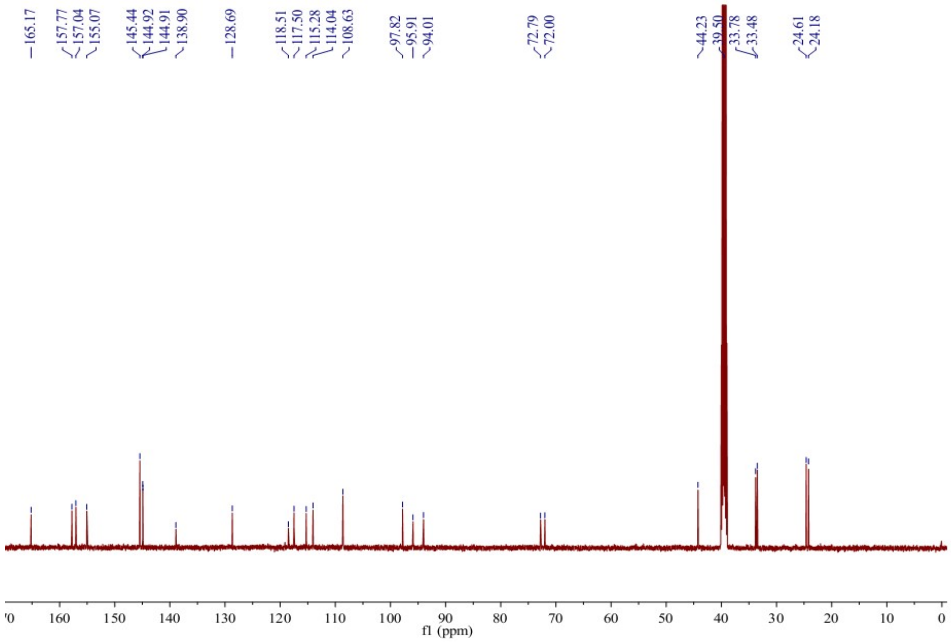

Figure S20. ^{13}C NMR of compound **JP-7** (DMSO-d_6)

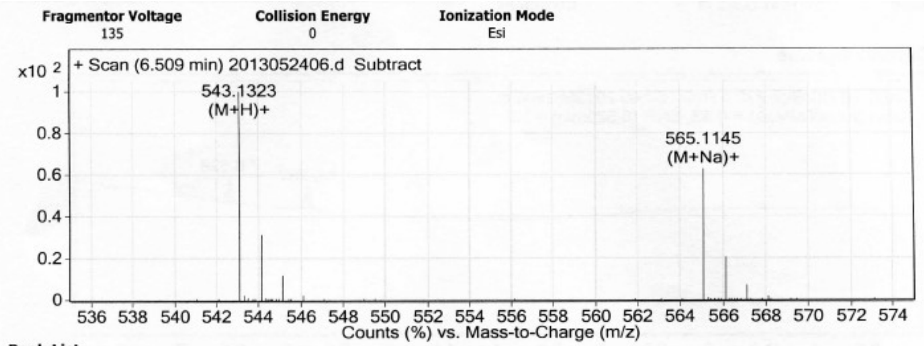

Peak List

m/z	z	Abund	Formula	Ion
116.9861		20876		
125.9864		14378		
130.1592		14976		
241.1417		13347		
441.0823	1	21551		
543.1323	1	78782	C27 H27 O10 S	(M+H)+
544.1366	1	24781	C27 H27 O10 S	(M+H)+
545.1339	1	9318	C27 H27 O10 S	(M+H)+
565.1145	1	49338	C27 H26 Na O10 S	(M+Na)+
566.1185	1	15998	C27 H26 Na O10 S	(M+Na)+

Figure S21. HRESIMS of compound **JP-7**

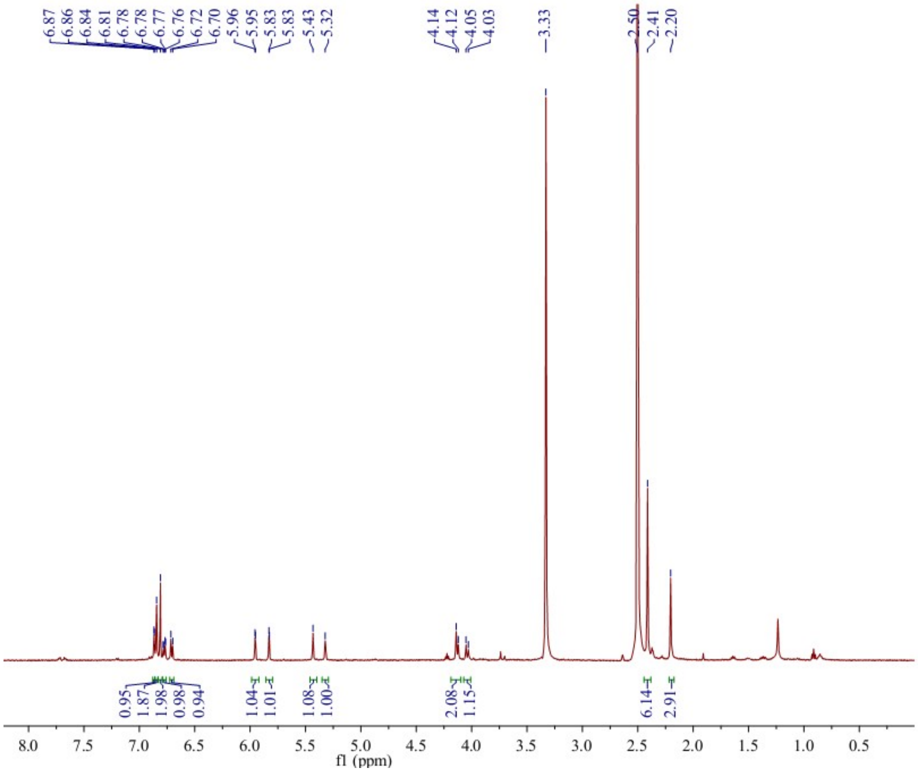

Figure S22. ^{1}H NMR of compound **JP-8** (DMSO-d_6)

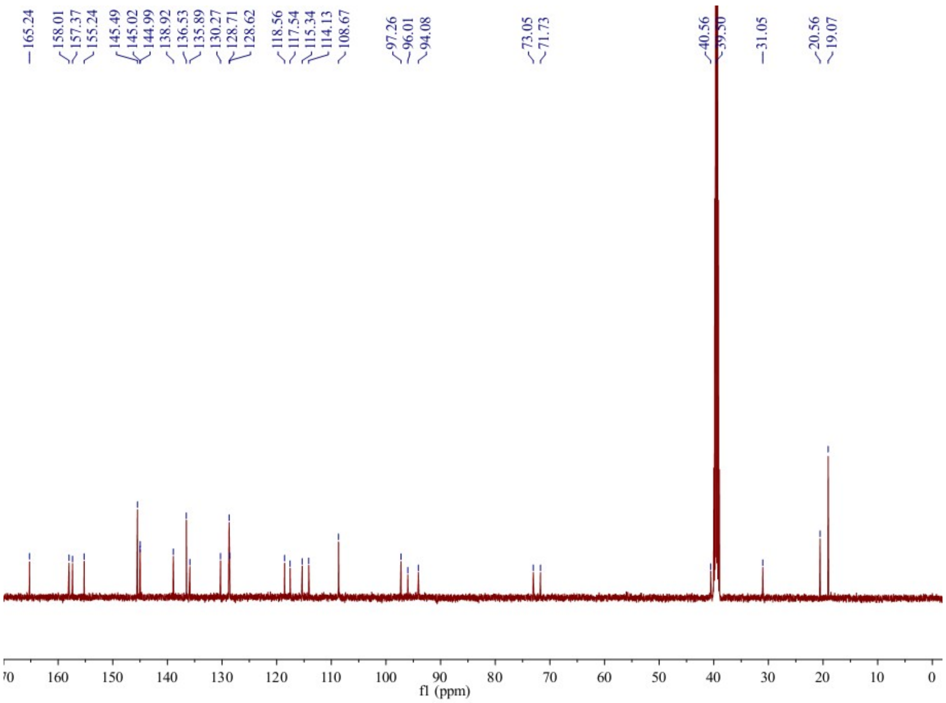

Figure S23. ^{13}C NMR of compound **JP-8** (DMSO-d_6)

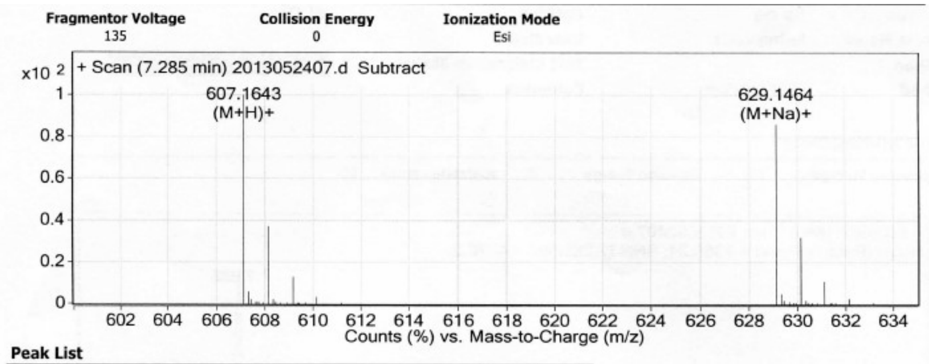

Peak List

m/z	z	Abund	Formula	Ion
271.0599		82905		
437.142		147683		
441.0823	1	325274		
607.1643	1	1006668	C32 H31 O10 S	(M+H)+
608.1677	1	376376	C32 H31 O10 S	(M+H)+
609.1668	1	130690	C32 H31 O10 S	(M+H)+
629.1464	1	864510	C32 H30 Na O10 S	(M+Na)+
630.1491	1	321265	C32 H30 Na O10 S	(M+Na)+
631.1479	1	106466	C32 H30 Na O10 S	(M+Na)+
881.1558	1	112834		

Figure S24. HRESIMS of compound **JP-8** (DMSO-d_6)

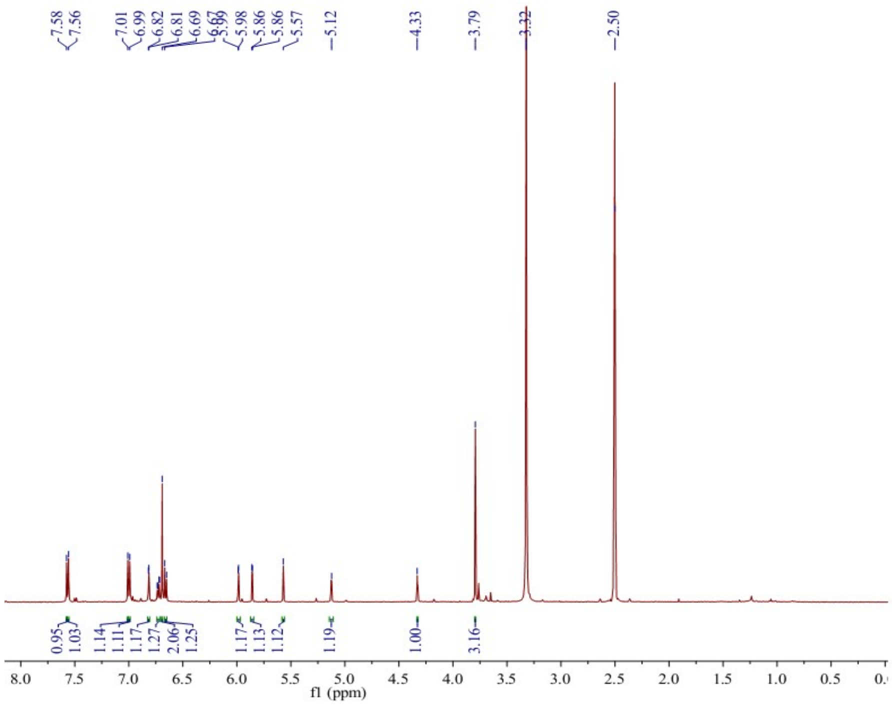

Figure S25. ^{1}H NMR of compound **JP-9** (DMSO-d_6)

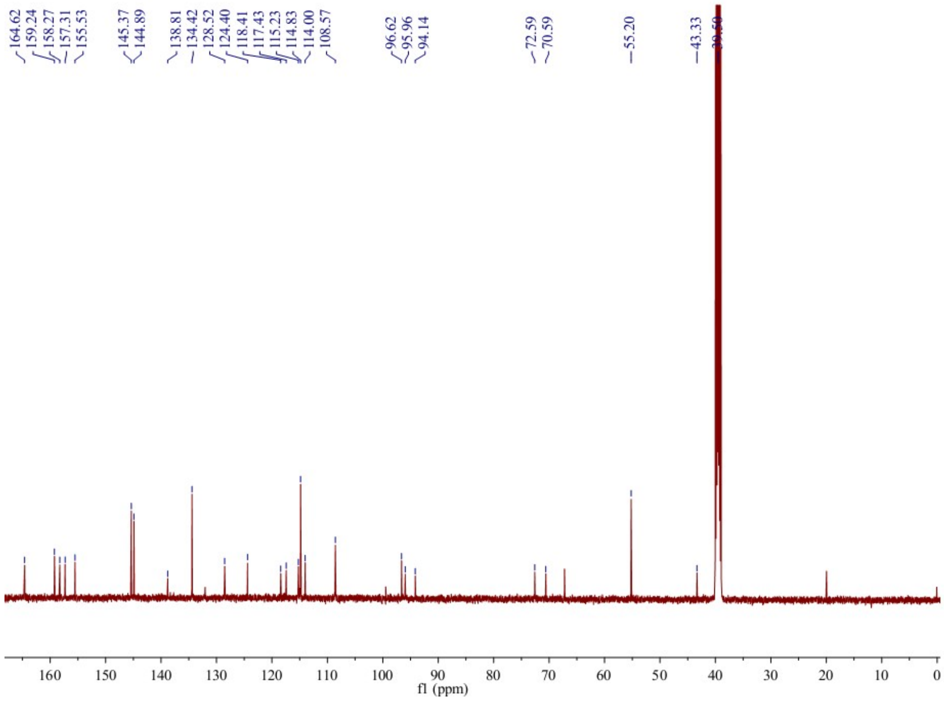

Figure S26. ^{13}C NMR of compound **JP-9** (DMSO-d_6)

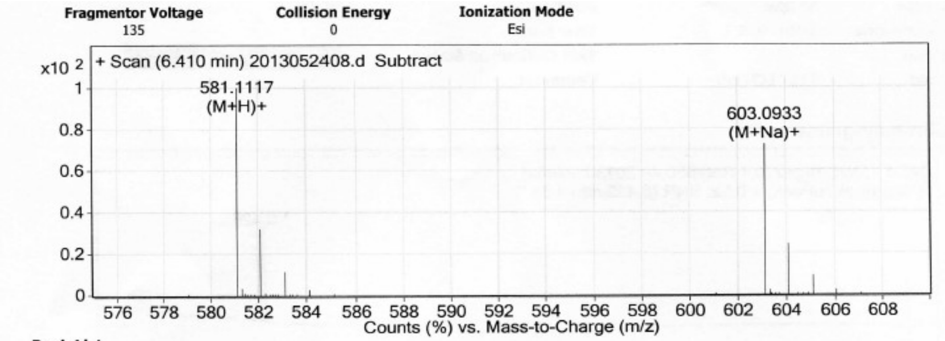

Peak List

m/z	z	Abund	Formula	Ion
116.9861		21383		
411.0908		28223		
441.0824	1	115543		
442.0848	1	28020		
581.1117	1	231439	C29 H25 O11 S	(M+H)+
582.1145	1	73361	C29 H25 O11 S	(M+H)+
583.1138	1	26775	C29 H25 O11 S	(M+H)+
603.0933	1	167462	C29 H24 Na O11 S	(M+Na)+
604.0961	1	55920	C29 H24 Na O11 S	(M+Na)+
605.0958	1	21732	C29 H24 Na O11 S	(M+Na)+

Figure S27. HRESIMS of compound **JP-9**

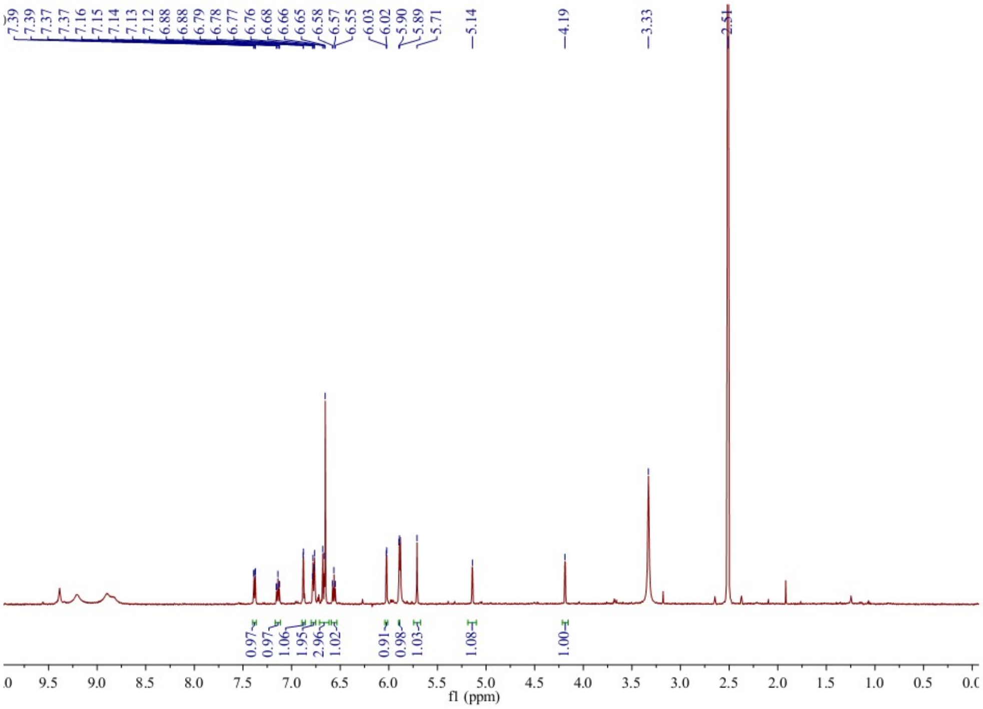

Figure S28. ^{1}H NMR of compound **JP-10** (DMSO-d_6)

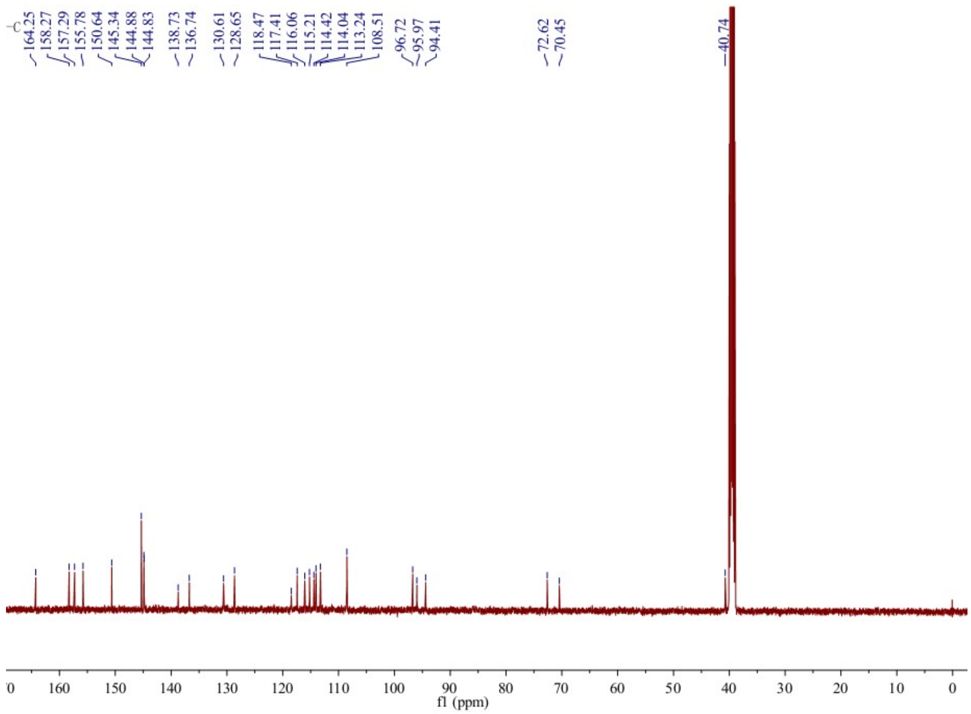

Figure S29. ^{13}C NMR of compound **JP-10** (DMSO-d_6)

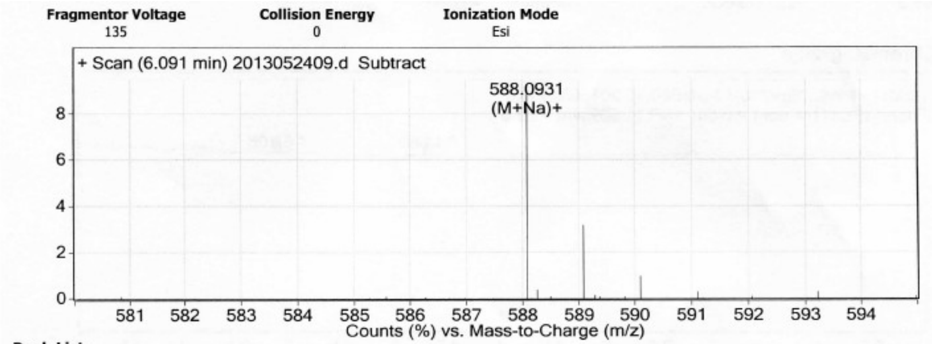

Peak List

m/z	z	Abund	Formula	Ion
116.9863		20676		
271.0618		15476		
441.0825	1	279169		
442.0858	1	62799		
588.0931		25182	C28 H23 N Na O10 S	(M+Na)+

Figure S30. HRESIMS of compound **JP-10**

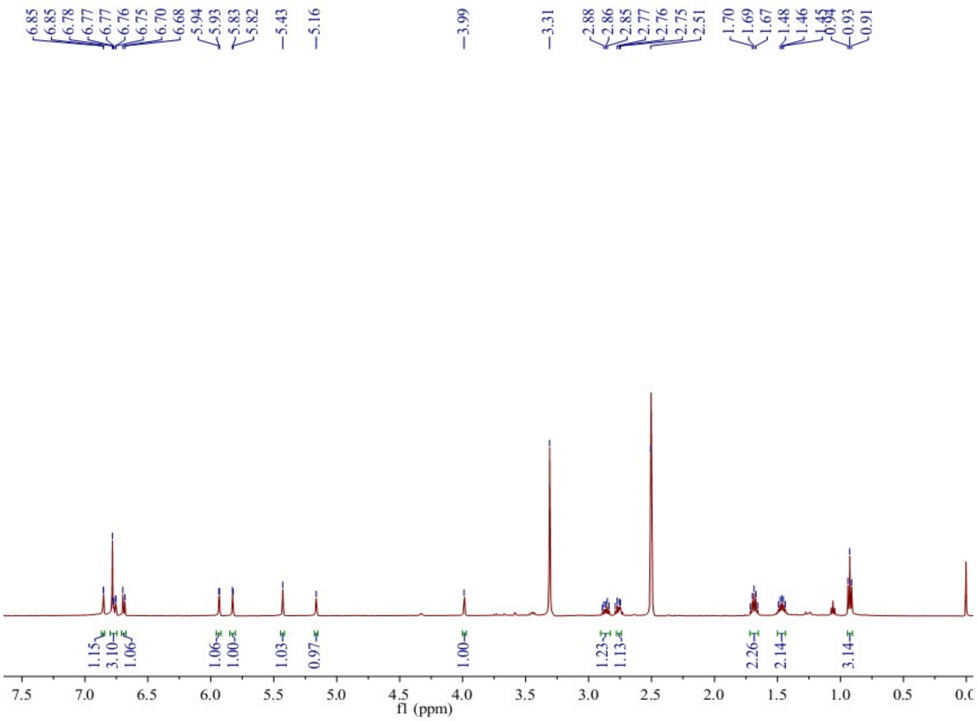

Figure S31. ^{1}H NMR of compound **JP-11** (DMSO-d_6)

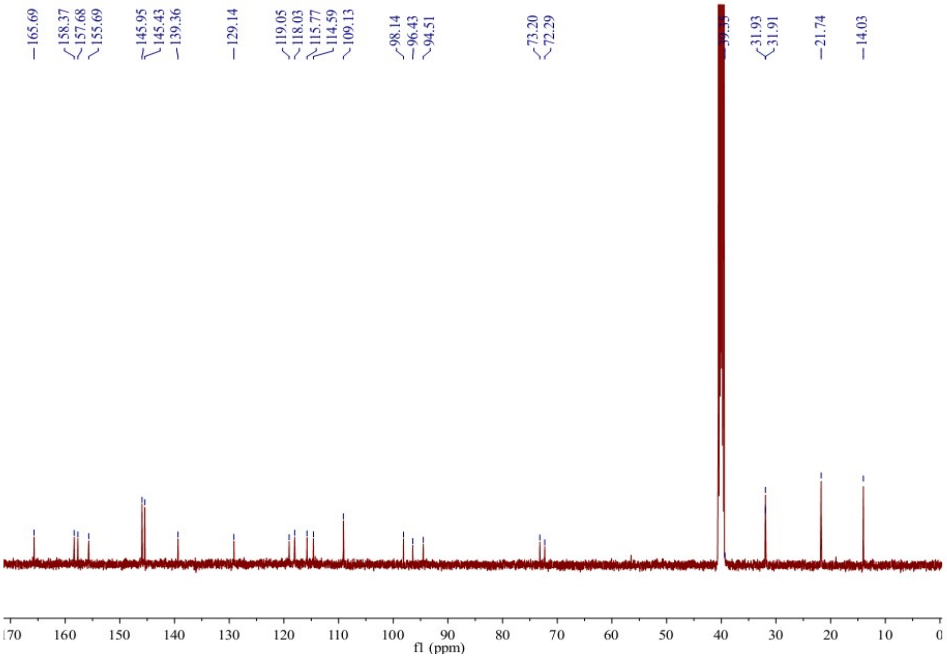

Figure S32. ^{13}C NMR of compound **JP-11** (DMSO-d_6)

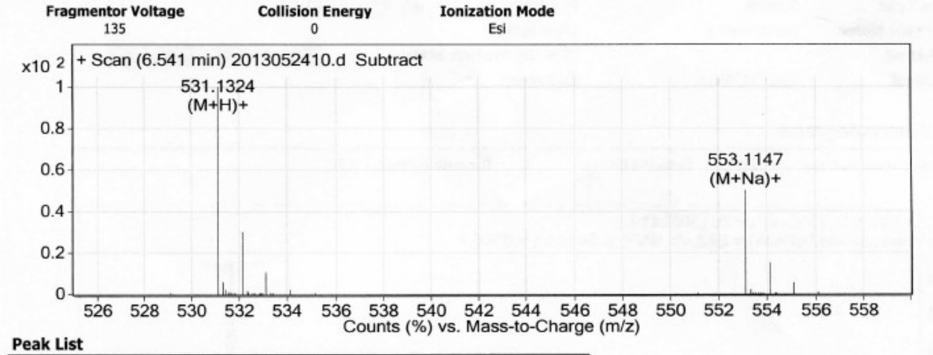

Peak List

m/z	z	Abund	Formula	Ion
361.1106		85683		
441.0821	1	393954		
442.0853	1	94664		
531.1324	1	1132225	C26 H27 O10 S	(M+H)+
532.1358	1	341021	C26 H27 O10 S	(M+H)+
533.1339	1	119694	C26 H27 O10 S	(M+H)+
553.1147	1	572590	C26 H26 Na O10 S	(M+Na)+
554.1176	1	170766	C26 H26 Na O10 S	(M+Na)+
1083.2393	1	180596		
1084.2414	1	106361		

Figure S33. HRESIMS of compound **JP-11**

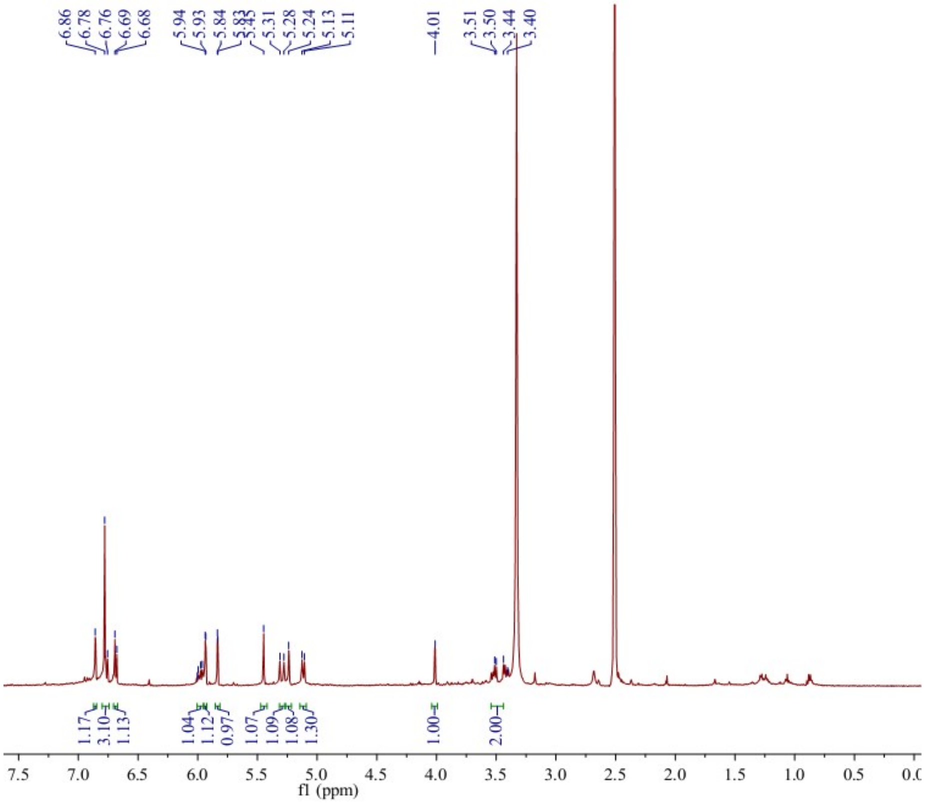

Figure S34. ^{1}H NMR of compound **JP-12** (DMSO-d_6)

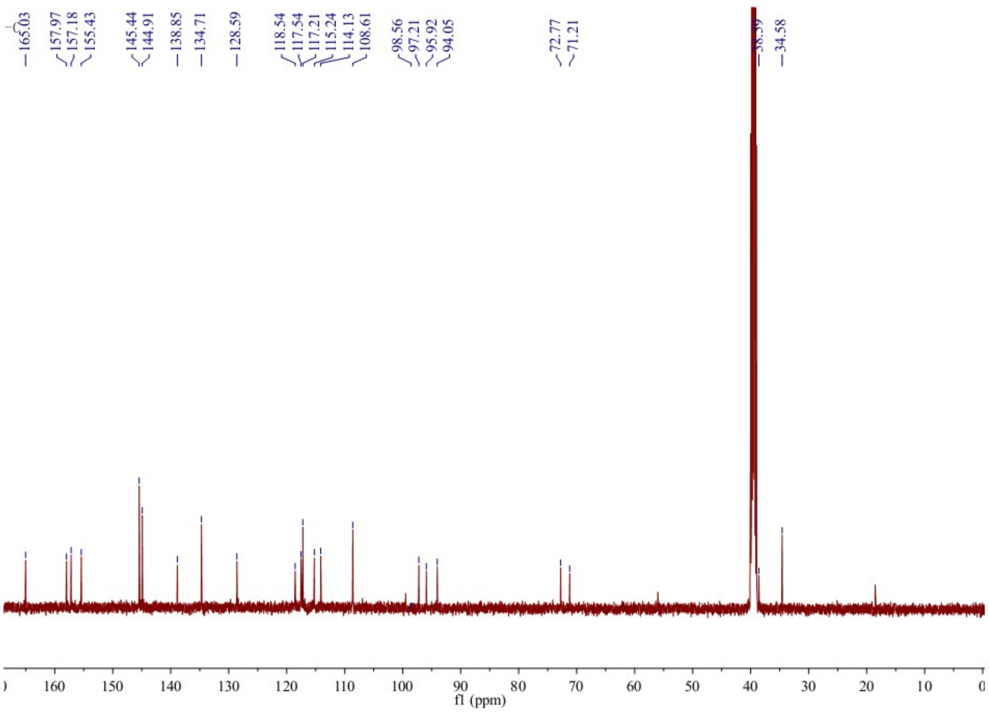

Figure S35. ^{13}C NMR of compound **JP-12** (DMSO-d_6)

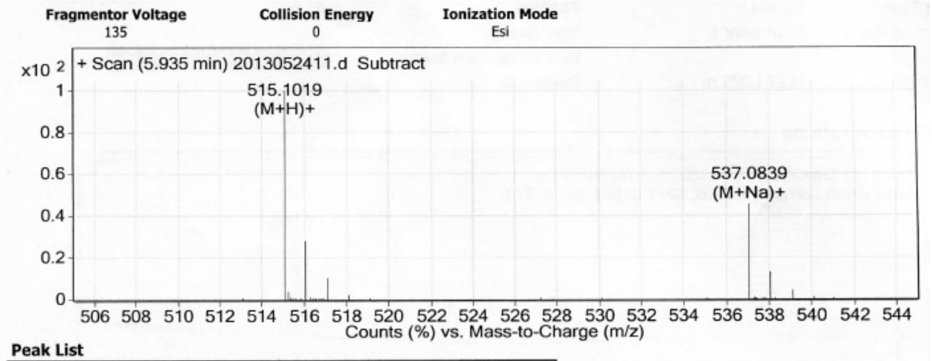

Peak List

m/z	z	Abund	Formula	Ion
130.1593		19260		
284.9611		22626		
286.9579		22987		
345.0791		24544		
441.0823		55139		
515.1019	1	331024	C25 H23 O10 S	(M+H)+
516.1038	1	93819	C25 H23 O10 S	(M+H)+
517.1028	1	33609	C25 H23 O10 S	(M+H)+
537.0839	1	150370	C25 H22 Na O10 S	(M+Na)+
538.086	1	43881	C25 H22 Na O10 S	(M+Na)+

Figure S36. HRESIMS of compound **JP-12**

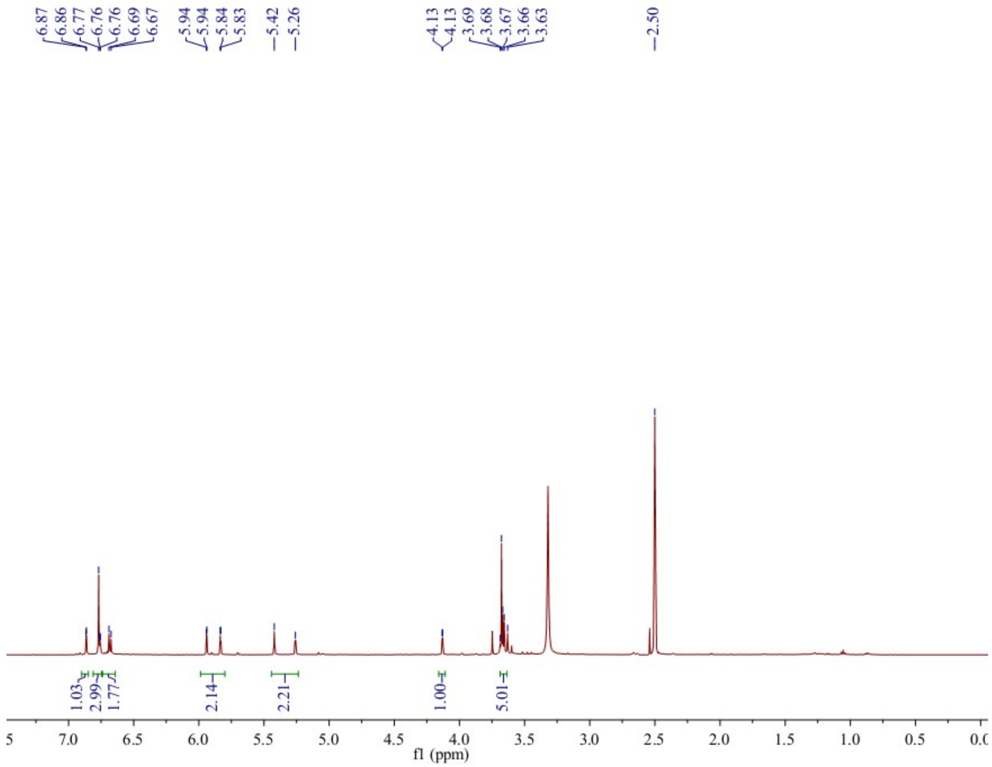

Figure S37. ^{1}H NMR of compound **JP-13** (DMSO-d_6)

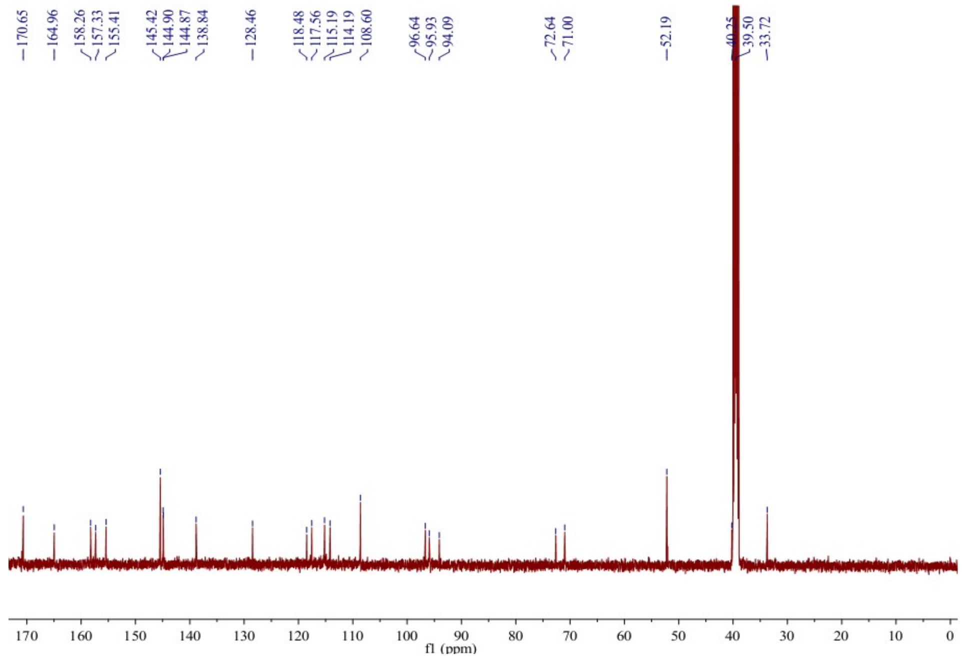

Figure S38. ^{13}C NMR of compound **JP-13** (DMSO-d_6)

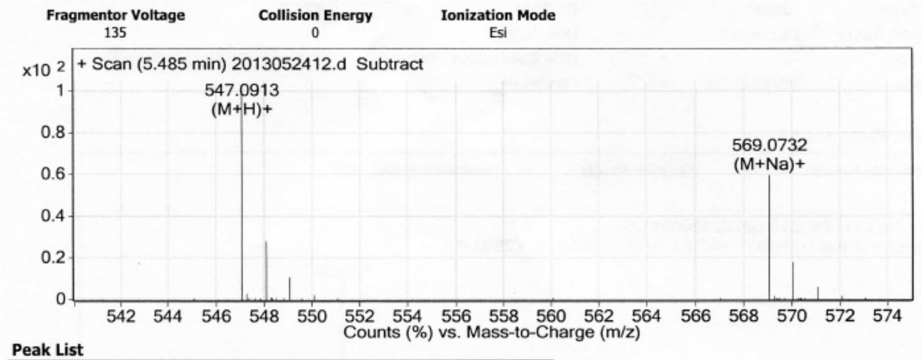

Peak List

m/z	z	Abund	Formula	Ion
116.9864		21253		
130.1592		17467		
151.1109		20183		
223.1301		44494		
441.0825	1	40617		
547.0913	1	167561	C25 H23 O12 S	(M+H)+
548.0934	1	46824	C25 H23 O12 S	(M+H)+
549.0932	1	18590	C25 H23 O12 S	(M+H)+
569.0732	1	100149	C25 H22 Na O12 S	(M+Na)+
570.0754	1	29723	C25 H22 Na O12 S	(M+Na)+

Figure S39. HRESIMS of compound **JP-13**

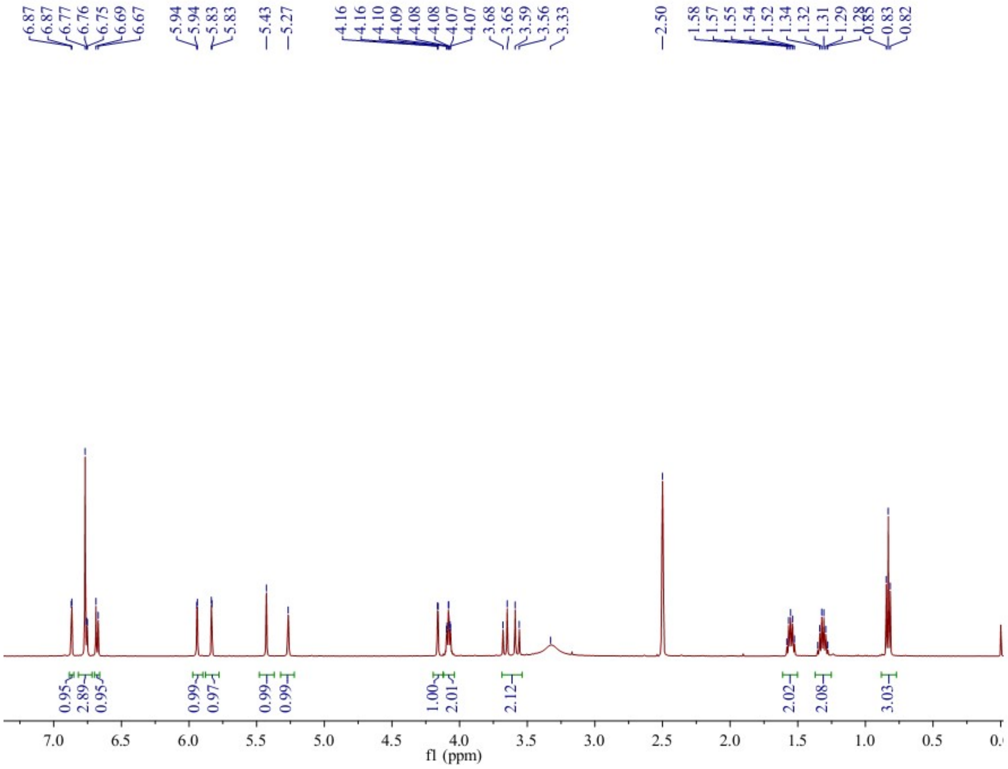

Figure S40. ^{1}H NMR of compound **JP-14** (DMSO-d_6)

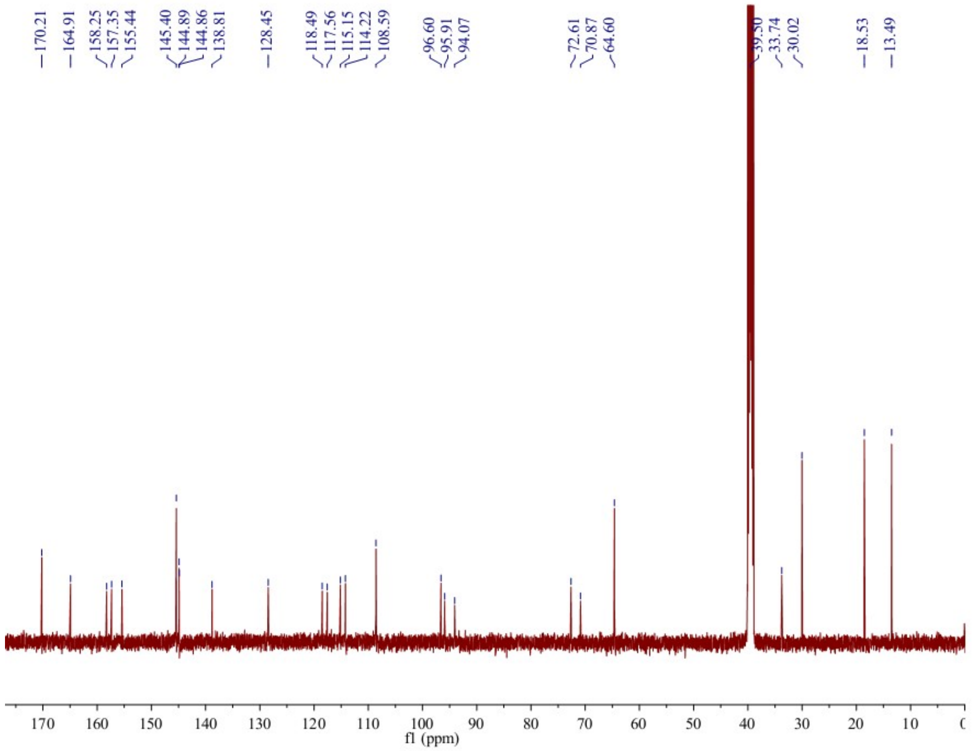

Figure S41. ^{13}C NMR of compound **JP-14** (DMSO-d_6)

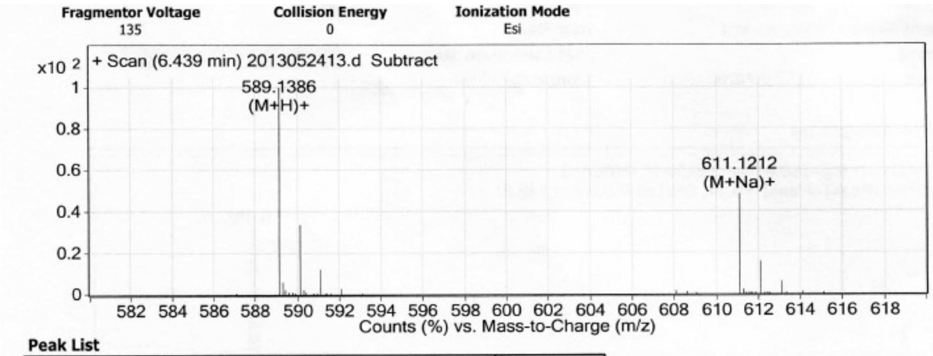

Peak List

m/z	z	Abund	Formula	Ion
441.0827		231788		
589.1386	1	1170971	C28 H29 O12 S	(M+H)+
589.3393		70992		
590.1428	1	390692	C28 H29 O12 S	(M+H)+
591.1411	1	137117	C28 H29 O12 S	(M+H)+
611.1212	1	561665	C28 H28 Na O12 S	(M+Na)+
612.1234	1	182465	C28 H28 Na O12 S	(M+Na)+
613.123	1	68577	C28 H28 Na O12 S	(M+Na)+
1199.2506	1	135614		
1200.2547	1	88474		

Figure S42. HRESIMD of compound **JP-14**

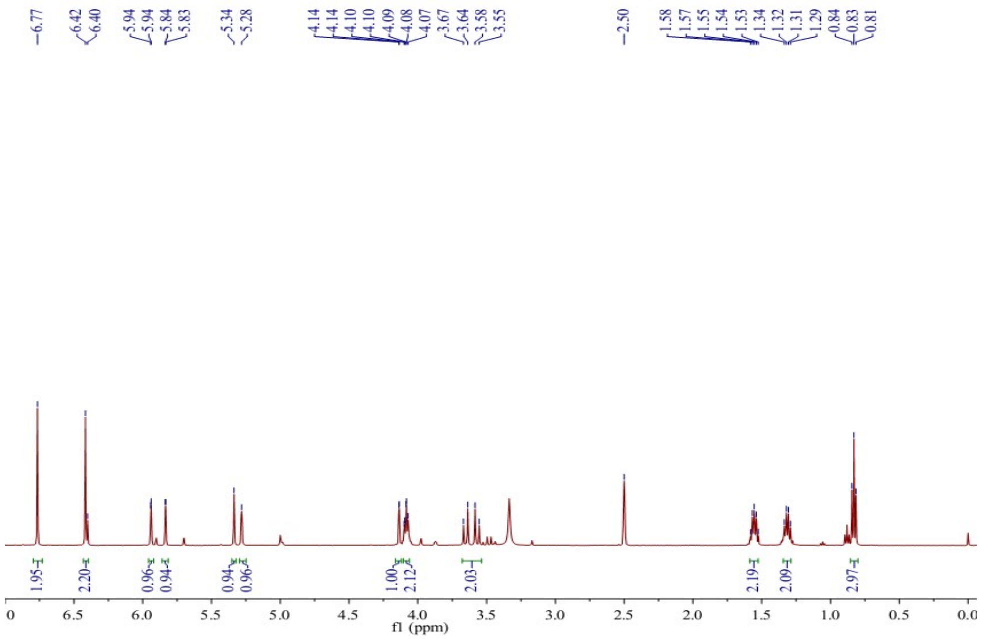

Figure S43. ^{1}H NMR of compound **JP-15** (DMSO-d_6)

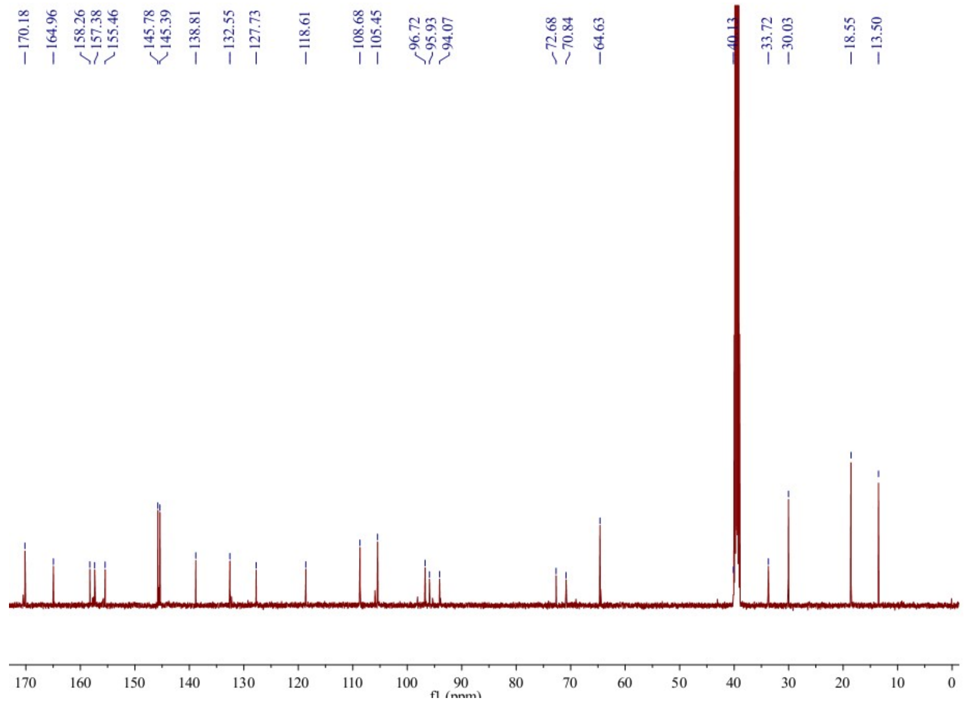

Figure S44. ^{13}C NMR of compound **JP-15** (DMSO-d_6)

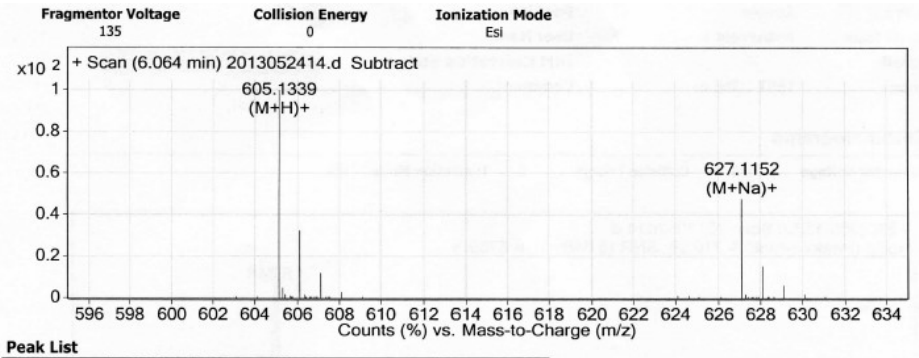

Peak List

m/z	z	Abund	Formula	Ion
287.0551		88907		
453.1217		143557		
457.0772		59997		
475.1037		64086		
605.1339	1	731649	C28 H29 O13 S	(M+H)+
606.1365	1	238446	C28 H29 O13 S	(M+H)+
607.1348	1	86270	C28 H29 O13 S	(M+H)+
627.1152	1	347795	C28 H28 Na O13 S	(M+Na)+
628.1182	1	110886	C28 H28 Na O13 S	(M+Na)+
629.1157	1	42651	C28 H28 Na O13 S	(M+Na)+

Figure S45. HRESIMS of compound **JP-15**

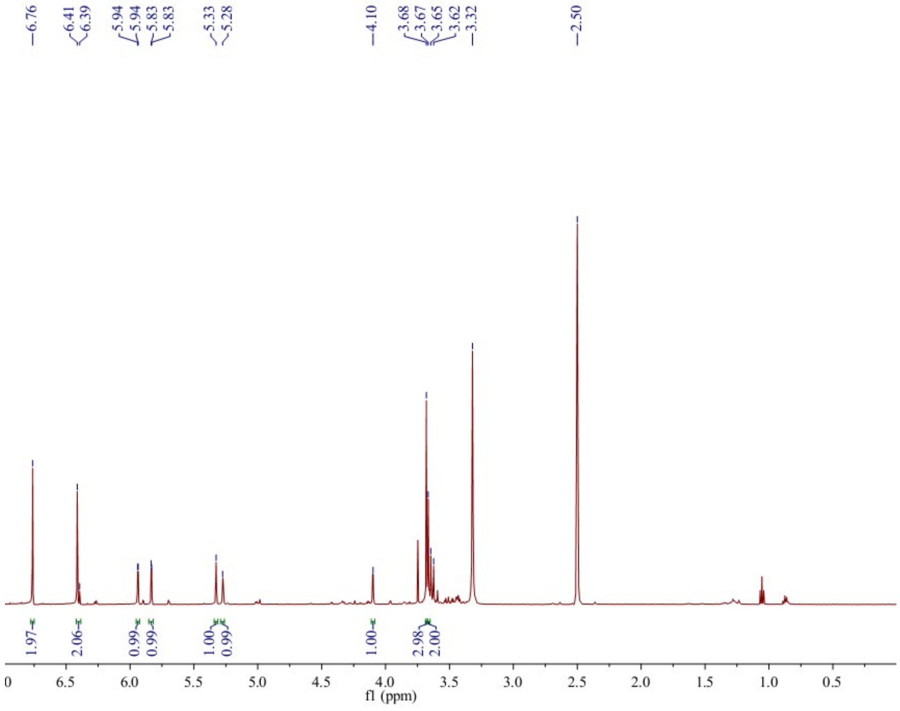

Figure S46. ^{1}H NMR of compound **JP-16** (DMSO-d_6)

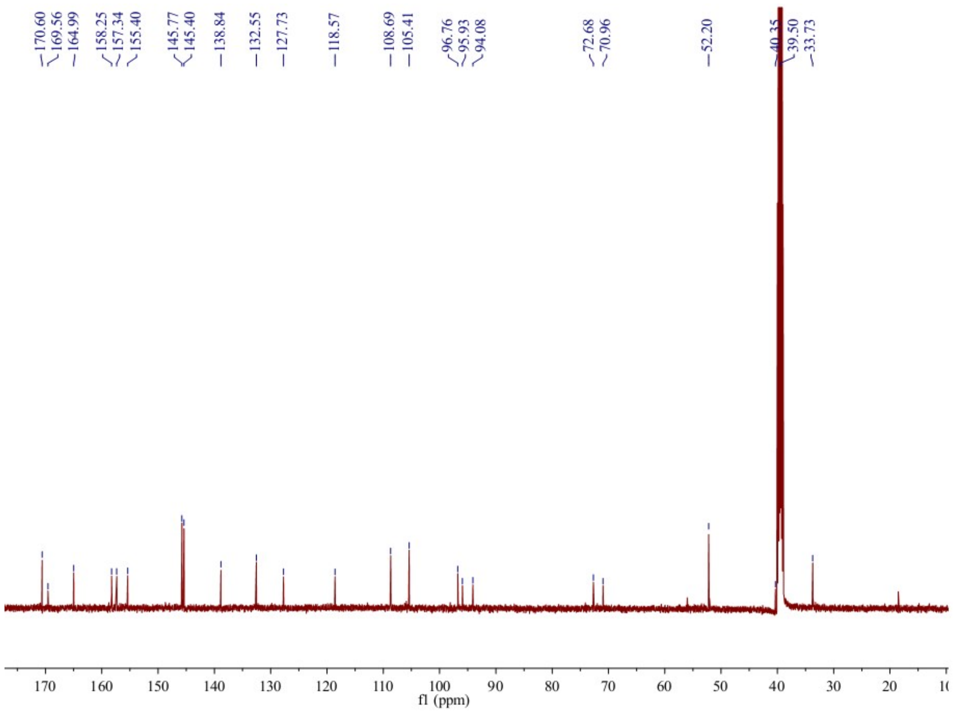

Figure S47. ^{13}C NMR of compound **JP-16** (DMSO-d_6)

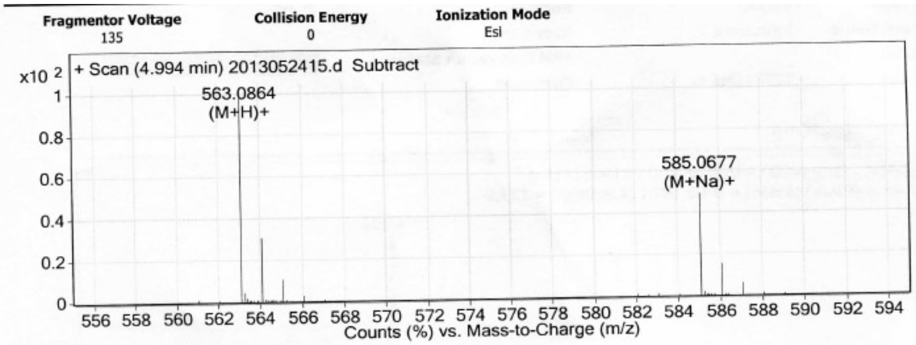

Peak List

m/z	z	Abund	Formula	Ion
211.0093		315469		
228.0359		85270		
232.9914		310418		
287.0551		79353		
457.0766		56348		
563.0864	1	534776	C25 H23 O13 S	(M+H)+
564.0894	1	166314	C25 H23 O13 S	(M+H)+
565.0884	1	59239	C25 H23 O13 S	(M+H)+
585.0677	1	263600	C25 H22 Na O13 S	(M+Na)+
586.0712	1	80385	C25 H22 Na O13 S	(M+Na)+

Figure S48. HRESIMS of compound **JP-16** (DMSO-d_6)

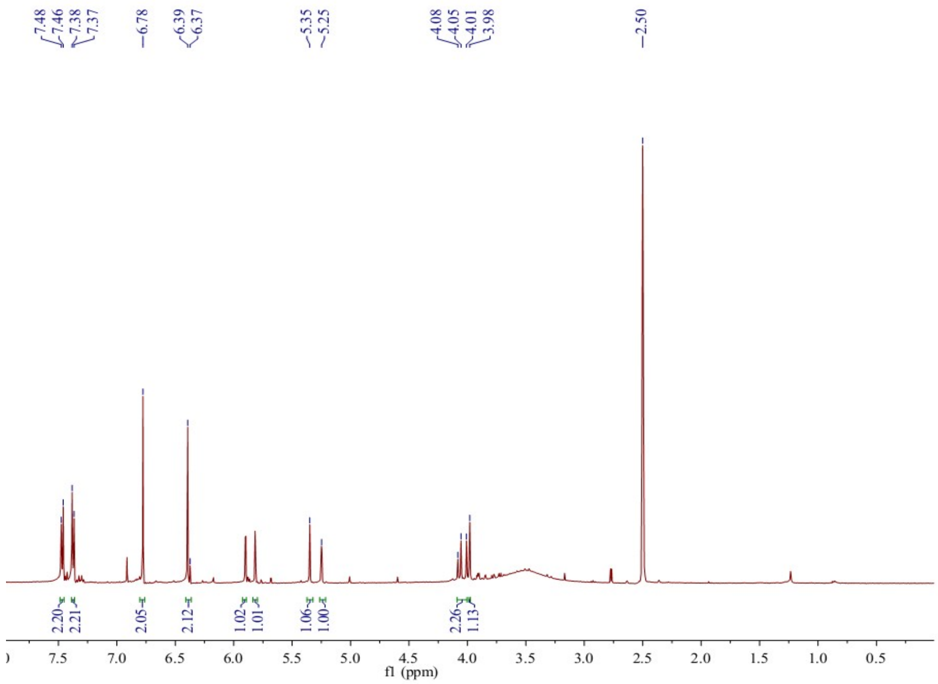

Figure S49. ^{1}H NMR of compound **JP-17** (DMSO-d_6)

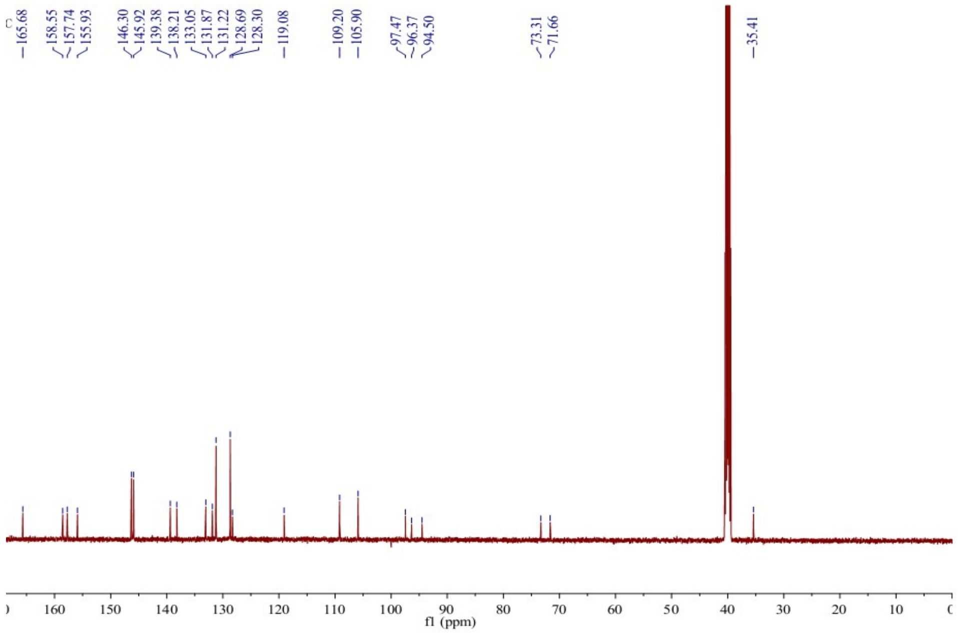

Figure S50. ^{13}C NMR of compound **JP-17** (DMSO-d_6)

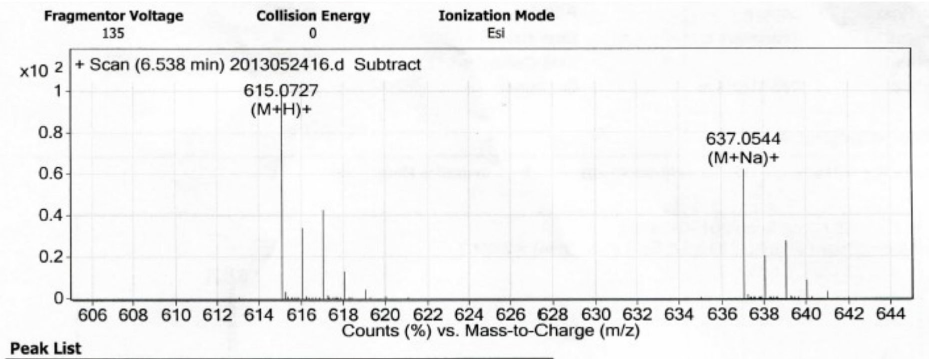

Peak List

m/z	z	Abund	Formula	Ion
287.0553		73379		
445.0506		44942		
457.0762		40676		
615.0727	1	359498	C29 H24 Cl O11 S	(M+H)+
616.0758	1	121664	C29 H24 Cl O11 S	(M+H)+
617.0711	1	154272	C29 H24 Cl O11 S	(M+H)+
618.0726	1	46331	C29 H24 Cl O11 S	(M+H)+
637.0544	1	224692	C29 H23 Cl Na O11 S	(M+Na)+
638.0573	1	74011	C29 H23 Cl Na O11 S	(M+Na)+
639.0523	1	100057	C29 H23 Cl Na O11 S	(M+Na)+

Figure S51. HRESIMS of compound **JP-17**

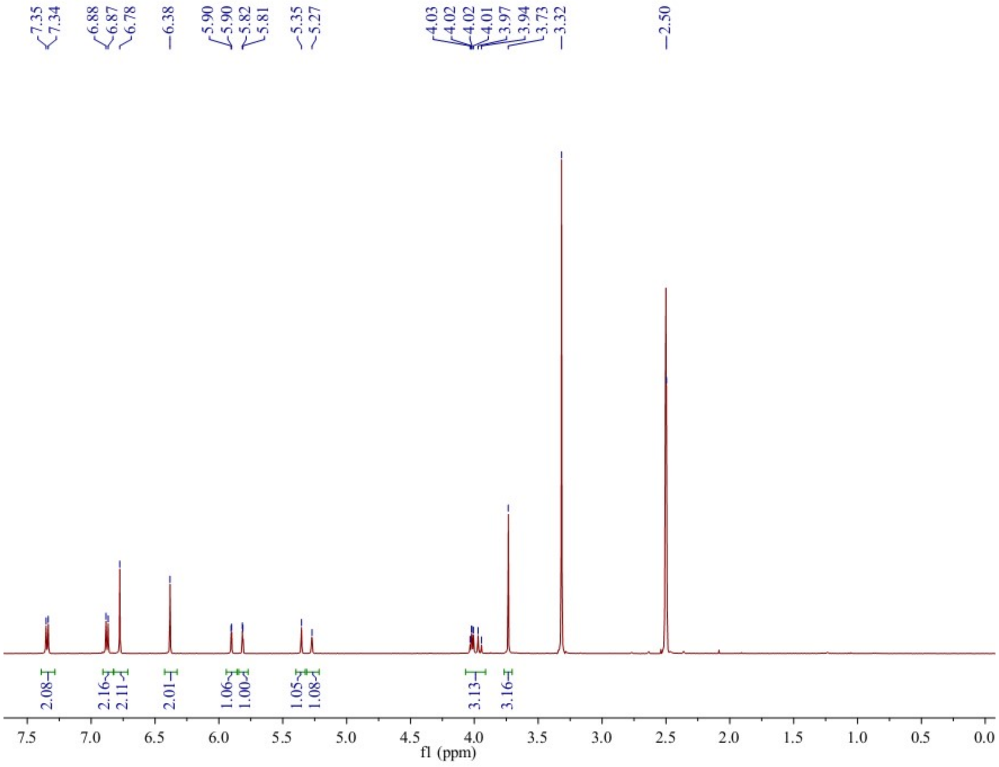

Figure S52. ^{1}H NMR of compound **JP-18** (DMSO-d_6)

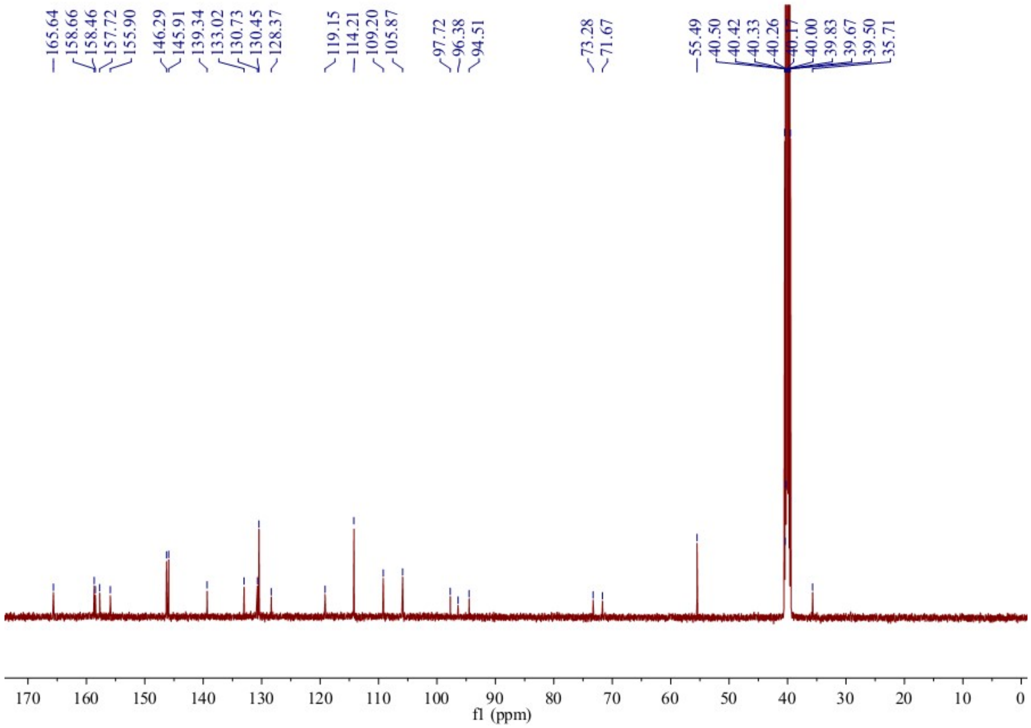

Figure S53. ^{13}C NMR of compound **JP-18** (DMSO-d_6)

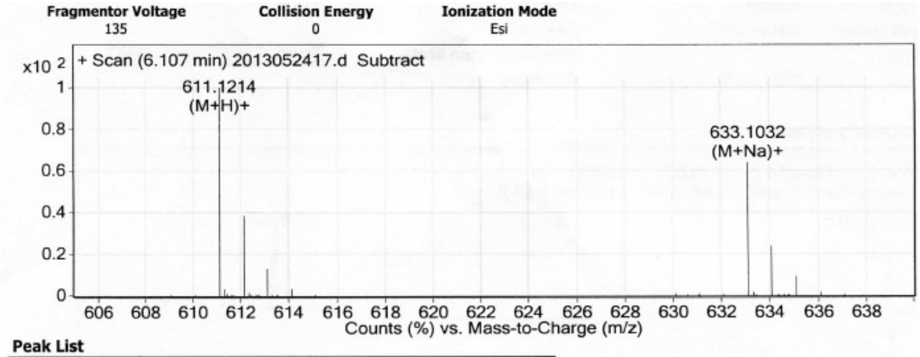

Peak List

m/z	z	Abund	Formula	Ion
116.9849		18455		
287.0526		46661		
441.0986		29142		
457.0747		26984		
611.1214	1	259112	C30 H27 O12 S	(M+H)+
612.1238	1	99017	C30 H27 O12 S	(M+H)+
613.1239	1	33273	C30 H27 O12 S	(M+H)+
633.1032	1	165728	C30 H26 Na O12 S	(M+Na)+
634.1071	1	61435	C30 H26 Na O12 S	(M+Na)+
635.105	1	24198	C30 H26 Na O12 S	(M+Na)+

Figure S54. HRESIMS of compound **JP-18**

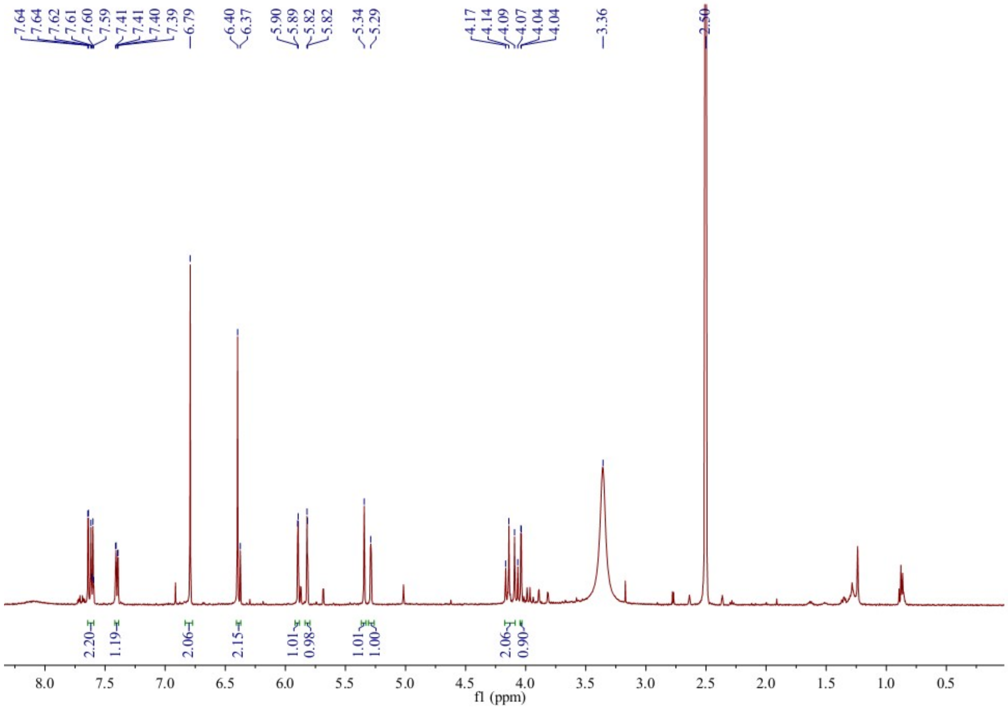

Figure S55. ^{1}H NMR of compound **JP-19** (DMSO-d_6)

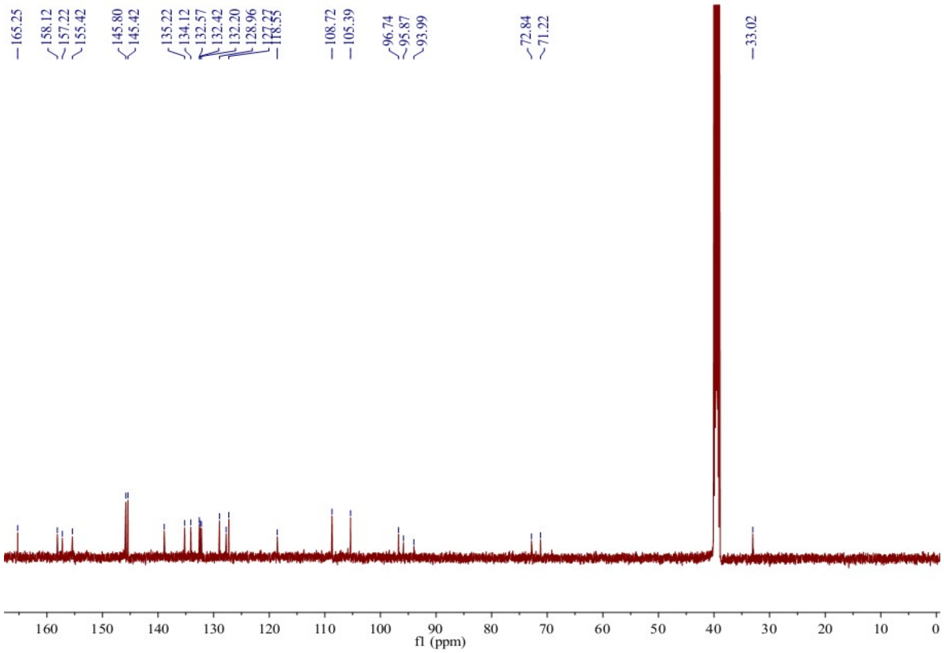

Figure S56. ^{13}C NMR of compound **JP-19** (DMSO-d_6)

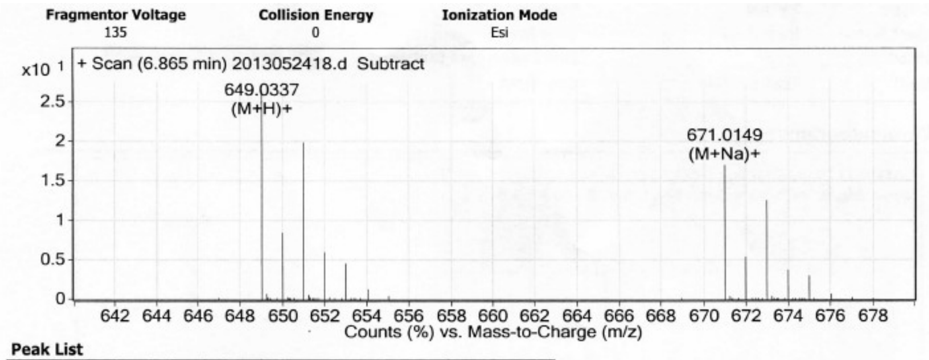

Peak List

m/z	z	Abund	Formula	Ion
158.154	1	819975		
159.1567	1	86760		
180.1356		313052		
287.0544		64395		
649.0337	1	215968	C29 H23 Cl2 O11 S	(M+H)+
650.037	1	69106	C29 H23 Cl2 O11 S	(M+H)+
651.0318	1	162809	C29 H23 Cl2 O11 S	(M+H)+
652.033	1	49400	C29 H23 Cl2 O11 S	(M+H)+
671.0149	1	138638	C29 H22 Cl2 Na O11 S	(M+Na)+
673.0126	1	103402	C29 H22 Cl2 Na O11 S	(M+Na)+

Figure S57. HRESIMS of compound **JP-19**

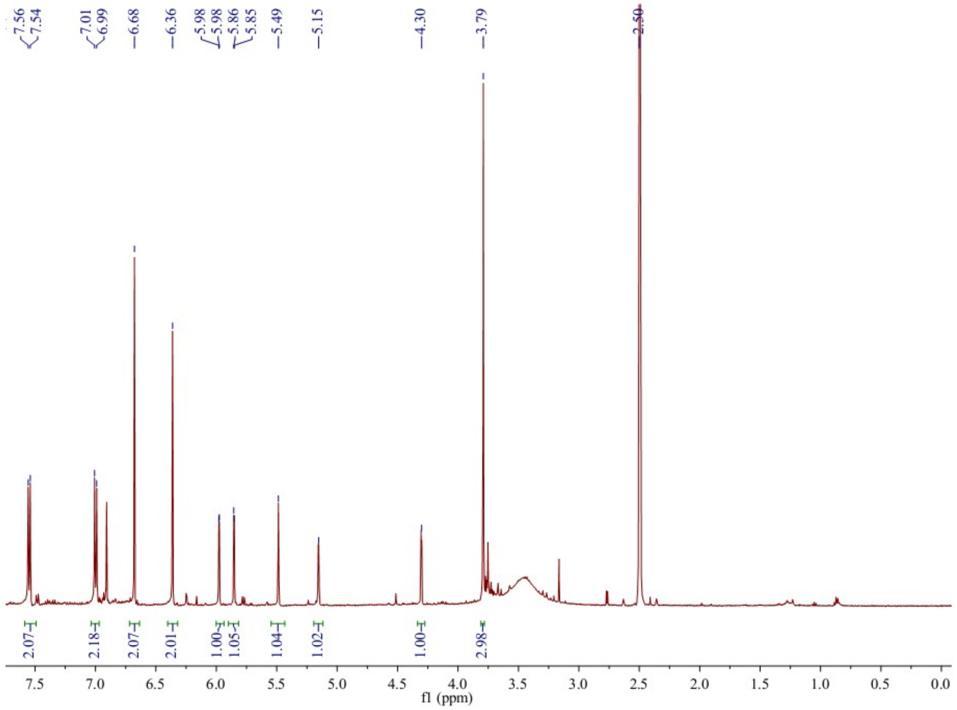

Figure S58. ^{1}H NMR of compound **JP-20** (DMSO-d_6)

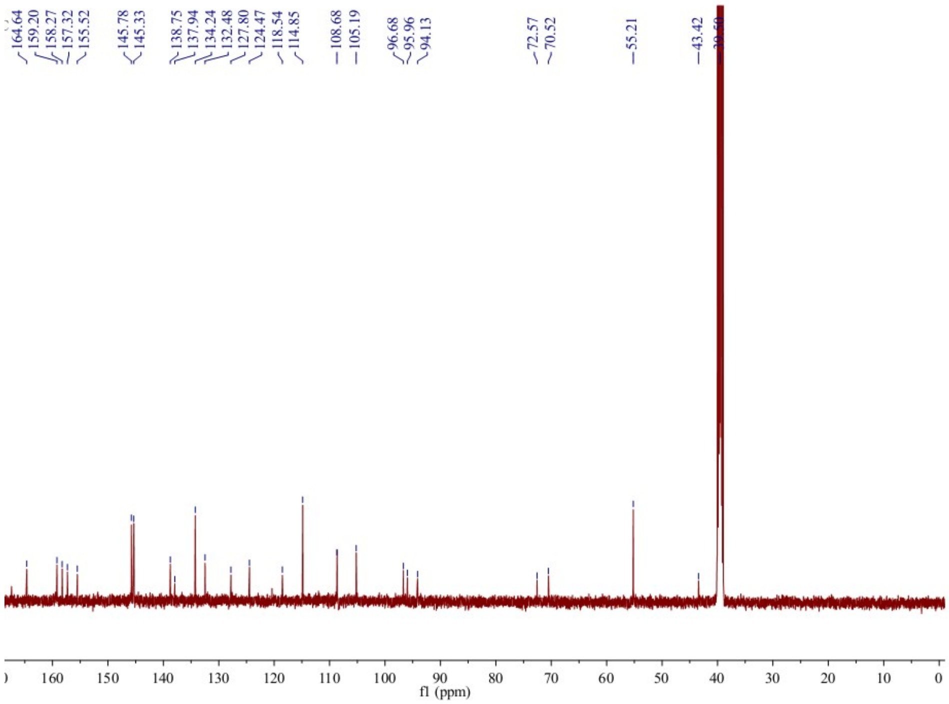

Figure S59. ^{13}C NMR of compound **JP-20** (DMSO-d_6)

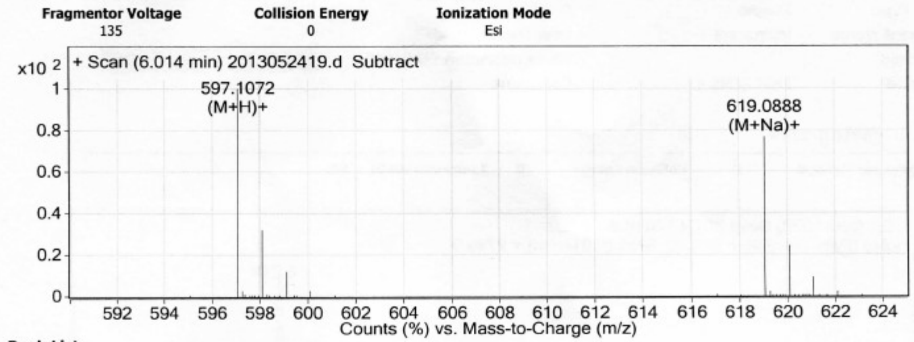

Peak List

m/z	z	Abund	Formula	Ion
287.0551	1	76282		
299.111		32261		
427.0854		30283		
457.0775	1	48092		
597.1072	1	202428	C29 H25 O12 S	(M+H)+
598.111	1	64821	C29 H25 O12 S	(M+H)+
599.1087	1	23625	C29 H25 O12 S	(M+H)+
619.0888	1	155185	C29 H24 Na O12 S	(M+Na)+
620.0913	1	49334	C29 H24 Na O12 S	(M+Na)+
621.0899	1	19159	C29 H24 Na O12 S	(M+Na)+

Figure S60. HRESIMS of compound **JP-20**

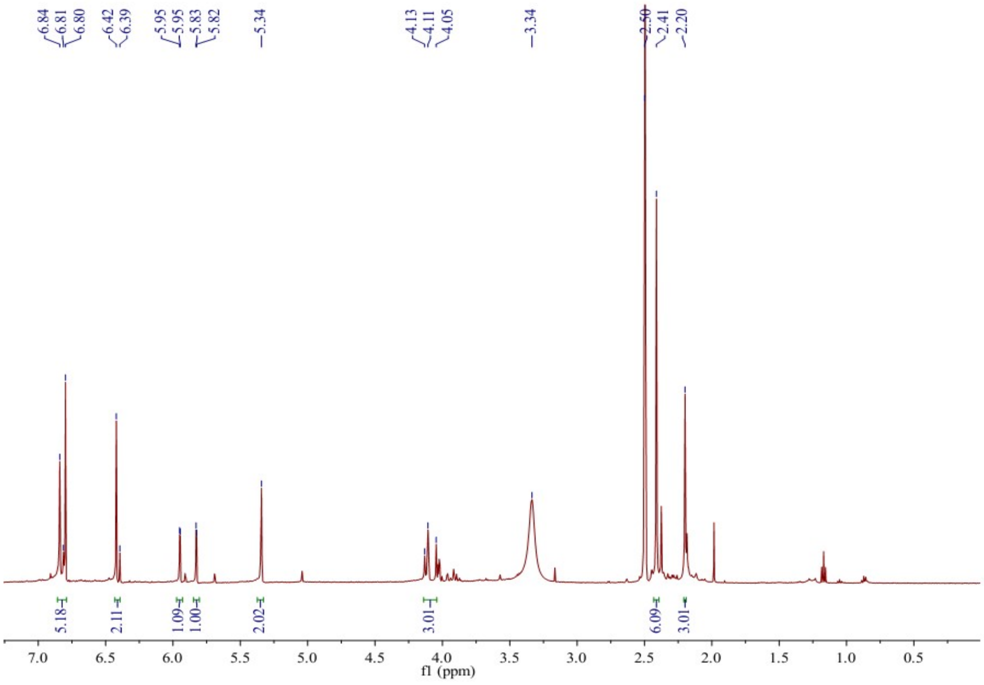

Figure S61. ^{1}H NMR of compound **JP-21** (DMSO-d_6)

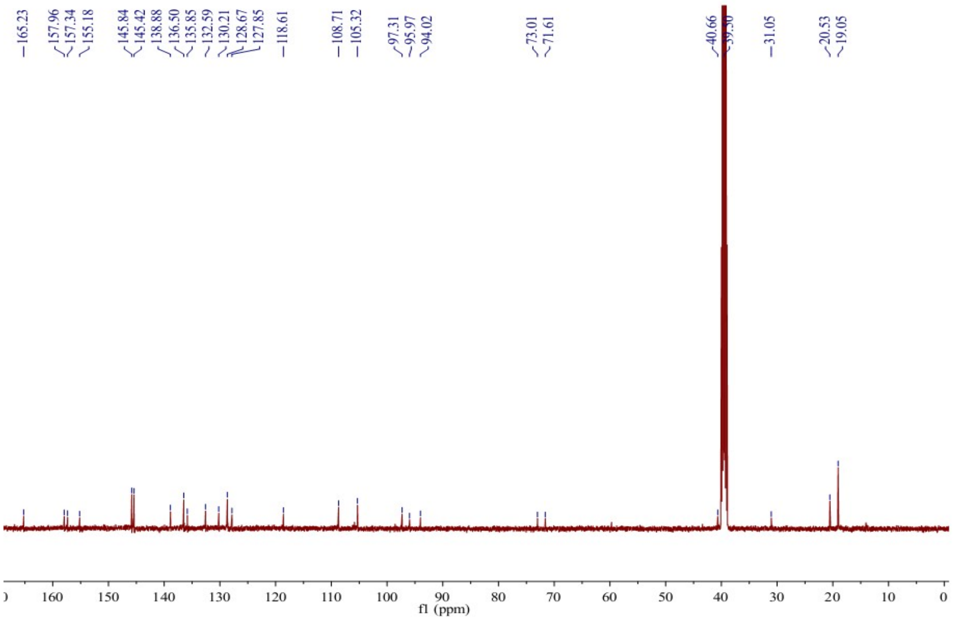

Figure S62. ^{13}C NMR of compound **JP-21** (DMSO-d_6)

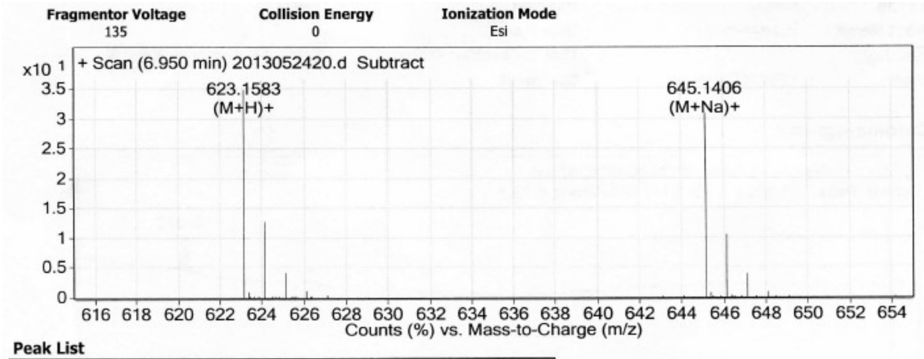

Peak List

m/z	z	Abund	Formula	Ion
158.1543	1	322437		
159.1571	1	32930		
180.1361		128776		
195.1738		33809		
235.1668		53039		
287.056		27700		
623.1583	1	112253	C32 H31 O11 S	(M+H)+
624.1617	1	41283	C32 H31 O11 S	(M+H)+
645.1406	1	99904	C32 H30 Na O11 S	(M+Na)+
646.1431	1	34290	C32 H30 Na O11 S	(M+Na)+

Figure S63. HRESIMS of compound **JP-21**

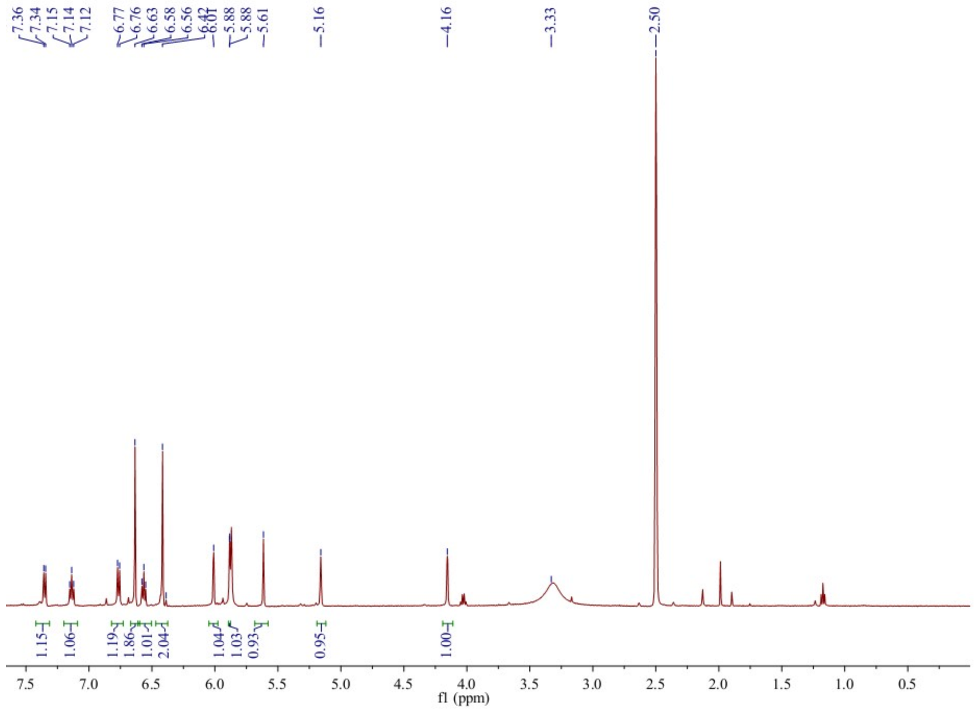

Figure S64. ^{1}H NMR of compound **JP-22** (DMSO-d_6)

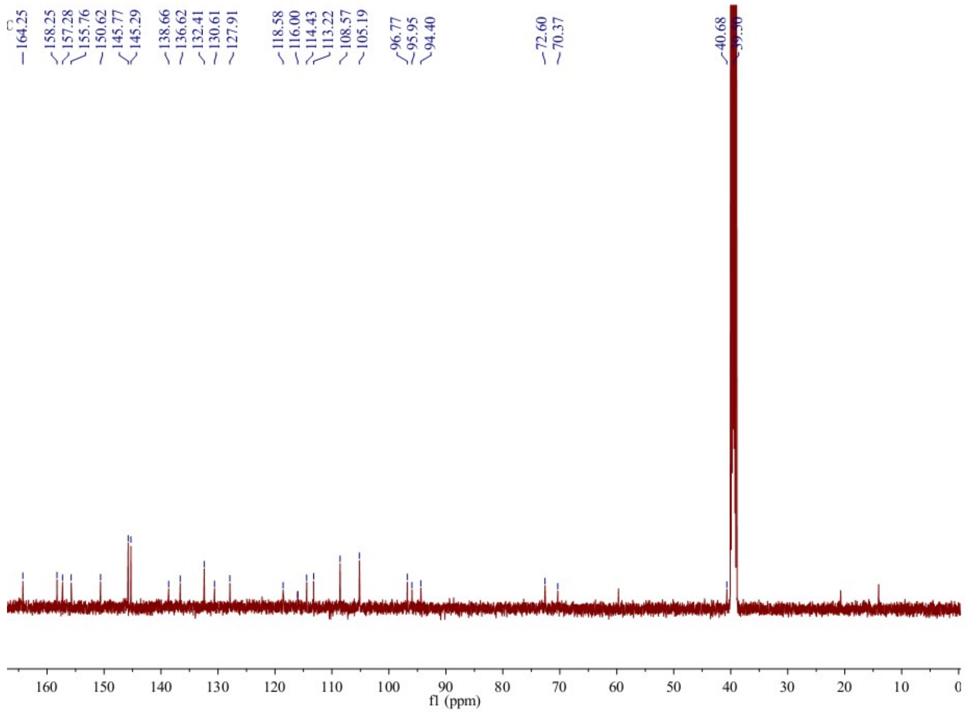

Figure S65. ^{1}H NMR of compound **JP-22** (DMSO-d_6)

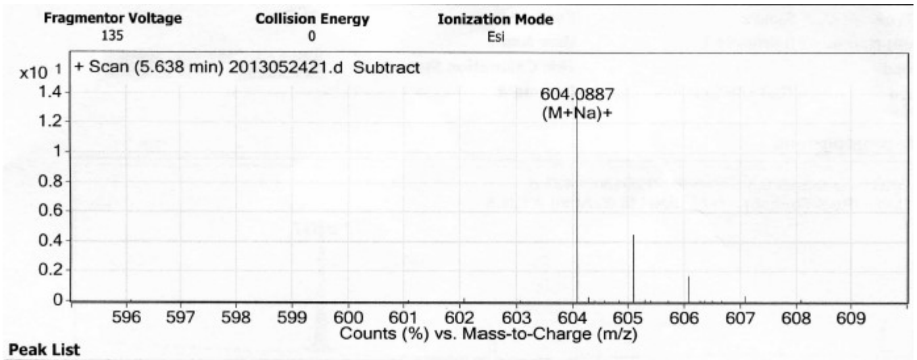

Peak List

m/z	z	Abund	Formula	Ion
163.0389		47425		
195.0693		31664		
217.0476		59996		
223.1299		40884		
287.0556	1	517214		
288.0583	1	83445		
412.0849		29663		
457.0775	1	407786		
458.0804	1	99411		
604.0887		71789	C28 H23 N Na O11 S	(M+Na)+

Figure S66. HRESIMS of compound **JP-22**

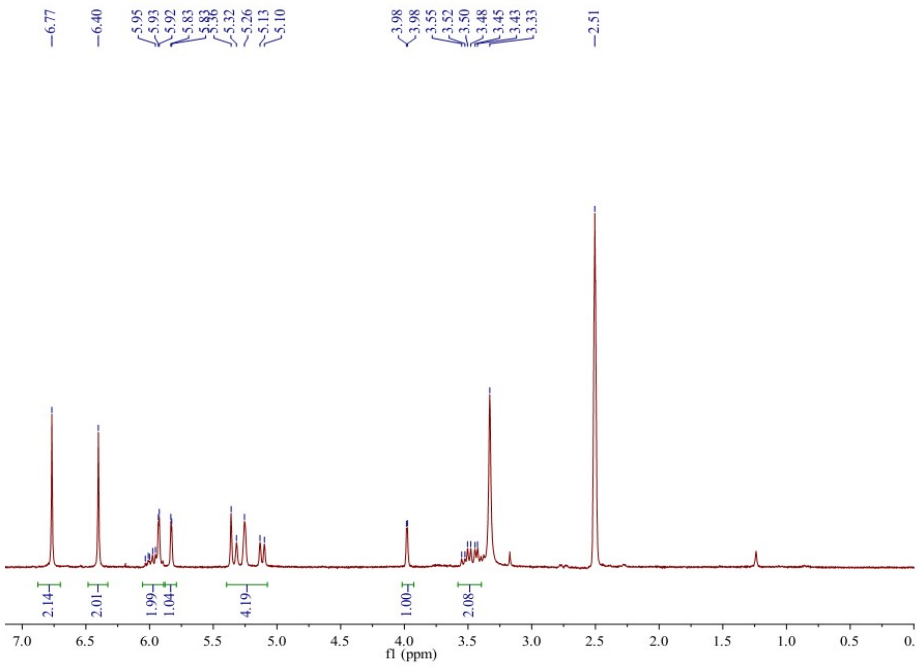

Figure S67. ^{1}H NMR of compound **JP-23** (DMSO-d_6)

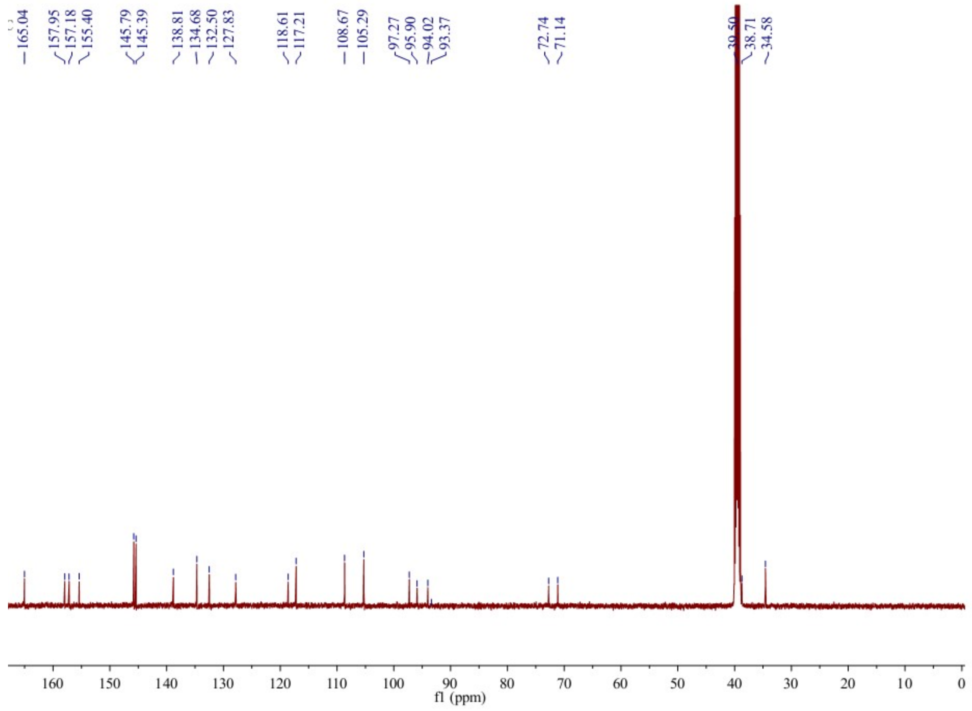

Figure S68. ^{13}C NMR of compound **JP-23** (DMSO-d_6)

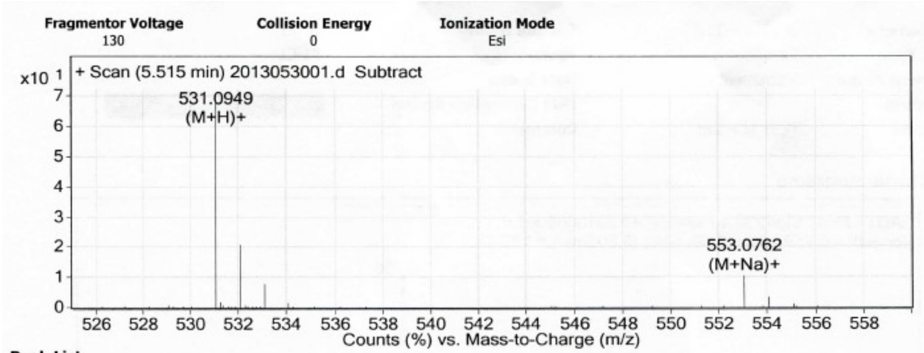

Peak List

m/z	z	Abund	Formula	Ion
123.1164		33331		
151.1108	1	43013		
169.1216	1	49637		
186.1483	1	27486		
211.097	1	63685		
223.1302		37980		
233.0783	1	58527		
246.1695	1	100841		
251.1355		34685		
531.0949	1	69497	C25 H23 O11 S	(M+H)+

Figure S69. HRESIMS of compound **JP-23**

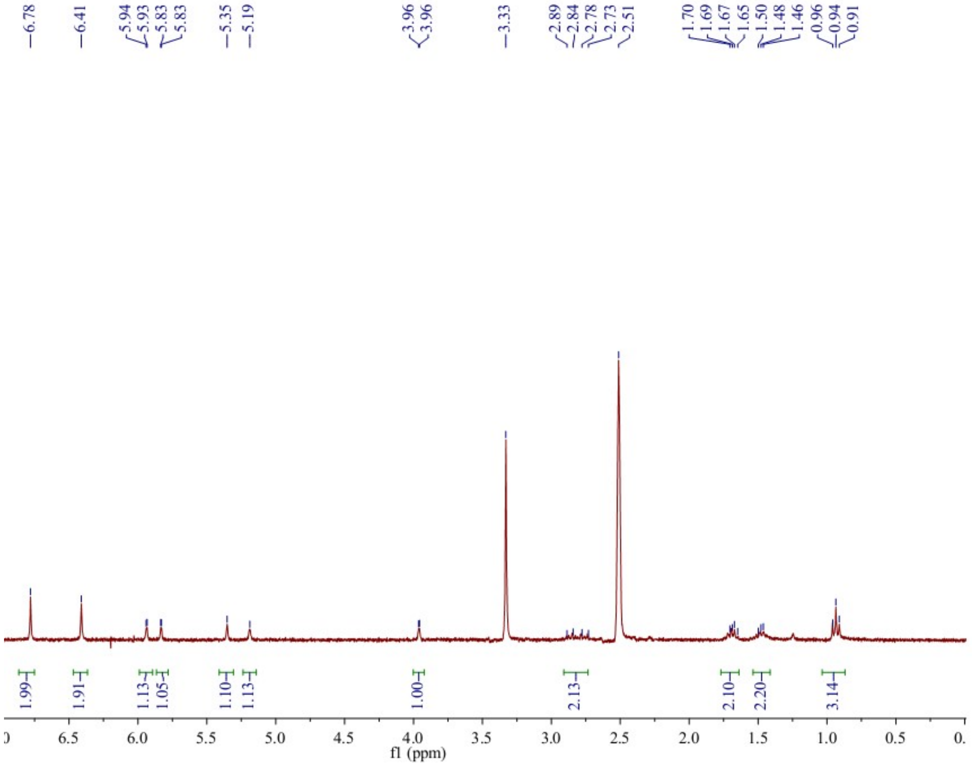

Figure S70. ^{1}H NMR of compound **JP-24** (DMSO-d_6)

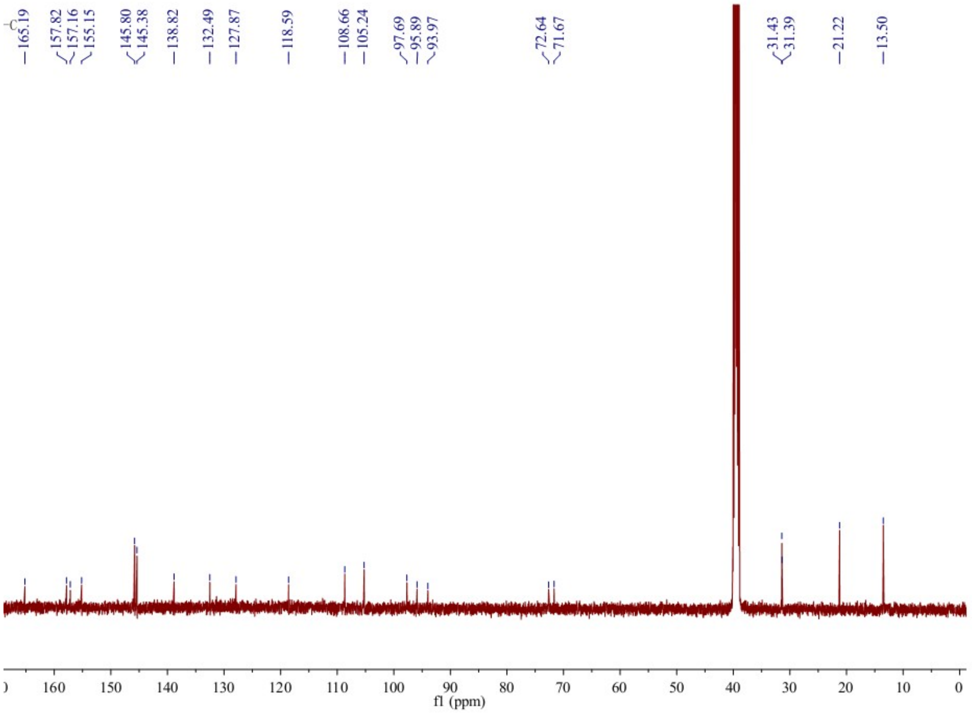

Figure S71. ^{13}C NMR of compound **JP-24** (DMSO-d_6)

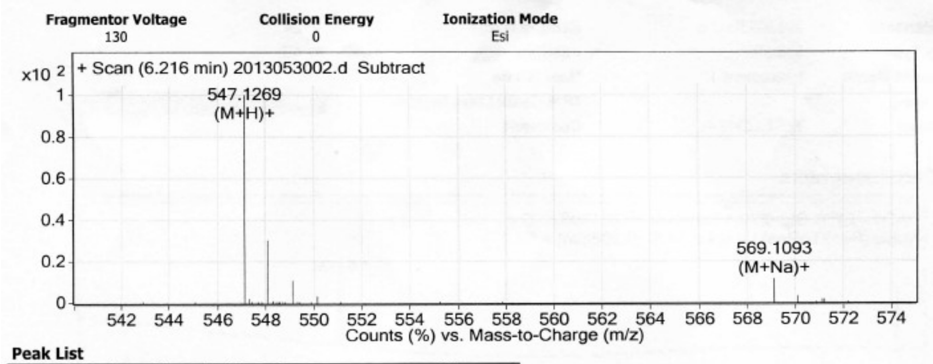

Peak List

m/z	z	Abund	Formula	Ion
246.1694		9721		
254.1727		9911		
277.1302		16176		
284.9615		9020		
286.9578		8474		
287.0534		7537		
294.1578		9155		
547.1269	1	59576	C26 H27 O11 S	(M+H)+
548.1312	1	18057	C26 H27 O11 S	(M+H)+
922.0098		10045		

Figure S72. HRESIMS of compound **JP-24**

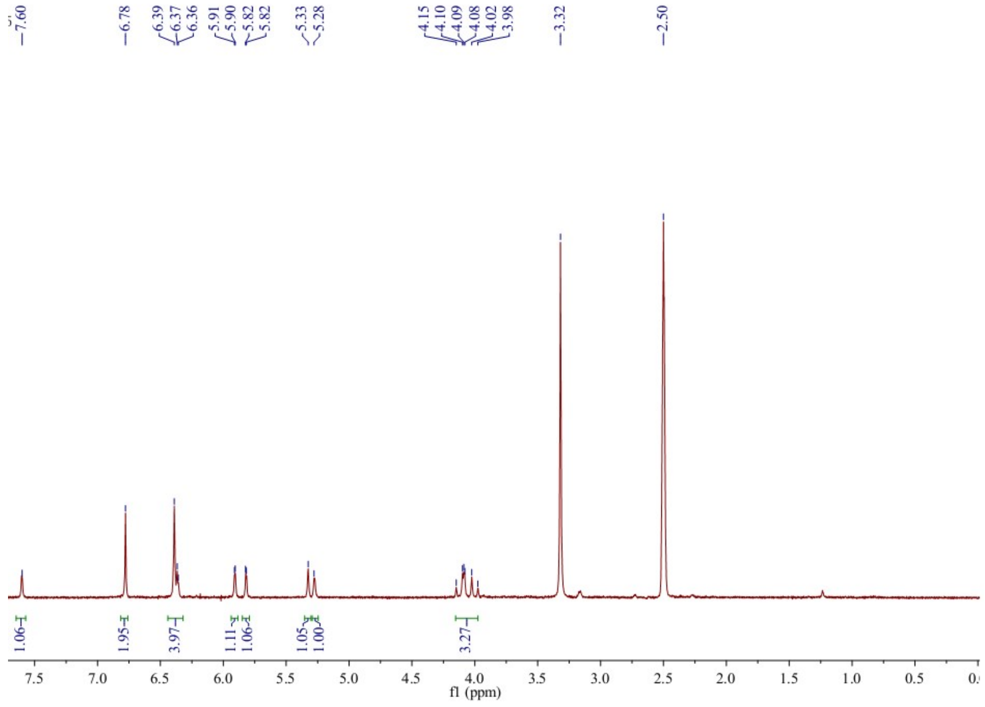

Figure S73. ^{1}H NMR of compound **JP-25** (DMSO-d_6)

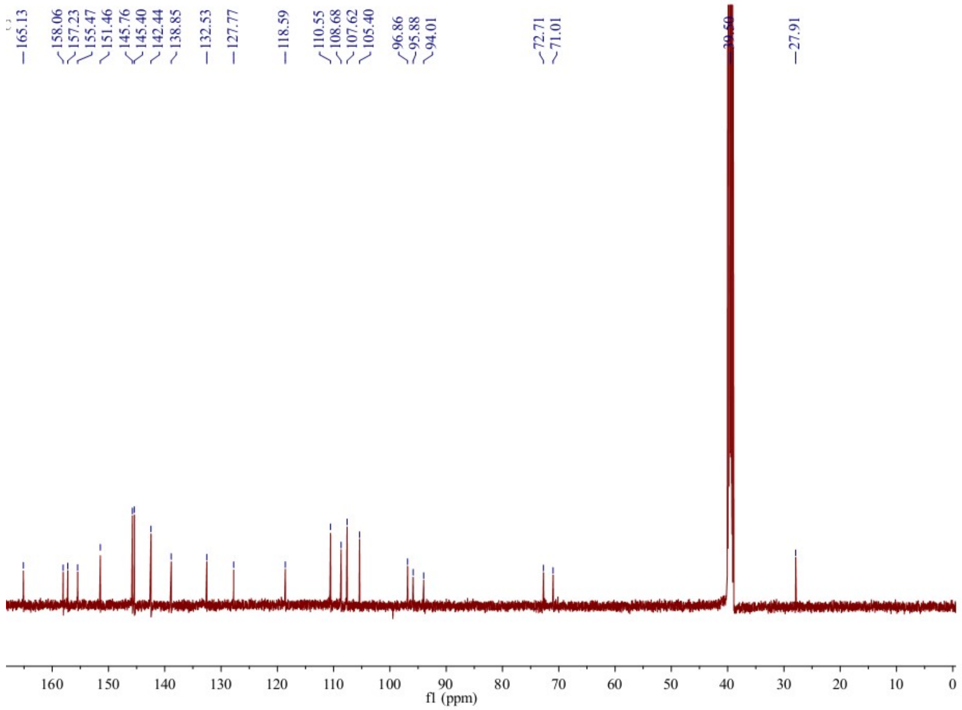

Figure S74. ^{13}C NMR of compound **JP-25** (DMSO-d_6)

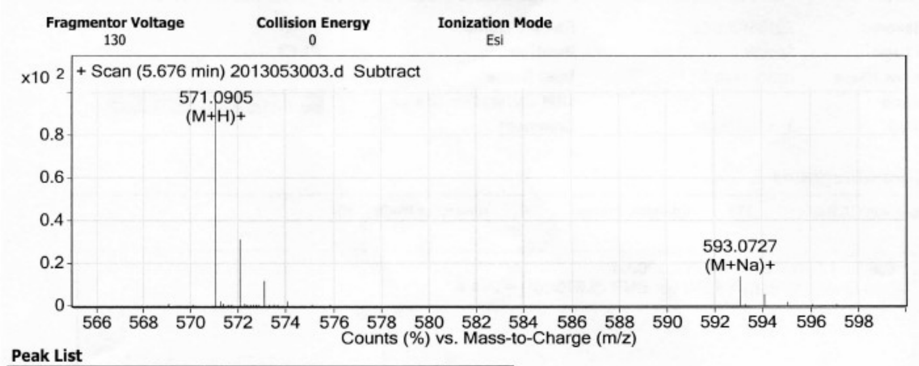

Peak List

m/z	z	Abund	Formula	Ion
123.1168		21110		
169.1222		32810		
211.0967	1	42863		
223.1298		23802		
233.0785	1	45290		
246.1696	1	84768		
251.1261	1	50968		
260.1848	1	25530		
571.0905	1	82542	C27 H23 O12 S	(M+H)+
572.0949	1	26499	C27 H23 O12 S	(M+H)+

Figure S75. HRESIMS of compound **JP-25**

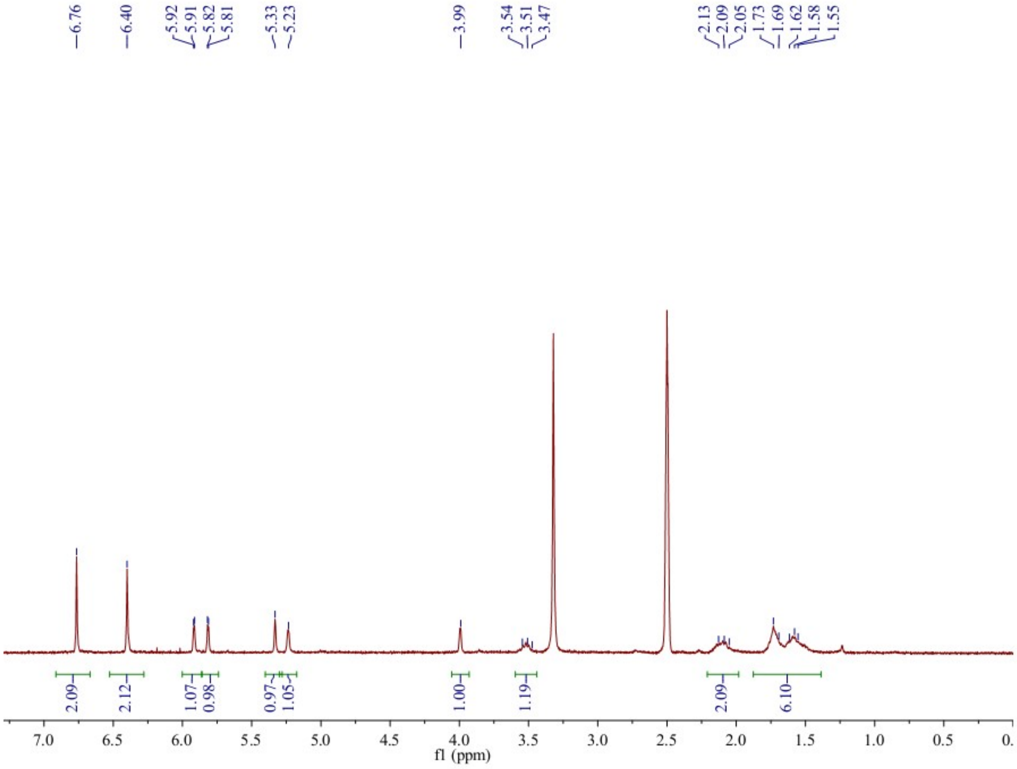

Figure S76. ^{1}H NMR of compound **JP-26** (DMSO-d_6)

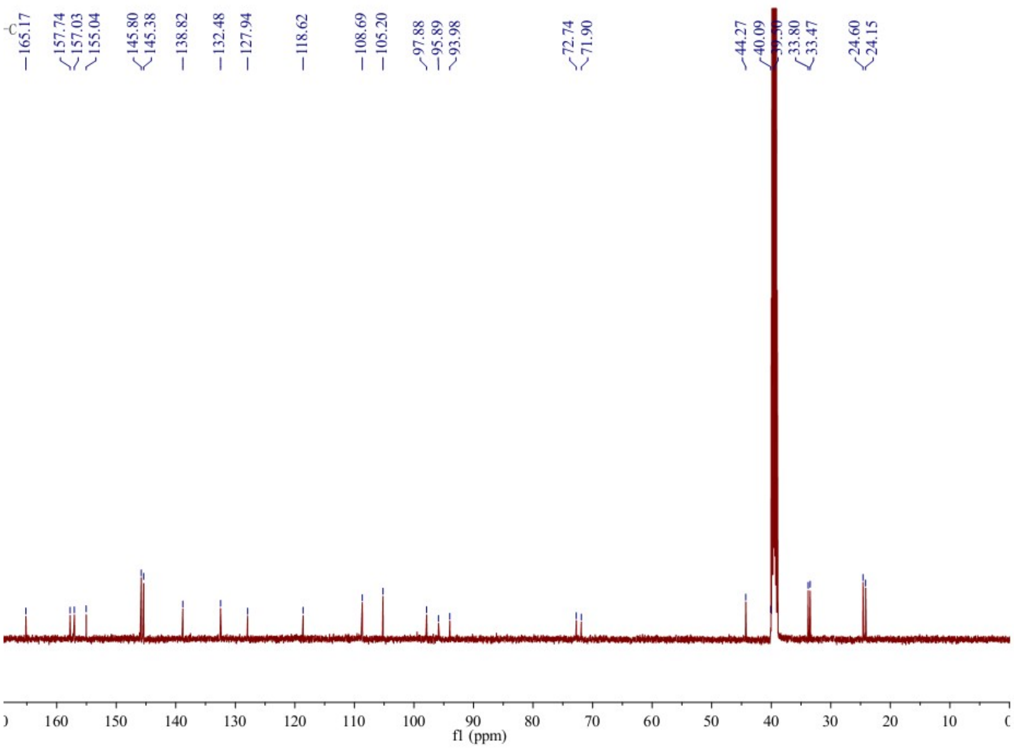

Figure S77. ^{1}H NMR of compound **JP-26** (DMSO-d_6)

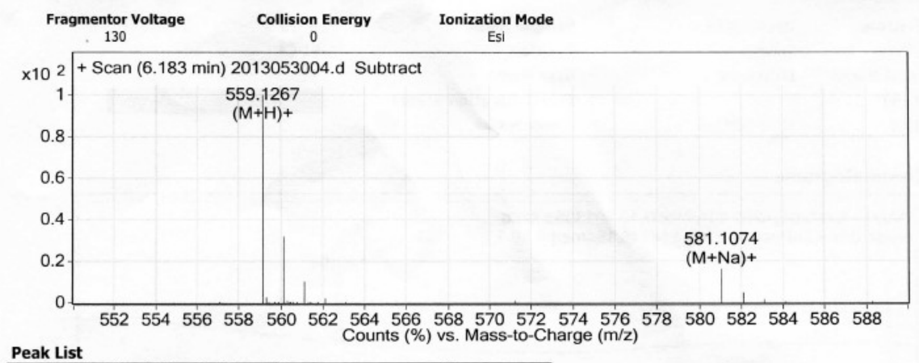

Peak List

m/z	z	Abund	Formula	Ion
246.1695		15689		
277.1283		28614		
284.96		9582		
287.0542		20543		
294.1571		15619		
559.1267	1	165233	C27 H27 O11 S	(M+H)+
560.1299	1	52544	C27 H27 O11 S	(M+H)+
561.1284	1	17263	C27 H27 O11 S	(M+H)+
581.1074		26427	C27 H26 Na O11 S	(M+Na)+
922.0098		10061		

Figure S78. HRESIMS of compound **JP-26**

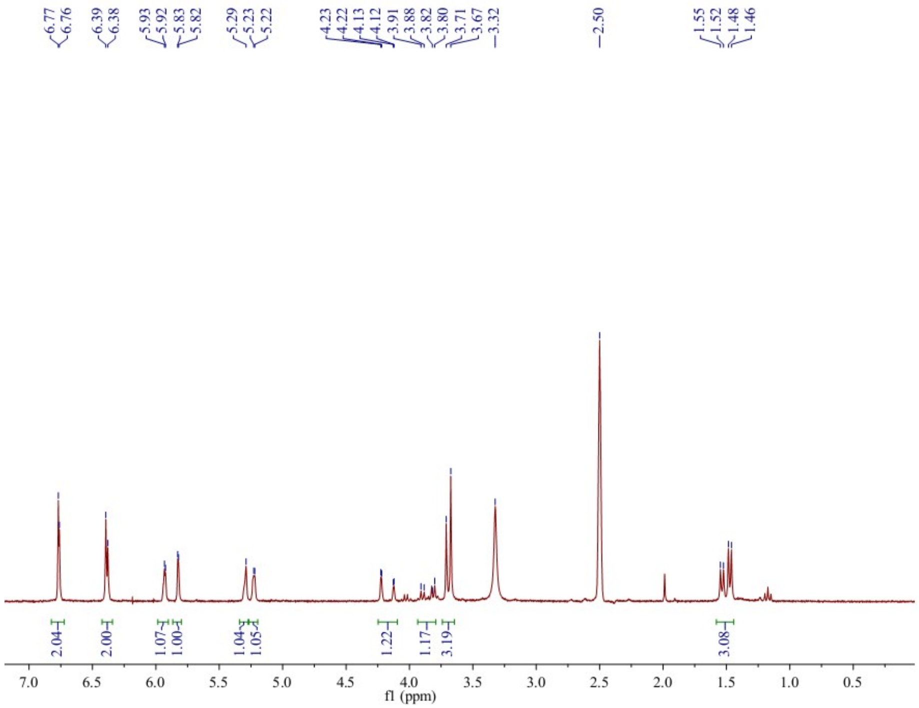

Figure S79. ^{1}H NMR of compound **JP-27** (DMSO-d_6)

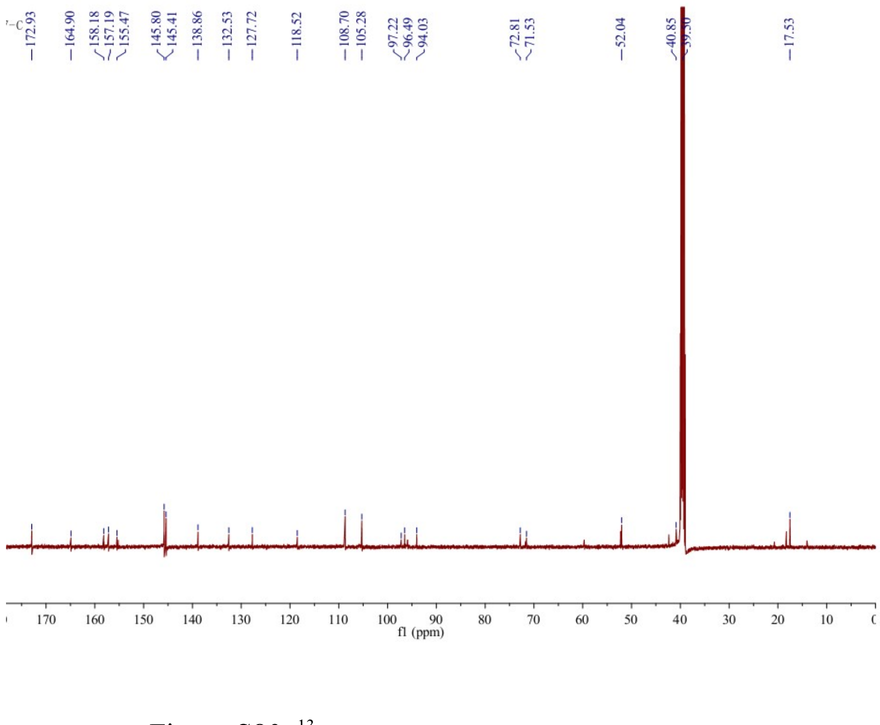

Figure S80. ^{13}C NMR of compound **JP-27** (DMSO-d_6)

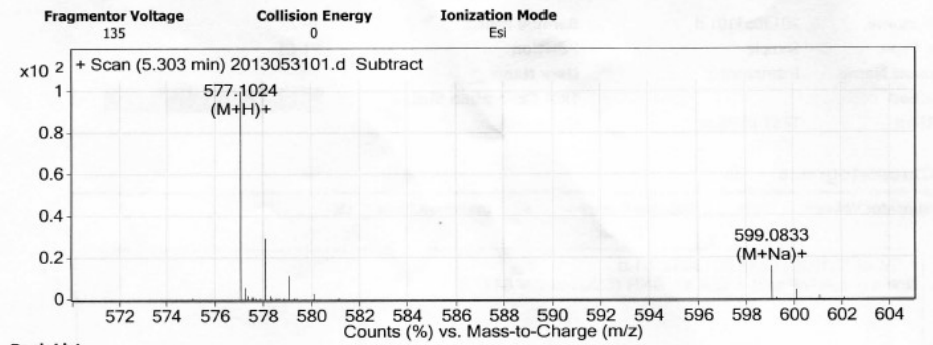

Peak List

m/z	z	Abund	Formula	Ion
123.1163		78883		
151.1106		48491		
169.1215		97574		
246.1697		223196		
251.1319		51715		
262.1649		73598		
577.1024	1	764612	C26 H25 O13 S	(M+H)+
578.1052	1	223019	C26 H25 O13 S	(M+H)+
579.1034	1	85962	C26 H25 O13 S	(M+H)+
599.0833		118250	C26 H24 Na O13 S	(M+Na)+

Figure S81. HRESIMS of compound **JP-27**

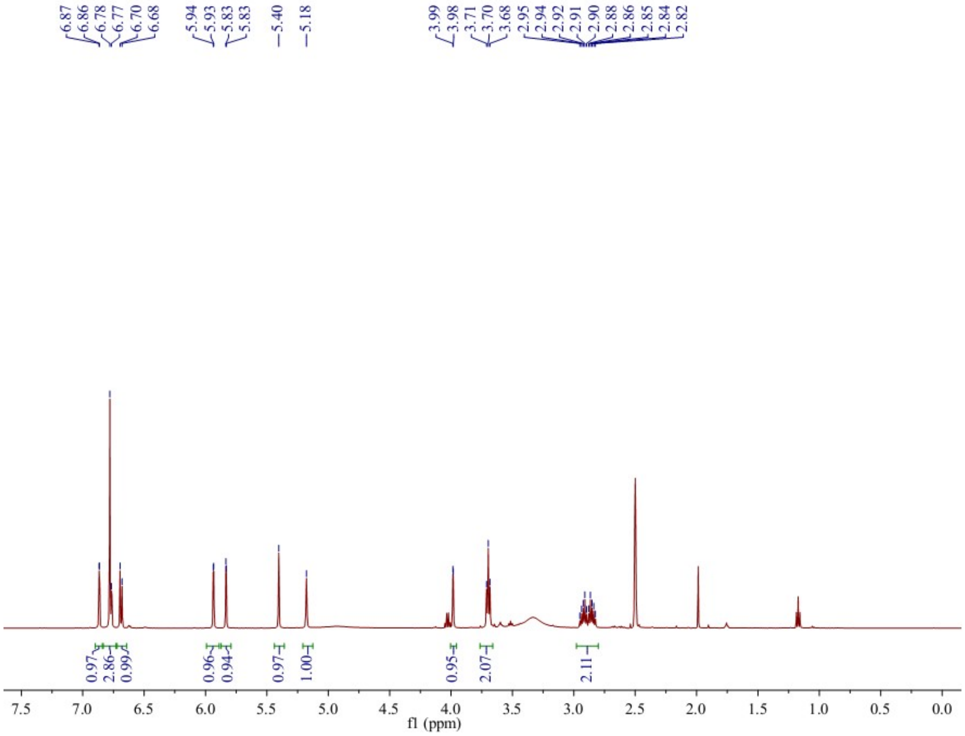

Figure S82. ^{1}H NMR of compound **JP-28** (DMSO-d_6)

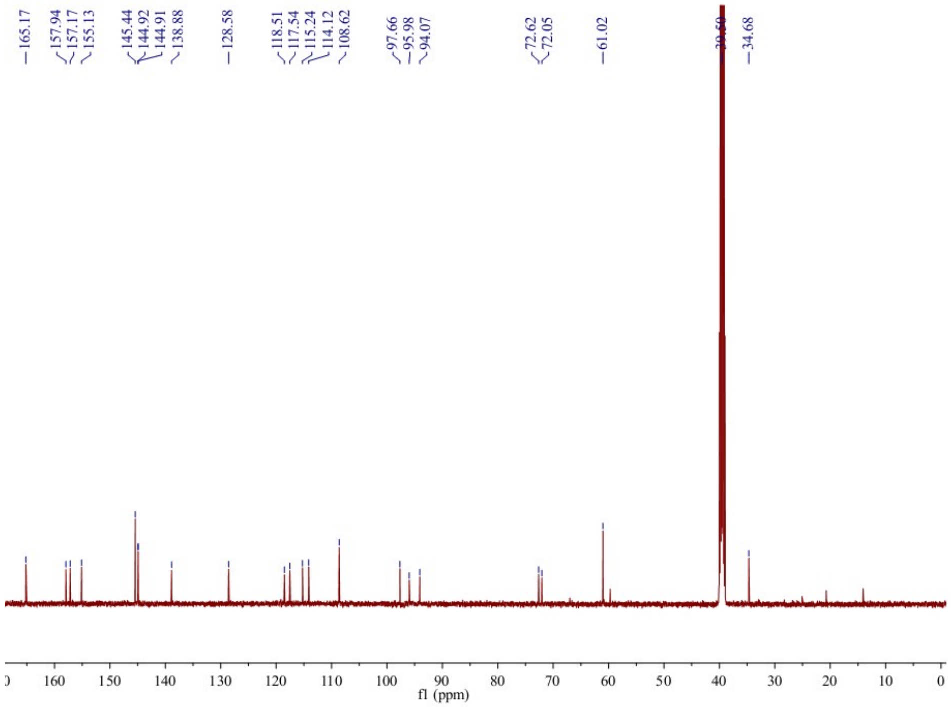

Figure S83. ^{13}C NMR of compound **JP-28** (DMSO-d_6)

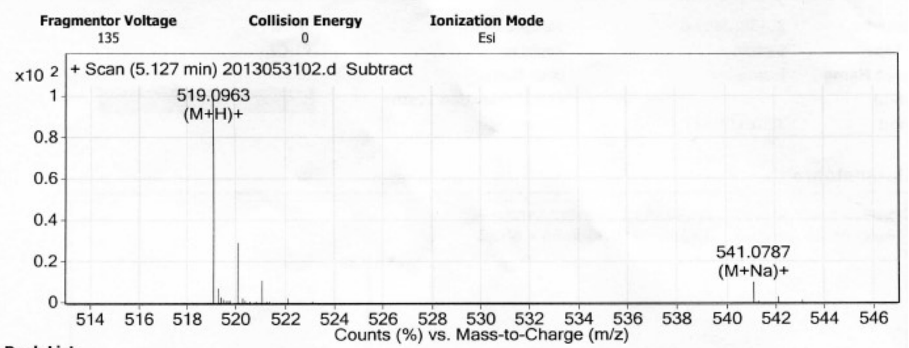

Peak List

m/z	z	Abund	Formula	Ion
441.0827	1	387153		
442.0856	1	97690		
519.0963	1	1833096	C24 H23 O11 S	(M+H)+
519.2892		125479		
520.1005	1	537954	C24 H23 O11 S	(M+H)+
521.0981	1	195682	C24 H23 O11 S	(M+H)+
541.0787		183533	C24 H22 Na O11 S	(M+Na)+
1059.1669	1	176300		
1060.1697	1	98567		

Figure S84. HRESIMS of compound **JP-28**

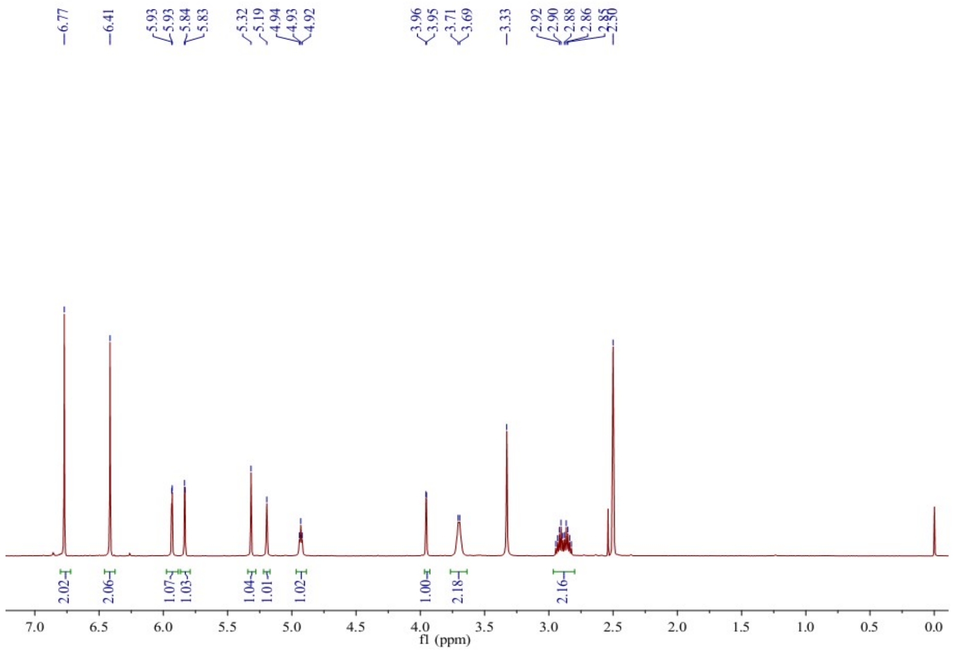

Figure S85. ^{1}H NMR of compound **JP-29** (DMSO-d_6)

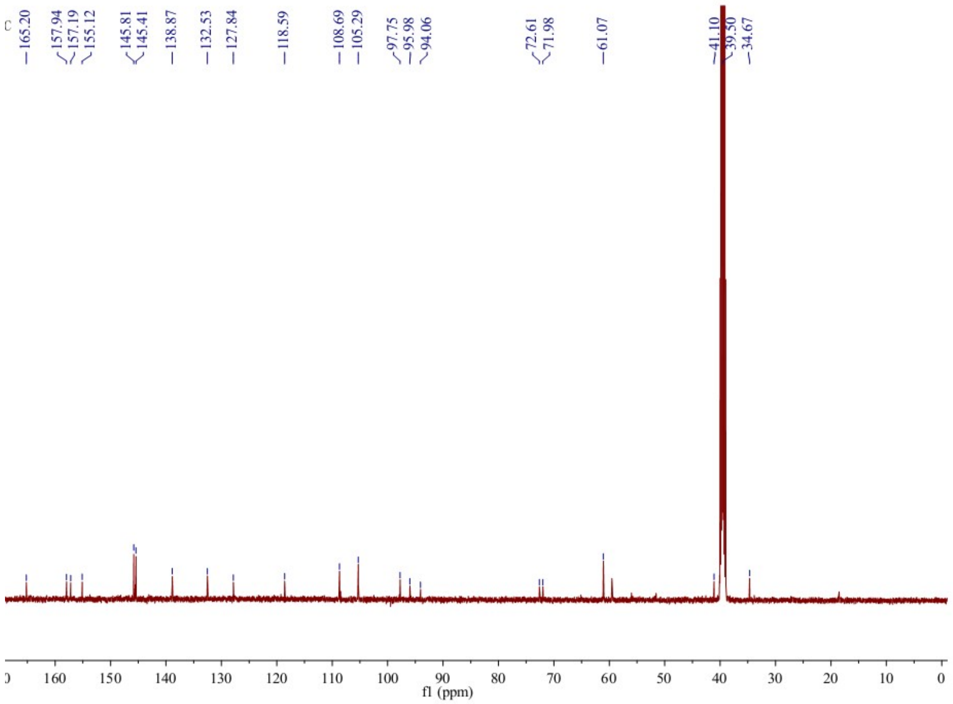

Figure S86. ^{13}C NMR of compound **JP-29** (DMSO-d_6)

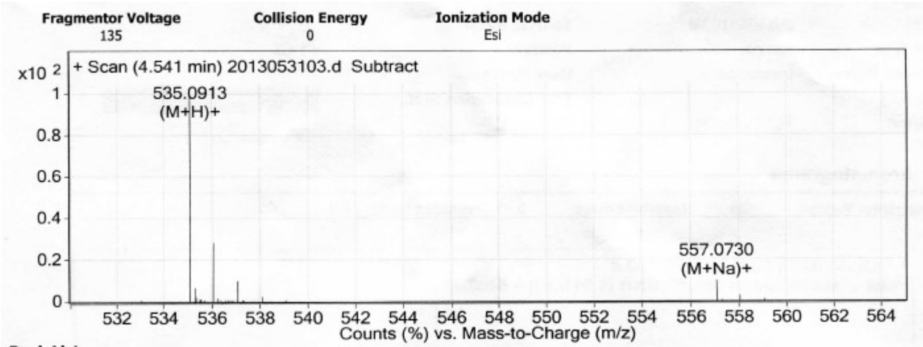

Peak List

m/z	z	Abund	Formula	Ion
287.0542		155581		
457.0771		147289		
535.0913	1	1276467	C24 H23 O12 S	(M+H)+
535.2834		80353		
536.0951	1	358624	C24 H23 O12 S	(M+H)+
537.0932	1	126788	C24 H23 O12 S	(M+H)+
557.073		123996	C24 H22 Na O12 S	(M+Na)+
1091.1559		90326		

Figure S87. HRESIMS of compound **JP-29**

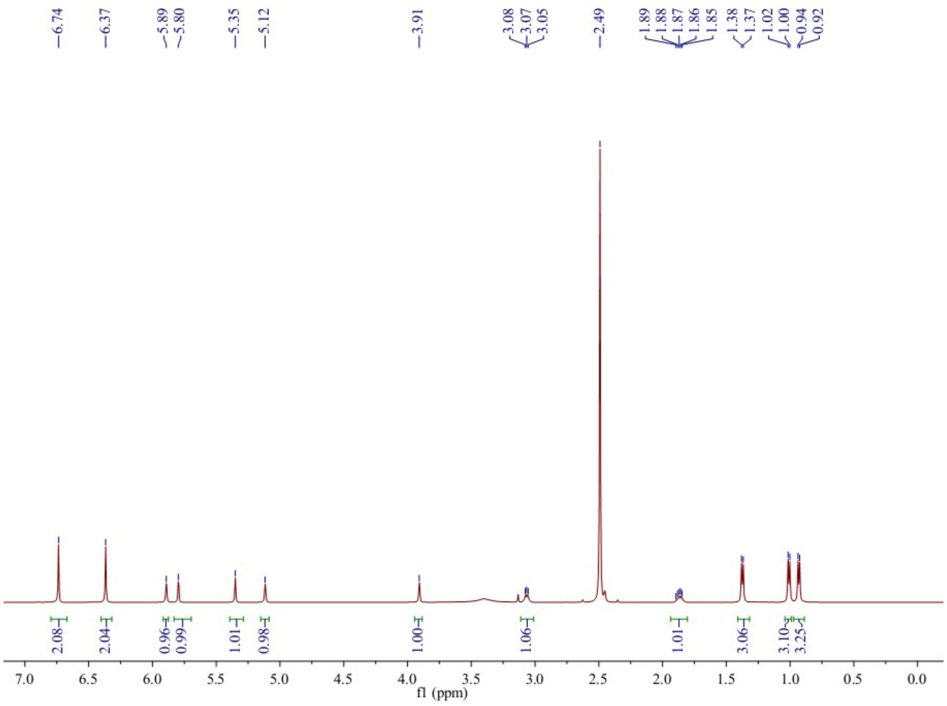

Figure S88. ^{1}H NMR of compound **JP-30** (DMSO-d_6)

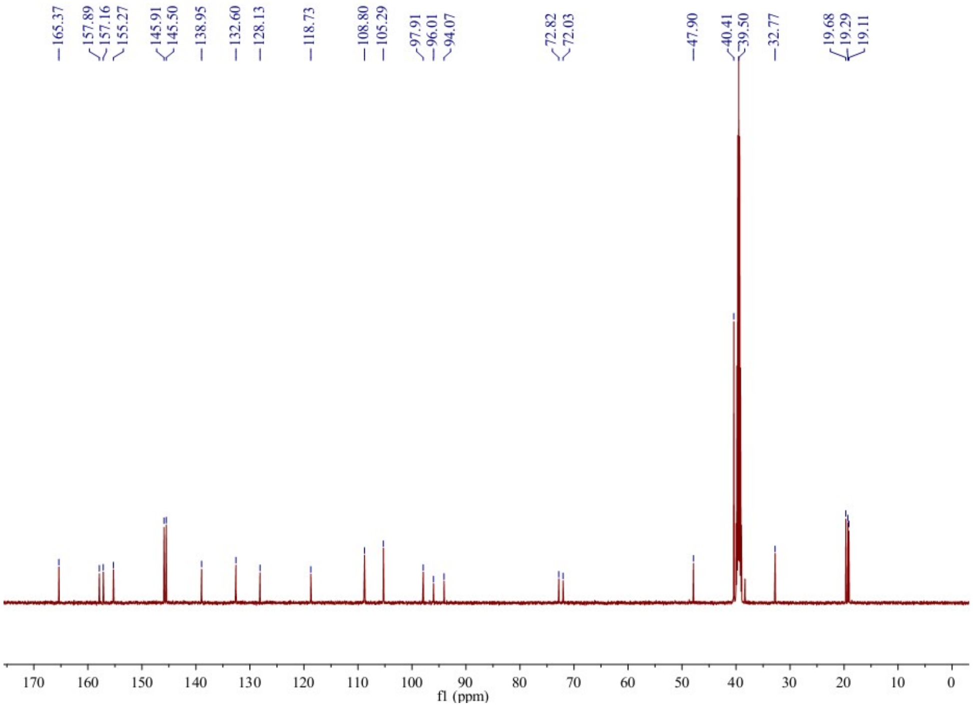

Figure S89. ^{13}C NMR of compound **JP-30** (DMSO-d_6)

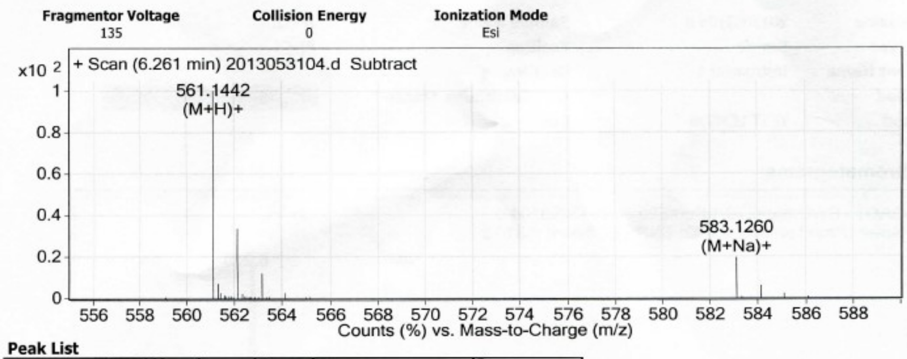

Peak List

m/z	z	Abund	Formula	Ion
287.0558		418742		
457.0781		336955		
561.1442	1	1913532	C27 H29 O11 S	(M+H)+
562.1484	1	643217	C27 H29 O11 S	(M+H)+
563.1464	1	228398	C27 H29 O11 S	(M+H)+
583.126	1	373040	C27 H28 Na O11 S	(M+Na)+
913.1477	1	339315		
914.1508	1	169640		
1143.2616	1	223077		
1144.2648	1	138912		

Figure S90. HRESIMS of compound **JP-30**

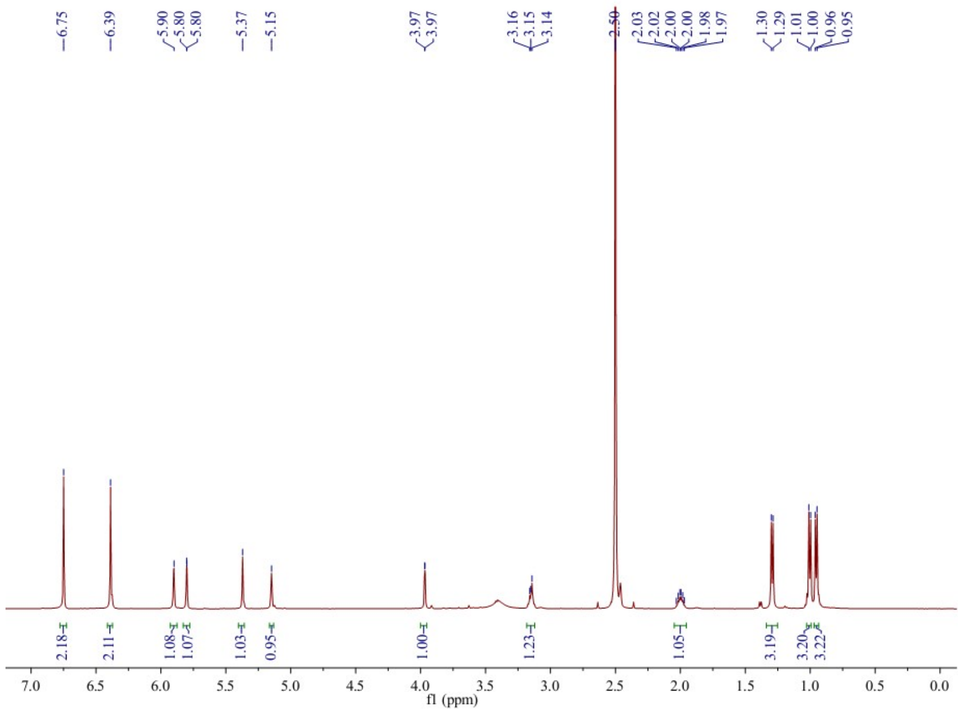

Figure S91. ^{1}H NMR of compound **JP-31** (DMSO-d_6)

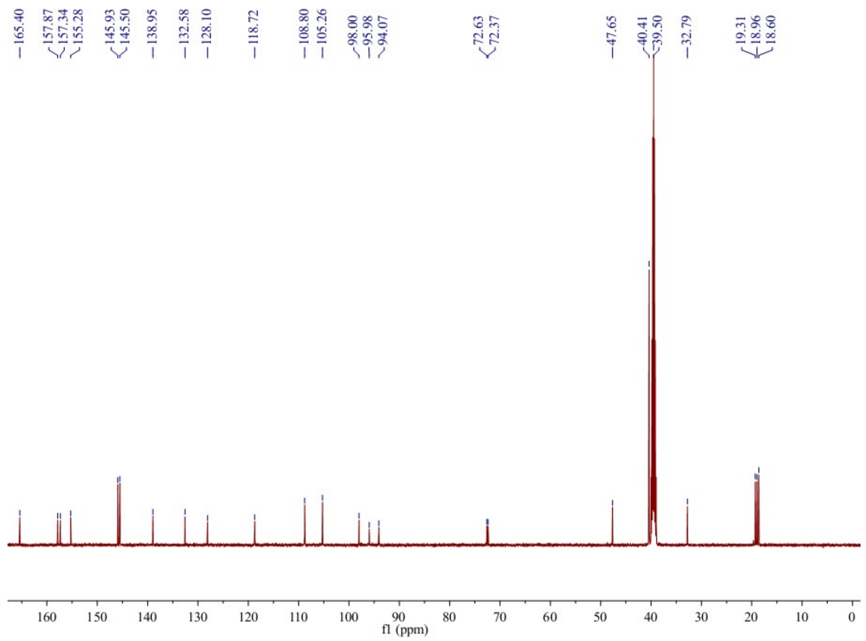

Figure S92. ^{13}C NMR of compound **JP-31** (DMSO-d_6)

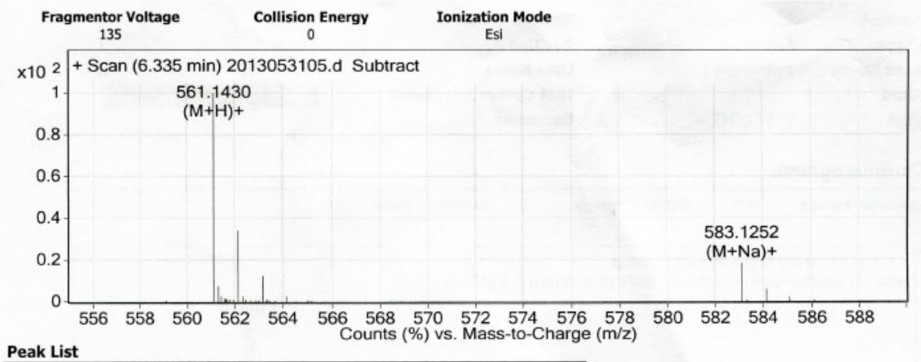

Peak List

m/z	z	Abund	Formula	Ion
287.0557		583719		
457.0775	1	501795		
561.143	1	2269394	C27 H29 O11 S	(M+H)+
562.1476	1	775663	C27 H29 O11 S	(M+H)+
563.1454	1	285019	C27 H29 O11 S	(M+H)+
583.1252	1	412824	C27 H28 Na O11 S	(M+Na)+
913.1462	1	595781		
914.1493	1	301561		
1143.2589	1	319335		
1144.2622	1	200091		

Figure S93. HRESIMS of compound **JP-31**

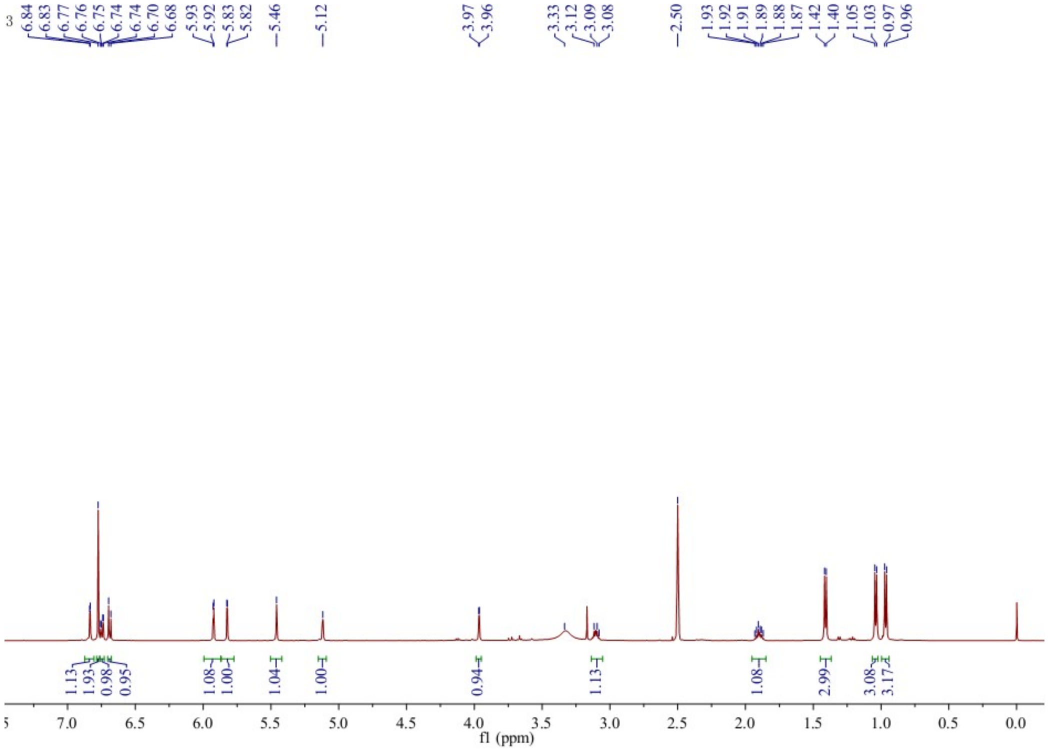

Figure S94. ^{1}H NMR of compound **JP-32** (DMSO-d_6)

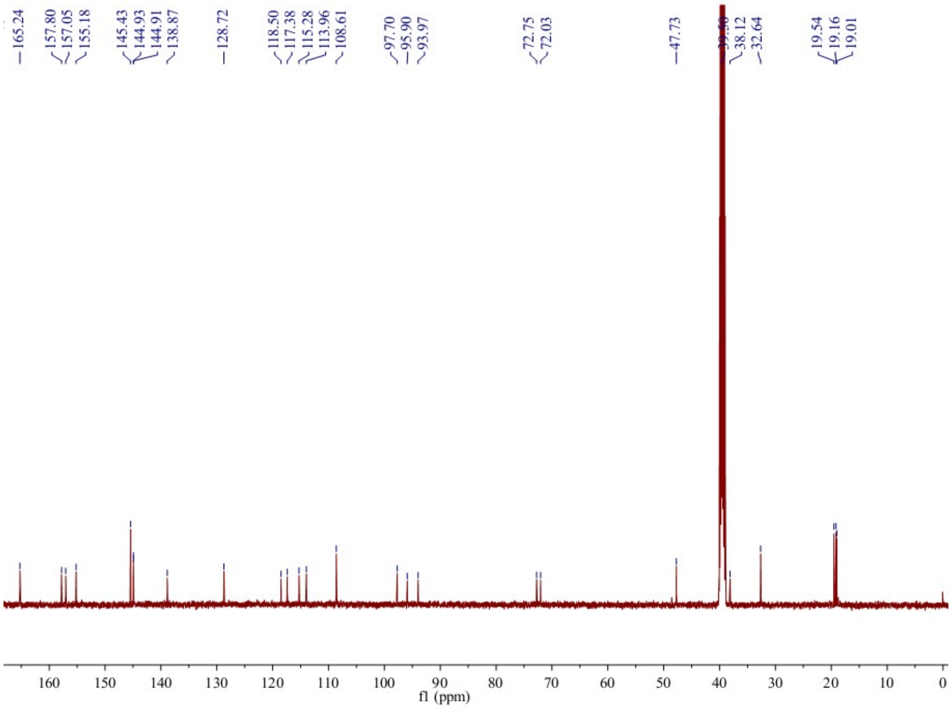

Figure S95. ^{13}C NMR of compound **JP-32** (DMSO-d_6)

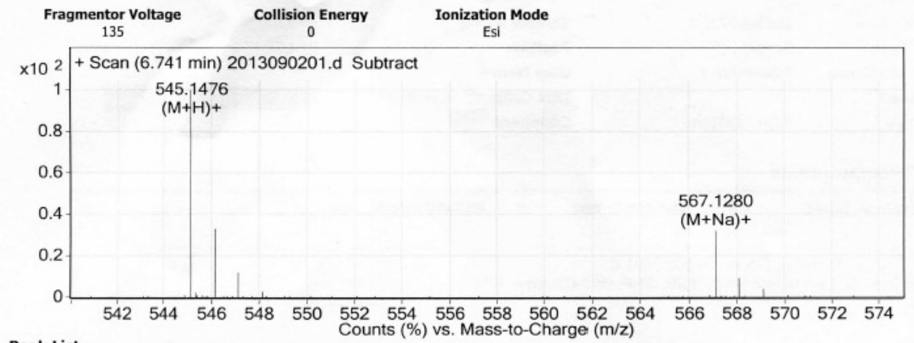

Peak List

m/z	z	Abund	Formula	Ion
110.0087		21137		
122.0707		14628		
125.9862		34290		
142.0347		15507		
158.9651		15869		
223.0982		21532		
545.1476	1	77522	C27 H29 O10 S	(M+H)+
546.1505	1	25642	C27 H29 O10 S	(M+H)+
567.128	1	24837	C27 H28 Na O10 S	(M+Na)+
922.0098	1	32135		

Figure S96. HRESIMS of compound **JP-32**

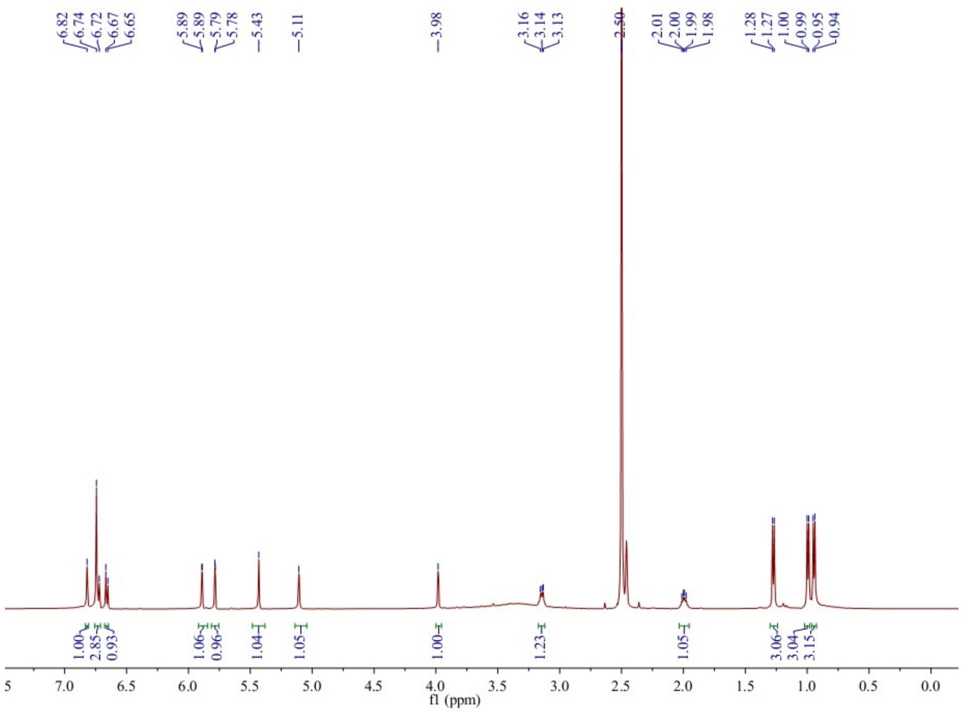

Figure S97. ^{1}H NMR of compound **JP-33** (DMSO-d_6)

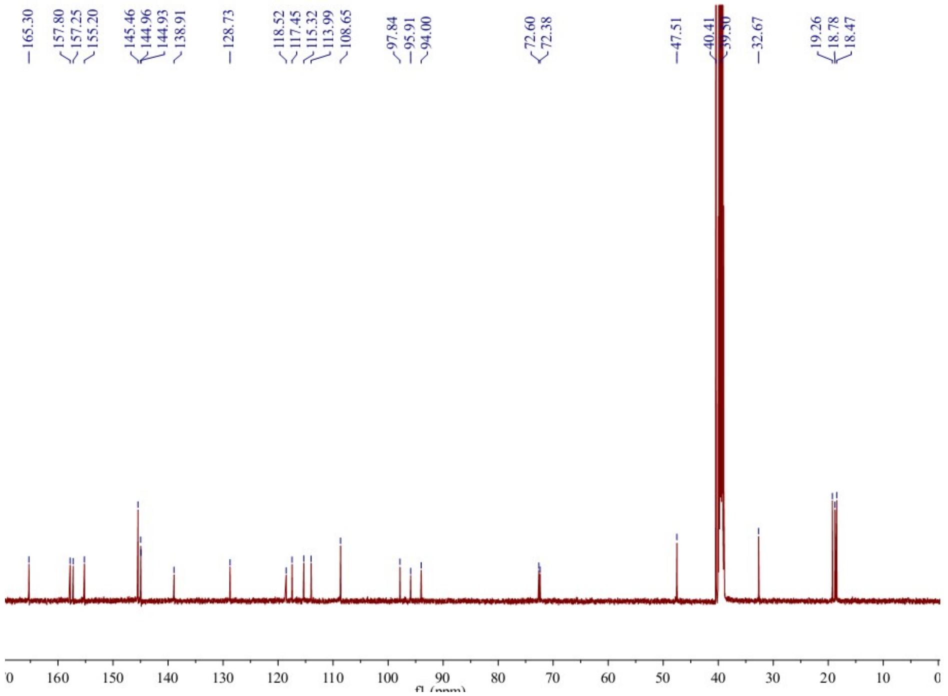

Figure S98. ^{13}C NMR of compound **JP-33** (DMSO-d_6)

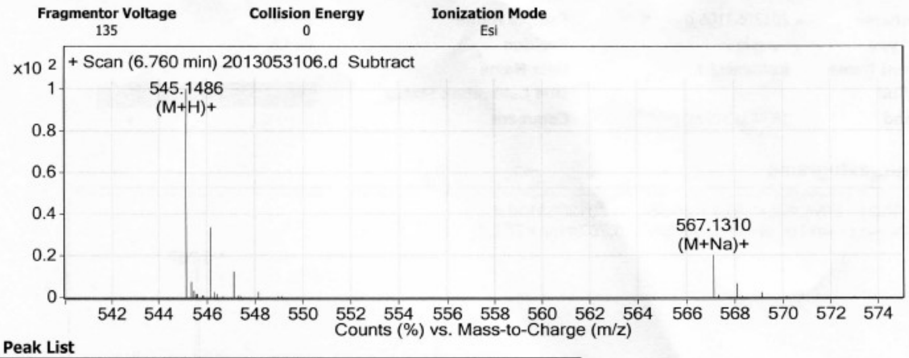

Peak List

m/z	z	Abund	Formula	Ion
441.083	1	1167640		
442.0864	1	310686		
545.1486	1	2659902	C27 H29 O10 S	(M+H)+
546.1528	1	894363	C27 H29 O10 S	(M+H)+
547.1515	1	327461	C27 H29 O10 S	(M+H)+
567.131	1	529237	C27 H28 Na O10 S	(M+Na)+
881.157	1	586311		
882.1603	1	290799		
1111.2701	1	505098		
1112.2737	1	320037		

Figure S99. HRESIMS of compound **JP-33**

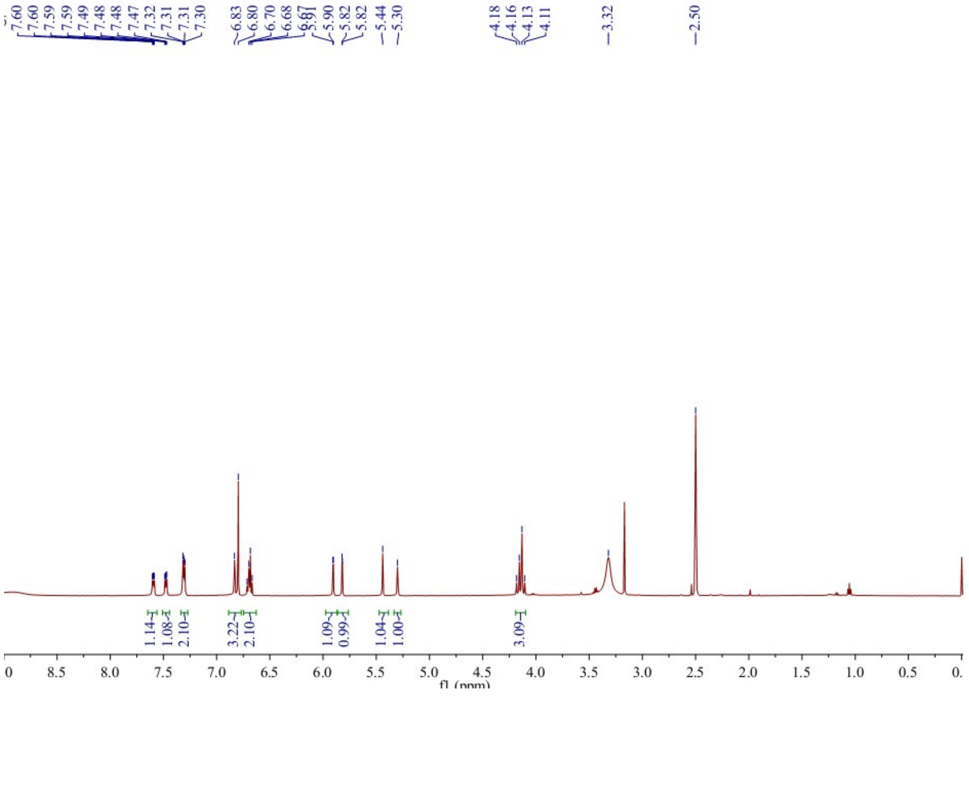

Figure S100. ^{1}H NMR of compound **JP-34** (DMSO-d_6)

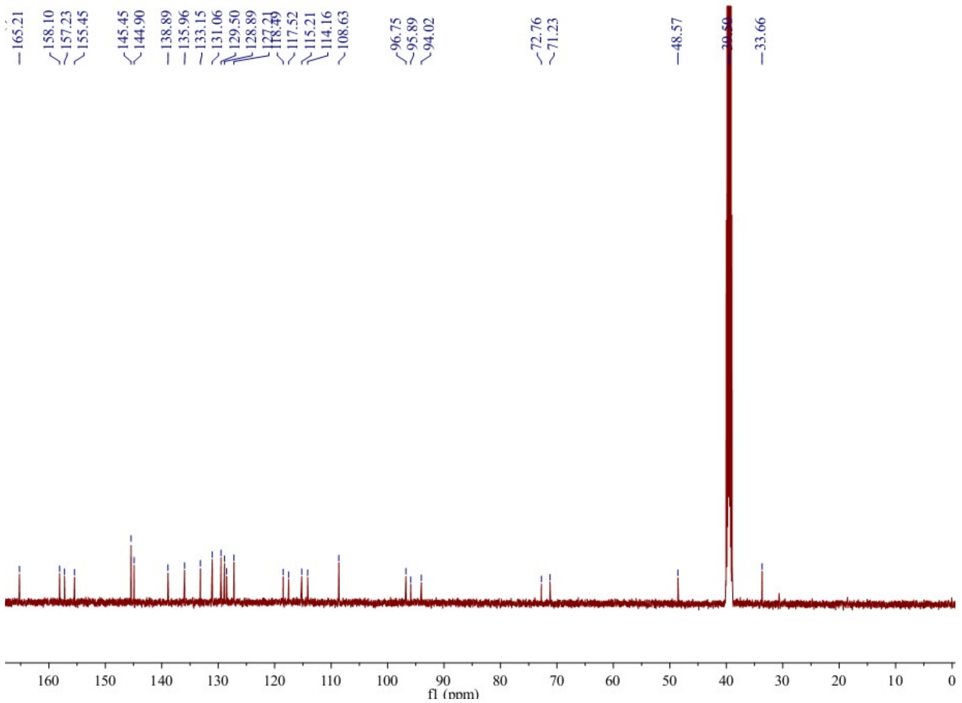

Figure S101. ^{13}C NMR of compound **JP-34** (DMSO-d_6)

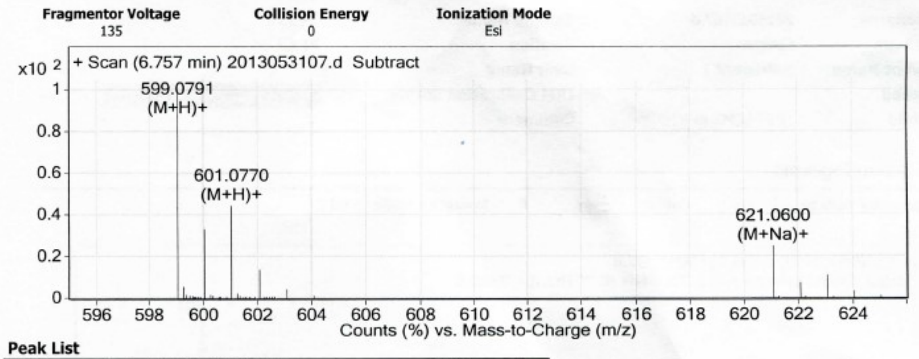

Peak List

m/z	z	Abund	Formula	Ion
271.06		53976		
429.0567		48484		
441.0824	1	193741		
599.0791	1	799718	C29 H24 Cl O10 S	(M+H)+
600.0816	1	264475	C29 H24 Cl O10 S	(M+H)+
601.077	1	353606	C29 H24 Cl O10 S	(M+H)+
602.0791	1	106466	C29 H24 Cl O10 S	(M+H)+
621.06	1	197906	C29 H23 Cl Na O10 S	(M+Na)+
622.0537	1	57984	C29 H23 Cl Na O10 S	(M+Na)+
623.0571	1	85862	C29 H23 Cl Na O10 S	(M+Na)+

Figure S102. HRESIMS of compound **JP-34**

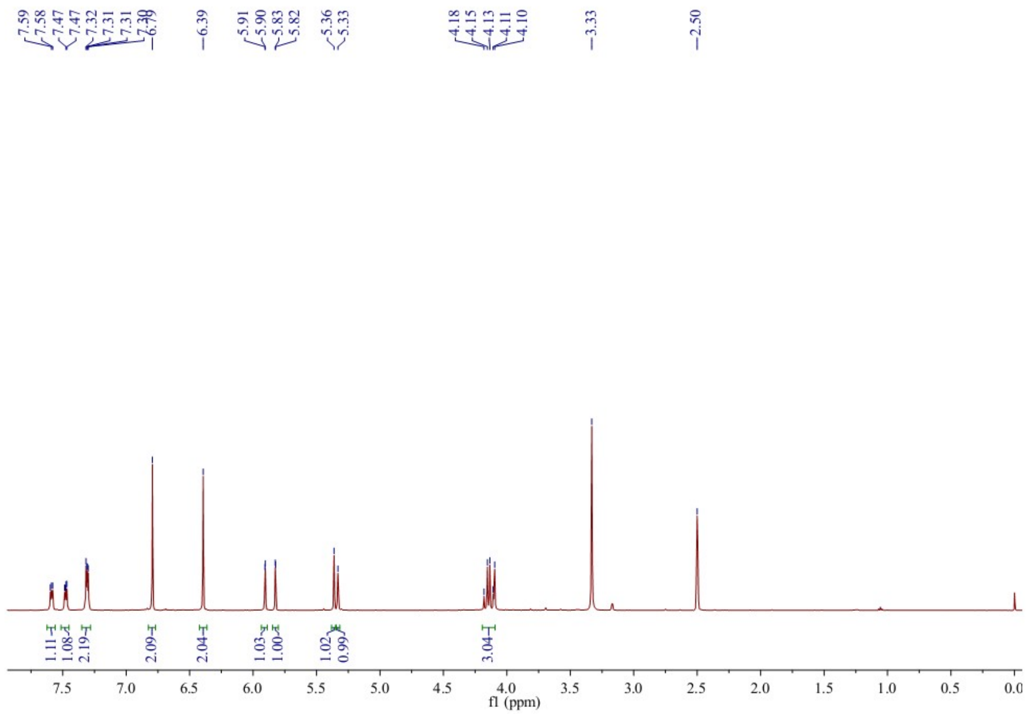

Figure S103. ^{1}H NMR of compound **JP-35** (DMSO-d_6)

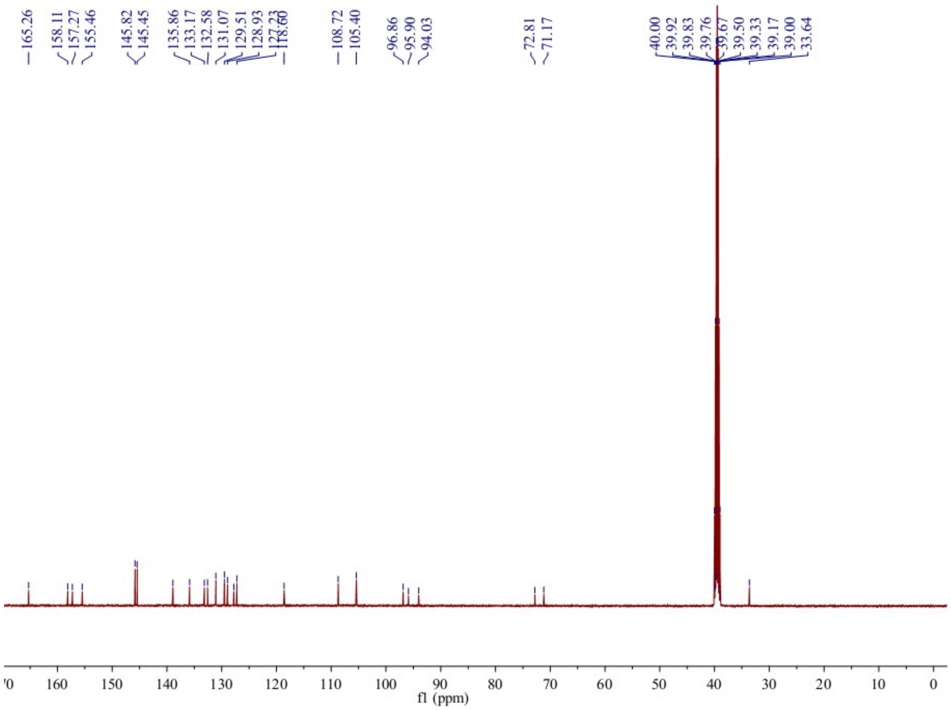

Figure S104. ^{13}C NMR of compound **JP-35** (DMSO-d_6)

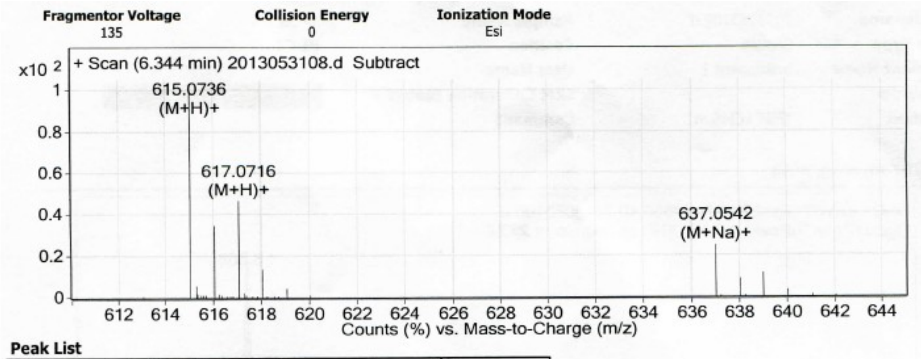

Peak List

m/z	z	Abund	Formula	Ion
287.0551		181057		
445.0507		76576		
457.0773		146418		
615.0736	1	880476	C29 H24 Cl O11 S	(M+H)+
616.0765	1	303254	C29 H24 Cl O11 S	(M+H)+
617.0716	1	408490	C29 H24 Cl O11 S	(M+H)+
618.0735	1	117258	C29 H24 Cl O11 S	(M+H)+
637.0542	1	220129	C29 H23 Cl Na O11 S	(M+Na)+
638.0568	1	76628	C29 H23 Cl Na O11 S	(M+Na)+
639.0521	1	99828	C29 H23 Cl Na O11 S	(M+Na)+

Figure S105. HRESIMS of compound **JP-35**

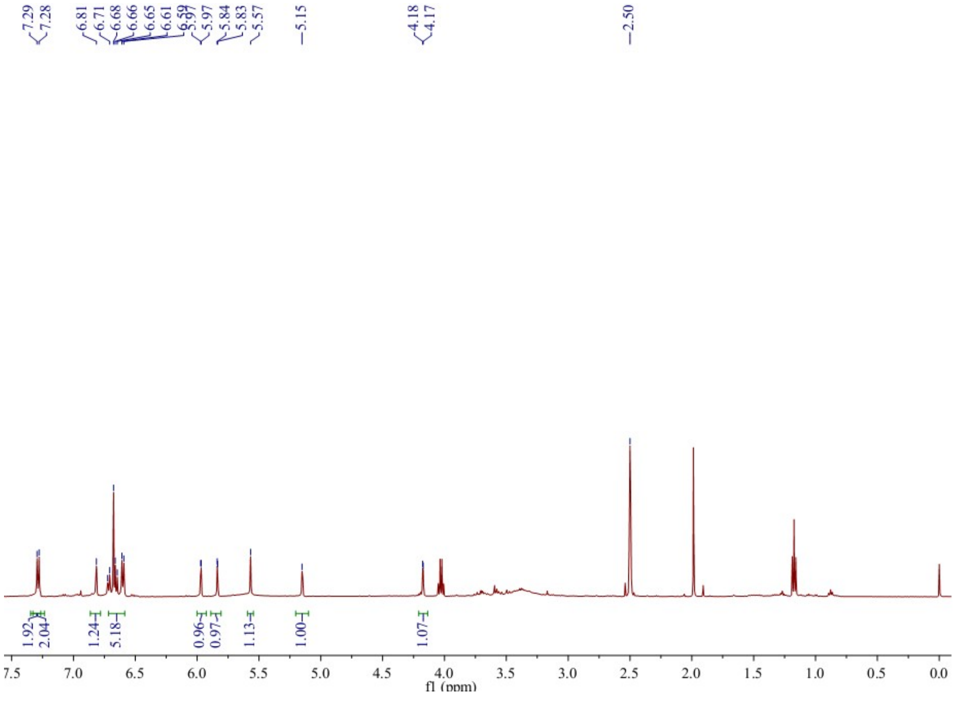

Figure S106. ^{1}H NMR of compound **JP-36** (DMSO-d_6)

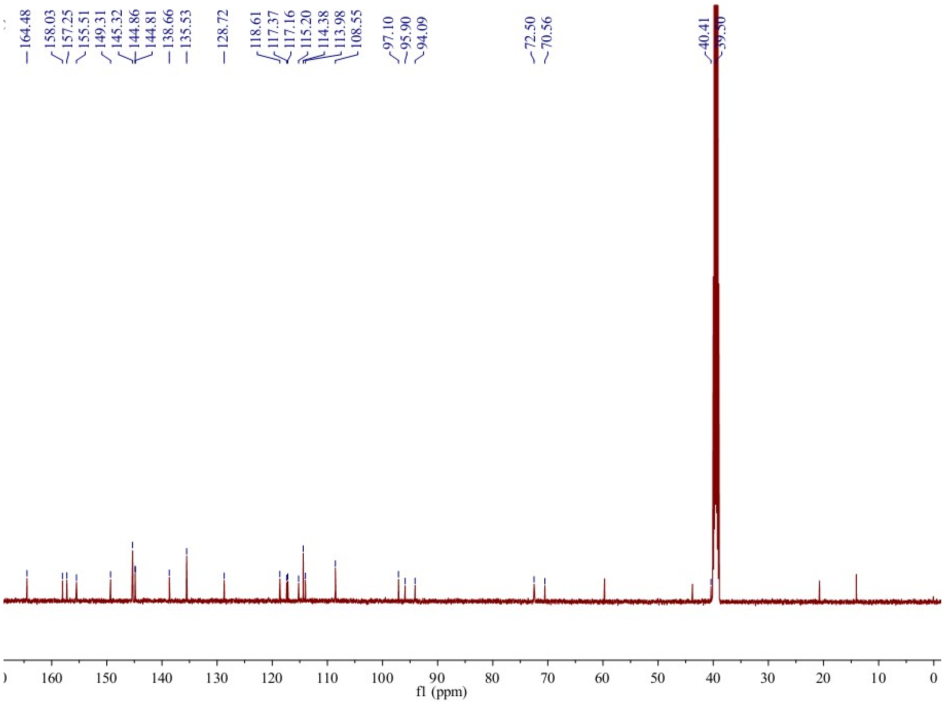

Figure S107. ^{13}C NMR of compound **JP-36** (DMSO-d_6)

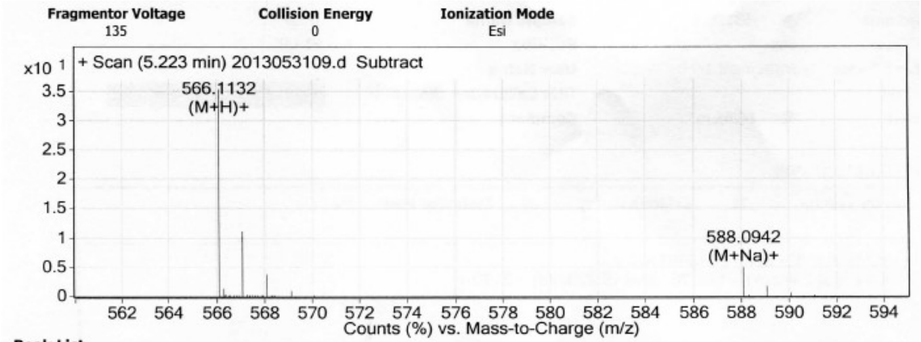

Peak List

m/z	z	Abund	Formula	Ion
107.0413		92430		
169.1108		144800		
271.06		78151		
396.0906		99936		
441.083	1	1089506		
441.2613		62471		
442.0864	1	277462		
443.0884	1	55364		
566.1132	1	384019	C28 H24 N O10 S	(M+H)+
567.1163	1	120577	C28 H24 N O10 S	(M+H)+

Figure S108. HRESIMS of compound **JP-36**

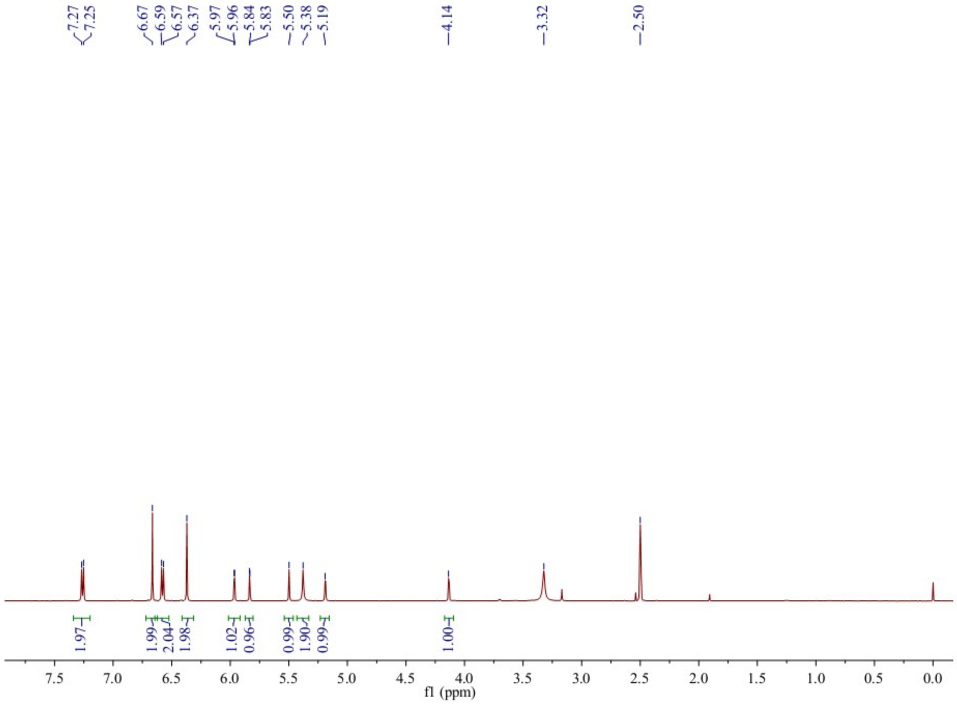

Figure S109. ^{1}H NMR of compound **JP-37** (DMSO-d_6)

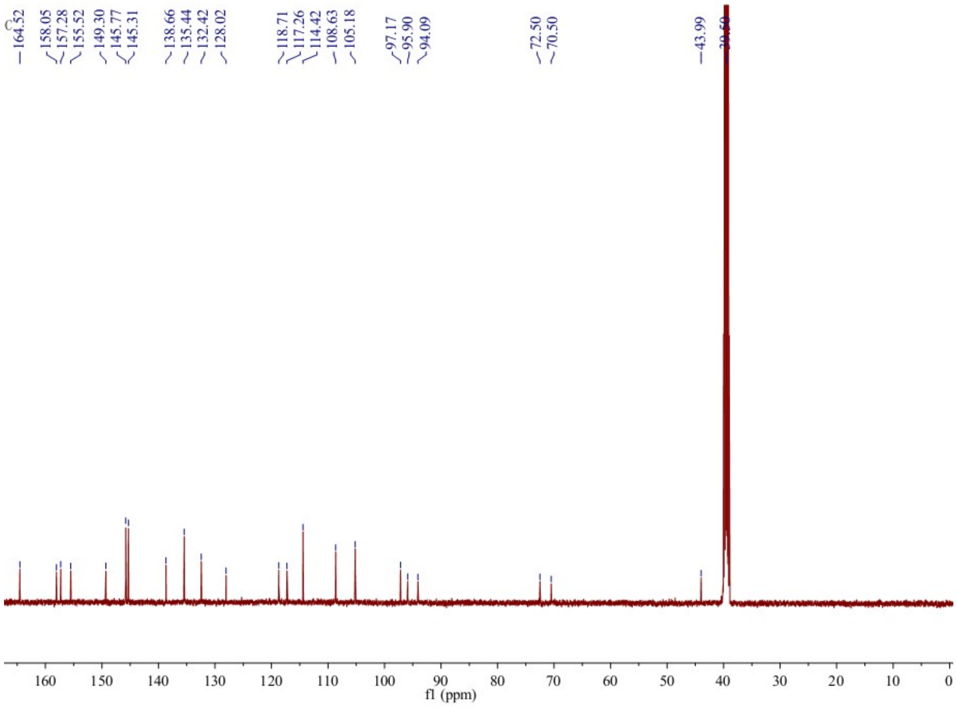

Figure S110. ^{13}C NMR of compound **JP-37** (DMSO-d_6)

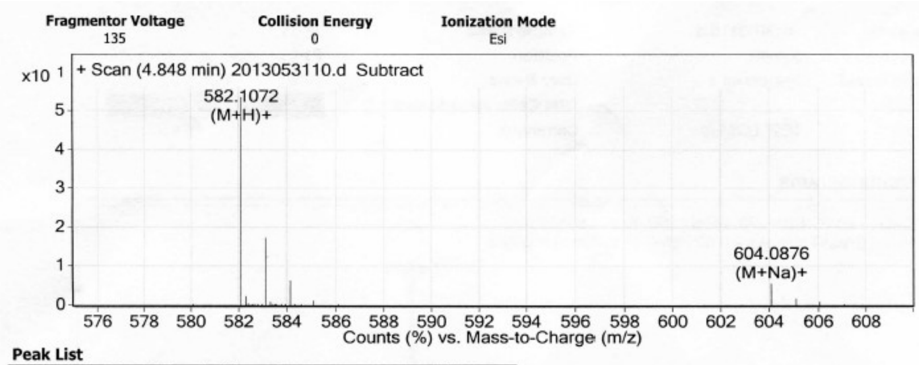

Peak List

m/z	z	Abund	Formula	Ion
287.0555	1	967040		
288.0585	1	163643		
412.0855	1	218413		
457.0775	1	962512		
458.0805	1	245747		
582.1072	1	516422	C28 H24 N O11 S	(M+H)+
583.1099	1	167266	C28 H24 N O11 S	(M+H)+
584.1089	1	60104	C28 H24 N O11 S	(M+H)+
913.1453	1	154641		
914.1486	1	74553		

Figure S111. HRESIMS of compound **JP-37** (DMSO-d_6)

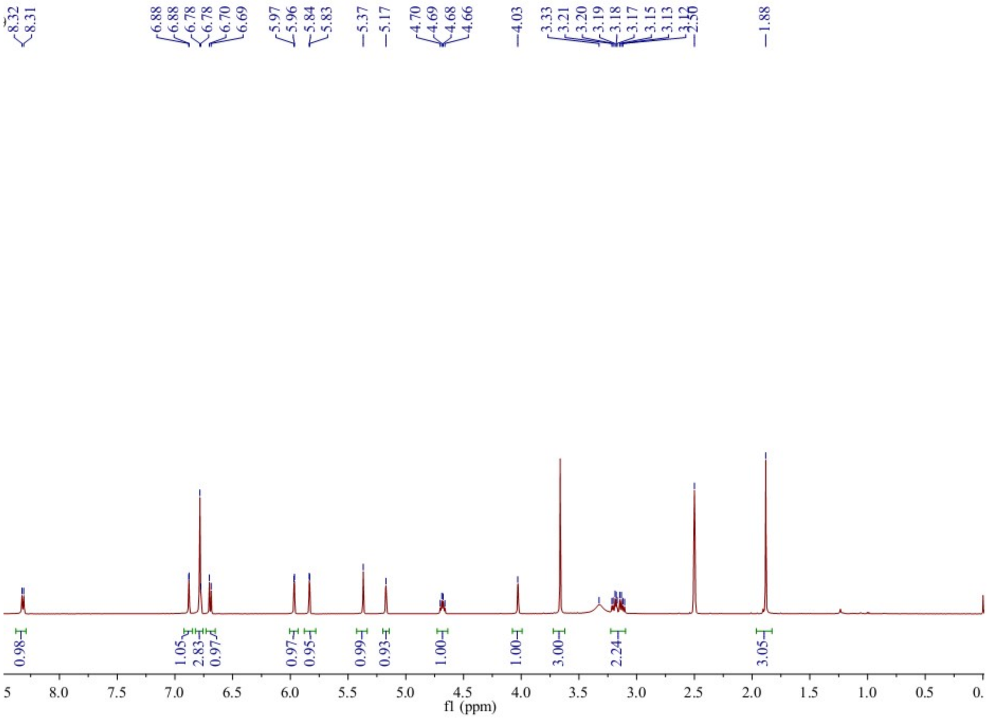

Figure S112. ^{1}H NMR of compound **JP-38** (DMSO-d_6)

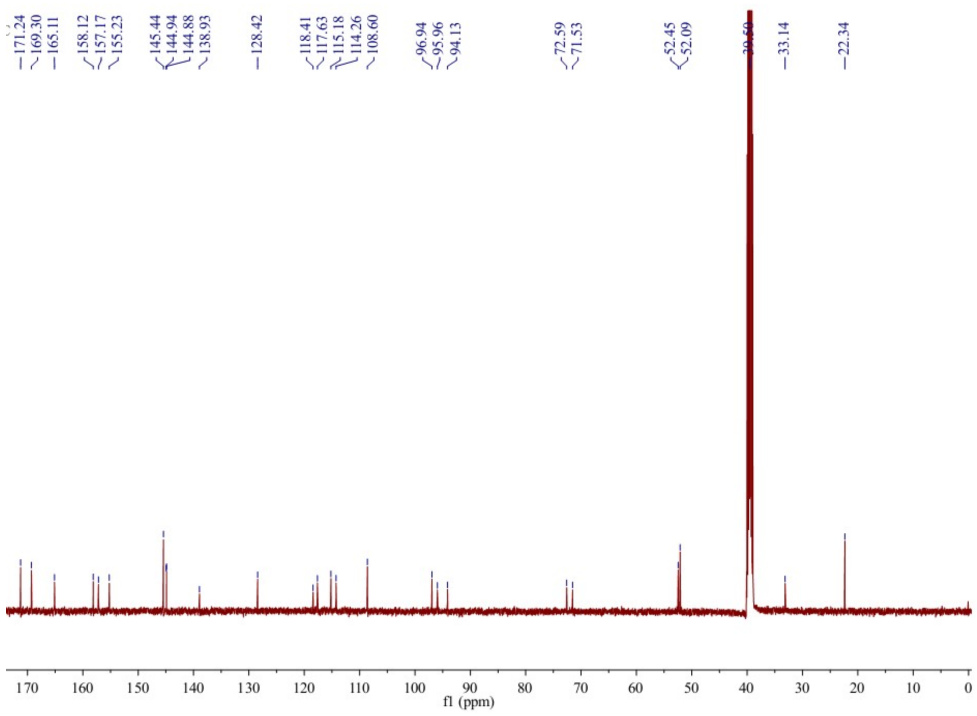

Figure S113. ^{13}C NMR of compound **JP-38** (DMSO-d_6)

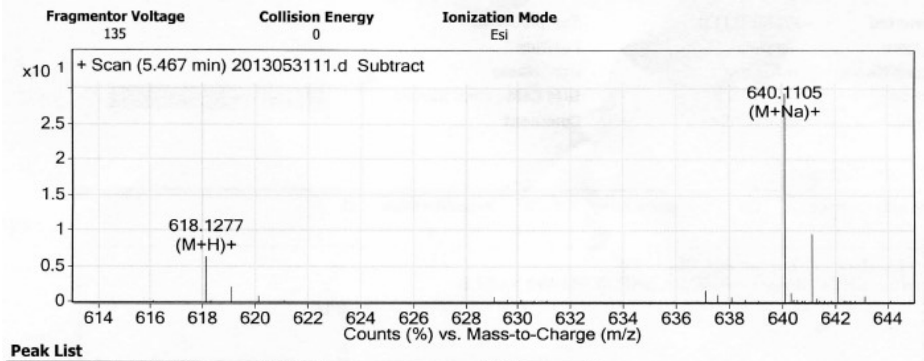

Peak List

m/z	z	Abund	Formula	Ion
441.0822	1	1594191		
441.2607		103136		
442.086	1	403704		
618.1277		100462	C28 H28 N O13 S	(M+H)+
640.1105	1	468174	C28 H27 N Na O13 S	(M+Na)+
641.113	1	152545	C28 H27 N Na O13 S	(M+Na)+
881.156		101506		
1257.2297	1	289100		
1258.2322	1	188729		
1259.2326	1	98908		

Figure S114. HRESIMS of compound **JP-38**

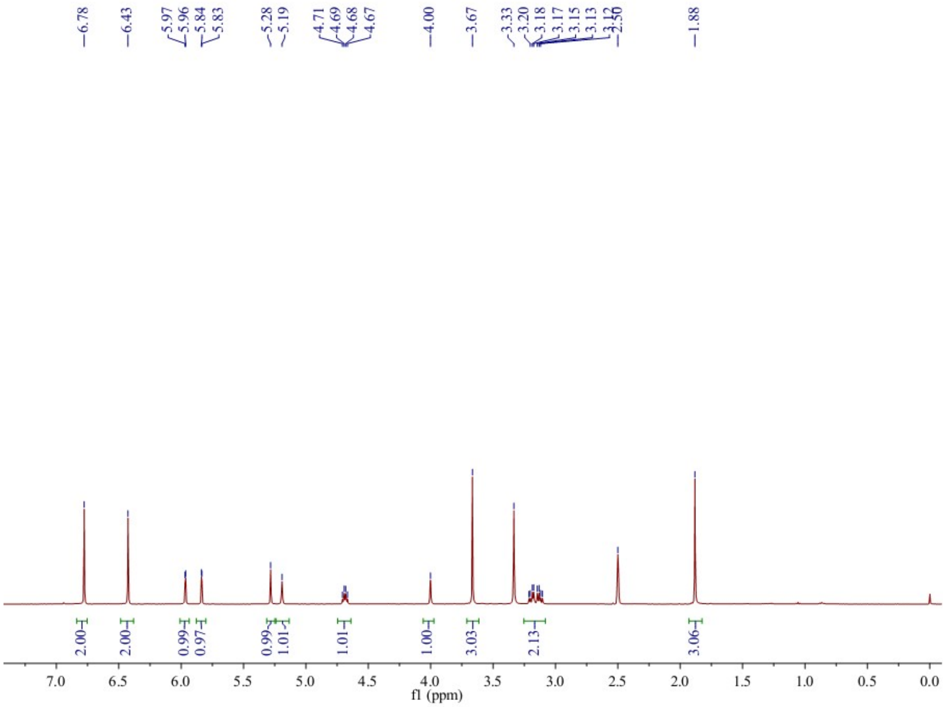

Figure S115. ^{1}H NMR of compound **JP-39** (DMSO-d_6)

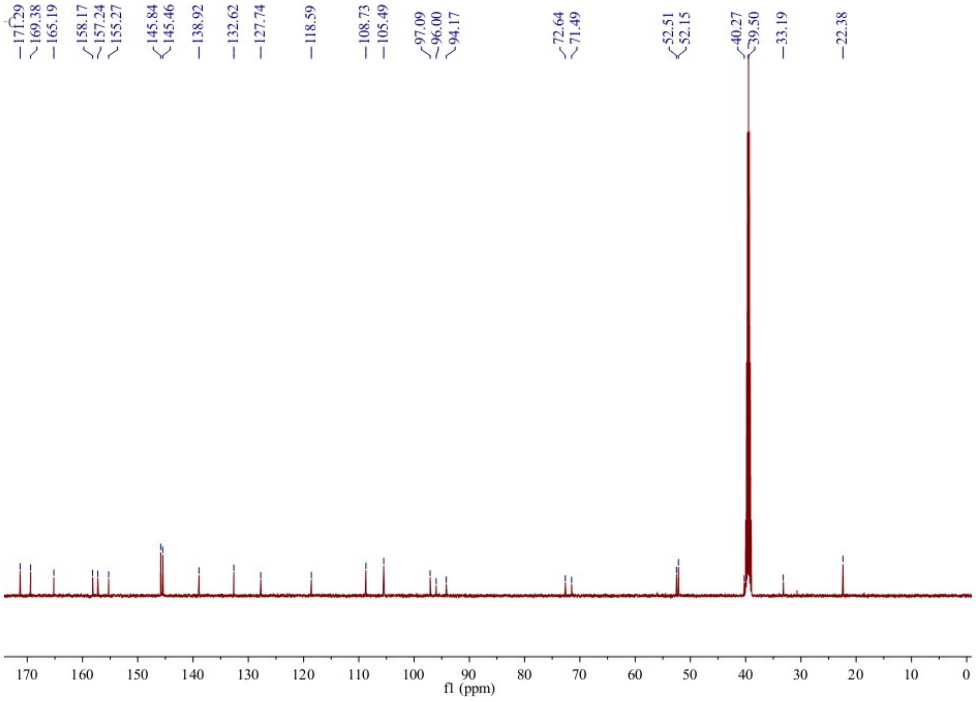

Figure S116. ^{13}C NMR of compound **JP-39** (DMSO-d_6)

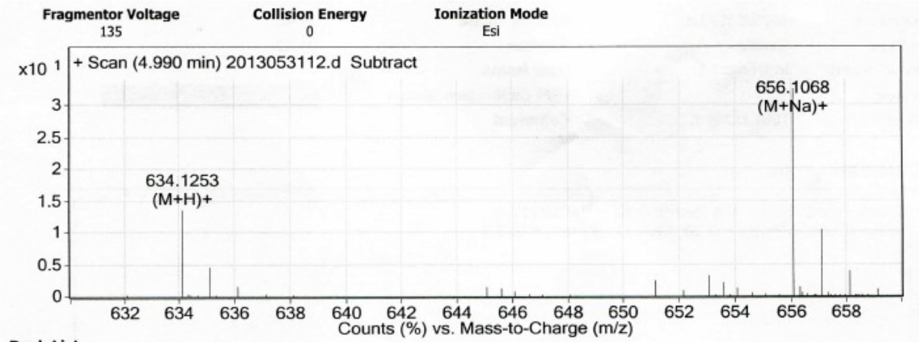

Peak List

m/z	z	Abund	Formula	Ion
287.0557	1	1140365		
288.0592	1	198850		
457.078	1	1578201		
458.0819	1	405190		
634.1253		213207	C28 H28 N O14 S	(M+H)+
656.1068	1	512703	C28 H27 N Na O14 S	(M+Na)+
657.1098	1	163941	C28 H27 N Na O14 S	(M+Na)+
913.1485	1	228321		
1289.2225	1	274707		
1290.226	1	175809		

Figure S117. HRESIMS of compound **JP-39**

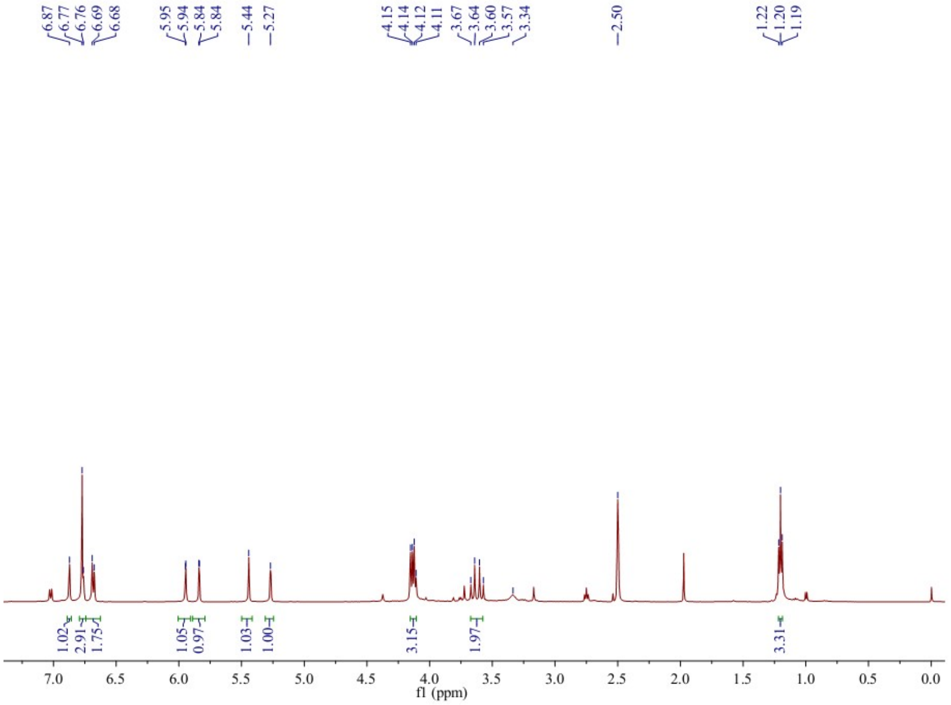

Figure S118. ^{1}H NMR of compound **JP-40** (DMSO-d_6)

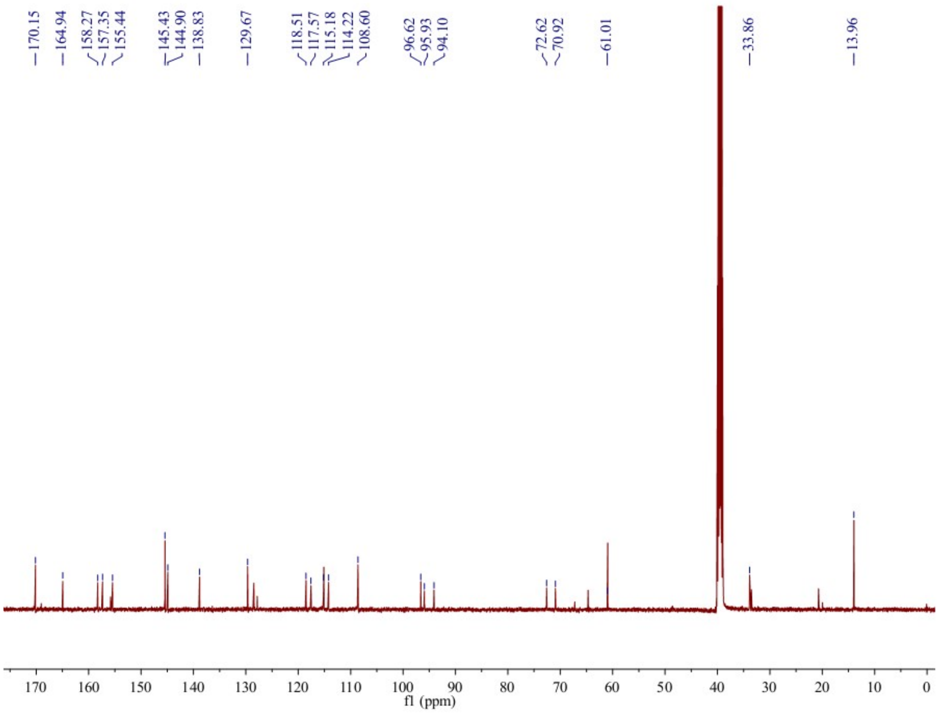

Figure S119. ^{13}C NMR of compound **JP-40** (DMSO-d_6)

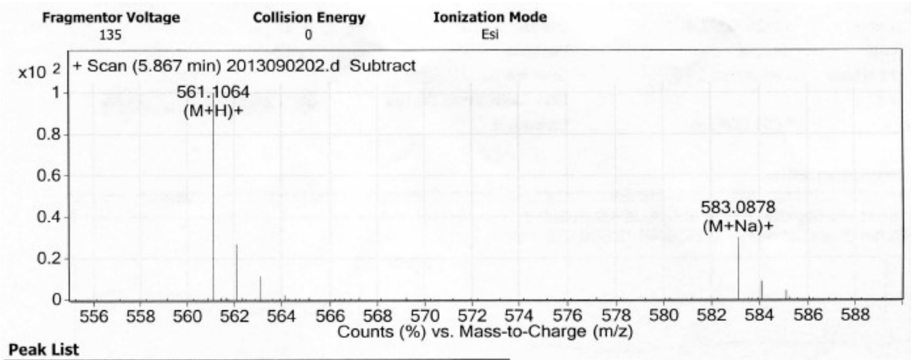

Peak List

m/z	z	Abund	Formula	Ion
125.9865		24807		
206.1389	1	29547		
211.1563	1	55222		
227.1493	1	24300		
251.1628	1	60670		
253.1743	1	25722		
284.9613		42637		
286.9581		42178		
433.2199	1	23364		
561.1064	1	74347	C26 H25 O12 S	(M+H)+

Figure S120. HRESIMS of compound **JP-40**

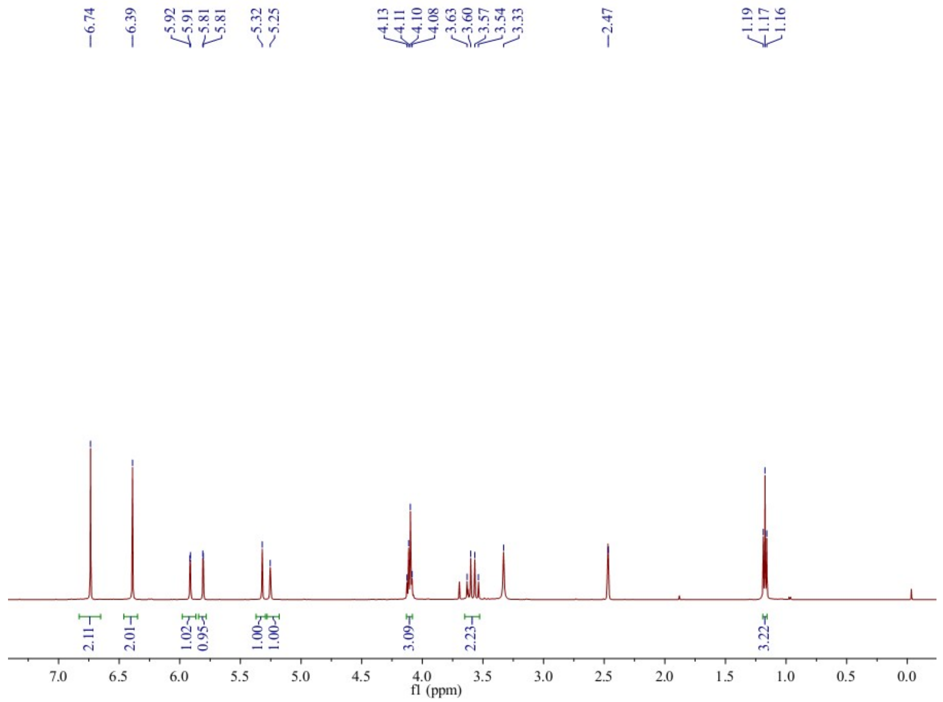

Figure S121. ^{1}H NMR of compound **JP-41** (DMSO-d_6)

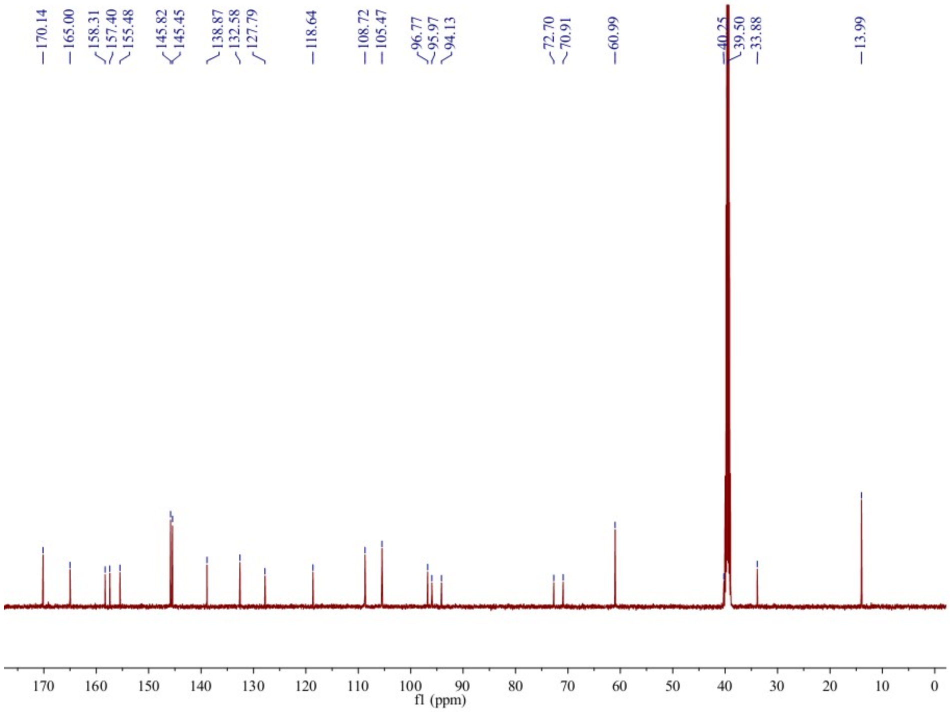

Figure S122. ^{13}C NMR of compound **JP-41** (DMSO-d_6)

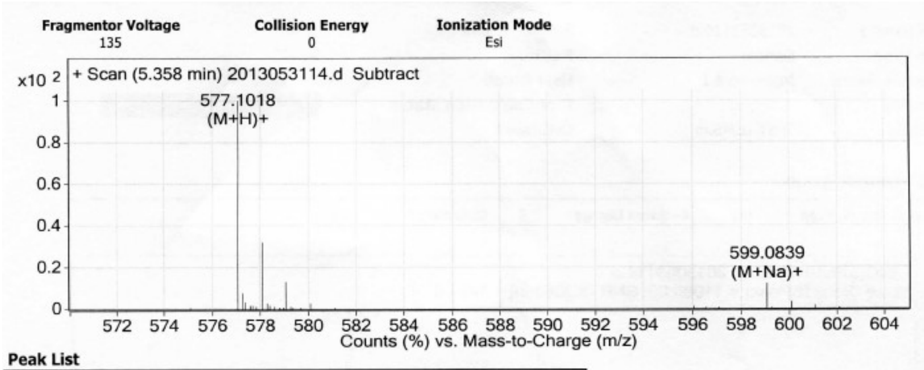

Peak List

m/z	z	Abund	Formula	Ion
287.0554		206102		
457.0771		214624		
577.1018	1	2760391	C26 H25 O13 S	(M+H)+
577.2995		214038		
578.1062	1	884921	C26 H25 O13 S	(M+H)+
579.1045	1	354533	C26 H25 O13 S	(M+H)+
599.0839		323185	C26 H24 Na O13 S	(M+Na)+
1175.1769	1	340817		
1176.1796	1	201670		

Figure S123. HRESIMS of compound **JP-41**

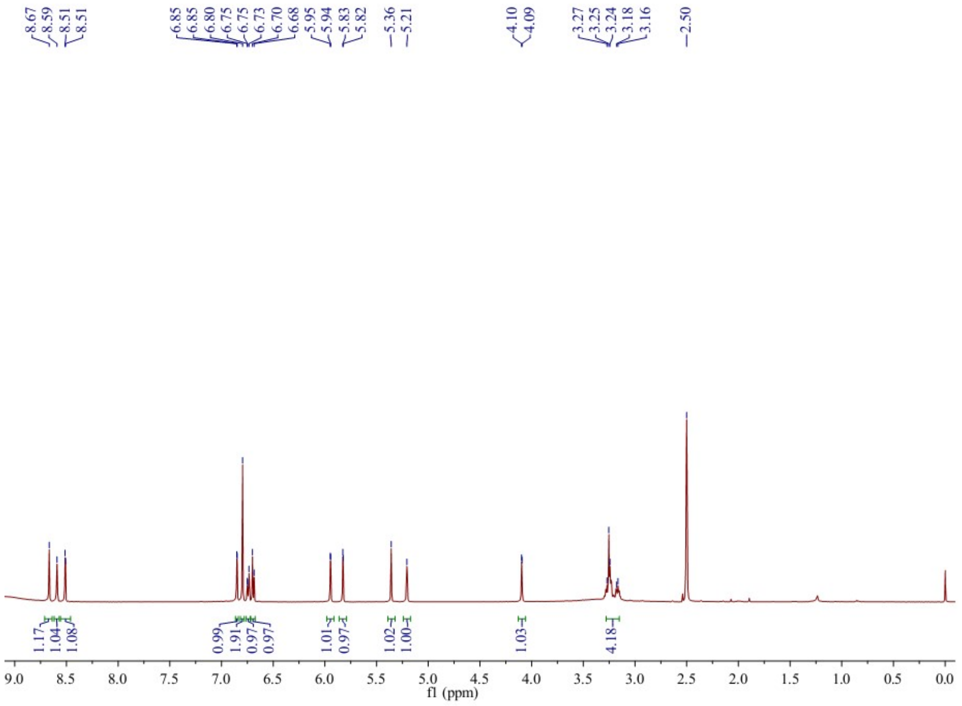

Figure S124. ^{1}H NMR of compound **JP-42** (DMSO-d_6)

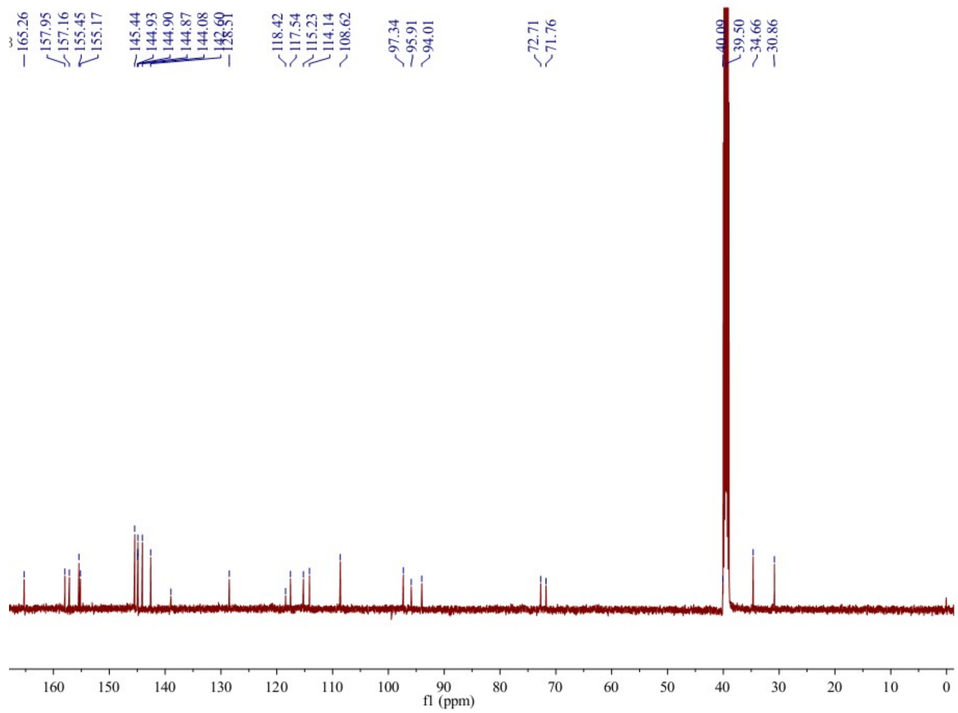

Figure S125. ^{13}C NMR of compound **JP-42** (DMSO-d_6)

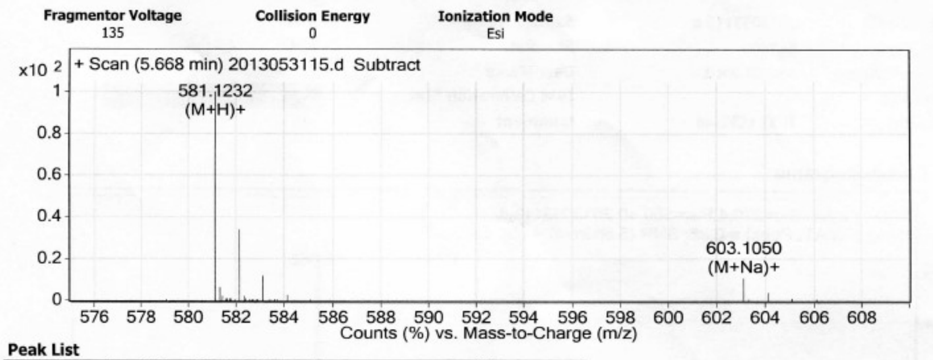

Peak List

m/z	z	Abund	Formula	Ion
246.1698		71512		
441.0832	1	877838		
442.0859	1	216250		
581.1232	1	1300136	C28 H25 N2 O10 S	(M+H)+
581.3251		82806		
582.127	1	442711	C28 H25 N2 O10 S	(M+H)+
583.1251	1	153610	C28 H25 N2 O10 S	(M+H)+
603.105		142601	C28 H24 N2 Na O10 S	(M+Na)+

Figure S126. HRESIMS of compound **JP-42**

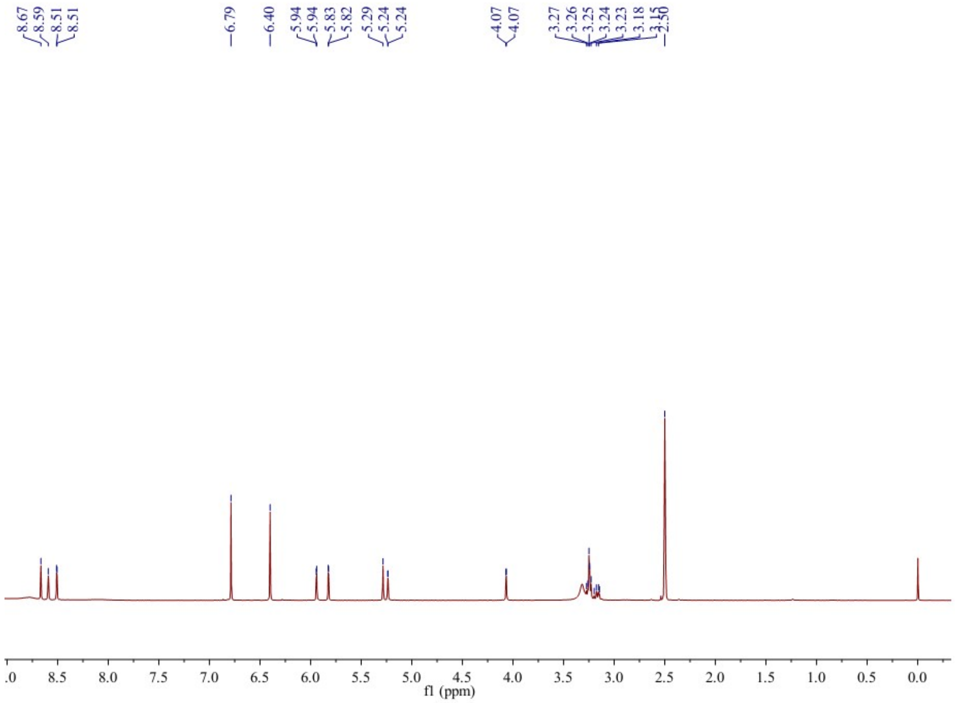

Figure S127. ^{1}H NMR of compound **JP-43** (DMSO-d_6)

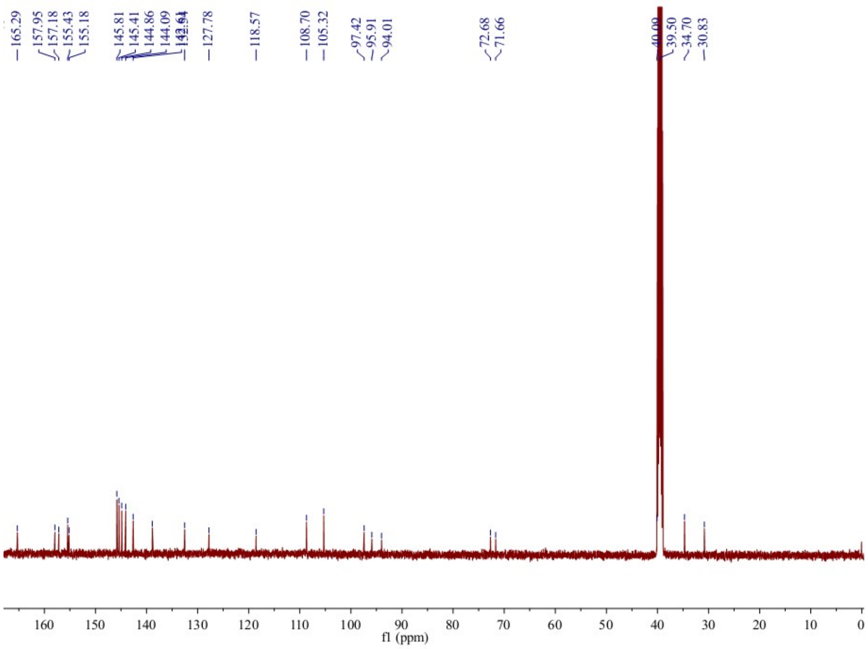

Figure S128. ^{13}C NMR of compound **JP-43** (DMSO-d_6)

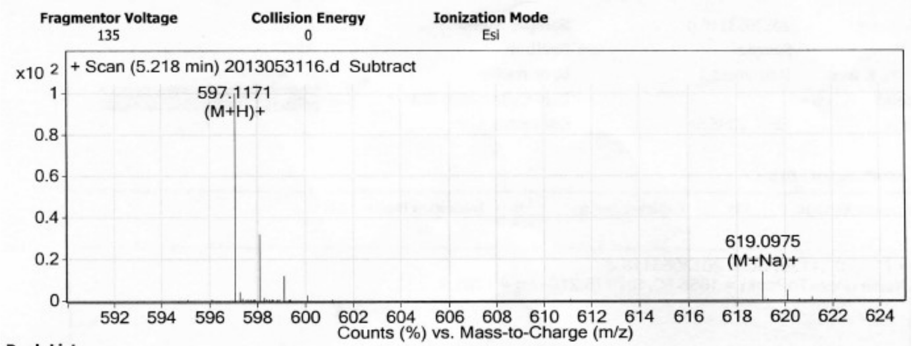

Peak List

m/z	z	Abund	Formula	Ion
123.116		81027		
169.1214		92080		
246.1691	1	177393		
251.1284		81443		
287.0544		129892		
457.076	1	206172		
597.1171	1	557082	C28 H25 N2 O11 S	(M+H)+
598.1198	1	177906	C28 H25 N2 O11 S	(M+H)+
599.1183	1	65547	C28 H25 N2 O11 S	(M+H)+
619.0975		74883	C28 H24 N2 Na O11 S	(M+Na)+

Figure S129. HRESIMS of compound **JP-43**

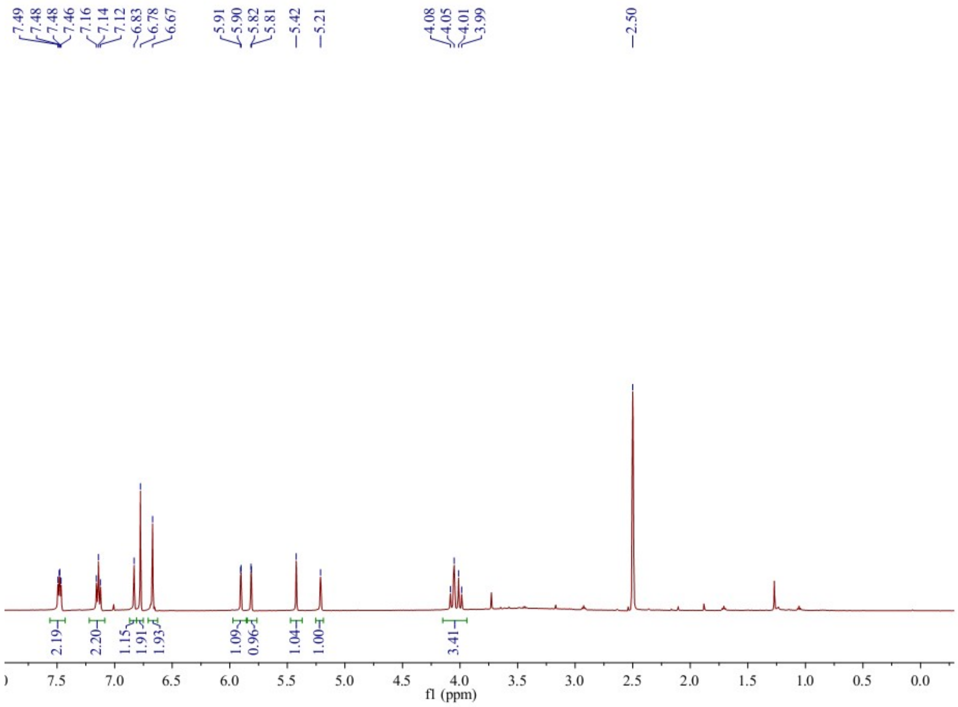

Figure S130. ^{1}H NMR of compound **JP-44** (DMSO-d_6)

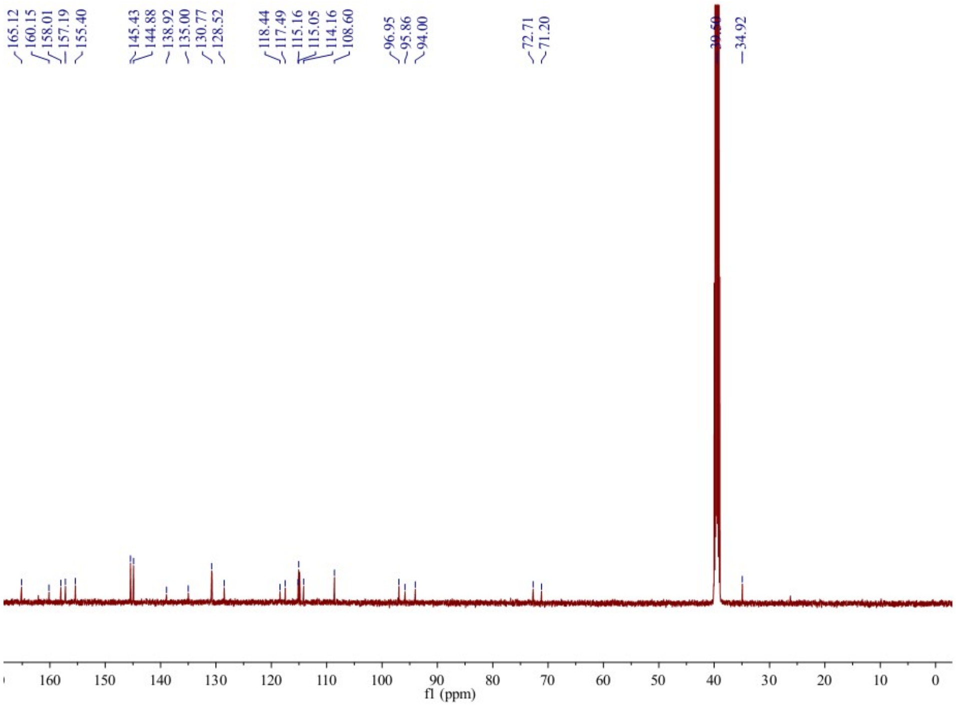

Figure S131. ^{13}C NMR of compound **JP-44** (DMSO-d_6)

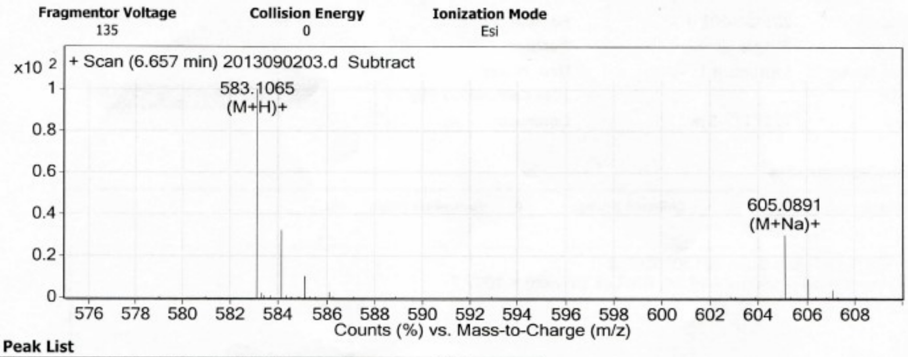

Peak List

m/z	z	Abund	Formula	Ion
110.0088		21769		
125.9862		31349		
214.1796		20624		
223.0984	1	37463		
245.0788		21052		
268.1878	1	33060		
583.1065	1	95691	C29 H24 F O10 S	(M+H)+
584.1099	1	31167	C29 H24 F O10 S	(M+H)+
605.0891	1	28934	C29 H23 F Na O10 S	(M+Na)+
922.0098	1	47877		

Figure S132. HRESIMS of compound **JP-44**

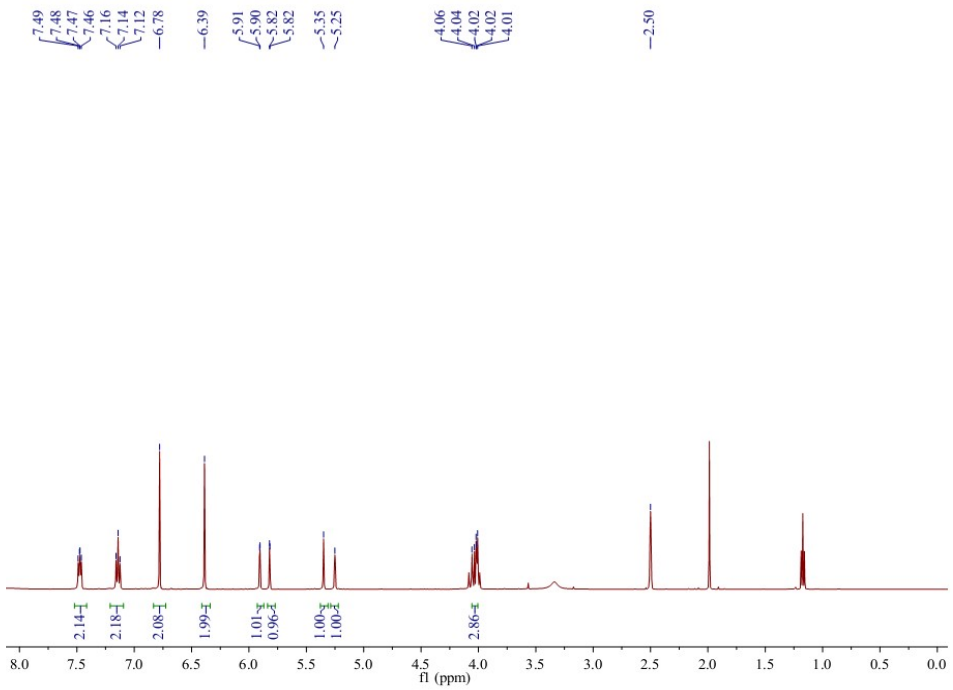

Figure S133. ^{1}H NMR of compound **JP-45** (DMSO-d_6)

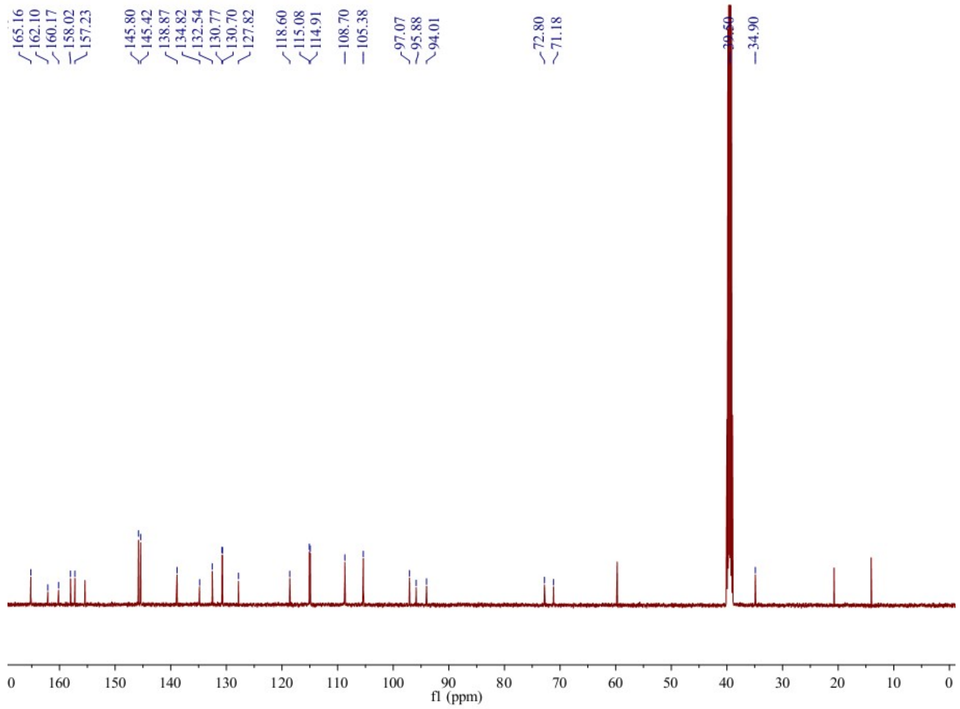

Figure S134. ^{1}H NMR of compound **JP-45** (DMSO-d_6)

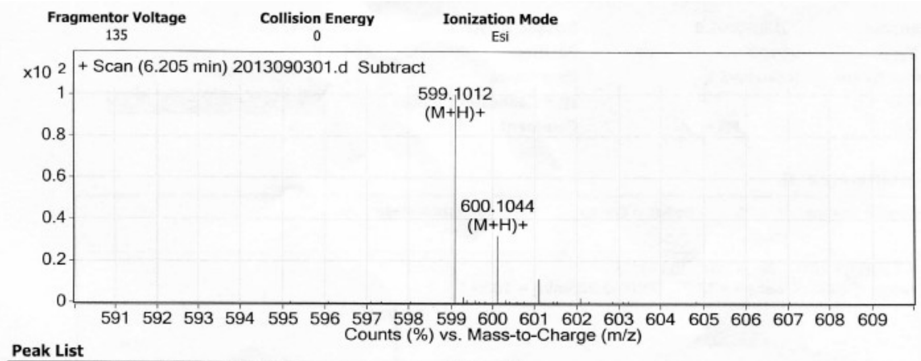

Peak List

m/z	z	Abund	Formula	Ion
110.0089		22774		
125.9863		24704		
142.0348		15298		
151.0352		20456		
216.1598		25031		
429.0807		19746		
599.1012	1	139631	C29 H24 F O11 S	(M+H)+
600.1044	1	44356	C29 H24 F O11 S	(M+H)+
601.1034	1	16198	C29 H24 F O11 S	(M+H)+
621.082		45774		

Figure S135. HRESIMS of compound **JP-45**

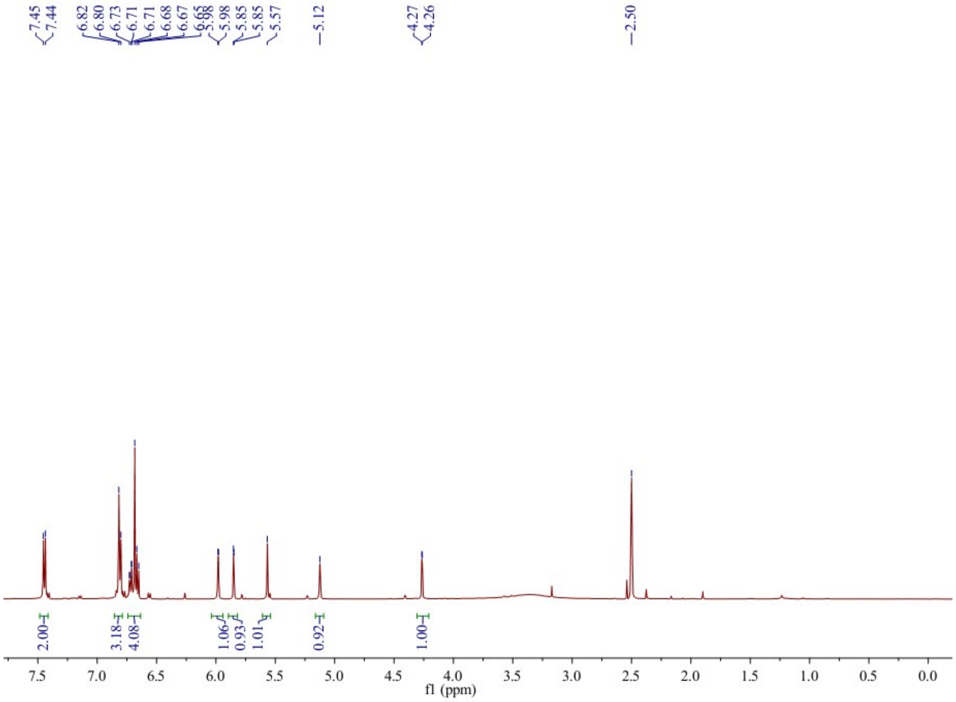

Figure S136. ^{1}H NMR of compound **JP-46** (DMSO-d_6)

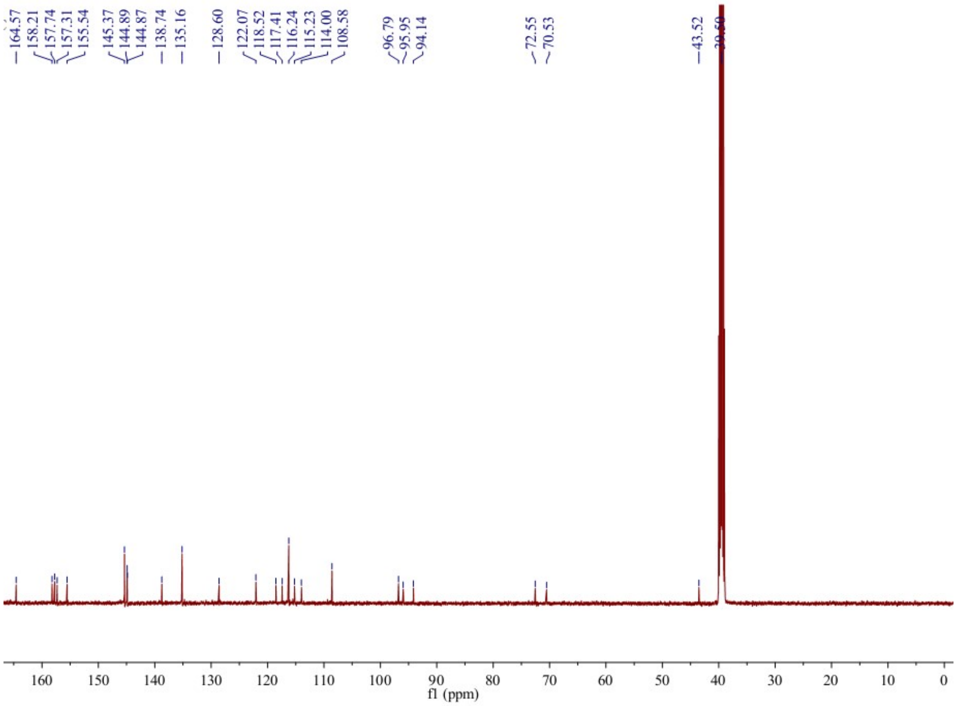

Figure S137. ^{13}C NMR of compound **JP-46** (DMSO-d_6)

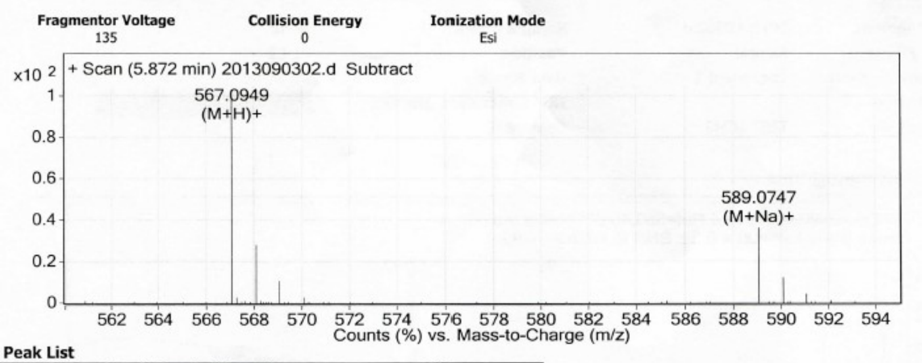

Peak List

m/z	z	Abund	Formula	Ion
110.0089		21092		
125.9862		29488		
211.0958		18663		
211.1663		17945		
251.1622	1	30476		
567.0949	1	76914	C28 H23 O11 S	(M+H)+
568.0977	1	21589	C28 H23 O11 S	(M+H)+
589.0747	1	28032	C28 H22 Na O11 S	(M+Na)+
922.0098	1	60628		
923.0115	1	17688		

Figure S138. HRESIMS of compound **JP-46**

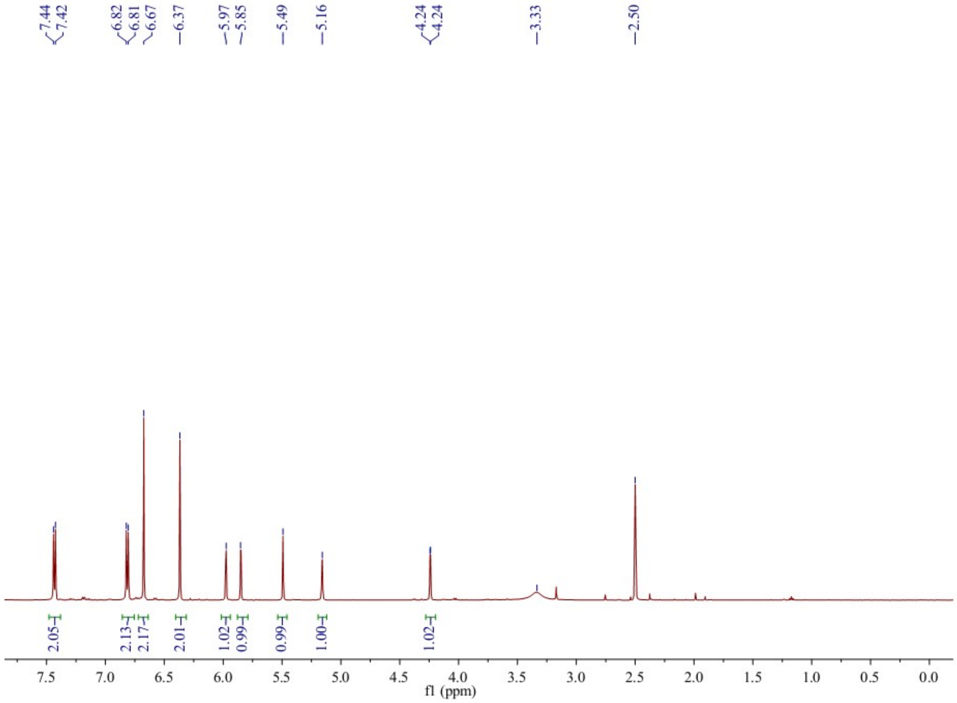

Figure S139. ^{1}H NMR of compound **JP-47** (DMSO-d_6)

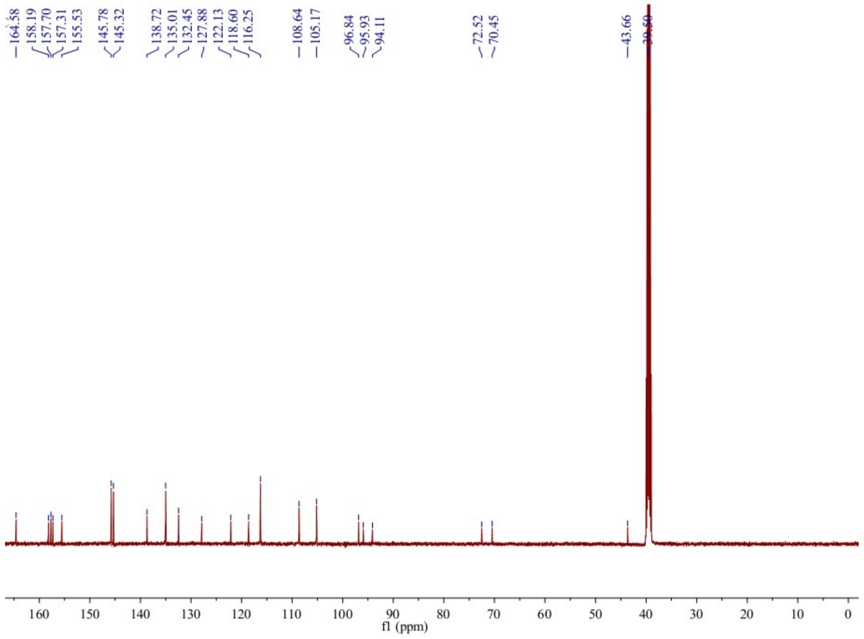

Figure S140. ^{13}C NMR of compound **JP-47** (DMSO-d_6)

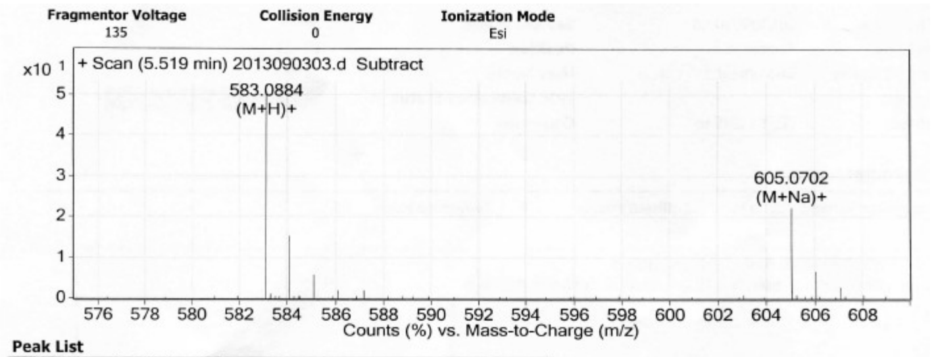

Peak List

m/z	z	Abund	Formula	Ion
110.0088	1	20152		
125.9863		27535		
142.0342	1	15132		
151.0351	1	20669		
151.107	1	14894		
167.0129		14421		
223.1301		18245		
583.0884	1	26228	C28 H23 O12 S	(M+H)+
605.0702	1	11550	C28 H22 Na O12 S	(M+Na)+
922.0098	1	51682		

Figure S141. HRESIMS of compound **JP-47**

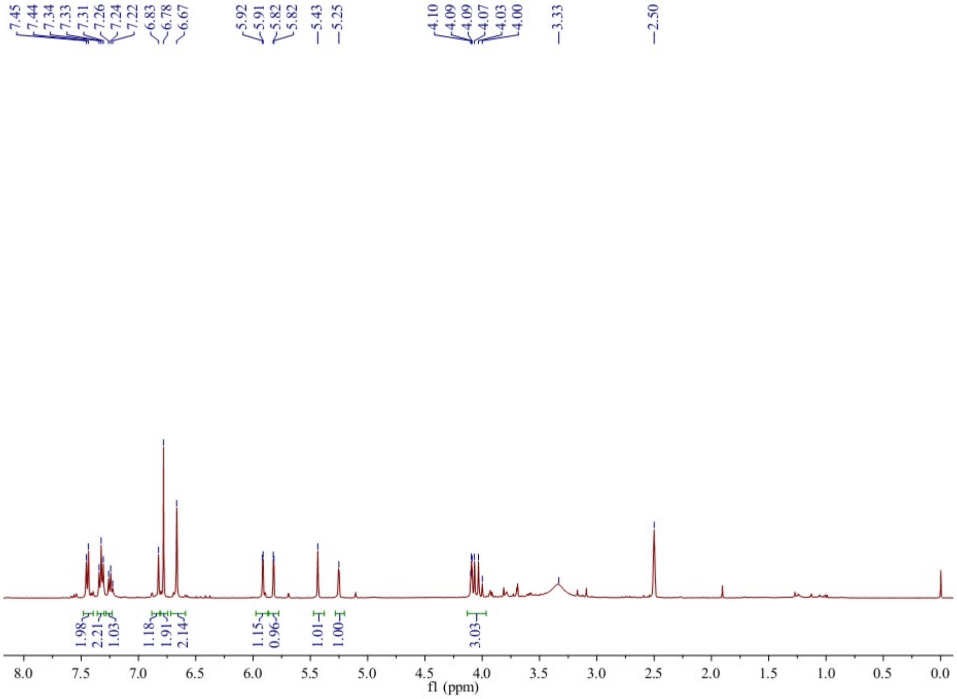

Figure S142. ^{1}H NMR of compound **JP-48** (DMSO-d_6)

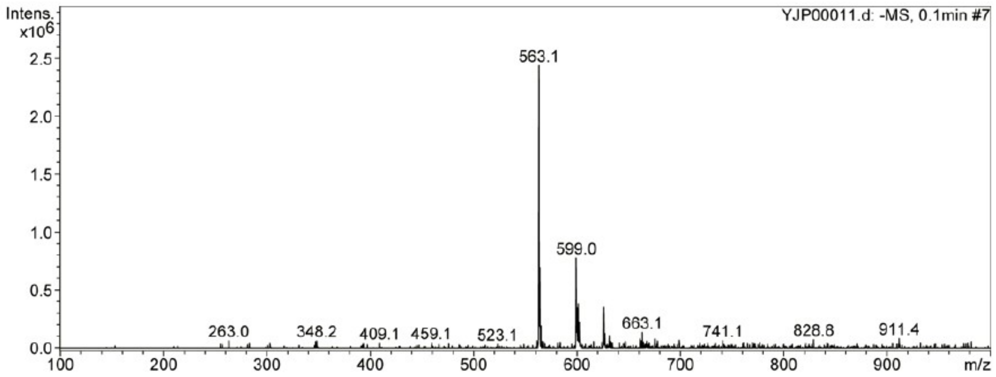

Figure S143. ESIMS of compound **JP-48**

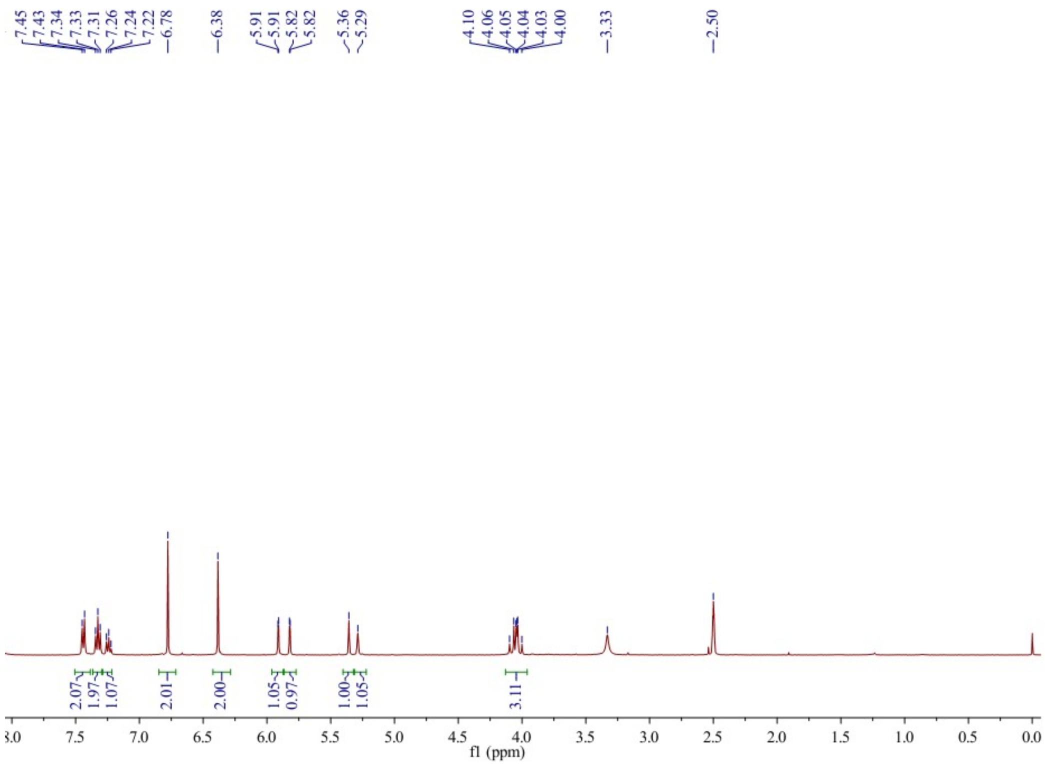

Figure S144. ^{1}H NMR of compound **JP-49** (DMSO-d_6)

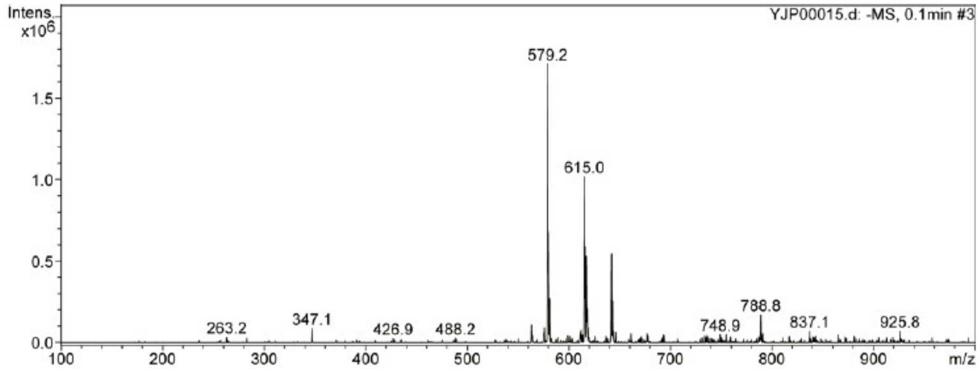

Figure S145. ESIMS of compound **JP-49**

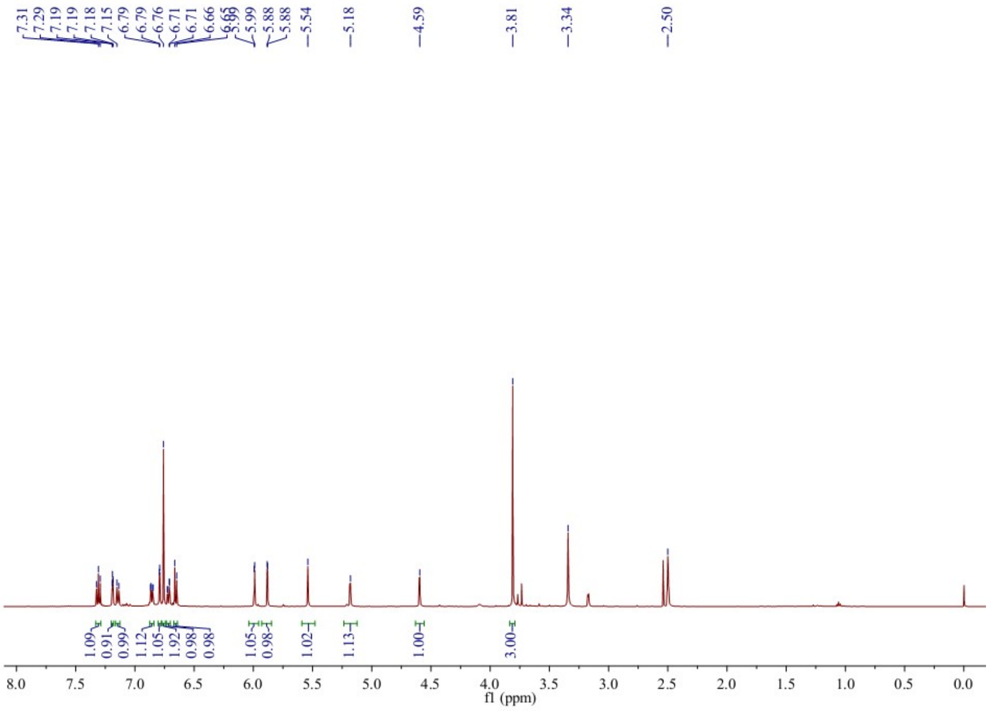

Figure S146. ^{1}H NMR of compound **JP-50** (DMSO-d_6)

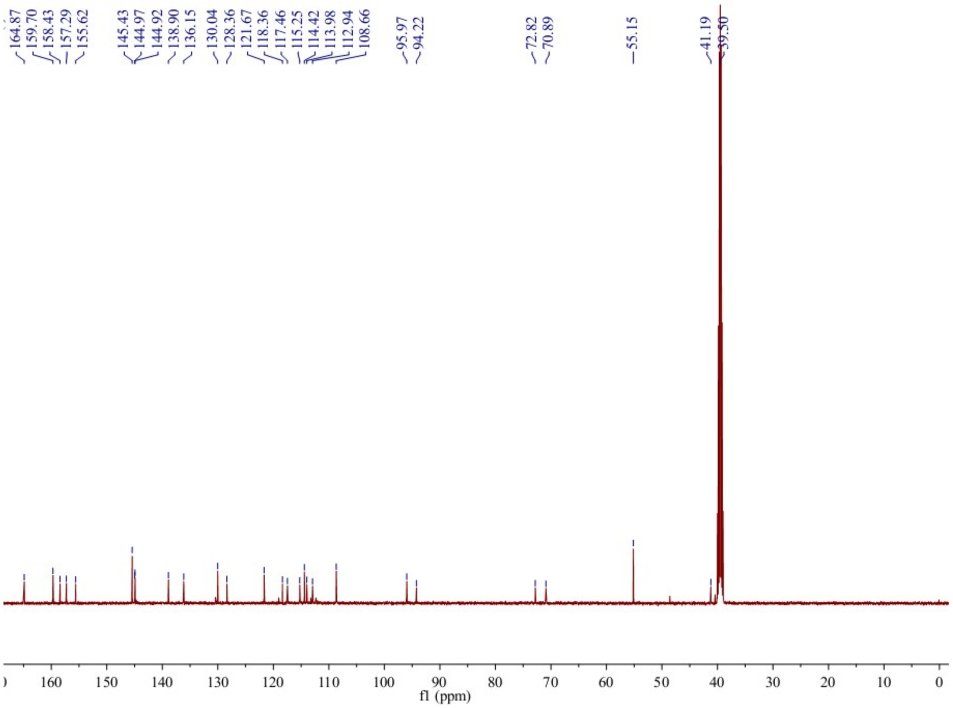

Figure S147. ^{13}C NMR of compound **JP-50** (DMSO-d_6)

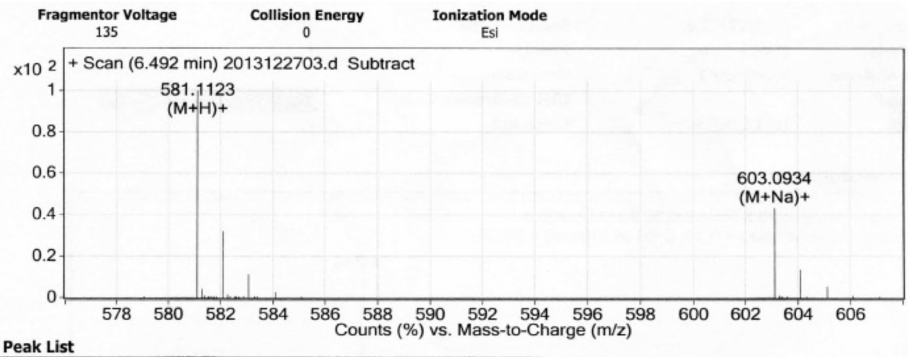

Peak List

m/z	z	Abund	Formula	Ion
271.0596		40241		
441.082	1	247986		
442.0849	1	60352		
581.1123	1	466764	C29 H25 O11 S	(M+H)+
582.1147	1	148939	C29 H25 O11 S	(M+H)+
583.1133	1	53250	C29 H25 O11 S	(M+H)+
603.0934	1	202980	C29 H24 Na O11 S	(M+Na)+
604.0968	1	64041	C29 H24 Na O11 S	(M+Na)+
1183.1977	1	54297		
1184.2001	1	36208		

Figure S148. HRESIMS of compound **JP-50**

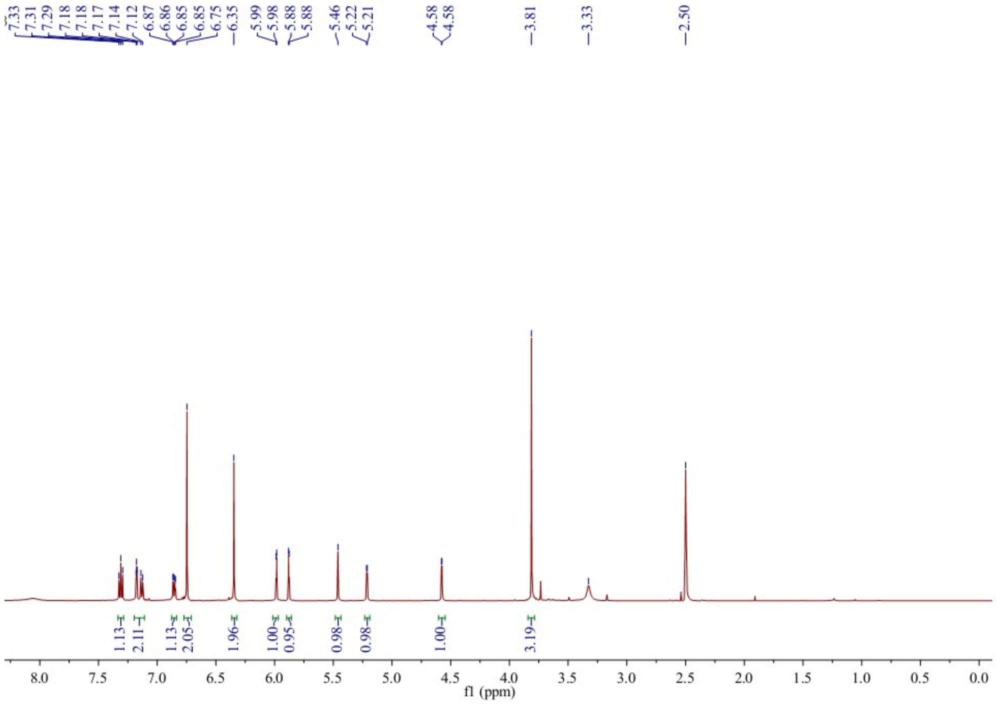

Figure S149. ^{1}H NMR of compound **JP-51** (DMSO-d_6)

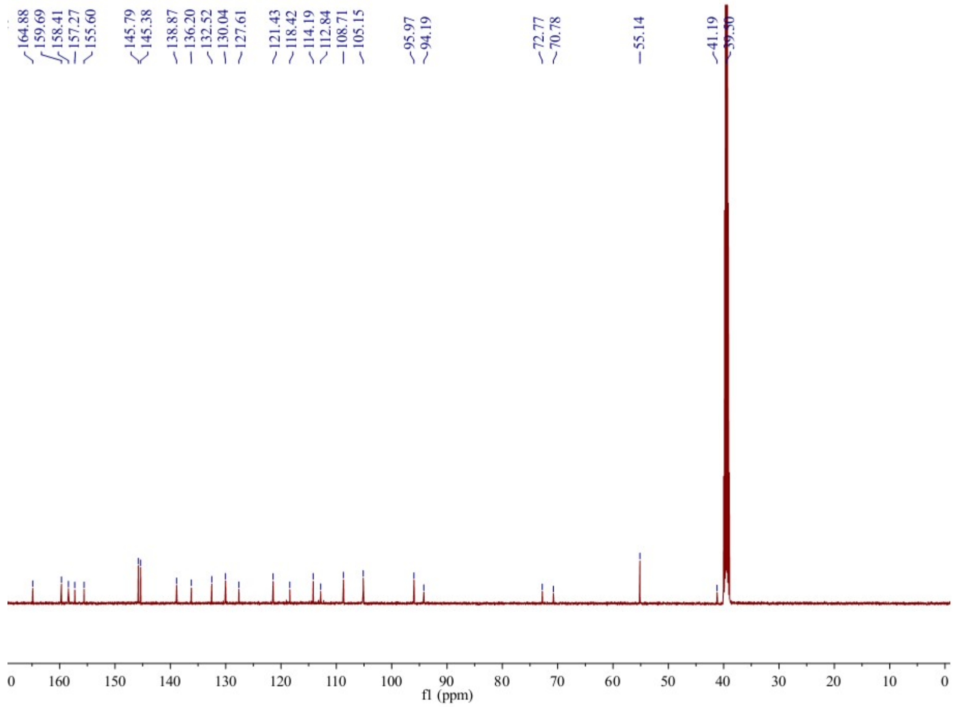

Figure S150. ^{13}C NMR of compound **JP-51** (DMSO-d_6)

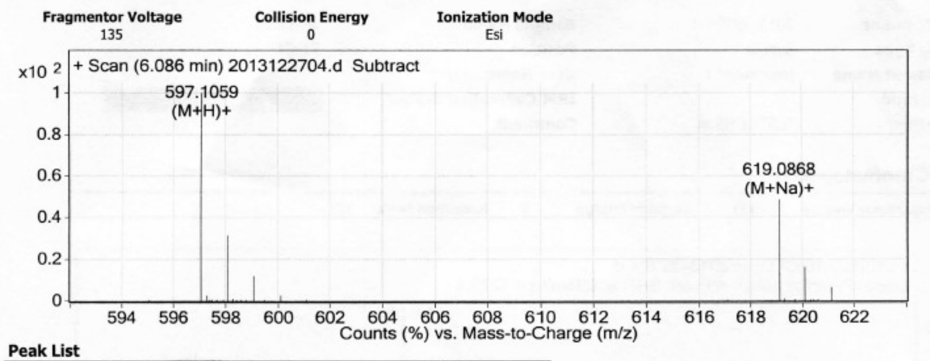

Peak List

m/z	z	Abund	Formula	Ion
187.1264		19489		
287.0545	1	58457		
299.1113		20354		
457.0765	1	46511		
458.0787	1	12835		
597.1059	1	170584	C29 H25 O12 S	(M+H)+
598.1093	1	53847	C29 H25 O12 S	(M+H)+
599.108	1	20157	C29 H25 O12 S	(M+H)+
619.0868	1	82886	C29 H24 Na O12 S	(M+Na)+
620.0906	1	28104	C29 H24 Na O12 S	(M+Na)+

Figure S151. HRESIMS of compound **JP-51**

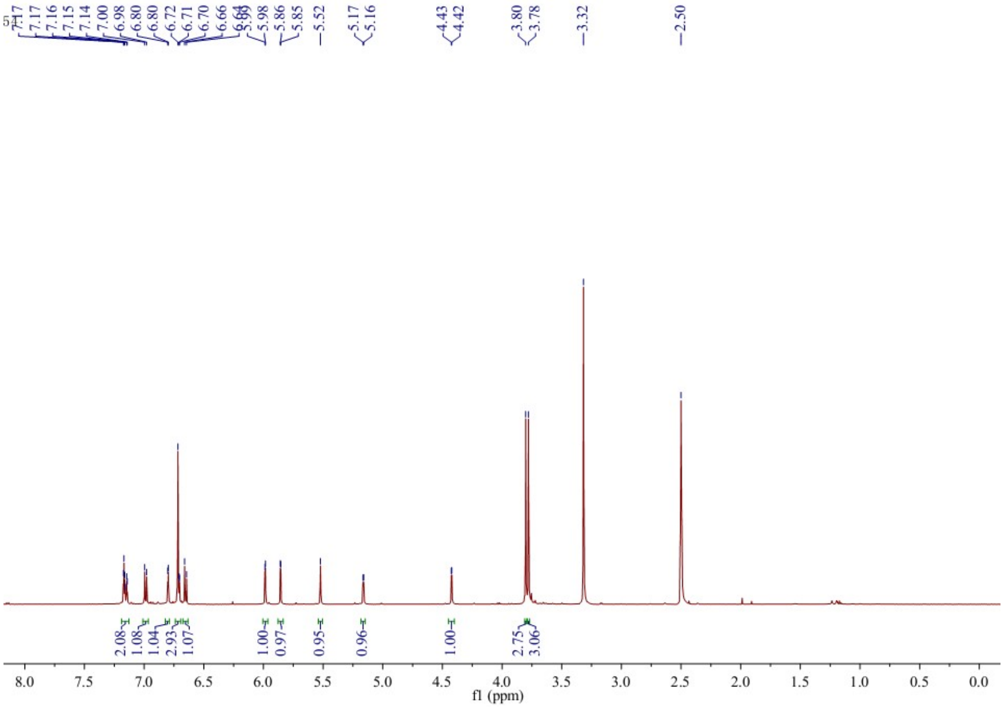

Figure S152. ^{1}H NMR of compound **JP-52** (DMSO-d_6)

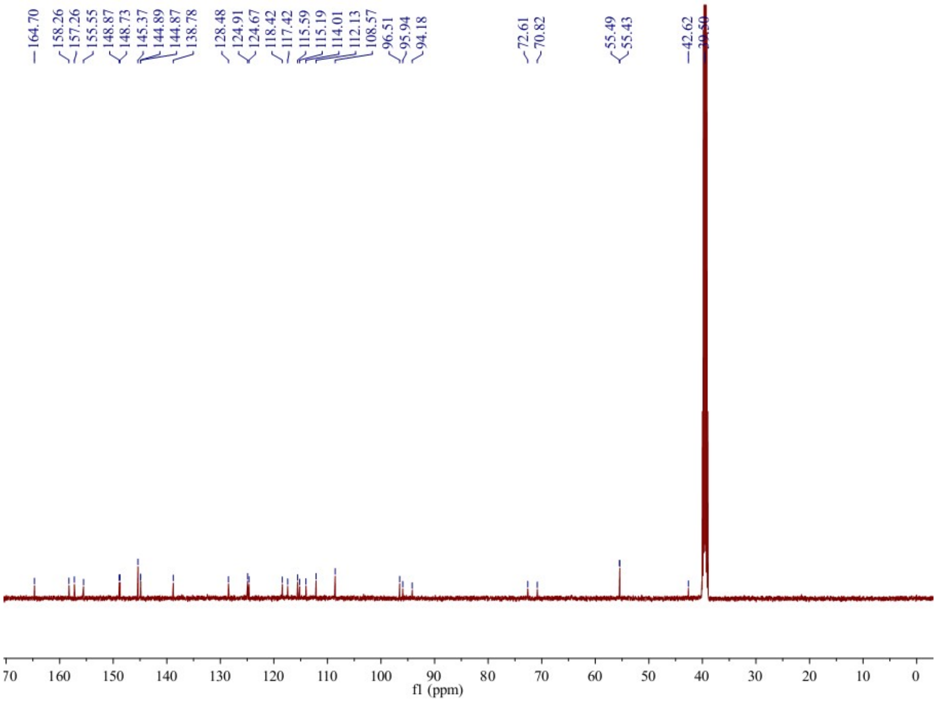

Figure S153. ^{13}C NMR of compound **JP-52** (DMSO-d_6)

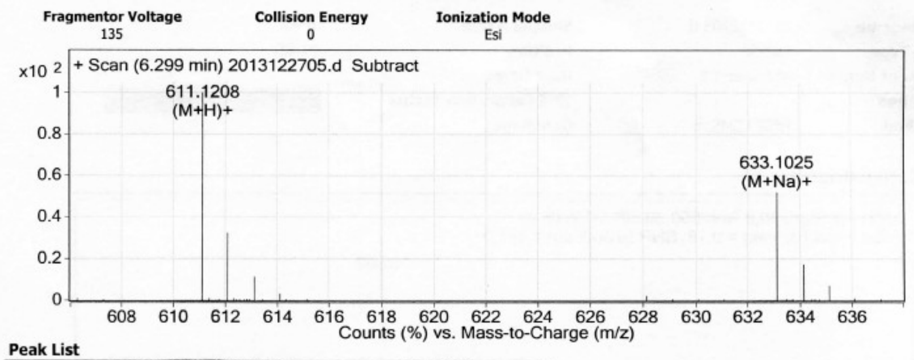

Peak List

m/z	z	Abund	Formula	Ion
271.0586		18249		
292.1546		27356		
314.1355	1	59185		
441.0821	1	133721		
442.0841	1	31368		
611.1208	1	179329	C30 H27 O12 S	(M+H)+
612.1247	1	58584	C30 H27 O12 S	(M+H)+
613.1215	1	20736	C30 H27 O12 S	(M+H)+
633.1025	1	93631	C30 H26 Na O12 S	(M+Na)+
634.1072	1	30823	C30 H26 Na O12 S	(M+Na)+

Figure S154. HRESIMS of compound **JP-52**

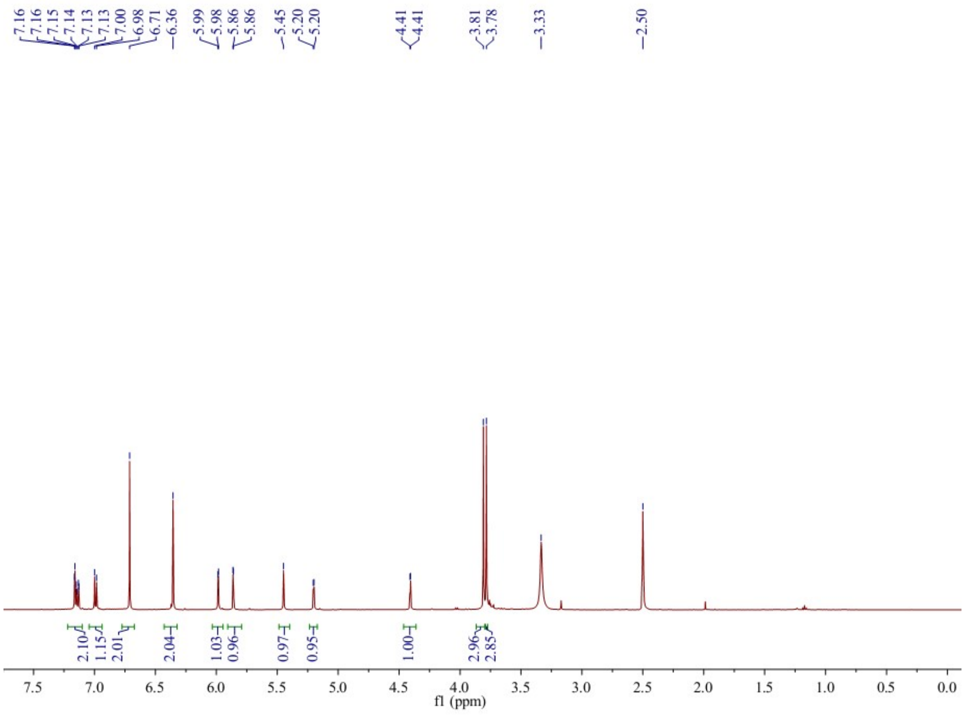

Figure S155. ^{1}H NMR of compound **JP-53** (DMSO-d_6)

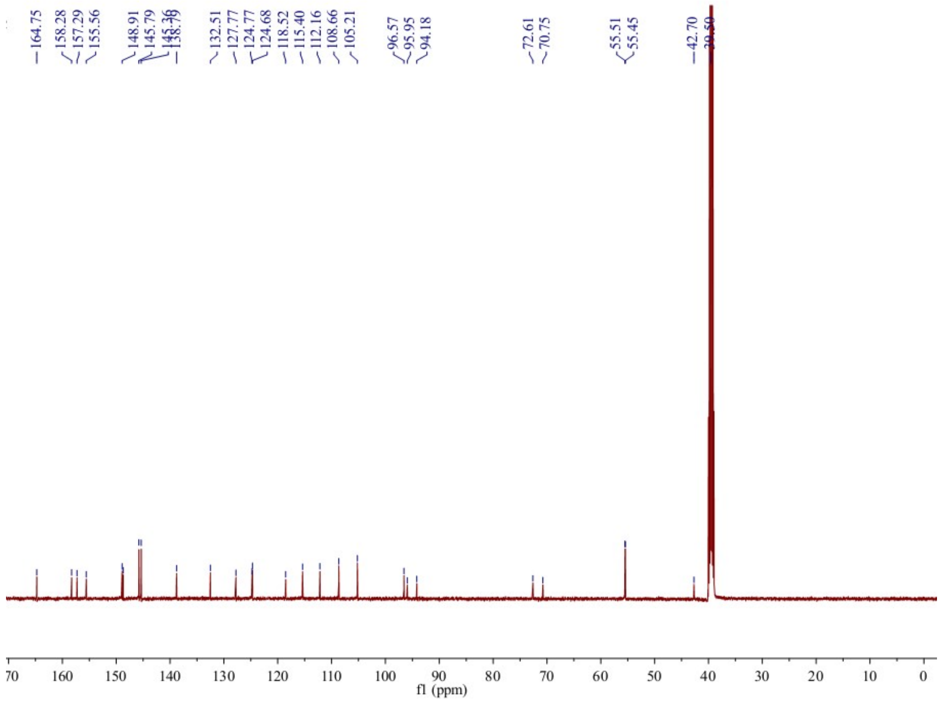

Figure S156. ^{13}C NMR of compound **JP-53** (DMSO-d_6)

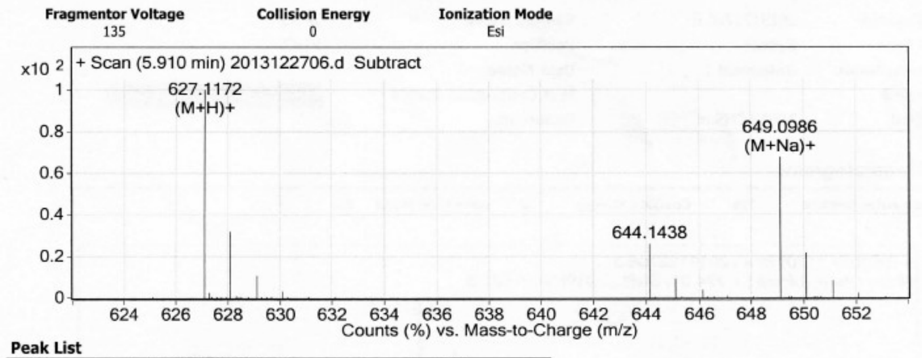

Peak List

m/z	z	Abund	Formula	Ion
187.126	1	74119		
287.055	1	52318		
457.0773	1	52089		
458.0807	1	12929		
627.1172	1	105399	C30 H27 O13 S	(M+H)+
628.1202	1	33419	C30 H27 O13 S	(M+H)+
629.1179	1	11597	C30 H27 O13 S	(M+H)+
644.1438	1	27380		
649.0986	1	71147	C30 H26 Na O13 S	(M+Na)+
650.1016	1	22785	C30 H26 Na O13 S	(M+Na)+

Figure S157. HRESIMS of compound **JP-53**

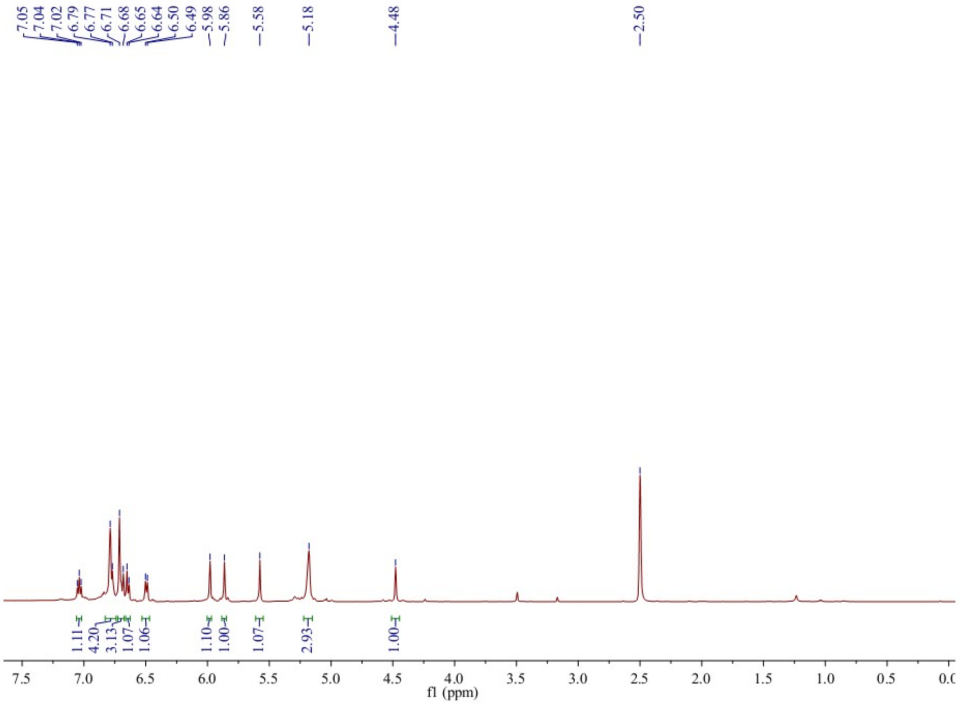

Figure S158. ^{1}H NMR of compound **JP-54** (DMSO-d_6)

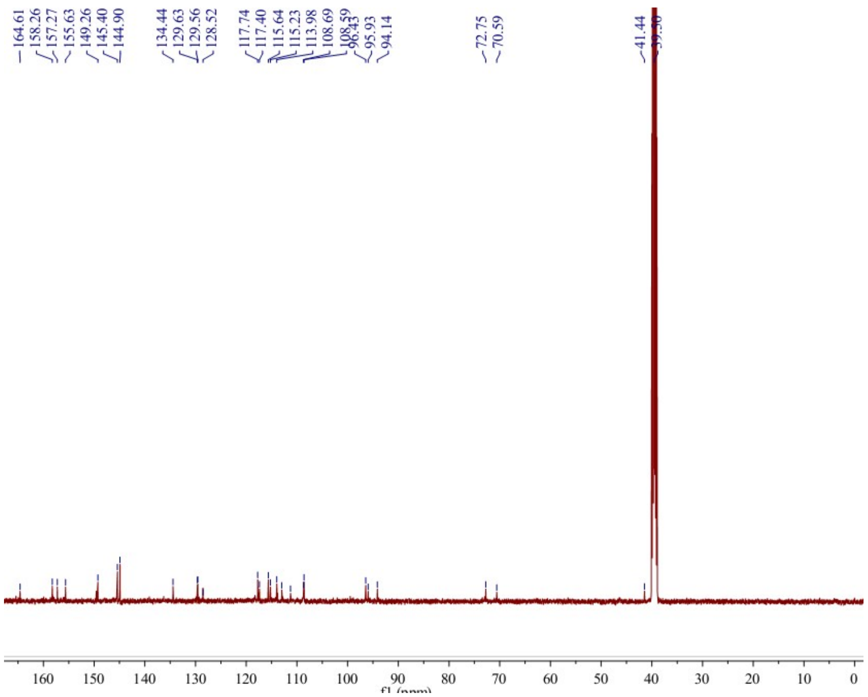

Figure S159. ^{13}C NMR of compound **JP-54** (DMSO-d_6)

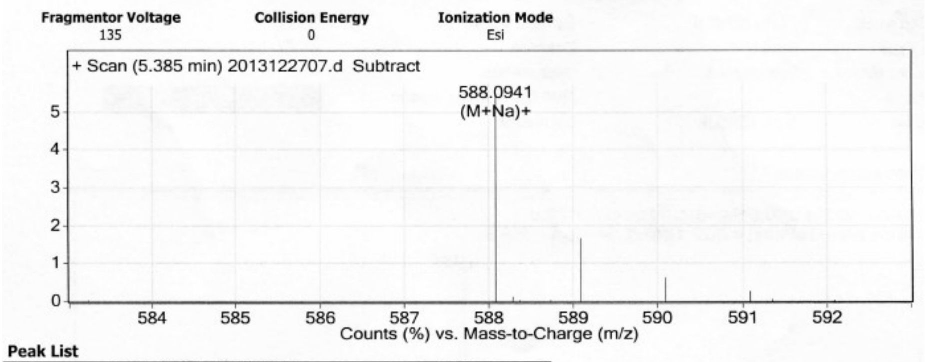

Peak List

m/z	z	Abund	Formula	Ion
247.0598		78802		
271.06		30573		
396.0901		68598		
441.0825	1	592284		
442.0852	1	139662		
475.3255		85196		
588.0941		32512	C28 H23 N Na O10 S	(M+Na)+

Figure S160. HRESIMS of compound **JP-54**

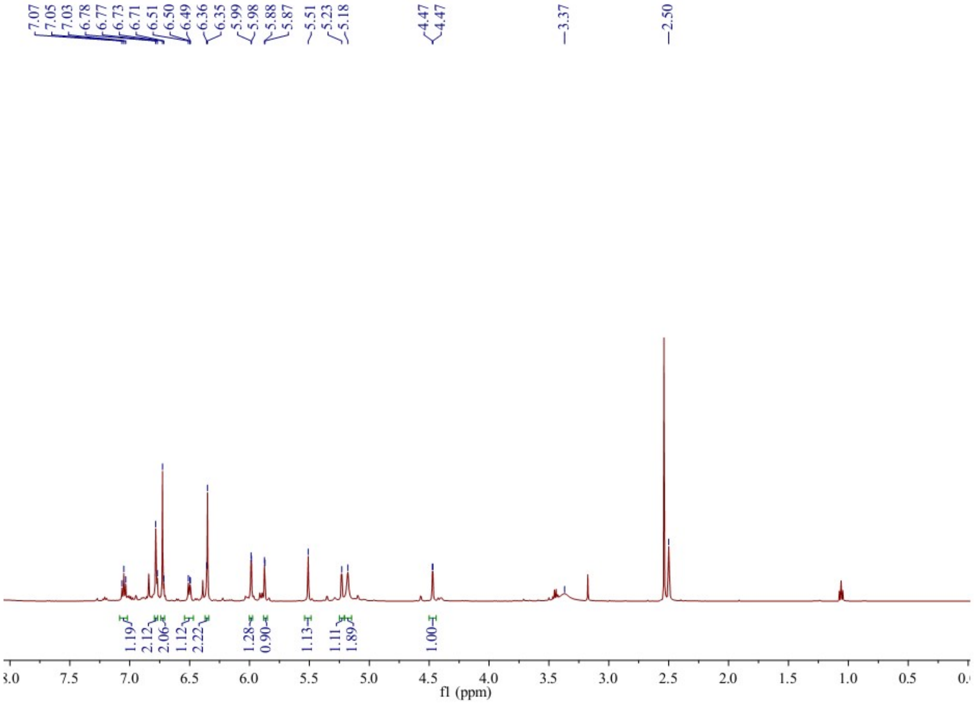

Figure S161. ^{1}H NMR of compound **JP-55** (DMSO-d_6)

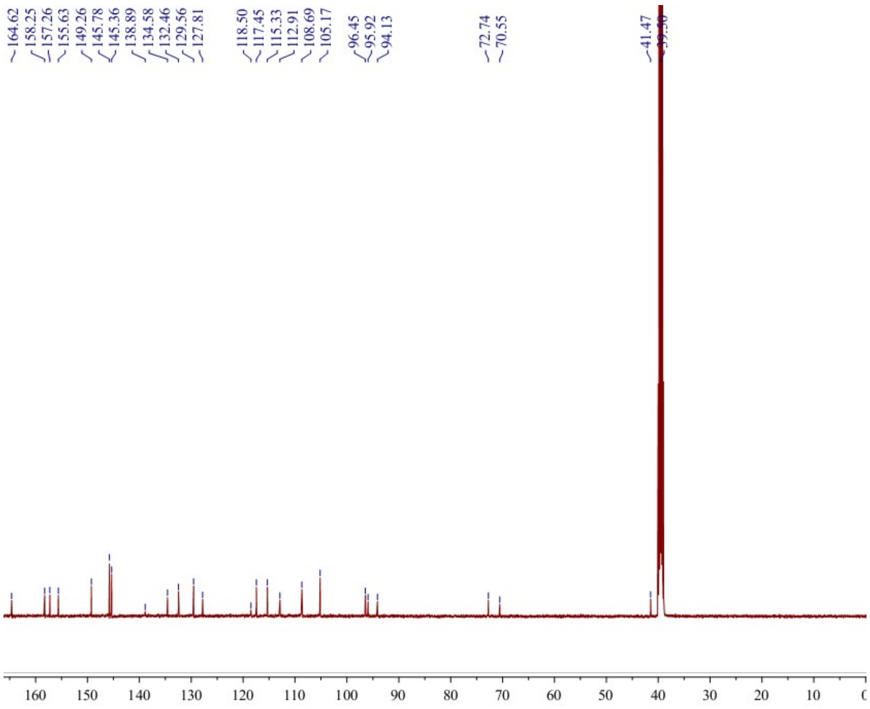

Figure S162. ^{13}C NMR of compound **JP-55** (DMSO-d_6)

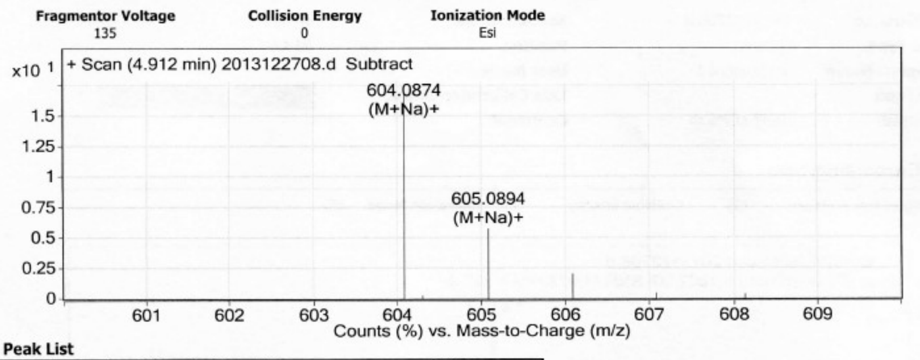

Peak List

m/z	z	Abund	Formula	Ion
263.0556		20687		
287.0539	1	87537		
288.0575	1	14660		
412.0834	1	20787		
413.0855	1	5799		
457.0749	1	87741		
458.0786	1	20742		
476.3058		15755		
481.2595		8617		
604.0874	1	14922	C28 H23 N Na O11 S	(M+Na)+

Figure S163. HRESIMS of compound **JP-55**

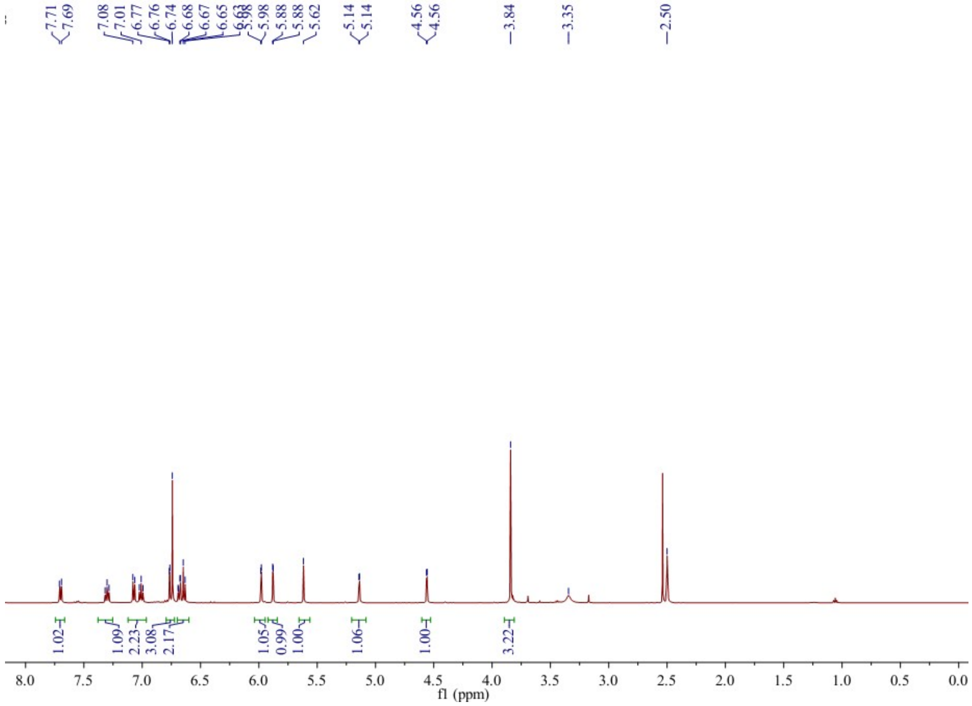

Figure S164. ^{1}H NMR of compound **JP-56** (DMSO-d_6)

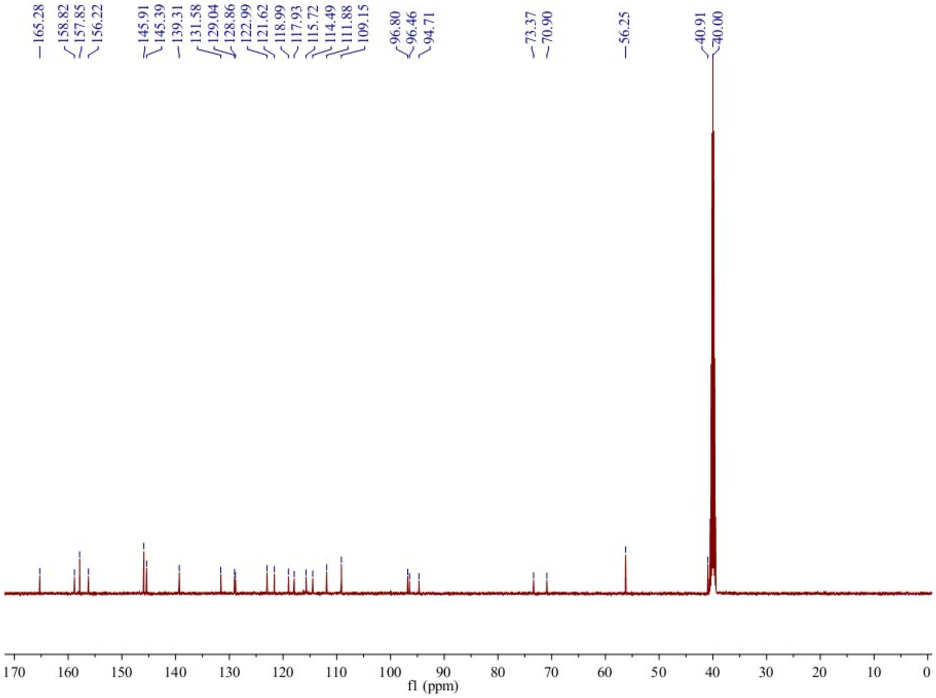

Figure S165. ^{13}C NMR of compound **JP-56** (DMSO-d_6)

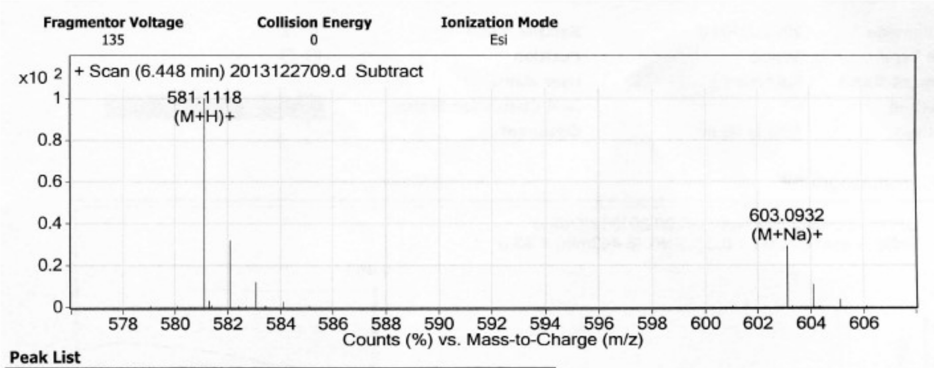

Peak List

m/z	z	Abund	Formula	Ion
271.0601		13964		
441.0815		57023		
581.1118	1	278740	C29 H25 O11 S	(M+H)+
582.1151	1	89147	C29 H25 O11 S	(M+H)+
583.1131	1	32679	C29 H25 O11 S	(M+H)+
603.0932	1	82178	C29 H24 Na O11 S	(M+Na)+
604.0953	1	29845	C29 H24 Na O11 S	(M+Na)+

Figure S166. HRESIMS of compound **JP-56**

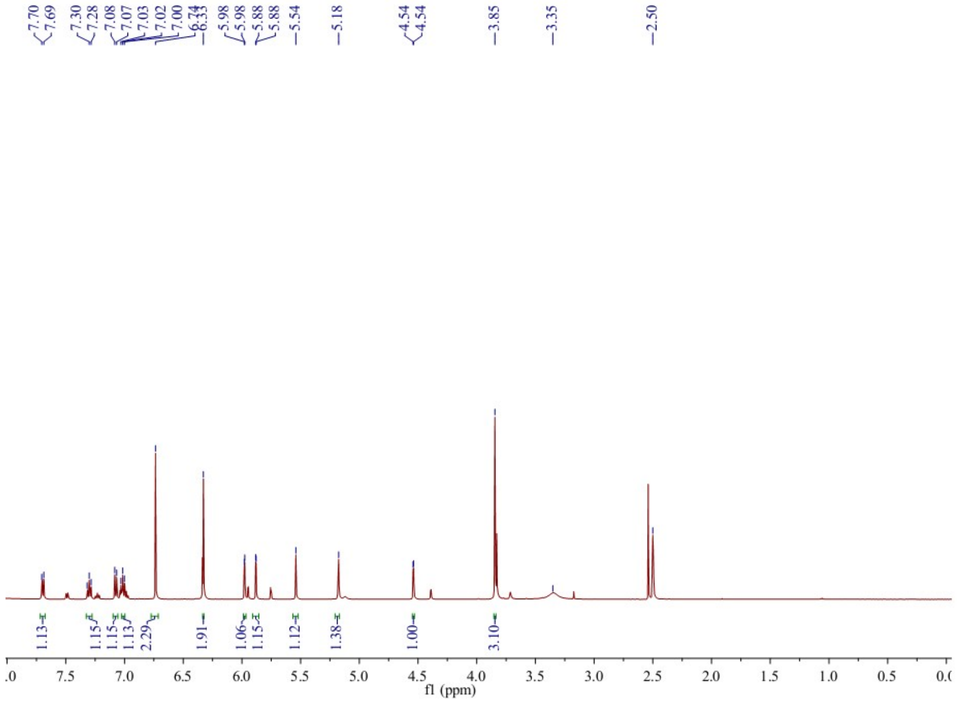

Figure S167. ^{1}H NMR of compound **JP-57** (DMSO-d_6)

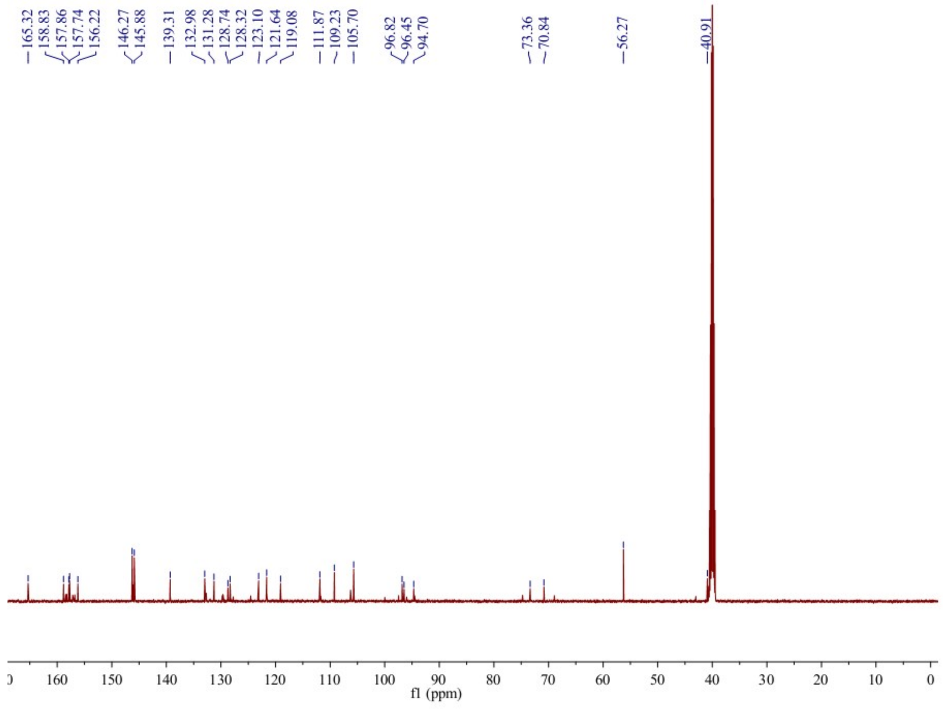

Figure S168. ^{13}C NMR of compound **JP-57** (DMSO-d_6)

Fragmentor Voltage	Collision Energy	Ionization Mode
135	0	Esi

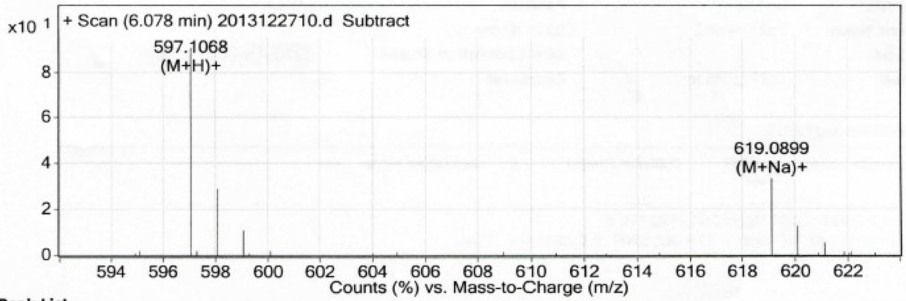

Peak List

m/z	z	Abund	Formula	Ion
116.9857		10014		
185.1518	1	7890		
187.1265	1	21198		
189.1123	1	9070		
207.1384	1	7411		
209.1139		7637		
211.0964	1	8904		
277.1292	1	9839		
299.1112	1	21081		
597.1068	1	19179	C29 H25 O12 S	(M+H)+

Figure S169. HRESIMS of compound **JP-57**

2. The reaction yield of compounds 1-57

No.	Name	Yield(%)
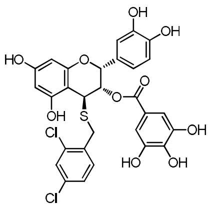 JP-1	4-(*S*)-(2,4-dichloro-benzylthio-yl)-Epicatechin gallate	32.1%
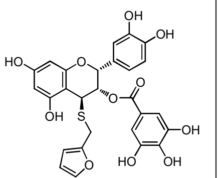 JP-2	4-(*S*)-(2-furfuryl-thio-yl)-Epicatechin gallate	33.2%
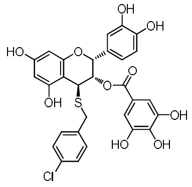 JP-3	4-(*S*)-(4-chloro-benzylthio-yl)-Epicatechin gallate	29.8%
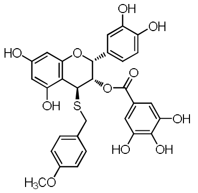 JP-4	4-(*S*)-(2-methoxyl-benzylthio-yl)-Epicatechin gallate	31.3%
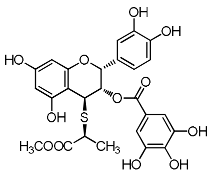 JP-5	4-(*S*)-(1-methoxy-1-oxo-2-(*S*)-propylthio-yl)-Epicatechin gallate	15.1%
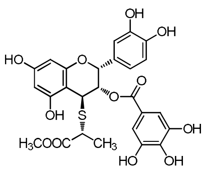 JP-6	4-(*S*)-(1-methoxy-1-oxo-2-(*R*)-propylthio-yl)-Epicatechin gallate	13.4%

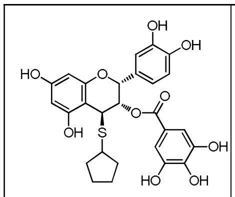 JP-7	4-(*S*)-(cyclo-pentylthio-yl)-Epicatechin gallate	15.7%
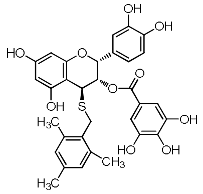 JP-8	4-(*S*)-(2,4,6-trimethyl-benzylthio-yl)-Epicatechin gallate	29.8%
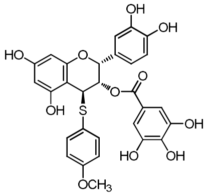 JP-9	4-(*S*)-(4-methoxy-phenylthio-yl)-Epicatechin gallate	25.7%
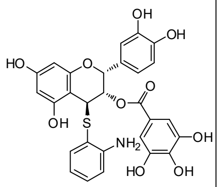 JP-10	4-(*S*)-(2-amino-phenylthio-yl)-<br>Epicatechin gallate	27.8%
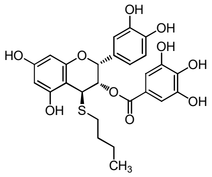 JP-11	4-(*S*)-(1-butylthio-yl)-Epicatechin gallate	15.7%
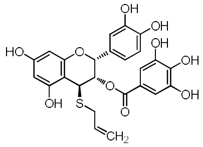 JP-12	4-(*S*)-(1-allylthio-yl)-Epicatechin gallate	17.6%
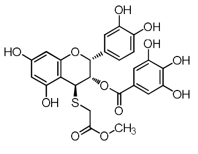 JP-13	4-(*S*)-(2-methoxy-2-oxo-ethylthio-yl)-Epicatechin gallate	27.9%

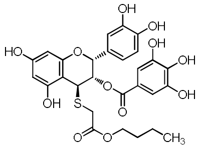 JP-14	4-(*S*)-(2-butoxy-2-oxo-ethylthio-yl)-Epicatechin gallate	29.7%
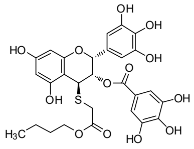 JP-15	4-(*S*)-(2-butoxy-2-oxo-ethylthio)-Epigallocatechin gallate	28.5%
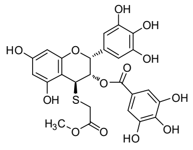 JP-16	4-(*S*)-(2-methoxy-2-oxo-ethylthio-yl)-Epigallocatechin gallate	27.9%
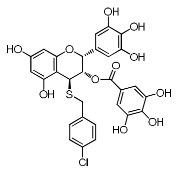 JP-17	4-(*S*)-(4-chloro-benzylthio-yl)-Epigallocatechin gallate	30.9%
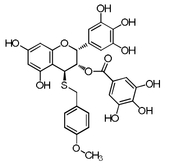 JP-18	4-(*S*)-(4-methoxy-benzylthio-yl)-Epigallocatechin gallate	29.8%
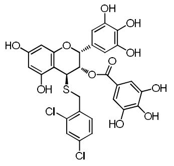 JP-19	4-(*S*)-(2,4-dichloro-benzylthio-yl)-Epigallocatechin gallate	30.8%
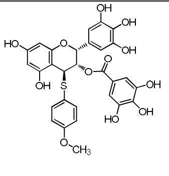 JP-20	4-(*S*)-(4-methoxy-phenylthio-yl)-Epigallocatechin gallate	29.7%

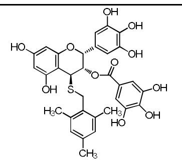 JP-21	4-(*S*)-(2,4,6-trimethyl-benzylthio-yl)-Epigallocatechin gallate	27.8%
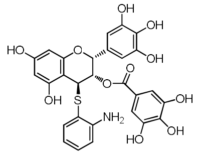 JP-22	4-(*S*)-(2-amino-phenylthio-yl)-Epicatechin gallate	21.3%
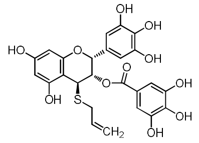 JP-23	4-(*S*)-(1-allylthio-yl)-Epigallocatechin gallate	13.6%
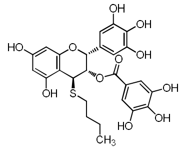 JP-24	4-(*S*)-(1-butylthio-yl)-Epigallocatechin gallate	11.3%
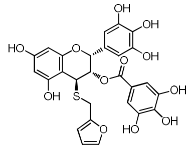 JP-25	4-(*S*)-(2-furfuryl-thio-yl)-Epigallocatechin gallate	29.7%
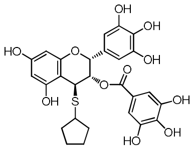 JP-26	4-(*S*)-(cyclo-pentylthio-yl)-Epigallocatechin gallate	17.6%
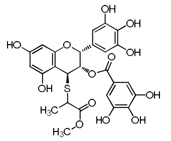 JP-27	4-(*S*)-(1-methoxy-1-oxo-2-propylthio-yl)-Epigallocatechin gallate	27.3%
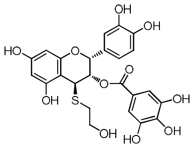 JP-28	4-(*S*)-(2-ethylthio-yl)-Epicatechin gallate	29.8%

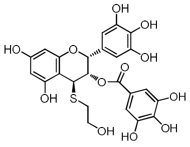 JP-29	4-(*S*)-(2-ethylthio-yl)-Epigallocatechin gallate	30.1%
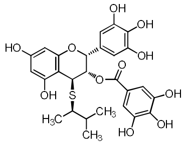 JP-30	4-(*S*)-(3-methyl-2-(*R*)-butylthio-yl)-Epigallocatechin gallate	11.3%
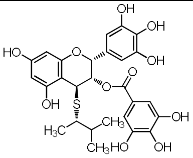 JP-31	4-(*S*)-(3-methyl-2-(*S*)-butylthio-yl)-Epigallocatechin gallate	9.1%
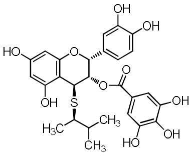 JP-32	4-(*S*)-(3-methyl-2-(*R*)-butylthio-yl)-Epicatechin gallate	9.8%
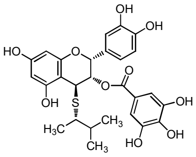 JP-33	4-(*S*)-(3-methyl-2-(*S*)-butylthio-yl)-Epicatechin gallate	11.7%
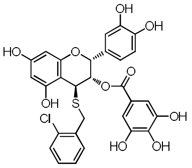 JP-34	4-(*S*)-(2-chloro-benzylthio-yl)-Epicatechin gallate	31.5%
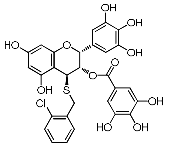 JP-35	4-(*S*)-(2-chloro-benzylthio-yl)-Epigallocatechin gallate	29.8%

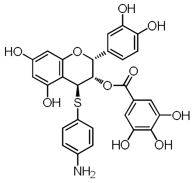<br>JP-36	4-(*S*)-(4-amino-phenylthio-yl)-Epicatechin gallate	27.9%
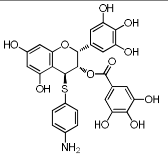<br>JP-37	4-(*S*)-(4-amino-phenylthio-yl)-Epigallocatechin gallate	28.7%
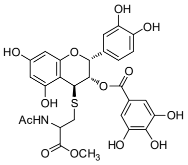<br>JP-38	4-(*S*)-(2-(*S*)-acetylamino-3-methoxy-3-oxo-propylthio-yl)-Epicatechin gallate	28.6%
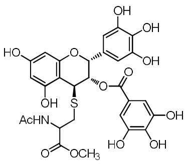<br>JP-39	4-(*S*)-(2-(*S*)-acetylamino-3-methoxy-3-oxo-propylthio-yl)-Epigallocatechin gallate	23.6%
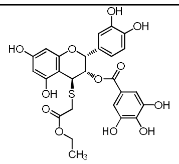<br>JP-40	4-(*S*)-(2-ethoxy-2-oxo-ethylthio-yl)-Epicatechin gallate	27.6%
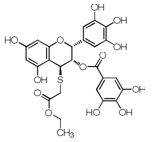<br>JP-41	4-(*S*)-(2-ethoxy-2-oxo-ethylthio-yl)-Epigallocatechin gallate	27.9%
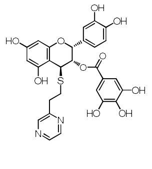<br>JP-42	4-(*S*)-(2-pyrazinyl-ethylthio-yl)-Epicatechin gallate	19.8%

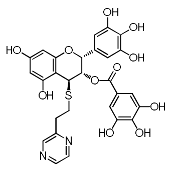 JP-43	4-(*S*)-(2-pyrazinyl-ethylthio-yl)-Epigallocatechin gallate	11.4%
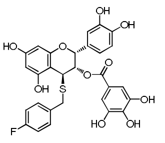 JP-44	4-(*S*)-(4-fluoro-benzylthio-yl)-Epicatechin gallate	27.9%
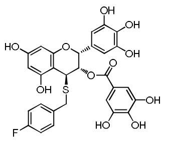 JP-45	4-(*S*)-(4-fluoro-benzylthio-yl)-Epigallocatechin gallate	28.3%
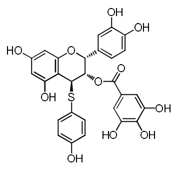 JP-46	4-(*S*)-(4-hydroxy-phenylthio-yl)-Epicatechin gallate	13.4%
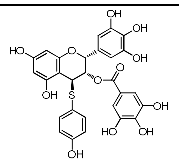 JP-47	4-(*S*)-(4-hydroxy-phenylthio-yl)-Epigallocatechin gallate	16.7%
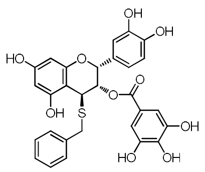 JP-48	4-(*S*)-(benzylthio-yl)-Epigallocatechin gallate	33.2%
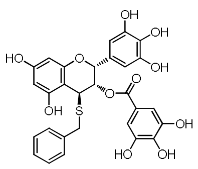 JP-49	4-(*S*)-(benzylthio-yl)-Epicatechin gallate	30.1%

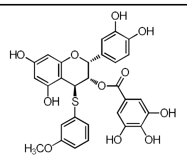 JP-50	4-(*S*)-(3-methoxy-phenylthio-yl)-Epicatechin gallate	23.5%
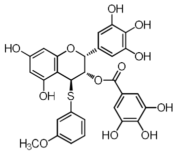 JP-51	4-(*S*)-(3-methoxy-phenylthio-yl)-Epigallocatechin gallate	26.7%
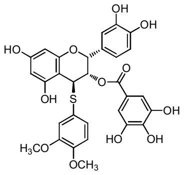 JP-52	4-(*S*)-(3,4-dimethoxy-phenylthio-yl)-Epicatechin gallate	28.6%
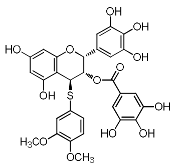 JP-53	4-(*S*)-(3,4-dimethoxy-phenylthio-yl)-Epigallocatechin gallate	27.1%
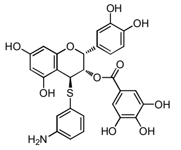 JP-54	4-(*S*)-(3-amino-phenylthio-yl)-Epicatechin gallate	19.8%
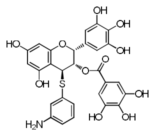 JP-55	4-(*S*)-(3-amino-phenylthio-yl)-Epigallocatechin gallate	20.1%
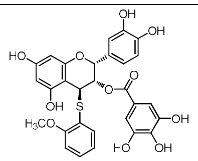 JP-56	4-(*S*)-(2-methoxy-phenylthio-yl)-Epicatechin gallate	29.8%

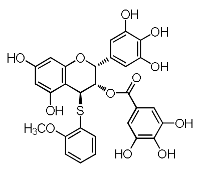 JP-57	4-(*S*)-(2-methoxy-phenylthio-yl)-Epigallocatechin gallate	30.1%

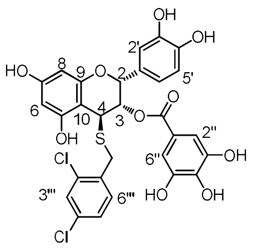

JP-1: UV λ_{max} (MeOH) nm: 278, 216; mp. 146.2~148.7°C; $[\alpha]_D^{25}$ -87.9° (*c* 0.1 MeOH); HR-ESI-MS (*m/z* 633.0386 $[M+H]^+$, calcd, 633.0383); ^{1}H NMR (500 MHz, DMSO-d_6) δ: 7.65 (1H, s, 3′′′-H), 7.62 (1H, dd, *J* = 8.0, 1.5, 5′′′-H), 7.41 (1H, d, *J* = 8.0, 6′′′-H), 6.87 (1H, d, *J* = 1.5, 2′-H), 6.82 (2H, s, 2′′, 6′′-H), 6.72 (1H, dd, *J* = 8.0, 1.5, 6′-H), 6.68 (1H, d, *J* = 8.0, 5′-H), 5.91 (1H, d, *J* = 2.0, 8-H), 5.83 (1H, d, *J* = 2.0, 6-H), 5.44 (1H, s, 3-H), 5.27 (1H, s, 2-H), 4.12 (1H, s, 4-H), 4.10 (2H, s, S-C-H); ^{13}C NMR (125 MHz, DMSO-d_6) δ: 165.2 (O-C-O), 158.1 (C-5), 157.2 (C-7), 155.4 (C-9), 145.4 (C-3′′, 5′′), 144.9 (C-3′, 4′), 138.9 (C-4′′), 135.4 (C-2′′′), 134.1 (C-4′′′), 132.4 (C-6′′′), 132.2 (C-1′), 128.9 (C-3′′′), 127.3 (C-1′′′, 5′′′), 118.4 (C-1′′), 117.5 (C-6′), 115.2 (C-5′), 114.2 (C-2′), 108.6 (C-2′′, 6′′), 96.6 (C-10), 95.9 (C-6), 94.0 (C-8), 72.8 (C-2), 71.3 (C-3), 40.0 (C-4), 33.1 (S-C).

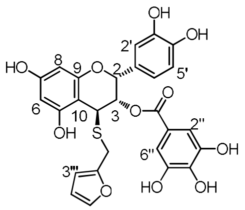

JP-2: UV λ_{max} (MeOH) nm: 278, 216; mp. 157.5~160.1°C; $[\alpha]_D^{25}$ -131.5° (*c* 0.1 MeOH); HR-ESI-MS (*m/z* 555.0966 $[M+H]^+$, calcd, 555.0956); ^{1}H NMR (500 MHz, DMSO-d_6) δ: 7.62 (1H, dd, *J* = 8.0, 1.5, 5′′′-H), 6.85 (1H, d, *J* = 1.5, 2′-H), 6.79 (2H,

s, 2″, 6″-H), 6.73 (2H, m, 5′, 6′-H), 6.41 (1H, d, *J* = 8.0, 4‴-H), 6.38 (1H, d, *J* = 8.0, 1.5, 3‴-H), 5.92 (1H, d, *J* = 1.2, 8-H), 5.84 (1H, d, *J* = 1.2, 6-H), 5.42 (1H, s, 3-H), 5.27 (1H, s, 2-H), 4.13 (1H, d, *J* = 1.2, 4-H), 4.08 (2H, s, S-C-H); ^{13}C NMR (125 MHz, DMSO-d_6) δ: 165.0 (O-C-O), 158.0 (C-5), 157.1 (C-7), 155.4 (C-9), 151.5 (C-2‴), 145.4 (C-3″, 5″), 144.8 (C-3′, 4′), 142.3 (C-5‴), 138.8 (C-4″), 128.5 (C-1′), 118.5 (C-1″), 117.5 (C-6′), 115.2 (C-5′), 114.1 (C-2′), 110.5 (C-4‴), 108.6 (C-2″, 6″), 107.5 (C-3‴), 96.8 (C-10), 95.9 (C-6), 94.0 (C-8), 72.6 (C-2), 71.0 (C-3), 40.0 (C-4), 27.9 (S-C).

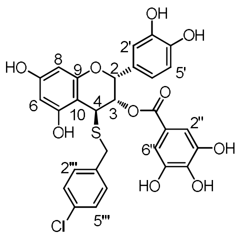

JP-3: UV λ_{max} (MeOH) nm: 278, 216; mp. 142.0~143.7°C; $[\alpha]_D^{25}$ -93.6° (*c* 0.1 MeOH); HR-ESI-MS (*m/z* 599.0782 [M+H]$^+$, calcd, 599.0773); ^{1}H NMR (500 MHz, DMSO-d_6) δ: 7.48 (2H, m, 3‴, 5‴-H), 7.39 (2H, m, 2‴, 6‴-H), 6.86 (1H, s, 2′-H), 6.79 (2H, s, 2″, 6″-H), 6.68 (2H, m, 5′, 6′-H), 5.92 (1H, d, *J* = 2.0, 8-H), 5.83 (1H, d, *J* = 2.0, 6-H), 5.44 (1H, s, 3-H), 5.23 (1H, s, 2-H), 4.05 (2H, s, S-C-H), 4.04 (1H, d, *J* = 2.0, 4-H); ^{13}C NMR (125 MHz, DMSO-d_6) δ: 165.1 (O-C-O), 158.1 (C-5), 157.2 (C-7), 155.4 (C-9), 145.4 (C-3″, 5″), 144.9 (C-3′, 4′), 138.9 (C-4″), 138.0 (C-1‴), 131.3 (C-4‴), 130.7 (C-2‴, 6‴), 128.5 (C-1′), 128.2 (C-3‴, 5‴), 118.5 (C-1″), 117.5 (C-6′), 115.2 (C-5′), 114.2 (C-2′), 108.6 (C-2″, 6″), 96.9 (C-10), 95.9 (C-6), 94.0 (C-8), 72.7 (C-2), 71.2 (C-3), 40.0 (C-4), 35.0 (S-C).

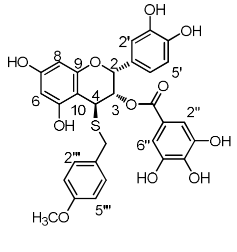

JP-4: UV λ_{max} (MeOH) nm: 278, 216; mp. 142.1~144.0°C; $[\alpha]_D^{25}$ -89.1° (*c* 0.1 MeOH); HR-ESI-MS (*m/z* 595.1265 [M+H]$^+$, calcd, 595.1269); ^{1}H NMR (500 MHz,

DMSO-d_6) δ: 7.37 (2H, m, 2‴, 6‴-H), 6.90 (2H, m, 3‴, 5‴-H), 6.84 (1H, s, 2′-H), 6.79 (2H, s, 2″, 6″-H), 6.67 (2H, m, 5′, 6′-H), 5.92 (1H, d, *J* = 2.5, 8-H), 5.83 (1H, d, *J* = 2.5, 6-H), 5.44 (1H, s, 3-H), 5.25 (1H, s, 2-H), 4.08 (1H, d, *J* = 2.0, 4-H), 4.00 (2H, dd, *J* = 27.0, 13.5, S-C-H), 3.75 (3H, s, -OCH_3); ^{13}C NMR (125 MHz, DMSO-d_6) δ: 165.0 (O-C-O), 158.1 (C-5), 157.9 (C-4‴), 157.1 (C-7), 155.3 (C-9), 145.4 (C-3″, 5″), 144.8 (C-3′, 4′), 138.8 (C-4″), 130.4 (C-1‴), 129.9 (C-2‴, 6‴), 128.5 (C-1′), 118.6 (C-1″), 117.4 (C-6′), 115.1 (C-5′), 114.1 (C-2′), 113.7 (C-3‴, 5‴), 108.6 (C-2″, 6″), 97.1 (C-10), 95.9 (C-6), 94.0 (C-8), 72.6 (C-2), 71.2 (C-3), 55.0 (C-OCH_3), 45.6 (C-4), 35.2 (S-C).

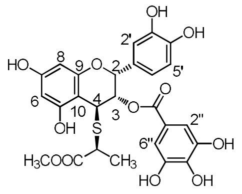

JP-5: UV λ_{max} (MeOH) nm: 278, 216; mp. 144.4~146.3°C; $[\alpha]_D^{25}$ -47.8° (*c* 0.1 MeOH); HR-ESI-MS (*m/z* 561.1065 $[M+H]^+$, calcd, 561.1061); ^{1}H NMR (500 MHz, DMSO-d_6) δ: 6.84 (1H, d, *J* = 2.0, 2′-H), 6.78 (2H, m, 2″, 6″-H), 6.75 (1H, dd, *J* = 8.0, 2.0, 6′-H), 6.69 (1H, d, *J* = 8.0, 5′-H), 5.95 (1H, d, *J* = 2.5, 8-H), 5.84 (1H, d, *J* = 2.5, 6-H), 5.33 (1H, s, 3-H), 5.30 (1H, s, 2-H), 4.17 (1H, d, *J* = 2.0, 4-H), 3.91 (1H, dd, *J* = 14.0, 7.0, S-C-H), 3.72 (3H, s, -O-C-O-CH_3), 1.55 (3H, d, *J* = 7.0, -C-CH_3); ^{13}C NMR (125 MHz, DMSO-d_6) δ: 173.0 (-$COOCH_3$), 165.0 (O-C-O), 158.2 (C-5), 157.2 (C-7), 155.2 (C-9), 145.5 (C-3″, 5″), 145.0 (C-3′), 144.9 (C-4′), 138.9 (C-4″), 128.5 (C-1′), 118.5 (C-1″), 117.5 (C-6′), 115.3 (C-5′), 114.1 (C-2′), 108.6 (C-2″, 6″), 97.1 (C-10), 96.0 (C-6), 94.1 (C-8), 72.8 (C-2), 71.8 (C-3), 52.3 (C-OCH_3), 42.4 (S-C), 40.0 (C-4), 18.3 (C-CH_3).

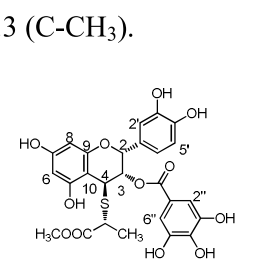

JP-6: UV λ_{max} (MeOH) nm: 278, 216; mp. 147.8~150.2°C; $[\alpha]_D^{25}$ -164.3° (*c* 0.1 MeOH); HR-ESI-MS (*m/z* 561.107 $[M+H]^+$, calcd, 561.1075); ^{1}H NMR (500 MHz,

DMSO-d_6) δ: 6.86 (1H, d, *J* = 2.0, 2′-H), 6.79 (2H, m, 2″, 6″-H), 6.76 (1H, dd, *J* = 8.0, 2.0, 6′-H), 6.69 (1H, d, *J* = 8.0, 5′H), 5.94 (1H, d, *J* = 2.5, 8-H), 5.84 (1H, d, *J* = 2.5, 6-H), 5.39 (1H, s, 3-H), 5.23 (1H, s, 2-H), 4.26 (1H, d, *J* = 2.0, 4-H), 3.84 (1H, dd, *J* = 14.0, 7.0, S-C-H), 3.69 (3H, s, -O-C-O-CH_3), 1.48 (3H, d, *J* = 7.0, -C-CH_3); ^{13}C NMR (125 MHz, DMSO-d_6) δ: 173.0 (-$COOCH_3$), 164.9 (O-C-O), 158.2 (C-5), 157.2 (C-7), 155.5 (C-9), 145.5 (C-3″, 5″), 145.0 (C-3′), 144.9 (C-4′), 138.9 (C-4″), 128.5 (C-1′), 118.5 (C-1″), 117.5 (C-6′), 115.3 (C-5′), 114.1 (C-2′), 108.7 (C-2″, 6″), 96.4 (C-10), 95.9 (C-6), 94.1 (C-8), 72.9 (C-2), 71.6 (C-3), 52.1 (C-OCH_3), 40.9 (S-C), 40.0 (C-4), 17.5 (C-CH_3).

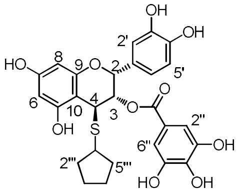

JP-7: UV λ_{max} (MeOH) nm: 278, 216; mp. 146.6~149.5°C; $[\alpha]_D^{25}$ -107.5° (*c* 0.1 MeOH); HR-ESI-MS (*m/z* 543.1323 [M+H]$^+$, calcd, 543.1319); ^{1}H NMR (500 MHz, DMSO-*d*$_6$) δ: 6.85 (1H, s, 2′-H), 6.76 (2H, m, 2″, 6″-H), 6.75 (1H, dd, *J* = 8.0, 2.0, 6′-H), 6.68 (1H, d, *J* = 8.0, 5′-H), 5.92 (1H, d, *J* = 2.0, 8-H), 5.81 (1H, d, *J* = 2.0, 6-H), 5.41 (1H, s, 3-H), 5.21 (1H, s, 2-H), 4.02 (1H, s, 4-H), 3.44 (1H, m, 1‴-H), 2.10 (2H, m, 2‴, 5‴-H), 1.73 (2H, m, 2‴, 5‴-H), 1.58 (4H, m, 3‴, 4‴-H); ^{13}C NMR (125 MHz, DMSO-*d*$_6$) δ: 165.2 (O-C-O), 157.8 (C-5), 157.0 (C-7), 155.1 (C-9), 145.4 (C-3″, 5″), 144.9 (C-3′, 4′), 138.9 (C-4″), 128.7 (C-1′), 118.5 (C-1″), 117.5 (C-6′), 115.3 (C-5′), 114.0 (C-2′), 108.6 (C-2″, 6″), 97.8 (C-10), 95.9 (C-6), 94.0 (C-8), 72.8 (C-2), 72.0 (C-3), 44.2 (C-4), 40.0 (S-C), 33.8 (C-2‴), 33.5 (C-5‴) , 24.6 (C-3‴), 24.2 (C-4‴).

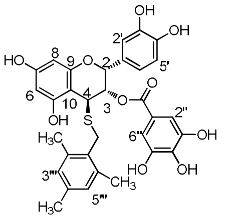

JP-8: UV λ_{max} (MeOH) nm: 278, 216; mp. 140.1~142.3°C; $[\alpha]_D^{25}$ -26° (*c* 0.1 MeOH); HR-ESI-MS (*m/z* 607.1643 [M+H]$^+$, calcd, 607.1646); ^{1}H NMR (500 MHz,

DMSO-d_6) δ: 6.88 (1H, d, *J* = 1.5, 2′-H), 6.86 (2H, s, 2″, 6″-H), 6.82 (2H, s, 3‴, 5‴-H), 6.79 (1H, dd, *J* = 8.5, 1.5, 6′-H), 6.72 (1H, d, *J* = 1.5, 5′-H), 5.96 (1H, d, *J* = 2.5, 8-H), 5.84 (1H, d, *J* = 2.5, 6-H), 5.45 (1H, s, 3-H), 5.33 (1H, s, 2-H), 4.14 (2H, s, 4-H), 4.05 (1H, s, S-C-H), 2.42 (6H, s, 2‴, 6‴-CH_3), 2.22 (3H, s, 4‴-CH_3); ^{13}C NMR (125 MHz, DMSO-d_6) δ: 165.2 (O-C-O), 158.0 (C-5), 157.4 (C-7), 155.2 (C-9), 145.5 (C-3″, 5″), 145.0 (C-3′, 4′), 138.9 (C-4″), 136.5 (C-2‴, 6‴), 135.9 (C-1‴), 130.3 (C-4‴), 128.7 (C-3‴, 5‴), 128.6 (C-1′), 118.6 (C-1″), 117.5 (C-6′), 115.3 (C-5′), 114.1 (C-2′), 108.7 (C-2″, 6″), 97.3 (C-10), 96.0 (C-6), 94.1 (C-8), 73.1 (C-2), 71.7 (C-3), 40.6 (C-4), 31.1 (S-C), 20.6 (C-4‴CH_3), 19.1 (C-2‴, 6‴CH_3).

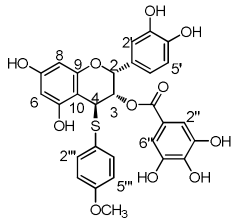

JP-9: UV λ_{max} (MeOH) nm: 278, 216; mp. 152.5~154.3°C; $[\alpha]_D^{25}$ -38.7° (*c* 0.1 MeOH); HR-ESI-MS (*m/z* 581.1117 $[M+H]^+$, calcd, 581.1112); ^{1}H NMR (500 MHz, DMSO-d_6) δ: 7.56 (2H, d, *J* = 9.0, 3‴, 5‴-H), 7.00 (2H, d, *J* = 9.0, 2‴, 6‴-H), 6.82 (1H, d, *J* = 2.0, 2′-H), 6.72 (1H, dd, *J* = 8.5, 2.0, 6′-H), 6.69 (2H, s, 2″, 6″-H), 6.67 (1H, d, *J* = 8.5, 5′-H), 5.98 (1H, d, *J* = 2.0, 8-H), 5.86 (1H, d, *J* = 2.0, 6-H), 5.57 (1H, s, 3-H), 5.13 (1H, brs, 2-H), 4.32 (2H, d, *J* = 2.0, 4-H), 3.79 (3H, s, -OCH_3); ^{13}C NMR (125 MHz, DMSO-d_6) δ: 164.6 (O-C-O), 159.2 (C-5), 158.3 (C-7), 157.3 (C-9), 155.5 (C-4‴), 145.4 (C-3″, 5″), 144.9 (C-3′, 4′), 138.8 (C-4″), 134.4 (C-2‴, 6‴), 128.5 (C-1′), 124.4 (C-1‴), 118.4 (C-1″), 117.4 (C-6′), 115.2 (C-5′), 114.8 (C-3‴, 5‴), 114.0 (C-2′), 108.6 (C-2″, 6″), 96.6 (C-10), 96.0 (C-6), 94.1 (C-8), 72.6 (C-2), 70.6 (C-3), 55.2 (C-OCH_3), 43.3 (C-4).

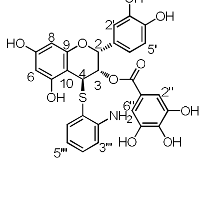

JP-10: UV λ_{max} (MeOH) nm: 278, 216; mp. 157.2~160.3^{o}C; $[\alpha]^{25}_{D}$ -192.1° (*c* 0.1 MeOH); HR-ESI-MS (*m/z* 588.0931 $[M+Na]^+$, calcd, 588.0935); ^{1}H NMR (500 MHz, DMSO-d_6) δ: 7.38 (1H, dd, *J* = 7.5, 1.5, 6‴-H), 7.14 (1H, m, 4‴-H), 6.88 (1H, d, *J* = 2.0, 2′-H), 6.77 (2H, m, 6′, 5‴-H), 6.67 (1H, d, *J* = 8.5, 5′-H), 6.66 (2H, s, 2″, 6″-H), 6.57 (1H, m, 3‴-H), 6.03 (1H, d, *J* = 2.5, 8-H), 5.89 (1H, d, *J* = 2.5, 6-H), 5.71 (1H, d, *J* = 2.5, 3-H), 5.15 (1H, d, *J* = 2.5, 2-H), 4.19 (1H, d, *J* = 2.5, 4-H); ^{13}C NMR (125 MHz, DMSO-d_6) δ: 164.3 (O-C-O), 158.3 (C-5), 157.3 (C-7), 155. 8 (C-9), 150.6 (C-2‴), 145.3 (C-3″, 5″), 144.9 (C-3′), 144.8 (C-4′), 138.7 (C-4″), 136.7 (C-6‴), 130.6 (C-1′), 128.7 (C-4‴), 118.5 (C-1″), 117.4 (C-6′), 116.1 (C-1‴), 115.2 (C-5′), 114.4 (C-5‴), 114.0 (C-2′), 113.2 (C-3‴), 108.5 (C-2″, 6″), 96.7 (C-10), 96.0 (C-6), 94.4 (C-8), 72.6 (C-2), 70.4 (C-3), 40.7 (C-4).

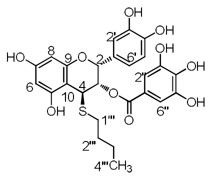

JP-11: UV λ_{max} (MeOH) nm: 278, 216; mp. 150.6~152.1^{o}C; $[\alpha]^{25}_{D}$ -78.7° (*c* 0.1 MeOH); HR-ESI-MS (*m/z* 531.1324 $[M+H]^+$, calcd, 531.1319); ^{1}H NMR (500 MHz, DMSO-d_6) δ: 6.86 (1H, d, *J* = 1.5, 2′-H), 6.79 (2H, s, 2″, 6″-H), 6.77 (1H, dd, *J* = 8.5, 1.5, 6′-H), 6.70 (1H, d, *J* = 8.5, 5′-H), 5.94 (1H, d, *J* = 2.0, 8-H), 5.84 (1H, d, *J* = 2.0, 6-H), 5.44 (1H, s, 3-H), 5.18(1H, s, 2-H), 4.00 (1H, d, *J* = 2.0, 4-H), 2.82 (1H, s, 1‴-H), 1.70 (2H, m, 2‴-H), 1.48 (2H, m, 3‴-H), 0.94 (3H, t, 4‴-H); ^{13}C NMR (125 MHz, DMSO-d_6) δ: 165.2 (O-C-O), 157.8 (C-5), 157.2 (C-7), 155.2 (C-9), 145.4 (C-3″, 5″), 144.9 (C-3′, 4′), 138.8 (C-4″), 128.6 (C-1′), 118.5 (C-1″), 117.5 (C-6′), 115.3 (C-5′), 114.1 (C-2′), 108.6 (C-2″, 6″), 97.6 (C-10), 95.9 (C-6), 94.0 (C-8), 72.7 (C-2), 71.8 (C-3), 40.0 (C-4), 31.4 (C-1‴, 2‴), 21.2 (C-3‴), 13.5 (C-4‴).

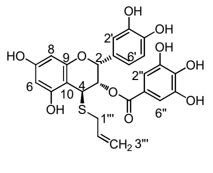

JP-12: UV λ_{max} (MeOH) nm: 278, 216; mp. 143.8~146.0^{o}C; $[\alpha]^{25}_{D}$ -89.9° (*c* 0.1

MeOH); HR-ESI-MS (*m/z* 515.1019 $[M+H]^+$, calcd, 515.1006); ^{1}H NMR (500 MHz, DMSO-d_6) δ: 6.86 (1H, d, *J* = 1.5, 2′-H), 6.79 (2H, s, 2″, 6″-H), 6.77 (1H, dd, *J* = 8.5, 1.5, 6′-H), 6.70 (1H, d, *J* = 8.5, 5′-H), 5.98 (1H, m, 2‴-H), 5.94 (1H, d, *J* = 2.0, 8-H), 5.84 (1H, d, *J* = 2.0, 6-H), 5.45 (1H, s, 3-H), 5.30 (1H, d, *J* = 18.0, 3‴-H), 5.25 (1H, s, 2-H), 5.11 (1H, d, *J* = 10.5, 3‴-H), 4.02 (1H, d, *J* = 2.0, 4-H), 3.49 (2H, m, 1‴-H); ^{13}C NMR (125 MHz, DMSO-d_6) δ: 165.5 (O-C-O), 158.5 (C-5), 157.7 (C-7), 155.9 (C-9), 145.9 (C-3″, 5″), 145.4 (C-3′, 4′), 139.4 (C-4″), 135.2 (C-2‴), 129.1 (C-1′), 119.0 (C-1″), 118.0 (C-3‴), 117.7 (C-6′), 115.7 (C-5′), 114.6 (C-2′), 109.1 (C-2″, 6″), 97.7 (C-10), 96.4 (C-6), 94.6 (C-8), 73.3 (C-2), 71.7 (C-3), 39.1 (C-4), 35.1 (C-1‴).

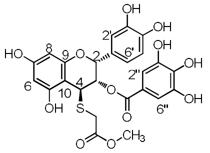

JP-13: UV λ_{max} (MeOH) nm: 278, 216; mp. 142.2~143.4°C; $[\alpha]_D^{25}$ -75.1° (*c* 0.1 MeOH); HR-ESI-MS (*m/z* 547.0913 $[M+H]^+$, calcd, 547.0905); ^{1}H NMR (500 MHz, DMSO-d_6) δ: 6.88 (1H, d, *J* = 1.5, 2′-H), 6.78 (2H, s, 2″, 6″-H), 6.77 (1H, dd, *J* = 8.0, 1.5, 6′-H), 6.69 (1H, d, *J* = 8.0, 5′-H), 5.95 (1H, d, *J* = 2.5, 8-H), 5.85 (1H, d, *J* = 2.5, 6-H), 5.44 (1H, s, 3-H), 5.27 (1H, s, 2-H), 4.14 (1H, d, *J* = 2.0, 4-H), 3.68 (3H, s, -O-C-O-CH_3), 3.67 (2H, m, S-C-H); ^{13}C NMR (125 MHz, DMSO-d_6) δ: 171.2 (O-C-OCH_3), 165.5 (O-C-O), 158.8 (C-5), 157.8 (C-7), 155.9 (C-9), 145.9 (C-3″, 5″), 145.4 (C-3′, 4′), 139.3 (C-4″), 129.0 (C-1′), 119.0 (C-1″), 118.1 (C-6′), 115.7 (C-5′), 114.7 (C-2′), 109.1 (C-2″, 6″), 97.1 (C-10), 96.4 (C-6), 94.6 (C-8), 73.1 (C-2), 71.5 (C-3), 52.7 (C-OCH_3), 40.8 (C-4), 34.2 (S-C).

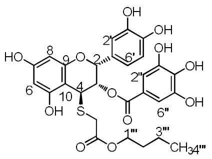

JP-14: UV λ_{max} (MeOH) nm: 278, 216; mp. 139.7~141.5°C; $[\alpha]_D^{25}$ -115.5° (*c* 0.1 MeOH); HR-ESI-MS (*m/z* 589.1386 $[M+H]^+$, calcd, 589.1388); ^{1}H NMR (500 MHz, DMSO-d_6) δ: 6.88 (1H, d, *J* = 2.0, 2′-H), 6.78 (2H, s, 2″, 6″-H), 6.77 (1H, dd, *J* = 8.0, 1.5, 6′-H), 6.69 (1H, d, *J* = 8.0, 5′-H), 5.95 (1H, d, *J* = 2.0, 8-H), 5.85 (1H, d, *J* = 2.0,

6-H), 5.44 (1H, s, 3-H), 5.28 (1H, s, 2-H), 4.17 (1H, d, J = 2.0, 4-H), 4.10 (2H, m, 1‴-H), 3.65 (2H, m, S-C-H), 1.57 (2H, m, 2‴-H), 1.32 (2H, m, 3‴-H), 0.85 (3H, t, 4‴-H); ^{13}C NMR (125 MHz, DMSO-d_6) δ: 170.7 (O-C-OCH$_2$), 165.4 (O-C-O),158.8 (C-5), 157.9 (C-7), 156.0 (C-9), 145.9 (C-3″, 5″), 145.4 (C-3′, 4′), 139.3 (C-4″), 129.0 (C-1′), 119.0 (C-1″), 118.1 (C-6′), 115.7 (C-5′), 114.7 (C-2′), 109.1 (C-2″, 6″), 97.1 (C-10), 96.4 (C-6), 94.5 (C-8), 73.1 (C-2), 71.4 (C-3), 65.1 (C-1‴), 40.0 (C-4), 34.2 (S-C), 30.5 (C-2‴), 19.0 (C-3‴), 14.0 (C-4‴).

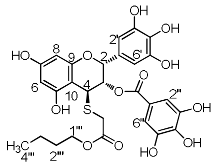

JP-15: UV λ_{max} (MeOH) nm: 278, 216; mp. 138.9~140.1°C; $[\alpha]_D^{25}$ -102.3° (c 0.1 MeOH); HR-ESI-MS (m/z 605.1339 $[M+H]^+$, calcd, 605.1337); ^{1}H NMR (500 MHz, DMSO-d_6) δ: 6.78 (2H, s, 2″, 6″-H), 6.43 (2H, s, 2′, 6′-H), 5.95 (1H, d, J = 2.0, 8-H), 5.85 (1H, d, J = 2.0, 6-H), 5.35 (1H, s, 3-H), 5.30 (1H, s, 2-H), 4.15 (1H, d, J = 1.5, 4-H), 4.10 (2H, m, 1‴-H), 3.63 (2H, m, S-C-H), 1.57 (2H, m, 2‴-H), 1.33 (2H, m, 3‴-H), 0.85 (3H, t, 4‴-H); ^{13}C NMR (125 MHz, DMSO-d_6) δ: 170.7 (O-C-OCH$_2$), 165.5 (O-C-O),158.8 (C-5), 157.9 (C-7), 156.0 (C-9), 146.3 (C-3′, 5′), 145.9 (C-3″, 5″), 139.3 (C-4″), 133.1 (C-4′), 128.2 (C-1′), 119.1 (C-1″), 109.1 (C-2″, 6″), 106.0 (C-2′, 6′), 97.2 (C-10), 96.4 (C-6), 94.6 (C-8), 73.2 (C-2), 71.3 (C-3), 65.1 (C-1‴), 40.0 (C-4), 34.2 (S-C), 30.5 (C-2‴), 19.1 (C-3‴), 14.0 (C-4‴).

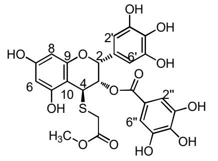

JP-16: UV λ_{max} (MeOH) nm: 278, 216; mp. 124.7~127.0°C; $[\alpha]_D^{25}$ -94.5° (c 0.1 MeOH); HR-ESI-MS (m/z 563.0864 $[M+H]^+$, calcd, 563.0867); ^{1}H NMR (500 MHz, DMSO-d_6) δ: 6.78 (2H, s, 2″, 6″-H), 6.43 (2H, s, 2′, 6′-H), 5.95 (1H, d, J = 2.0, 8-H), 5.85 (1H, d, J = 2.0, 6-H), 5.34 (1H, s, 3-H), 5.29 (1H, s, 2-H), 4.11 (1H, d, J = 2.0, 4-H), 3.69 (3H, m, -O-C-O-CH$_3$), 3.63 (2H, m, S-CH$_2$); ^{13}C NMR (125 MHz,

DMSO-d_6) δ: 171.1 (O-C-OCH_3), 165.5 (O-C-O), 158.8 (C-5), 157.8 (C-7), 155.9 (C-9), 146.3 (C-3′, 5′), 145.9 (C-3″, 5″), 139.3 (C-4″), 133.1 (C-4′), 128.2 (C-1′), 119.1 (C-1″), 109.2 (C-2″, 6″), 105.7 (C-2′, 6′), 97.3 (C-10), 96.4 (C-6), 94.6 (C-8), 73.2 (C-2), 71.5 (C-3), 52.7 (C-OCH_3), 40.0 (C-4), 34.2 (S-C).

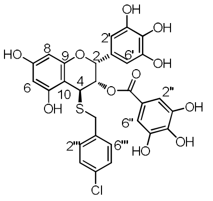

JP-17: UV λ_{max} (MeOH) nm: 278, 216; mp. 145.1~147.4°C; $[\alpha]_D^{25}$ -123.6° (*c* 0.1 MeOH); HR-ESI-MS (*m/z* 615.0727 $[M+H]^+$, calcd, 615.0722); ^1H NMR (500 MHz, DMSO-d_6) δ: 7.48 (2H, d, *J* = 8.5, 3‴, 5‴-H), 7.39 (2H, d, *J* = 8.5, 2‴, 6‴-H), 6.79 (2H, s, 2″, 6″-H), 6.41 (2H, s, 2′, 6′-H), 5.91 (1H, d, *J* = 2.5, 8-H), 5.83 (1H, d, *J* = 2.5, 6-H), 5.36 (1H, s, 3-H), 5.26(1H, s, 2-H), 4.07 (2H, m, S-CH_2), 4.00 (1H, s, 4-H); ^{13}C NMR (125 MHz, DMSO-d_6) δ: 165.7 (O-C-O), 158.5 (C-5), 157.7 (C-7), 155.9 (C-9), 146.3 (C-3′, 5′), 145.9 (C-3″, 5″), 139.4 (C-4″), 138.2 (C-1‴), 133.1 (C-4′), 131.9 (C-4‴), 131.2 (C-2‴, 6‴), 128.7 (C-3‴, 5‴), 128.3 (C-1′), 119.1 (C-1″), 109.2 (C-2″, 6″), 105.9 (C-2′, 6′), 97.5 (C-10), 96.4 (C-6), 94.5 (C-8), 73.3 (C-2), 71.7 (C-3), 40.0 (C-4), 35.4 (S-C).

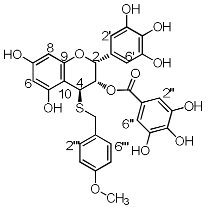

JP-18: UV λ_{max} (MeOH) nm: 278, 216; mp. 135.4~136.7°C; $[\alpha]_D^{25}$ -76.6° (*c* 0.1 MeOH); HR-ESI-MS (*m/z* 611.1214 $[M+H]^+$, calcd, 611.1218); ^1H NMR (500 MHz, DMSO-d_6) δ: 7.36 (2H, d, *J* = 8.5, 2‴, 6‴-H), 6.88 (2H, d, *J* = 8.5, 3‴, 5‴-H), 6.79 (2H, s, 2″, 6″-H), 6.40 (2H, s, 2′, 6′-H), 5.92 (1H, d, *J* = 2.0, 8-H), 5.83 (1H, d, *J* = 2.0, 6-H), 5.37 (1H, s, 3-H), 5.29 (1H, s, 2-H), 4.03 (2H, d, *J* = 2.0, 4-H), 4.01 (2H, m, S-CH_2), 3.75 (3H, s, -OCH_3); ^{13}C NMR (125 MHz, DMSO-d_6) δ: 165.6 (O-C-O), 158.7 (C-5), 158.5 (C-4‴), 157.7 (C-7), 155.9 (C-9), 146.3 (C-3′, 5′), 145.9 (C-3″, 5″),

139.4 (C-4″), 133.0 (C-4′), 130.7 (C-1‴), 130.5 (C-2‴, 6‴), 128.4 (C-1′), 119.2 (C-1″), 114.2 (C-3‴, 5‴), 109.2 (C-2″, 6″), 105.9 (C-2′, 6′), 97.7 (C-10), 96.4 (C-6), 94.5 (C-8), 73.3 (C-2), 71.7 (C-3), 55.5 (C-OCH_3), 40.0 (C-4), 35.7 (S-C).

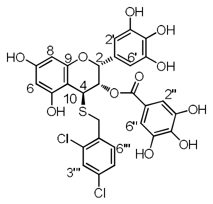

JP-19: UV λ_{max} (MeOH) nm: 278, 216; mp. 145.3~146.7°C; $[\alpha]_D^{25}$ -57.1° (*c* 0.1 MeOH); HR-ESI-MS (*m/z* 649.0337 $[M+H]^+$, calcd, 649.0333); ^{1}H NMR (500 MHz, DMSO-d_6) δ: 7.65 (1H, d, *J* = 2.5, 3‴-H), 7.62 (1H, dd, *J* = 8.5, 2.5, 5‴-H), 7.41 (1H, d, *J* = 8.5, 6‴-H), 6.80 (2H, s, 2″, 6″-H), 6.41 (2H, s, 2′, 6′-H), 5.91 (1H, d, *J* = 2.0, 8-H), 5.83 (1H, d, *J* = 2.0, 6-H), 5.36 (1H, s, 3-H), 5.30 (1H, s, 2-H), 4.13 (2H, m, S-CH_2), 4.05 (1H, d, *J* = 2.0, 4-H); ^{13}C NMR (125 MHz, DMSO-d_6) δ: 165.8 (O-C-O), 158.6 (C-5), 157.7 (C-7), 155.9 (C-9), 146.3 (C-3′, 5′), 145.9 (C-3″, 5″), 139.4 (C-4″), 135.7 (C-2‴), 134.6 (C-4‴), 133.1 (C-6‴), 132.9 (C-4′), 132.7 (C-1′), 129.5 (C-3‴), 128.3 (C-1‴), 127.8 (C-5‴), 119.1 (C-1″), 109.2 (C-2″, 6″), 105.9 (C-2′, 6′), 97.3 (C-10), 96.4 (C-6), 94.5 (C-8), 73.4 (C-2), 71.7 (C-3), 40.9 (C-4), 33.5 (S-C).

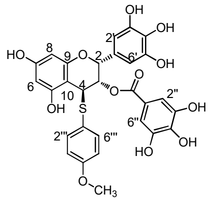

JP-20: UV λ_{max} (MeOH) nm: 278, 216; mp. 129.3~131.0°C; $[\alpha]_D^{25}$ -41.5° (*c* 0.1 MeOH); HR-ESI-MS (*m/z* 597.1072 $[M+H]^+$, calcd, 597.1061); ^{1}H NMR (500 MHz, DMSO-d_6) δ: 7.56 (2H, d, *J* = 8.5, 3‴, 5‴-H), 7.02 (2H, d, *J* = 8.5, 2‴, 6‴-H), 6.70 (2H, s, 2″, 6″-H), 6.38 (2H, s, 2′, 6′-H), 5.99 (1H, d, *J* = 2.5, 8-H), 5.87 (1H, d, *J* = 2.5, 6-H), 5.51 (1H, s, 3-H), 5.17 (1H, s, 2-H), 4.32 (1H, d, *J* = 2.0, 4-H), 3.81 (3H, s, -OCH_3); ^{13}C NMR (125 MHz, DMSO-d_6) δ: 164.6 (O-C-O), 159.2 (C-5), 158.3 (C-7), 157.3 (C-9), 155.5 (C-4‴), 145.8 (C-3′, 5′), 145.3 (C-3″, 5″), 138.8 (C-4″), 134.2 (C-2‴, 6‴), 132.5 (C-4′), 127.8 (C-1′), 124.5 (C-1‴), 118.5 (C-1″), 114.9 (C-3‴, 5‴),

108.7 (C-2″, 6″), 105.2 (C-2′, 6′), 96.7 (C-10), 96.0 (C-6), 94.1 (C-8), 72.6 (C-2), 70.5 (C-3), 55.2 (C-OCH_3), 43.4 (S-C).

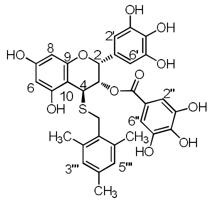

JP-21: UV λ_{max} (MeOH) nm: 278, 216; mp. 139.9~141.3°C; $[\alpha]_D^{25}$ -9.4° (*c* 0.1 MeOH); HR-ESI-MS (*m/z* 623.1583 $[M+H]^+$, calcd, 623.1582); ^{1}H NMR (500 MHz, DMSO-d_6) δ: 6.85 (2H, s, 3‴, 5‴-H), 6.80 (2H, s, 2″, 6″-H), 6.43 (2H, s, 2′, 6′-H), 5.95 (1H, d, *J* = 2.0, 8-H), 5.83 (1H, d, *J* = 2.0, 6-H), 5.35 (2H, s, 3, 2-H), 4.11 (1H, s, 4-H), 4.09 (2H, s, S-CH_2), 2.42 (6H, s, 2‴, 6‴-CH_3), 2.20 (3H, s, 4‴-CH_3); ^{13}C NMR (125 MHz, DMSO-d_6) δ: 165.2 (O-C-O), 158.0 (C-5), 157.3 (C-7), 155.2 (C-9), 145.8 (C-3′, 5′), 145.4 (C-3″, 5″), 138.9 (C-4″), 136.5 (C-2‴, 6‴), 135.9 (C-1‴), 132.6 (C-4‴), 130.2 (C-4′), 128.7 (C-3‴, 5‴), 127.9 (C-1′), 118.6 (C-1″), 108.7 (C-2″, 6″), 105.3 (C-2′, 6′), 97.3 (C-10), 96.0 (C-6), 94.0 (C-8), 73.0 (C-2), 71.6 (C-3), 40.7 (C-4), 31.0 (S-C), 20.5 (4‴-CH_3), 19.1 (2‴, 6‴-CH_3).

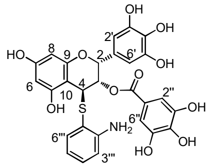

JP-22: UV λ_{max} (MeOH) nm: 278, 216; mp. 159.7~162.1°C; $[\alpha]_D^{25}$ -189.3° (*c* 0.1 MeOH); HR-ESI-MS (*m/z* 604.0887 $[M+H]^+$, calcd, 604.0884); ^{1}H NMR (500 MHz, DMSO-d_6) δ: 7.35 (1H, d, *J* = 7.5, 6‴-H), 7.14 (1H, m, 4‴-H), 6.76 (1H, d, *J* = 7.5, 5‴-H), 6.63 (2H, s, 2″, 6″-H), 6.57 (1H, m, 3‴-H), 6.42 (2H, s, 2′, 6′-H), 6.01 (1H, d, *J* = 1.0, 8-H), 5.88 (1H, d, *J* = 1.0, 6-H), 5.61 (1H, s, 3-H), 5.16 (1H, s, 2-H), 4.16 (1H, s, 4-H); ^{13}C NMR (125 MHz, DMSO-d_6) δ: 164.2 (O-C-O), 158.3 (C-5), 157.3 (C-7), 155.8 (C-9), 150.6 (C-2‴), 145.8 (C-3′, 5′), 145.3 (C-3″, 5″), 138.7 (C-4″), 136.6 (C-6‴), 132.4 (C-4′), 130.6 (C-1′), 127.9 (C-4‴), 118.6 (C-1″), 116.0 (C-1‴), 114.4 (C-5‴), 113.2 (C-3‴), 108.6 (C-2″, 6″), 105.2 (C-2′, 6′), 96.8 (C-10),

96.0 (C-6), 94.4 (C-8), 72.6 (C-2), 70.4 (C-3), 40.7 (C-4).

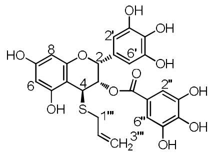

JP-23: UV λ_{max} (MeOH) nm: 278, 216; mp. 140.3~142.1°C; $[\alpha]^{25}_{D}$ -101.6° (*c* 0.1 MeOH); HR-ESI-MS (*m/z* 531.0949 $[M+H]^+$, calcd, 531.0956); ^{1}H NMR (500 MHz, DMSO-d_6) δ: 6.76 (2H, s, 2″, 6″-H), 6.40 (2H, s, 2′, 6′-H), 6.01 (1H, m, 2‴-H), 5.92 (1H, d, *J* = 2.0, 8-H), 5.82 (1H, d, *J* = 2.0, 6-H), 5.36 (1H, s, 3-H), 5.31 (1H, d, *J* = 18.0, 3‴-H), 5.25 (1H, s, 2-H), 5.11 (1H, d, *J* = 10.5, 3‴-H), 3.97 (1H, s, 4-H), 3.46 (2H, m, 1‴-H); ^{13}C NMR (125 MHz, DMSO-d_6) δ: 165.0 (O-C-O), 157.9 (C-5), 157.2 (C-7), 155.4 (C-9), 145.8 (C-3′, 5′), 145.4 (C-3″, 5″), 138.8 (C-4″), 134.7 (C-2‴), 132.5 (C-4′), 127.8 (C-1′), 118.6 (C-1″), 117.2 (C-3‴), 108.7 (C-2″, 6″), 105.3 (C-2′, 6′), 97.3 (C-10), 95.9 (C-6), 94.0 (C-8), 72.7 (C-2), 71.1 (C-3), 38.7 (C-4), 34.6 (S-C).

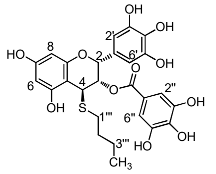

JP-24: UV λ_{max} (MeOH) nm: 278, 216; mp. 145.6~147.4°C; $[\alpha]^{25}_{D}$ -88.3° (*c* 0.1 MeOH); HR-ESI-MS (*m/z* 547.1269 $[M+H]^+$, calcd, 547.1269); ^{1}H NMR (500 MHz, DMSO-d_6) δ: 6.77 (2H, s, 2″, 6″-H), 6.40 (2H, s, 2′, 6′-H), 5.92 (1H, d, *J* = 2.0, 8-H), 5.82 (1H, d, *J* = 2.0, 6-H), 5.34 (1H, s, 3-H), 5.18 (1H, s, 2-H), 3.95 (1H, s, 4-H), 2.81 (2H, m, 1‴-H), 1.69 (2H, m, 2‴-H), 1.46 (2H, m, 3‴-H), 0.92 (3H, t, 4‴-H); ^{13}C NMR (125 MHz, DMSO-d_6) δ: 165.2 (O-C-O), 157.8 (C-5), 157.2 (C-7), 155.2 (C-9), 145.8 (C-3′, 5′), 145.4 (C-3″, 5″), 138.8 (C-4″), 132.5 (C-4′), 127.9 (C-1′), 118.6 (C-1″), 108.7 (C-2″, 6″), 105.2 (C-2′, 6′), 97.7 (C-10), 95.9 (C-6), 94.0 (C-8), 72.6 (C-2), 71.7 (C-3), 40.0 (C-4), 31.4 (C-1‴, 2‴), 21.2 (C-3‴), 13.5 (C-4‴).

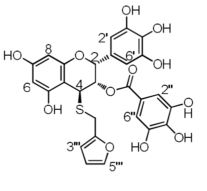

JP-25: UV λ_{max} (MeOH) nm: 278, 216; mp. 162.1~163.4^{o}C; $[\alpha]_D^{25}$ -112.3° (*c* 0.1 MeOH); HR-ESI-MS (*m/z* 571.0905 [M+H]$^+$, calcd, 571.0905); ^{1}H NMR (500 MHz, DMSO-d_6) δ: 7.60 (1H, s, 5‴-H), 6.78 (2H, s, 2″, 6″-H), 6.39 (2H, s, 2′, 6′-H), 6.38 (2H, m, 3‴, 4‴-H), 5.91 (1H, d, *J* = 1.8, 8-H), 5.82 (1H, d, *J* = 1.8, 6-H), 5.33 (1H, s, 3-H), 5.28 (1H, s, 2-H), 4.09 (1H, d, *J* = 1.2, 4-H), 4.05 (2H, m, S-C-H); ^{13}C NMR (125 MHz, DMSO-d_6) δ: 165.2 (O-C-O), 158.1 (C-5), 157.2 (C-7), 155.5 (C-9), 151.5 (C-2‴), 145.8 (C-3′, 5′), 142.4 (C-3″, 5″), 138.9 (C-5‴), 132.5 (C-4″), 127.8 (C-1′), 118.6 (C-1″), 110.6 (C-4‴), 108.7 (C-2″, 6″), 107.6 (C-2′, 6′), 105.4 (C-3‴), 96.9 (C-10), 95.9 (C-6), 94.0 (C-8), 72.7 (C-2), 71.0 (C-3), 40.0 (C-4), 27.9 (S-C).

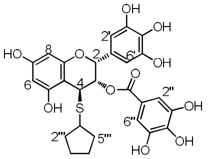

JP-26: UV λ_{max} (MeOH) nm: 278, 216; mp. 175.5~176.2^{o}C; $[\alpha]_D^{25}$ -75.5° (*c* 0.1 MeOH); HR-ESI-MS (*m/z* 559.1267 [M+H]$^+$, calcd, 559.1269); ^{1}H NMR (500 MHz, DMSO-d_6) δ: 6.77 (2H, s, 2″, 6″-H), 6.40 (2H, s, 2′, 6′-H), 5.91 (1H, d, *J* = 2.0, 8-H), 5.82 (1H, d, *J* = 2.0, 6-H), 5.33 (1H, s, 3-H), 5.24 (1H, s, 2-H), 3.99 (1H, d, *J* = 2.0, 4-H), 3.51 (1H, m, 1‴-H), 2.09 (2H, m, 2‴, 5‴-H), 1.74 (2H, m, 2‴, 5‴-H), 1.55 (4H, m, 3‴, 4‴-H); ^{13}C NMR (125 MHz, DMSO-d_6) δ: 165.2 (O-C-O), 157.7 (C-5), 157.0 (C-7), 155.0 (C-9), 145.8 (C-3′, 5′), 145.4 (C-3″, 5″), 138.8 (C-4″), 132.5 (C-4′), 127.9 (C-1′), 118.6 (C-1″), 108.7 (C-2″, 6″), 105.2 (C-2′, 6′), 97.9 (C-10), 95.9 (C-6), 94.0 (C-8), 72.7 (C-2), 71.9 (C-3), 44.3 (S-C), 40.0 (C-4), 33.8 (C-2‴), 33.5 (C-5‴), 24.6 (C-3‴), 24.2 (C-4‴).

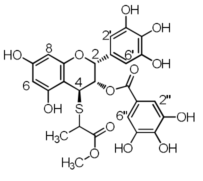

JP-27: UV λ_{max} (MeOH) nm: 278, 216; mp. 143.2~145.1°C; $[\alpha]_D^{25}$ -92.7° (*c* 0.1 MeOH); HR-ESI-MS (*m/z* 577.1024 $[M+H]^+$, calcd, 577.101); ^{1}H NMR (500 MHz, DMSO-d_6) δ: 6.77 (2H, d, *J* = 2.5, 2″, 6″-H), 6.38 (2H, d, *J* = 2.5, 2′, 6′-H), 5.92 (1H, d, *J* = 2.0, 8-H), 5.82 (1H, d, *J* = 2.0, 6-H), 5.29 (1H, s, 3-H), 5.23 (1H, s, 2-H), 4.15 (1H, s, 4-H), 3.85 (1H, m, S-C-H), 3.69 (3H, s, -OCH_3), 1.48 (3H, d, *J* = 7.0, -CH_3); ^{13}C NMR (125 MHz, DMSO-d_6) δ: 172.9 (O-C-OCH_3), 164.9 (O-C-O),158.2 (C-5), 157.2 (C-7), 155.5 (C-9), 145.8 (C-3′, 5′), 145.4 (C-3″, 5″), 138.9 (C-4″), 132.6 (C-4′), 127.9 (C-1′), 118.6 (C-1″), 108.7 (C-2″, 6″), 105.3 (C-2′, 6′), 96.5 (C-10), 95.9 (C-6), 94.0 (C-8), 72.8 (C-2), 71.5 (C-3), 52.0 (C-OCH_3), 40.8 (S-C), 40.0 (C-4), 17.5 (C-CH_3).

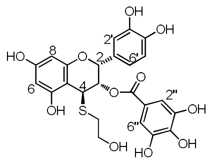

JP-28: UV λ_{max} (MeOH) nm: 278, 216; mp. 140.4~142.8°C; HR-ESI-MS (*m/z* 519.0963 $[M+H]^+$, calcd, 519.0956); ^{1}H NMR (500 MHz, DMSO-d_6) δ: 6.86 (1H, d, *J* = 2.0, 2′-H), 6.78 (2H, s, 2″, 6″-H), 6.77 (1H, dd, *J* = 8.0, 2.0, 6′-H), 6.69 (1H, d, *J* = 8.0, 5′-H), 5.94 (1H, d, *J* = 2.5, 8-H), 5.83 (1H, d, *J* = 2.5, 6-H), 5.40 (1H, brs, 3-H), 5.18 (1H, brs, 2-H), 3.98 (1H, d, *J* = 2.0, 4-H), 3.70 (2H, t, -CH_2-OH), 2.89 (2H, m, S-CH_2); ^{13}C NMR (125 MHz, DMSO-d_6) δ: 165.2 (O-C-O), 157.9 (C-5), 157.2 (C-7), 155.1 (C-9), 145.4 (C-3″, 5″), 144.9 (C-3′, 4′), 138.9 (C-4″), 128.6 (C-1′), 118.5 (C-1″), 117.5 (C-6′), 115.2 (C-5′), 114.1 (C-2′), 108.6 (C-2″, 6″), 97.7 (C-10), 96.0 (C-6), 94.1 (C-8), 72.6 (C-2), 72.1 (C-3), 61.0 (C-OH), 41.1 (C-4), 34.8 (S-C).

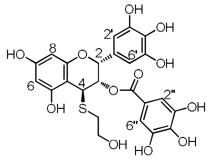

JP-29: UV λ_{max} (MeOH) nm: 278, 216; mp. 142.1~143.5°C; HR-ESI-MS (*m/z* 535.0913 $[M+H]^+$, calcd, 535.0905); ^{1}H NMR (500 MHz, DMSO-d_6) δ: 6.77 (2H, s, 2″, 6″-H), 6.41 (2H, s, 2′, 6′-H), 5.93 (1H, s, 8-H), 5.83 (1H, s, 6-H), 5.31 (1H, s, 3-H), 5.18 (1H, s, 2-H), 3.93 (1H, s, 4-H), 3.70 (2H, m, C-OH), 2.87 (2H, m, S-CH_2); ^{13}C

NMR (125 MHz, DMSO-d_6) δ: 165.2 (O-C-O), 157.9 (C-5), 157.2 (C-7), 155.1 (C-9), 145.8 (C-3′, 5′), 145.4 (C-3″, 5″), 138.9 (C-4″), 132.5 (C-4′), 127.8 (C-1′), 118.6 (C-1″), 108.7 (C-2″, 6″), 105.3 (C-2′, 6′), 97.7 (C-10), 96.0 (C-6), 94.1 (C-8), 72.6 (C-2), 72.0 (C-3), 61.1 (C-OH), 41.1 (C-4), 34.7 (S-C).

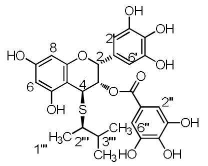

JP-30: $[\alpha]_D^{25}$ -65.2° (*c* 0.1 MeOH); HR-ESI-MS (*m/z* 561.1442 $[M+H]^+$, calcd, 561.1425); ^{1}H NMR (500 MHz, DMSO-d_6) δ: 6.74 (2H, s, 2″, 6″-H), 6.37 (2H, s, 2′, 6′-H), 5.89 (1H, d, J = 2.0, 8-H), 5.80 (1H, d, J = 2.0, 6-H), 5.35 (1H, s, 3-H), 5.12 (1H, s, 2-H), 3.91 (1H, d, J = 2.0, 4-H), 3.07 (1H, m, 2‴-H), 1.87 (1H, m, 3‴-H), 1.38 (3H, d, J = 3.0, 1‴-H), 1.01 (3H, d, J = 6.5, 3‴-CH_3), 0.93 (3H, d, J = 6.5, 3‴-CH_3); ^{13}C NMR (125 MHz, DMSO-d_6) δ: 165.4 (O-C-O), 157.9 (C-5), 157.2 (C-7), 155.3 (C-9), 145.9 (C-3′, 5′), 145.5 (C-3″, 5″), 139.0 (C-4″), 132.6 (C-4′), 128.1 (C-1′), 118.5 (C-1″), 108.8 (C-2″, 6″), 105.3 (C-2′, 6′), 97.9 (C-10), 96.0 (C-6), 94.1 (C-8), 72.8 (C-2), 72.0 (C-3), 47.9 (C-2‴), 40.4 (C-4), 32.8 (C-3‴), 19.7 (C-1‴), 19.3 (C-3‴-CH_3), 19.1 (C-3‴-CH_3).

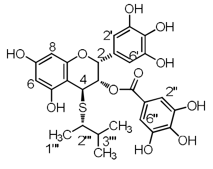

JP-31: $[\alpha]_D^{25}$ -49.1° (*c* 0.1 MeOH); HR-ESI-MS (*m/z* 561.143 $[M+H]^+$, calcd, 561.1425); ^{1}H NMR (500 MHz, DMSO-d_6) δ: 6.75 (2H, s, 2″, 6″-H), 6.39 (2H, s, 2′, 6′-H), 5.90 (1H, d, J = 2.0, 8-H), 5.80 (1H, d, J = 2.0, 6-H), 5.37 (1H, s, 3-H), 5.15 (1H, s, 2-H), 3.97 (1H, d, J = 2.0, 4-H), 3.15 (1H, m, 2‴-H), 2.00 (1H, m, 3‴-H), 1.30 (3H, d, J = 3.0, 1‴-H), 1.00 (3H, d, J = 6.5, 3‴-CH_3), 0.96 (3H, d, J = 6.5, 3‴-CH_3); ^{13}C NMR (125 MHz, DMSO-d_6) δ: 165.4 (O-C-O), 157.9 (C-5), 157.3 (C-7), 155.3 (C-9), 145.9 (C-3′, 5′), 145.5 (C-3″, 5″), 139.0 (C-4″), 132.6 (C-4′), 128.1 (C-1′), 118.7 (C-1″), 108.8 (C-2″, 6″), 105.3 (C-2′, 6′), 98.0 (C-10), 96.0 (C-6), 94.1 (C-8),

72.6 (C-2), 72.4 (C-3), 47.7 (C-2‴), 40.4 (C-4), 32.8 (C-3‴), 19.3 (C-1‴), 19.0 (C-3‴-CH_3), 18.6 (C-3‴-CH_3).

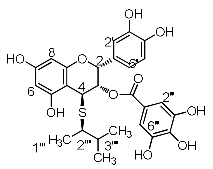

JP-32: UV λ_{max} (MeOH) nm: 278, 216; mp. 144.4~146.0°C; $[\alpha]_D^{25}$ -90.7° (*c* 0.1 MeOH); HR-ESI-MS (*m/z* 545.1476 $[M+H]^+$, calcd, 545.1476); ^1H NMR (500 MHz, DMSO-d_6) δ: 6.84 (1H, d, *J* = 2.0, 2′-H), 6.77 (2H, s, 2″, 6″-H), 6.75 (1H, dd, *J* = 8.0, 2.0, 6′-H), 6.74 (1H, dd, *J* = 8.0, 5′-H), 5.93 (1H, d, *J* = 2.0, 8-H), 5.83 (1H, d, *J* = 2.0, 6-H), 5.46 (1H, s, 3-H), 5.12 (1H, s, 2-H), 3.97 (1H, d, *J* = 2.0, 4-H), 3.09 (1H, m, 2‴-H), 1.89 (1H, m, 3‴-H), 1.41 (3H, d, *J* = 7.0, 1‴-H), 1.03 (3H, d, *J* = 6.5, 3‴-CH_3), 0.96 (3H, d, *J* = 6.5, 3‴-CH_3); ^{13}C NMR (125 MHz, DMSO-d_6) δ: 165.2 (O-C-O), 157.8 (C-5), 157.1 (C-7), 155.2 (C-9), 145.4 (C-3″, 5″), 144.9 (C-3′, 4′), 138.9 (C-4″), 128.7 (C-1′), 118.5 (C-1″), 117.4 (C-6′), 115.3 (C-5′), 114.0 (C-2′), 108.6 (C-2″, 6″), 97.7 (C-10), 95.9 (C-6), 94.0 (C-8), 72.8 (C-2), 72.0 (C-3), 47.7 (C-2‴), 38.1 (C-4), 32.6 (C-3‴), 19.5 (C-1‴), 19.2 (C-3‴-CH_3), 19.0 (C-3‴-CH_3).

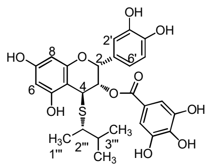

JP-33: UV λ_{max} (MeOH) nm: 278, 216; mp. 143.0~144.9°C; $[\alpha]_D^{25}$ -102.3° (*c* 0.1 MeOH); HR-ESI-MS (*m/z* 545.1486 $[M+H]^+$, calcd, 545.1476); ^1H NMR (500 MHz, DMSO-d_6) δ: 6.82 (1H, d, *J* = 2.0, 2′-H), 6.74 (2H, s, 2″, 6″-H), 6.72 (1H, dd, *J* = 8.0, 2.0, 6′-H), 6.66 (1H, dd, *J* = 8.0, 5′-H), 5.89 (1H, d, *J* = 2.0, 8-H), 5.79 (1H, d, *J* = 2.0, 6-H), 5.43 (1H, s, 3-H), 5.11 (1H, s, 2-H), 3.98 (1H, d, *J* = 2.0, 4-H), 3.14 (1H, m, 2‴-H), 2.00 (1H, m, 3‴-H), 1.28 (3H, d, *J* = 6.5, 1‴-H), 1.00 (3H, d, *J* = 6.5, 3‴-CH_3), 0.94 (3H, d, *J* = 6.5, 3‴-CH_3); ^{13}C NMR (125 MHz, DMSO-d_6) δ: 165.3 (O-C-O), 157.8 (C-5), 157.3 (C-7), 155.2 (C-9), 145.5 (C-3″, 5″), 145.0 (C-3′), 144.9 (C-4′), 138.9 (C-4″), 128.7 (C-1′), 118.5 (C-1″), 117.4 (C-6′), 115.3 (C-5′), 114.0

(C-2′), 108.7 (C-2″, 6″), 97.8 (C-10), 96.0 (C-6), 94.0 (C-8), 72.6 (C-2), 72.4 (C-3), 47.5 (C-2‴), 40.4 (C-4), 32.7 (C-3‴), 19.3 (C-1‴), 18.9 (C-3‴-CH_3), 18.5 (C-3‴-CH_3).

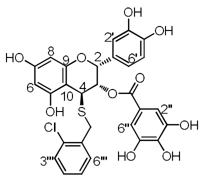

JP-34: UV λ_{max} (MeOH) nm: 278, 216; mp. 145.8~147.9°C; $[\alpha]^{25}_{D}$ -99.3° (*c* 0.1 MeOH); HR-ESI-MS (*m/z* 599.0791 $[M+H]^+$, calcd, 599.0773); ^{1}H NMR (500 MHz, DMSO-d_6) δ: 7.59 (1H, m, 3‴-H), 7.48 (1H, m, 5‴-H), 7.31 (2H, m, 4‴, 6‴-H), 6.83 (1H, s, 2′-H), 6.80 (2H, s, 2″, 6″-H), 6.68 (2H, m, 5′, 6′-H), 5.91 (1H, d, *J* = 2.0, 8-H), 5.82 (1H, d, *J* = 2.0, 6-H), 5.44 (1H, s, 3-H), 5.30 (1H, s, 2-H), 4.15 (2H, m, S-CH_2), 4.13 (1H, s, 4-H); ^{13}C NMR (125 MHz, DMSO-d_6) δ: 165.2 (O-C-O), 158.1 (C-5), 157.2 (C-7), 155.5 (C-9), 145.5 (C-3″, 5″), 144.9 (C-3′), 138.9 (C-4′), 136.0 (C-4″), 133.2 (C-6‴, 1′), 131.1 (C-2‴), 129.5 (C-1‴), 128.9 (C-5‴), 128.5 (C-3‴), 127.2 (C-4‴), 118.5 (C-1″), 117.5 (C-6′), 115.2 (C-5′), 114.2 (C-2′), 108.6 (C-2″, 6″), 96.8 (C-10), 95.9 (C-6), 94.0 (C-8), 72.8 (C-2), 71.2 (C-3), 33.7 (C-4), 30.7 (S-C).

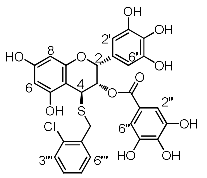

JP-35: UV λ_{max} (MeOH) nm: 278, 216; mp. 149.1~151.3°C; $[\alpha]^{25}_{D}$ -107.2° (*c* 0.1 MeOH); HR-ESI-MS (*m/z* 615.0736 $[M+H]^+$, calcd, 615.0722); ^{1}H NMR (500 MHz, DMSO-d_6) δ: 7.59 (1H, m, 3‴-H), 7.47 (1H, m, 5‴-H), 7.31 (2H, m, 4‴, 6‴-H), 6.79 (2H, s, 2″, 6″-H), 6.39 (2H, s, 2′, 6′-H), 5.90 (1H, d, *J* = 2.0, 8-H), 5.82 (1H, d, *J* = 2.0, 6-H), 5.36 (1H, s, 3-H), 5.33 (1H, s, 2-H), 4.14 (2H, m, S-CH_2), 4.10 (1H, s, 4-H); ^{13}C NMR (125 MHz, DMSO-d_6) δ: 165.3 (O-C-O), 158.1 (C-5), 157.3 (C-7), 155.5 (C-9), 145.8 (C-3′, 5′), 145.4 (C-3″, 5″), 138.9 (C-4″), 135.9 (C-4′), 133.2 (C-6‴), 132.6 (C-1′), 131.1 (C-2‴), 129.5 (C-1‴), 128.9 (C-5‴), 127.8 (C-3‴), 127.2 (C-4‴), 118.6 (C-1″), 108.6 (C-2″, 6″), 105.4 (C-2′, 6′), 96.8 (C-10), 95.9 (C-6), 94.0

(C-8), 72.8 (C-2), 71.2 (C-3), 33.7 (C-4), 30.7 (S-C).

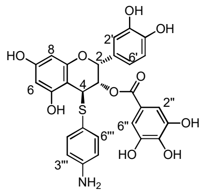

JP-36: UV λ_{max} (MeOH) nm: 278, 216; mp. 143.7~146.4°C; $[\alpha]_D^{25}$ -39.8° (*c* 0.1 MeOH); HR-ESI-MS (*m/z* 566.1132 $[M+H]^+$, calcd, 566.1115); ^1H NMR (500 MHz, DMSO-d_6) δ: 7.28 (2H, d, *J* = 8.0, 2‴, 6‴-H), 6.82 (1H, s, 2′-H), 6.71 (1H, d, *J* = 8.5, 6′-H), 6.68 (2H, s, 2″, 6″-H), 6.65 (2H, d, *J* = 8.5, 5′-H), 6.60 (2H, d, *J* = 8.0, 3‴, 5‴-H), 5.91 (1H, d, *J* = 2.0, 8-H), 5.83 (1H, d, *J* = 2.0, 6-H), 5.57 (1H, s, 3-H), 5.15 (1H, s, 2-H), 4.17 (1H, s, 4-H); ^{13}C NMR (125 MHz, DMSO-d_6) δ: 164.5 (O-C-O), 158.0 (C-5), 157.3 (C-7), 155.5 (C-9), 149.3 (C-4‴), 145.3 (C-3″, 5″), 144.9 (C-3′), 144.8 (C-4′), 138.7 (C-4″), 135.5 (C-2‴, 6‴), 128.7 (C-1′), 118.6 (C-1‴), 117.4 (C-1″), 117.2 (C-6′), 115.2 (C-5′), 114.4 (C-3‴, 5‴), 114.0 (C-2′), 108.5 (C-2″, 6″), 97.1 (C-10), 95.9 (C-6), 94.1 (C-8), 72.5 (C-2), 70.6 (C-3), 40.4 (C-4).

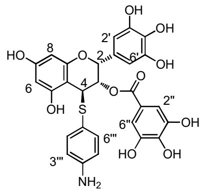

JP-37: UV λ_{max} (MeOH) nm: 278, 216; mp. 146.8~148.5°C; $[\alpha]_D^{25}$ -46.8° (*c* 0.1 MeOH); HR-ESI-MS (*m/z* 582.1072$[M+H]^+$, calcd, 582.1065); ^1H NMR (500 MHz, DMSO-d_6) δ: 7.25 (2H, d, *J* = 8.0, 2‴, 6‴-H), 6.67 (2H, s, 2″, 6″-H), 6.58 (2H, d, *J* = 8.0, 3‴, 5‴-H), 6.37 (2H, s, 2′, 6′-H), 5.96 (1H, d, *J* = 2.0, 8-H), 5.83 (1H, d, *J* = 2.0, 6-H), 5.50 (1H, s, 3-H), 5.19 (1H, s, 2-H), 4.14 (1H, s, 4-H); ^{13}C NMR (125 MHz, DMSO-d_6) δ: 164.5 (O-C-O), 158.0 (C-5), 157.3 (C-7), 155.5 (C-9), 149.3 (C-4‴), 145.8 (C-3′, 5′), 145.3 (C-3″, 5″), 138.7 (C-4″), 135.4 (C-2‴, 6‴), 132.4 (C-4′), 128.0 (C-1′), 118.7 (C-1‴), 117.3 (C-1″), 114.4 (C-3‴, 5‴), 108.6 (C-2″, 6″), 105.2 (C-2′, 6′), 97.2 (C-10), 95.9 (C-6), 94.1 (C-8), 72.5 (C-2), 70.5 (C-3), 44.0 (C-4).

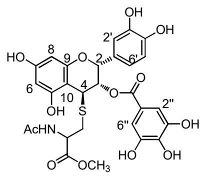

JP-38: UV λ_{max} (MeOH) nm: 278, 216; mp. 158.9~161.3°C; $[\alpha]_D^{25}$ -77.0° (*c* 0.1 MeOH); HR-ESI-MS (*m/z* 618.1277 $[M+H]^+$, calcd, 618.1276); ^{1}H NMR (500 MHz, DMSO-*d6*) δ: 6.88 (1H, d, *J* = 2.0, 2′-H), 6.78 (3H, m, 2″, 6″, 6′-H), 6.69 (1H, d, *J* = 8.0, 5′-H), 5.97 (1H, d, *J* = 1.5, 8-H), 5.83 (1H, d, *J* = 1.5, 6-H), 5.37 (1H, s, 3-H), 5.17 (1H, s, 2-H), 4.68 (1H, m, -N-CH), 4.03 (1H, s, 4-H), 3.67 (3H, s, $-OCH_3$), 3.15 (2H, m, $-S-CH_2$), 1.88 (3H, s, $-C-CH_3$); ^{13}C NMR (125 MHz, DMSO-*d6*) δ: 171.2 (N-C-O), 169.3 ($-COO-CH_3$), 164.1 (O-C-O), 158.1 (C-5), 157.2 (C-7), 155.2 (C-9), 145.4 (C-3″, 5″), 144.9 (C-3′, 4′), 138.9 (C-4″), 128.4 (C-1′), 118.4 (C-1″), 117.6 (C-6′), 115.2 (C-5′), 114.3 (C-2′), 108.6 (C-2″, 6″), 96.9 (C-10), 96.0 (C-6), 94.1 (C-8), 72.6 (C-2), 71.5 (C-3), 52.5 (N-CH), 52.1 ($-OCH_3$), 40.4 (C-4), 33.1 (S-C), 22.3 ($O=C-CH_3$).

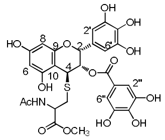

JP-39: UV λ_{max} (MeOH) nm: 278, 216; mp. 166.0~167.3°C; $[\alpha]_D^{25}$ -89° (*c* 0.1 MeOH); HR-ESI-MS (*m/z* 634.1253 $[M+H]^+$, calcd, 634.1225); ^{1}H NMR (500 MHz, DMSO-*d6*) δ: 6.78 (2H, s, 2″, 6″-H), 6.43 (2H, s, 2′, 6′-H), 5.94 (1H, d, *J* = 2.0, 8-H), 5.83 (1H, d, *J* = 2.0, 6-H), 5.28 (1H, s, 3-H), 5.19 (1H, s, 2-H), 4.69 (1H, m, -N-CH), 4.00 (1H, s, 4-H), 3.67 (3H, s, $-OCH_3$), 3.15 (2H, m, $-N-CH_2$), 1.88 (3H, s, $-C-CH_3$); ^{13}C NMR (125 MHz, DMSO-*d6*) δ: 171.3 (N-C-O), 169.4 ($-COO-CH_3$), 165.2 (O-C-O), 158.2 (C-5), 157.2 (C-7), 155.3 (C-9), 145.8 (C-3′, 5′), 145.5 (C-3″, 5″), 138.9 (C-4″), 132.6 (C-4′), 127.7 (C-1′), 118.6 (C-1″), 108.7 (C-2″, 6″), 105.5 (C-2′, 6′), 97.1 (C-10), 96.0 (C-6), 94.2 (C-8), 72.6 (C-2), 71.5 (C-3), 52.5 (N-CH), 52.2 ($-OCH_3$), 40.4 (C-4), 33.2 (S-C), 22.4 ($O=C-CH_3$).

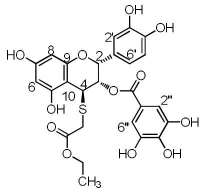

JP-40: UV λ_{max} (MeOH) nm: 278, 216; mp. 142.2~143.4°C; $[\alpha]_D^{25}$ -119.8° (*c* 0.1 MeOH); HR-ESI-MS (*m/z* 561.1064 $[M+H]^+$, calcd, 561.1061); ^{1}H NMR (500 MHz, DMSO-d_6) δ: 6.87 (1H, s, 2′-H), 6.77 (2H, s, 2″, 6″-H), 6.76 (1H, dd, *J* = 8.0, 1.5, 6′-H), 6.69 (1H, d, *J* = 8.0, 5′-H), 5.95 (1H, d, *J* = 2.5, 8-H), 5.84 (1H, d, *J* = 2.5, 6-H), 5.44 (1H, s, 3-H), 5.27 (1H, s, 2-H), 4.14 (2H, m, S-C-H), 4.11 (1H, brs, 4-H), 3.64 (3H, m, -O-CH_2), 1.20 (3H, t, H-CH_3); ^{13}C NMR (125 MHz, DMSO-d_6) δ: 170.2 (O-C-OCH_2), 164.9 (O-C-O), 158.3 (C-5), 157.4 (C-7), 155.4 (C-9), 145.4 (C-3″, 5″), 144.9 (C-3′, 4′), 138.8 (C-4″), 129.7 (C-1′), 118.5 (C-1″), 117.6 (C-6′), 115.2 (C-5′), 114.2 (C-2′), 109.1 (C-2″, 6″), 96.6 (C-10), 95.9 (C-6), 94.1 (C-8), 72.6 (C-2), 70.9 (C-3), 61.0 (C-OCH_2), 33.9 (C-4), 33.5 (S-C), 14.0 (C-CH_3).

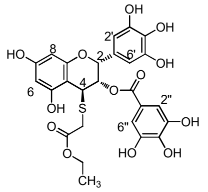

JP-41: UV λ_{max} (MeOH) nm: 278, 216; mp. 147.8~149.2°C; $[\alpha]_D^{25}$ -120.8° (*c* 0.1 MeOH); HR-ESI-MS (*m/z* 577.1018 $[M+H]^+$, calcd, 577.101); ^{1}H NMR (500 MHz, DMSO-d_6) δ: 6.74 (1H, s, 2″, 6″-H), 6.39 (2H, s, 2′, 6′-H), 5.92 (1H, d, *J* = 2.5, 8-H), 5.81 (1H, d, *J* = 2.5, 6-H), 5.32 (1H, s, 3-H), 5.25 (1H, s, 2-H), 4.11 (2H, m, S-C-H), 4.09(1H, brs, 4-H), 3.60 (3H, m, -O-CH_2), 1.17 (3H, t, H-CH_3); ^{13}C NMR (125 MHz, DMSO-d_6) δ: 170.1 (O-C-OCH_2), 165.0 (O-C-O), 158.3 (C-5), 157.4 (C-7), 155.5 (C-9), 145.8 (C-3′, 5′), 145.5 (C-3″, 5″), 138.9 (C-4″), 132.6 (C-4′), 127.8 (C-1′), 118.7 (C-1″), 108.7 (C-2″, 6″), 105.5 (C-2′, 6′), 96.8 (C-10), 96.0 (C-6), 94.1 (C-8), 72.7 (C-2), 70.9 (C-3), 61.0 (C-OCH_2), 40.3 (C-4), 33.9 (S-C), 14.0 (C-CH_3).

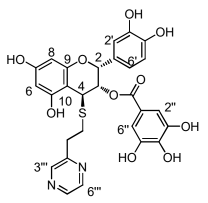

JP-42: UV λ_{max} (MeOH) nm: 278, 216; mp. 158.9~161.3°C; $[\alpha]_D^{25}$ -93.6° (*c* 0.1 MeOH); HR-ESI-MS (*m/z* 581.1232 $[M+H]^+$, calcd, 581.1224); ^1H NMR (500 MHz, DMSO-d_6) δ: 8.67 (1H, s, 6‴-H), 8.59 (1H, s, 3‴-H), 8.51 (1H, d, *J* = 2.0, 5‴-H), 6.85 (1H, d, *J* = 1.5, 2′-H), 6.80 (2H, s, 2″, 6″-H), 6.74 (1H, dd, *J* = 8.0, 1.5, 6′-H), 6.71 (1H, d, *J* = 8.0, 5′-H), 5.95 (1H, d, *J* = 2.0, 8-H), 5.83 (1H, d, *J* = 2.0, 6-H), 5.36 (1H, s, 3-H), 5.21 (1H, s, 2-H), 4.10 (1H, d, *J* = 1.5, 4-H), 3.25 (2H, m, 2‴-CH_2), 3.18 (2H, m, S-CH_2); ^{13}C NMR (125 MHz, DMSO-d_6) δ: 165.3 (O-C-O), 158.0 (C-5), 157.2 (C-7), 155.5 (C-9), 155.2 (C-2‴), 145.4 (C-3″, 5″), 144.9 (C-3′, 4′, 3‴, 6‴), 144.1 (C-5‴), 142.6 (C-4″), 128.3 (C-1′), 118.4 (C-1″), 117.5 (C-6′), 115.2 (C-5′), 114.1 (C-2′), 108.6 (C-2″, 6″), 97.3 (C-10), 95.9 (C-6), 94.0 (C-8), 72.7 (C-2), 71.8 (C-3), 40.0 (C-4), 34.7 (2‴-CH_2), 30.1 (S-C).

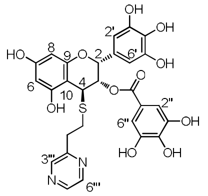

JP-43: UV λ_{max} (MeOH) nm: 278, 216; mp. 166.0~167.3°C; $[\alpha]_D^{25}$ -91.4° (*c* 0.1 MeOH); HR-ESI-MS (*m/z* 597.1171 $[M+H]^+$, calcd, 597.1174); ^1H NMR (500 MHz, DMSO-d_6) δ: 8.67 (1H, s, 6‴-H), 8.59 (1H, s, 3‴-H), 8.51 (1H, d, *J* = 2.0, 5‴-H), 6.79 (2H, s, 2″, 6″-H), 6.40 (2H, s, 2′, 6′-H), 5.94 (1H, d, *J* = 2.0, 8-H), 5.83 (1H, d, *J* = 2.0, 6-H), 5.29 (1H, s, 3-H), 5.24 (1H, s, 2-H), 4.07 (1H, d, *J* = 1.5, 4-H), 3.25 (2H, m, 2‴-CH_2), 3.18 (2H, m, S-C-H); ^{13}C NMR (125 MHz, DMSO-d_6) δ: 165.3 (O-C-O), 158.0 (C-5), 157.2 (C-7), 155.4 (C-9), 155.2 (C-2‴), 145.8 (C-3′, 5′), 145.4 (C-3″, 5″), 144.9 (C-3‴), 144.1 (C-6‴), 142.6 (C-5‴), 138.9 (C-4″), 132.3 (C-4′), 127.8 (C-1′), 118.6 (C-1″), 108.7 (C-2″, 6″), 105.3 (C-2′, 6′), 97.4 (C-10), 95.9 (C-6), 94.0 (C-8), 72.7 (C-2), 71.7 (C-3), 40.0 (C-4), 34.7 (2‴-CH_2), 30.1 (S-C).

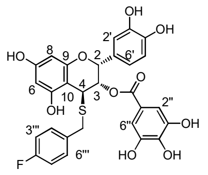

JP-44: UV λ_{max} (MeOH) nm: 278, 216; mp. 143.2~145.3°C; $[\alpha]_D^{25}$ -101.1° (*c* 0.1 MeOH); HR-ESI-MS (*m/z* 583.1065 $[M+H]^+$, calcd, 583.1069); ^{1}H NMR (500 MHz, DMSO-d_6) δ: 7.49 (2H, m, 2‴, 6‴H), 7.48 (2H, m, 3‴, 5‴H), 7.14 (1H, s, 2′-H), 6.83 (2H, s, 2″, 6″-H), 6.67 (2H, brs, 5′, 6′-H), 5.90 (1H, d, *J* = 2.0, 8-H), 5.81 (1H, d, *J* = 2.0, 6-H), 5.42 (1H, s, 3-H), 5.21 (1H, s, 2-H), 4.05 (2H, m, S-C-H), 4.01 (1H, d, *J* = 2.0, 4-H); ^{13}C NMR (125 MHz, DMSO-d_6) δ: 165.1 (O-C-O), 160.2 (C-4‴), 158.0 (C-5), 157.2 (C-7), 155.4 (C-9), 145.4 (C-3″, 5″), 144.9 (C-3′, 4′), 138.9 (C-4″), 135.0 (C-1‴), 130.8 (C-2‴, 6‴), 128.5 (C-1′), 118.4 (C-1″), 117.5 (C-6′), 115.2 (C-3‴, 5‴), 115.1 (C-5′), 114.0 (C-2′), 108.6 (C-2″, 6″), 97.0 (C-10), 95.9 (C-6), 94.0 (C-8), 72.7 (C-2), 71.2 (C-3), 40.0 (C-4), 34.9 (S-C).

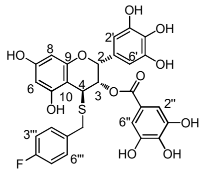

JP-45: UV λ_{max} (MeOH) nm: 278, 216; mp. 144.6~147.1°C; $[\alpha]_D^{25}$ -95.2° (*c* 0.1 MeOH); HR-ESI-MS (*m/z* 599.1012 $[M+H]^+$, calcd, 599.1018); ^{1}H NMR (500 MHz, DMSO-d_6) δ: 7.48 (2H, t, 2‴, 6‴-H), 7.14 (2H, t, *J* = 8.5, 3‴, 5‴-H), 6.78 (2H, s, 2″, 6″-H), 6.39 (1H, s, 2′, 6′-H), 5.90 (1H, d, *J* = 2.0, 8-H), 5.82 (1H, d, *J* = 2.0, 6-H), 5.35 (1H, s, 3-H), 5.25 (1H, brs, 2-H), 4.06 (2H, m, S-CH_2), 4.02(1H, m, 4-H); ^{13}C NMR (125 MHz, DMSO-d_6) δ: 165.2 (O-C-O), 162.1 (C-4‴), 160.2 (C-5), 158.0 (C-7), 157.2 (C-9), 145.8 (C-3′, 5′), 145.4 (C-3″, 5″), 138.9 (C-4″), 134.8 (C-1‴), 132.5 (C-4′), 130.8 (C-2‴, 6‴), 127.8 (C-1′), 118.6 (C-1″), 115.1 (C-3‴, 5‴), 108.7 (C-2″, 6″), 105.4 (C-2′, 6′), 97.1 (C-10), 95.9 (C-6), 94.0 (C-8), 72.8 (C-2), 71.2 (C-3), 40.0 (C-4), 34.9 (C-CH_2).

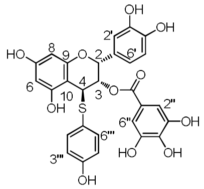

JP-46: UV λ_{max} (MeOH) nm: 278, 216; mp. 153.1~155.6°C; $[\alpha]_D^{25}$ -29.1° (*c* 0.1 MeOH); HR-ESI-MS (*m/z* 567.0949 $[M+H]^+$, calcd, 567.0956); ^{1}H NMR (500 MHz, DMSO-d_6) δ: 7.45 (2H, d, *J* = 8.5, 2‴, 6‴-H), 6.82 (1H, d, *J* = 1.5, 2′-H), 6.80 (2H, d, *J* = 8.5, 3‴, 5‴-H), 6.73 (1H, dd, *J* = 8.0, 1.5, 6′-H), 6.70 (2H, s, 2″, 6″-H), 6.67 (1H, d, *J* = 8.0, 5′-H), 5.98 (1H, d, *J* = 2.0, 8-H), 5.85 (1H, d, *J* = 2.0, 6-H), 5.49 (1H, s, 3-H), 5.12 (1H, brs, 2-H), 4.27 (1H, d, *J* = 2.0, 4-H); ^{13}C NMR (125 MHz, DMSO-d_6) δ: 164.6 (O-C-O), 158.2 (C-5), 157.7 (C-7), 157.3 (C-9), 155.5 (C-4‴), 145.4 (C-3″, 5″), 144.9 (C-3′, 4′), 138.7 (C-4″), 135.2 (C-2‴, 6‴), 128.6 (C-1′), 122.1 (C-1‴), 118.5 (C-1″), 117.4 (C-6′), 116.2 (C-3‴, 5‴), 115.2 (C-5′), 114.0 (C-2′), 108.6 (C-2″, 6″), 96.8 (C-10), 96.0 (C-6), 94.1 (C-8), 72.6 (C-2), 70.5 (C-3), 43.5 (C-4).

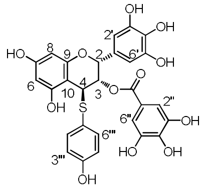

JP-47: UV λ_{max} (MeOH) nm: 278, 216; mp. 151.3~153.5°C; $[\alpha]_D^{25}$ -49.3° (*c* 0.1 MeOH); HR-ESI-MS (*m/z* 583.0884 $[M+H]^+$, calcd, 583.0905); ^{1}H NMR (500 MHz, DMSO-d_6) δ: 7.44 (2H, d, *J* = 8.5, 2‴, 6‴-H), 6.82 (2H, d, *J* = 8.5, 3‴, 5‴-H), 6.67 (2H, s, 2″, 6″-H), 6.37 (1H, s, 2′, 6′-H), 5.97 (1H, d, *J* = 2.0, 8-H), 5.85 (1H, d, *J* = 2.0, 6-H), 5.49 (1H, s, 3-H), 5.16 (1H, brs, 2-H), 4.24 (1H, d, *J* = 2.0, 4-H); ^{13}C NMR (125 MHz, DMSO-d_6) δ: 164.6 (O-C-O), 158.2 (C-5), 157.7 (C-7), 157.3 (C-9), 155.5 (C-4‴), 145.8 (C-3′, 5′), 145.3 (C-3″, 5″), 138.7 (C-4″), 135.0 (C-2‴, 6‴), 132.5 (C-4′), 127.9 (C-1′), 122.1 (C-1‴), 118.6 (C-1″), 116.3 (C-3‴, 5‴), 108.6 (C-2″, 6″), 105.2 (C-2′, 6′), 96.8 (C-10), 95.9 (C-6), 94.1 (C-8), 72.5 (C-2), 70.5 (C-3), 43.7 (C-4).

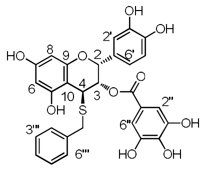

JP-48: UV λ_{max} (MeOH) nm: 278, 216; mp. 135.6~138.1^{o}C; ESI-MS: (*m/z* 563.1 $[M-H]^-$, 599.0 $[M+Cl]^-$); ^{1}H NMR (400 MHz, DMSO-d_6) δ: 7.45 (2H, m, 2‴, 6‴-H), 7.33 (2H, t, 3‴, 5‴-H), 7.23 (1H, t, 4‴-H), 6.83 (1H, brs, 2′-H), 6.78 (2H, s, 2″, 6″-H), 6.67 (2H, s, 5′, 6′-H), 5.92 (1H, d, *J* = 2.0, 8-H), 5.82 (1H, d, *J* = 2.0, 6-H), 5.43 (1H, brs, 3-H), 5.25 (1H, brs, 2-H), 4.07(1H, d, *J* = 2.0, 4-H), 4.05 (1H, dd, *J* = 27.0, 13.0, S-C-H).

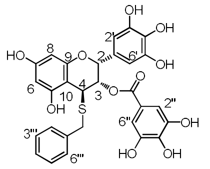

JP-49: UV λ_{max} (MeOH) nm: 278, 216; mp. 146.3~147.4^{o}C; ESI-MS: (*m/z* 579.2 $[M-H]^-$, 615.0 $[M+Cl]^-$); ^{1}H NMR (400 MHz, DMSO-d_6) δ: 7.44 (2H, m, 2‴, 6‴-H), 7.33 (2H, t, 3‴, 5‴-H), 7.24 (1H, t, 4‴-H), 6.78 (2H, s, 2″, 6″-H), 6.38 (2H, s, 2′, 6′-H), 5.92 (1H, d, *J* = 2.0, 8-H), 5.82 (1H, d, *J* = 2.0, 6-H), 5.36(1H, brs, 3-H), 5.29 (1H, brs, 2-H), 4.05 (1H, dd, *J* = 27.0, 13.0, S-C-H), 4.04(1H, d, *J* = 2.0, 4-H).

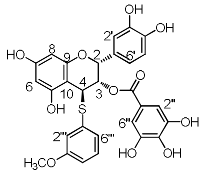

JP-50: UV λ_{max} (MeOH) nm: 278, 216; mp. 140.5~142.6^{o}C; $[\alpha]_D^{25}$ -27.6° (*c* 0.1 MeOH); HR-ESI-MS (*m/z* 581.1123 $[M+H]^+$, calcd, 581.1112); ^{1}H NMR (500 MHz, DMSO-d_6) δ: 7.31(1H, m, 5‴-H), 7.19 (1H, d, *J* = 2.5, 2‴-H), 7.14 (1H, dd, *J* = 9.0, 2.5, 6‴-H), 6.86 (1H, dd, *J* = 9.0, 2.5, 4‴-H), 6.79 (1H, d, *J* = 2.5, 2′-H), 6.76 (2H, s, 2″, 6″-H), 6.71 (1H, dd, *J* = 9.0, 2.5, 6′-H), 6.65 (1H, d, *J* = 9.0, 5′-H), 5.99 (1H, d, *J* = 2.5, 8-H), 5.88 (1H, d, *J* = 2.5, 6-H), 5.54 (1H, d, *J* = 2.5, 3-H), 5.18 (1H, d, *J* = 2.5, 2-H), 4.59 (1H, d, *J* = 2.5, 4-H), 3.81 (3H, s, -OCH_3); ^{13}C NMR (125 MHz, DMSO-d_6) δ: 164.9 (O-C-O), 159.7 (C-3‴), 158.4 (C-5), 157.3 (C-7), 155.6 (C-9), 145.4 (C-3″,

5″), 145.0 (C-3′), 144.9 (C-4′), 138.9 (C-4″), 136.2 (C-1‴), 130.0 (C-1′), 128.4 (C-5‴), 121.7 (C-6‴), 118.4 (C-1″), 117.5 (C-6′), 115.3 (C-5′), 114.4 (C-2′), 114.0 (C-2‴), 112.9 (C-4‴), 108.7 (C-2″, 6″), 96.0 (C-10), 95.9 (C-6), 94.2 (C-8), 72.8 (C-2), 70.9 (C-3), 55.2 (C-OCH_3), 41.2 (C-4).

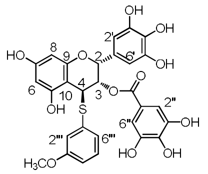

JP-51: UV λ_{max} (MeOH) nm: 278, 216; mp. 149.6~152.3°C; $[\alpha]_D^{25}$ -32.3° (*c* 0.1 MeOH); HR-ESI-MS (*m/z* 597.1059 $[M+H]^+$, calcd, 597.1061); ^1H NMR (500 MHz, DMSO-d_6) δ: 7.31 (1H, m, 5‴-H), 7.18 (1H, d, *J* = 2.5, 2‴-H), 7.13(1H, dd, *J* = 9.0, 2.5, 6‴-H), 6.86 (1H, dd, *J* = 9.0, 2.5, 4‴-H), 6.75 (2H, s, 2″, 6″-H), 6.35 (2H, s, 2′, 6′-H), 5.99 (1H, d, *J* = 2.5, 8-H), 5.88 (1H, d, *J* = 2.5, 6-H), 5.46 (1H, s, 3-H), 5.22 (1H, d, *J* = 2.5, 2-H), 4.58 (1H, d, *J* = 2.5, 4-H), 3.81 (3H, s, -OCH_3); ^{13}C NMR (125 MHz, DMSO-d_6) δ: 164.9 (O-C-O), 159.7 (C-3‴), 158.4 (C-5), 157.3 (C-7), 155.6 (C-9), 145.8 (C-3′, 5′), 145.4 (C-3″, 5″), 138.9 (C-4″), 136.2 (C-1‴), 132.5 (C-4′), 130.0 (C-1′), 127.6 (C-5‴), 121.4 (C-6‴), 118.4 (C-1″), 114.2 (C-2‴), 112.8 (C-4‴), 108.7 (C-2″, 6″), 105.2 (C-2′, 6′), 96.0 (C-10), 95.9 (C-6), 94.2 (C-8), 72.8 (C-2), 70.8 (C-3), 55.1 (C-OCH_3), 41.2 (C-4).

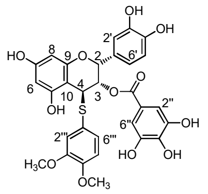

JP-52: UV λ_{max} (MeOH) nm: 278, 216; mp. 149.6~151.5°C; $[\alpha]_D^{25}$ -42.8° (*c* 0.1 MeOH); HR-ESI-MS (*m/z* 611.1208 $[M+H]^+$, calcd, 611.1218); ^1H NMR (500 MHz, DMSO-d_6) δ: 7.17 (1H, dd, *J* = 9.0, 2.5, 6‴-H), 7.16 (1H, d, *J* = 2.5, 2‴-H), 7.15 (1H, d, *J* = 9.0, 5‴-H), 6.80 (1H, d, *J* = 2.5, 2′-H), 6.72 (2H, s, 2″, 6″-H), 6.71 (1H, dd, *J* = 9.0, 2.5, 6′-H), 6.65 (1H, d, *J* = 9.0, 5′-H), 5.99 (1H, d, *J* = 2.5, 8-H), 5.85 (1H, d, *J* = 2.5, 6-H), 5.52 (1H, brs, 3-H), 5.16 (1H, brs, 2-H), 4.43 (1H, d, *J* = 2.5, 4-H), 3.80 (3H, s, 4‴-OCH_3), 3.78 (3H, s, 3‴-OCH_3); ^{13}C NMR (125 MHz, DMSO-d_6) δ: 164.7

(O-C-O), 158.3 (C-5), 157.3 (C-7), 155.6 (C-9), 148.9 (C-4‴), 148.7 (C-3‴), 145.4 (C-3″, 5″), 144.9 (C-3′), 144.8 (C-4′), 138.8 (C-4″), 128.5 (C-1′), 124.9 (C-1‴), 124.7 (C-6‴), 118.4 (C-1″), 117.4 (C-6′), 115.6 (C-5′), 115.2 (C-2′), 114.0 (C-5‴), 112.1 (C-2‴), 108.6 (C-2″, 6″), 96.5 (C-10), 95.9 (C-6), 94.2 (C-8), 72.6 (C-2), 70.8 (C-3), 55.5 (C-4‴-OCH_3), 55.4 (C-3‴-OCH_3), 42.6 (C-4).

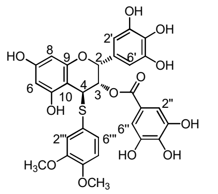

JP-53: UV λ_{max} (MeOH) nm: 278, 216; mp. 150.1~152.3°C; $[\alpha]_D^{25}$ -31.8° (*c* 0.1 MeOH); HR-ESI-MS (*m/z* 627.1172 $[M+H]^+$, calcd, 627.1167); ^{1}H NMR (500 MHz, DMSO-d_6) δ: 7.16 (1H, dd, *J* = 9.0, 2.5, 6‴-H), 7.14 (1H, d, *J* = 2.5, 2‴-H), 7.00 (1H, d, *J* = 9.0, 2.5, 5‴-H), 6.71 (1H, s, 2″, 6″-H), 6.36 (2H, s, 2′, 6′-H), 5.99 (1H, d, *J* = 2.5, 8-H), 5.86 (1H, d, *J* = 2.5, 6-H), 5.45 (1H, brs, 3-H), 5.20 (1H, brs, 2-H), 4.41 (2H, d, *J* = 2.5, 4-H), 3.81 (3H, s, 4‴-OCH_3), 3.78 (3H, s, 3‴-OCH_3); ^{13}C NMR (125 MHz, DMSO-d_6) δ: 164.8 (O-C-O), 158.3 (C-5), 157.3 (C-7), 155.6 (C-9), 148.9 (C-4‴), 148.7 (C-3‴), 145.8 (C-3′, 5′), 145.4 (C-3″, 5″), 138.8 (C-4″), 132.5 (C-4′), 127.8 (C-1′), 124.8 (C-1‴), 124.7 (C-6‴), 118.5 (C-1″), 115.4 (C-5‴), 112.2 (C-2‴), 108.7 (C-2″, 6″), 105.2 (C-2′, 6′), 96.6 (C-10), 96.0 (C-6), 94.2 (C-8), 72.6 (C-2), 70.8 (C-3), 55.5 (C-4‴-OCH_3), 55.4 (C-3‴-OCH_3), 42.7 (C-4).

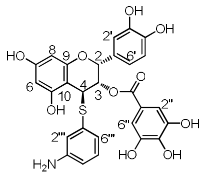

JP-54: UV λ_{max} (MeOH) nm: 278, 216; mp. 144.3~145.6°C; $[\alpha]_D^{25}$ -20.2° (*c* 0.1 MeOH); HR-ESI-MS (*m/z* 588.0941 $[M+H]^+$, calcd, 588.0935); ^{1}H NMR (500 MHz, DMSO-d_6) δ: 7.04 (1H, m, 2‴-H), 6.79 (2H, m, 5‴, 6‴-H), 6.77 (1H, d, *J* = 2.5, 2′-H), 6.71 (2H, s, 2″, 6″-H), 6.68 (1H, dd, *J* = 9.0, 2.5, 6′-H), 6.65 (1H, d, *J* = 9.0, 5′-H), 6.50 (1H, dd, *J* = 9.0, 2.5, 4‴-H), 5.98 (1H, brs, 8-H), 5.86 (1H, brs, 6-H), 5.58 (1H, brs, 3-H), 5.18 (1H, brs, 2-H), 4.48 (1H, brs, 4-H); ^{13}C NMR (125 MHz, DMSO-d_6) δ:

164.6 (O-C-O), 158.3 (C-5), 157.3 (C-7), 155.6 (C-9), 149.3 (C-3‴), 145.4 (C-3″, 5″), 144.9 (C-3′, 4′), 134.4 (C-4″), 129.6 (C-1‴), 129.5 (C-1′), 128.5 (C-5‴), 117.7 (C-1″), 117.4 (C-6′), 115.6 (C-6‴), 115.2 (C-5′), 114.0 (C-2′), 113.0 (C-2‴), 111.3 (C-4‴), 108.6 (C-2″, 6″), 96.4 (C-10), 95.9 (C-6), 94.1 (C-8), 72.8 (C-2), 70.6 (C-3), 41.4 (C-4).

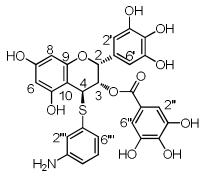

JP-55: UV λ_{max} (MeOH) nm: 278, 216; mp. 149.2~151.5°C; $[\alpha]_D^{25}$ -16.6° (*c* 0.1 MeOH); HR-ESI-MS (*m/z* 604.0874 $[M+H]^+$, calcd, 604.0884); ^{1}H NMR (500 MHz, DMSO-d_6) δ: 7.05 (1H, m, 2‴-H), 6.78 (2H, m, 5‴, 6‴-H), 6.73 (2H, s, 2″, 6″-H), 6.51 (1H, dd, *J* = 8.0, 2.0, 4‴-H), 6.35 (1H, s, 2′, 6′-H), 5.98 (1H, d, *J* = 2.0, 8-H), 5.87 (1H, d, *J* = 2.0, 6-H), 5.51 (1H, brs, 3-H), 5.23 (1H, brs, 2-H), 5.18 (2H, brs, $-NH_2$), 4.47 (1H, d, *J* = 2.0, 4-H); ^{13}C NMR (125 MHz, DMSO-d_6) δ: 164.6 (O-C-O), 158.3 (C-5), 157.3 (C-7), 155.6 (C-9), 149.3 (C-3‴), 145.8 (C-3′, 5′), 145.4 (C-3″, 5″), 138.9 (C-4″), 134.9 (C-1‴), 132.5 (C-4′), 129.6 (C-1′), 127.8 (C-5‴), 118.5 (C-1″), 117.5 (C-6‴), 115.3 (C-2‴), 112.9 (C-4‴), 108.7 (C-2″, 6″), 105.2 (C-2′, 6′), 96.5 (C-10), 95.9 (C-6), 94.1 (C-8), 72.7 (C-2), 70.6 (C-3), 41.5 (C-4).

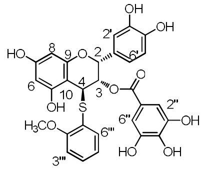

JP-56: UV λ_{max} (MeOH) nm: 278, 216; mp. 139.5~142.7°C; $[\alpha]_D^{25}$ -66.3° (*c* 0.1 MeOH); HR-ESI-MS (*m/z* 581.1118 $[M+H]^+$, calcd, 581.1112); ^{1}H NMR (500 MHz, DMSO-d_6) δ: 7.70 (1H, dd, *J* = 8.0, 1.5, 6‴-H), 7.30 (1H, m, 4‴-H), 7.06 (1H, dd, *J* = 8.0, 1.5, 3‴-H), 7.01 (1H, m, 5‴-H), 6.76 (1H, d, *J* = 2.0, 2′-H), 6.74 (2H, s, 2″, 6″-H), 6.68 (1H, dd, *J* = 8.0, 2.0, 6′-H), 6.50 (1H, d, *J* = 8.0, 5′-H), 5.98 (1H, d, *J* = 2.0, 8-H), 5.88 (1H, d, *J* = 2.0, 6-H), 5.62 (1H, brs, 3-H), 5.14 (1H, brs, 2-H), 4.56 (1H, d, *J* = 2.0, 4-H), 3.84 (3H, s, $-OCH_3$); ^{13}C NMR (125 MHz, DMSO-d_6) δ: 164.8 (O-C-O),

158.3 (C-5), 157.4 (C-7), 155.7 (C-9), 145.4 (C-3″, 5″), 144.9 (C-3′, 4′), 138.8 (C-4″), 131.1 (C-1′), 128.5 (C-6‴), 128.4 (C-4‴), 122.5 (C-1″), 121.1 (C-5‴), 118.5 (C-1‴), 117.4 (C-6′), 115.2 (C-5′), 114.0 (C-2′), 111.4 (C-3‴), 108.7 (C-2″, 6″), 96.3 (C-10), 96.0 (C-6), 94.2 (C-8), 72.9 (C-2), 70.4 (C-3), 55.8 (C-OCH_3), 40.4 (C-4).

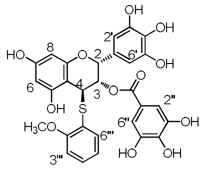

JP-57: UV λ_{max} (MeOH) nm: 278, 216; mp. 157.3~159.5°C; $[\alpha]_D^{25}$ -87.5° (*c* 0.1 MeOH); HR-ESI-MS (*m/z* 597.1068 $[M+H]^+$, calcd, 597.1061); ^{1}H NMR (500 MHz, DMSO-d_6) δ: 7.70 (1H, dd, *J* = 8.0, 1.5, 6‴-H), 7.30 (1H, m, 4‴-H), 7.08 (1H, dd, *J* = 8.0, 1.5, 3‴-H), 7.02 (1H, m, 5‴-H), 6.74 (2H, s, 2″, 6″-H), 6.33 (1H, s, 2′, 6′-H), 5.98 (1H, d, *J* = 2.5, 8-H), 5.88 (1H, d, *J* = 2.5, 6-H), 5.54 (1H, brs, 2-H), 5.18 (1H, d, *J* = 2.0, 3-H), 4.54 (1H, d, *J* = 2.5, 4-H), 3.85 (3H, s, -OCH_3); ^{13}C NMR (125 MHz, DMSO-d_6) δ: 164.8 (O-C-O), 158.3 (C-5), 157.4 (C-7), 155.7 (C-9), 145.8 (C-3′, 5′), 145.4 (C-3″, 5″), 138.8 (C-4″), 132.5 (C-4′), 130.8 (C-1′), 128.2 (C-6‴), 127.8 (C-4‴), 122.6 (C-1″), 121.1 (C-5‴), 118.6 (C-1‴), 111.4 (C-3‴), 108.7 (C-2″, 6″), 105.2 (C-2′, 6′), 96.3 (C-10), 96.0 (C-6), 94.2 (C-8), 72.9 (C-2), 70.3 (C-3), 55.8 (C-OCH_3), 40.4 (C-4).

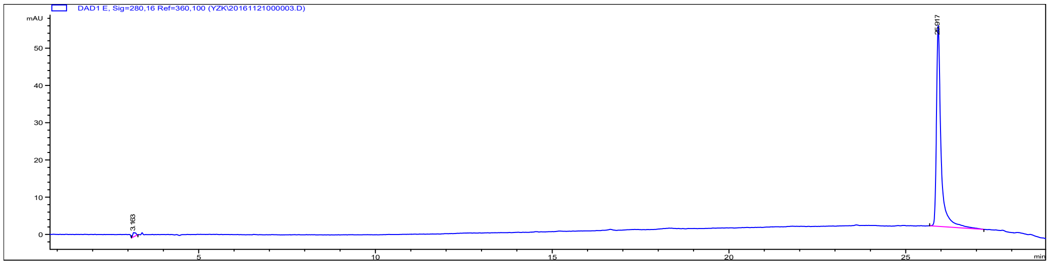

#	Time	Peak area	Peak height	Peak width	Symmetry factor	Peak area %
1	3.163	6.4	1.1	0.0823	0.544	1.282
2	25.917	491.5	53.9	0.1324	0.528	98.718

Figure S170. HPLC analysis of the purity of JP-8 (98.7%). Chromatographic column: Apollo C_{18} 5μm, 250 × 4.6 mm; mobile phase: methanol-water (0.2% acetic acid) (5:95-100:0, 30min); flow rate: 1mL/min; column temperature: 40℃; detection wavelength: 280nm.

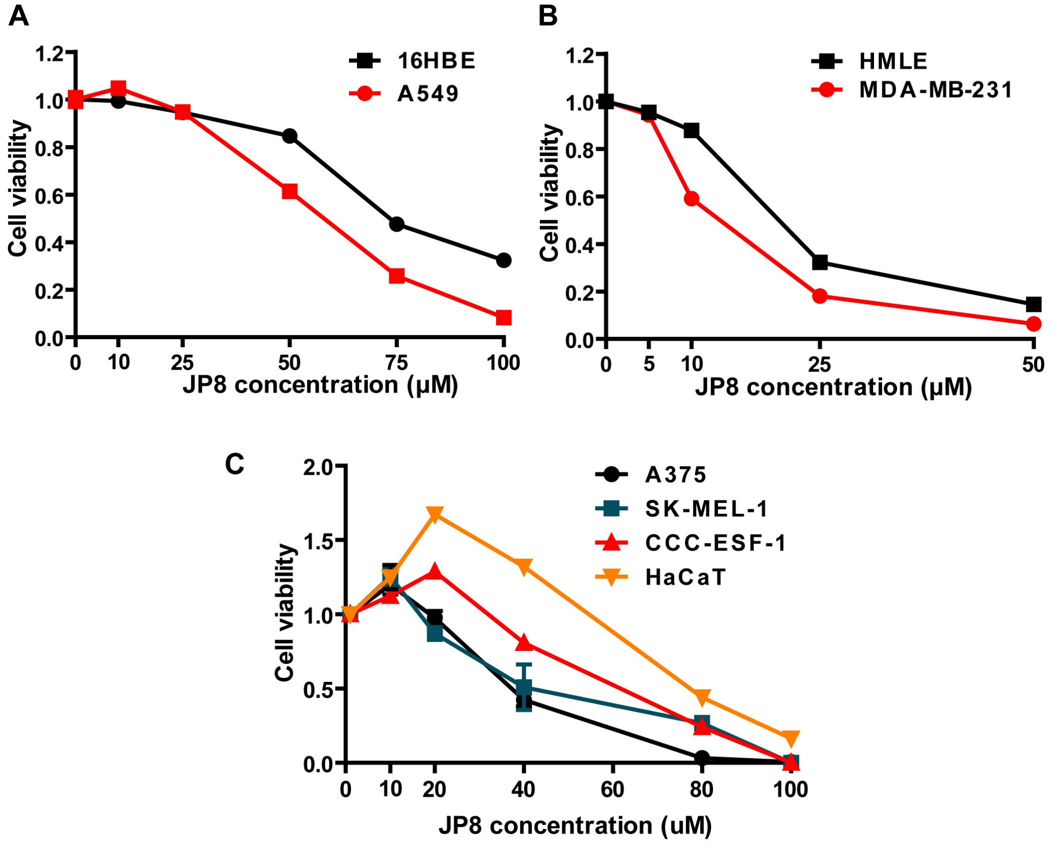

Figure S171. Effects of JP8 on the growth inhibition of human lung cancer cell A549, human breast cancer cell MDA-MB-231, human malignant melanoma cell A375 and human malignant melanoma cell SK-MEL-1. The cytotoxity of JP8 on human bronchial epithelial cell 16HBE, human mammary epithelial cell HMLE, immortalized human keratinocyte cell HaCat and huaman embryonic skin fibroblast cell CCC-ESF-1 was also determined. Cells were grown in 96 well plates and treated with JP8 at 5-100 μM or DMSO (control) for 48 h. Cytotoxicty was measured using cell counting kit-8 (CCK-8).

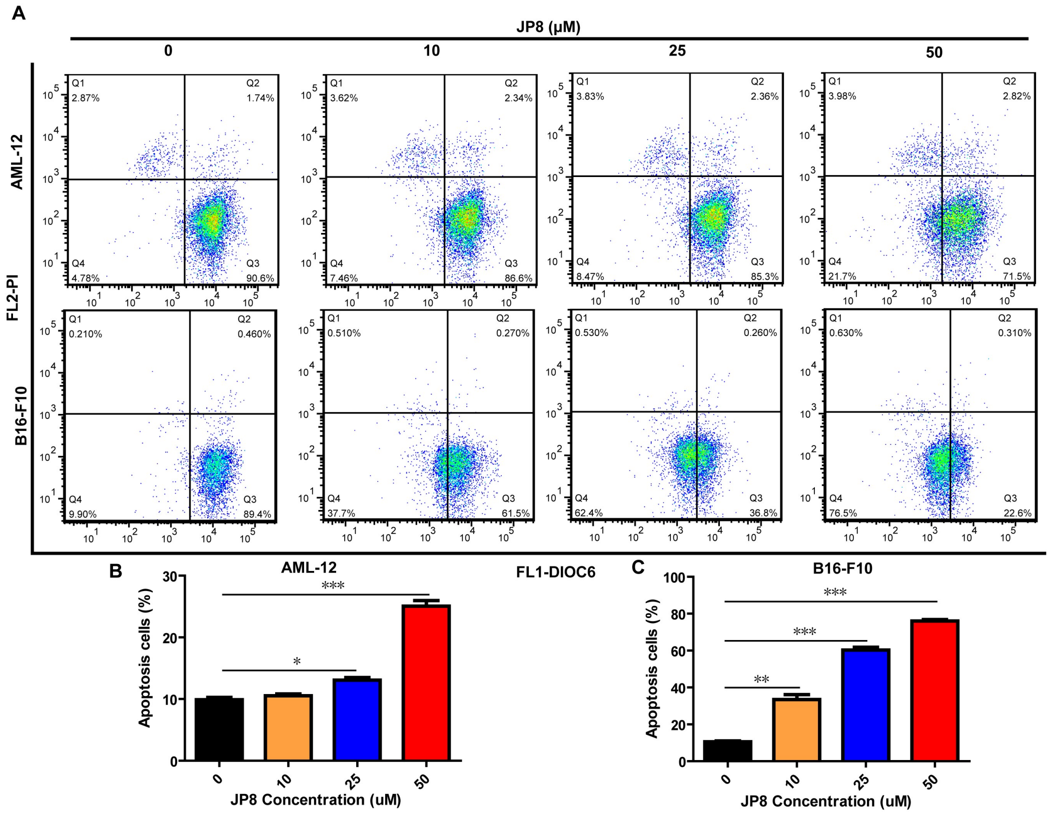

Figure S172. Apoptotic effects of JP8 on B16-F10 and AML-12 cells. Cells were double stained by DIOC6/PI after JP8 treatment at indicated concentration for 48h (A). The drop in mitochondria transmembrane potential can be detected with the dye DIOC6. Live cells are featured with DIOC ^{high} and PI ^{low}(the Q3 quadrant), early apoptotic cells are featured with DIOC ^{low} and PI ^{low} (the Q4 quadrant) and late apoptotic cells are featured with DIOC ^{low} and PI ^{high} (the Q4 quadrant). Q1 and Q4 quadrant cells were quantified as apoptosis cells (B and C).

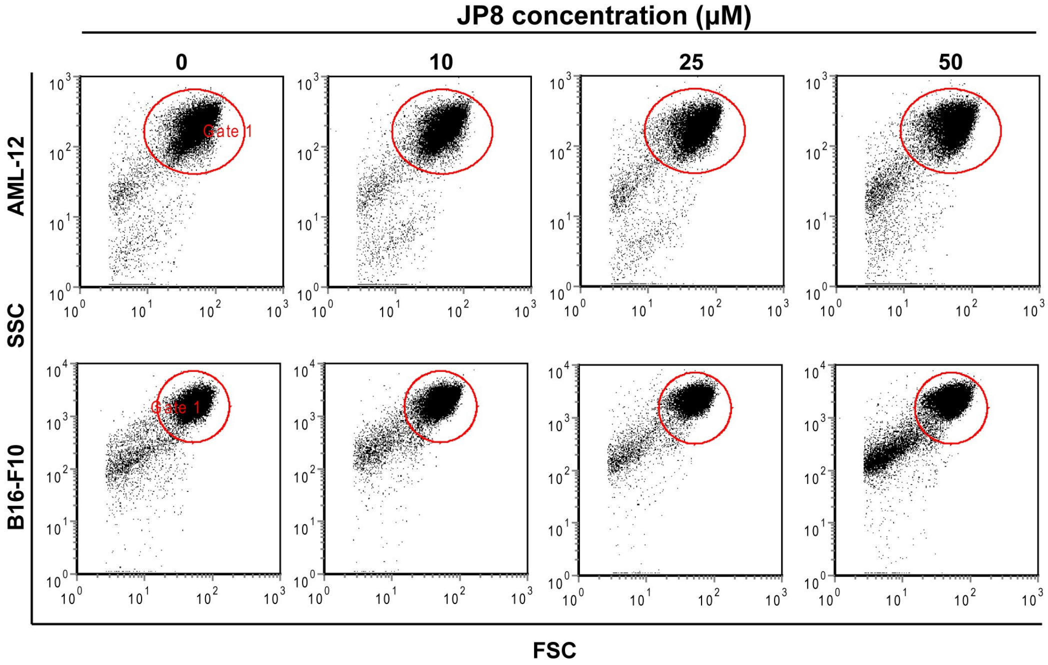

Figure S173. Morphological displays of JP8 treated AML-12 and B16 cells evidenced with FSC/SSC criteria.

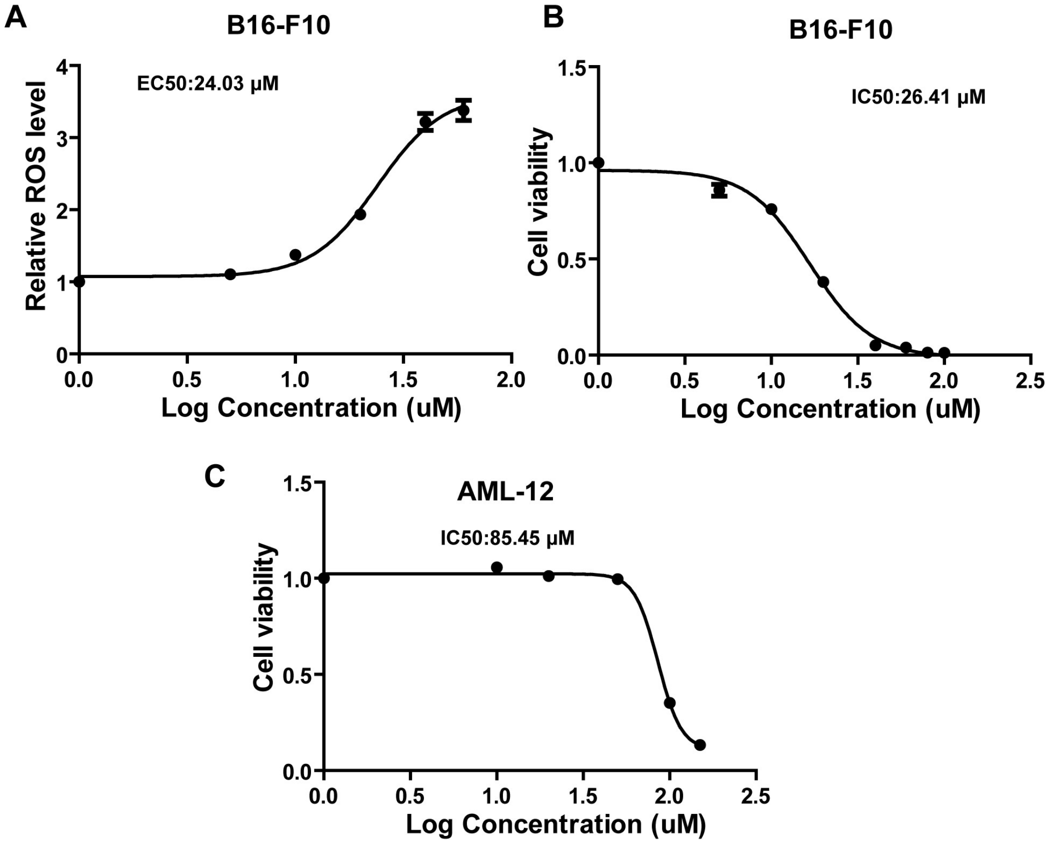

Figure S174. Does-response curves of JP8 on ROS induction and cell growth inhibition on B16-F10 and AML-12 cells. Cells were grown in 12 wells plate and incubated with different concentration of JP8 for 48h. ROS levels were measured and regression curves were fitted (A). Cells were grown in 96 well plates and treated with JP8 at different concentration for 48 h. Cytotoxicty was measured using cell counting kit-8 (CCK-8) and regression curves were fitted (B and C).

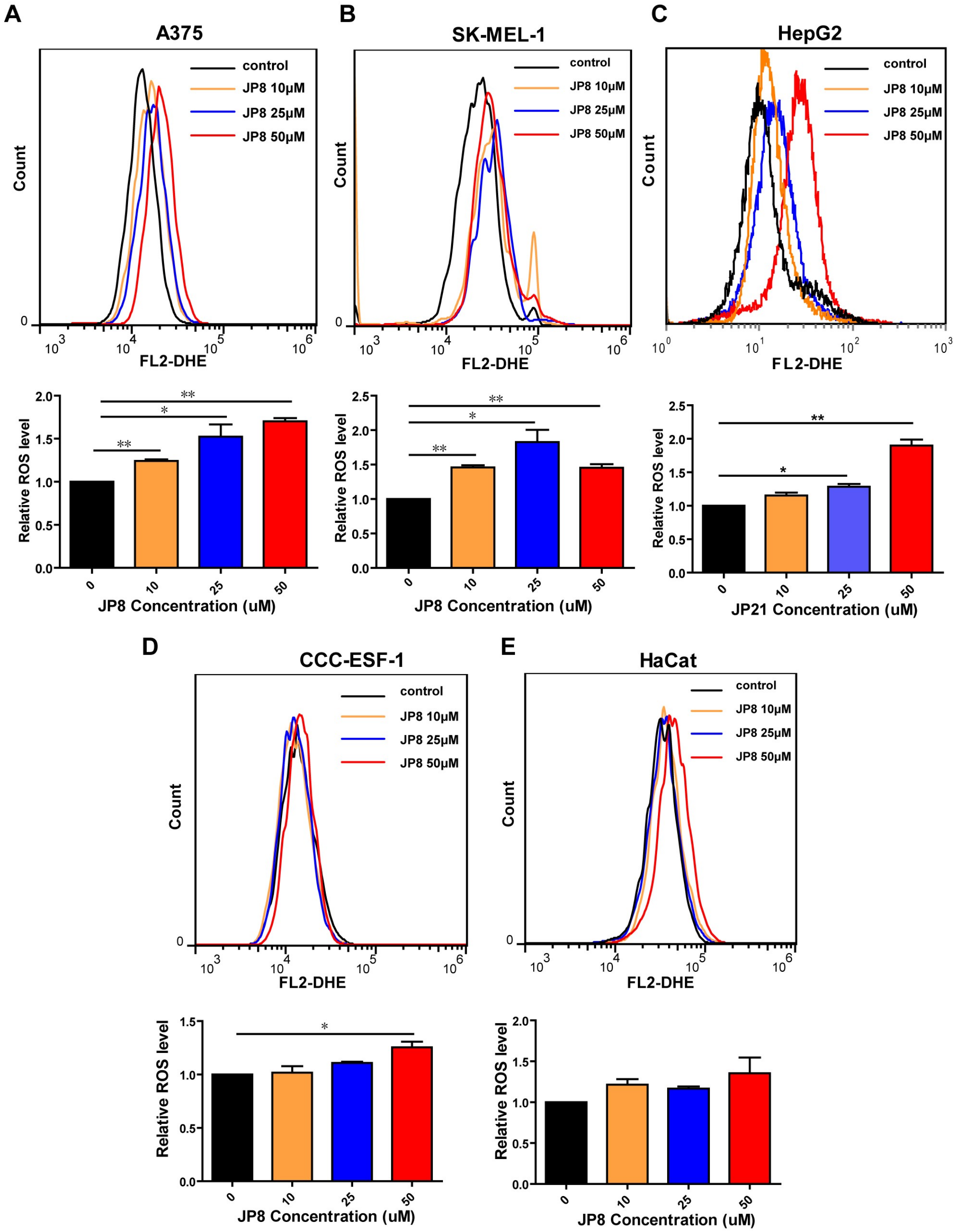

Figure S175. ROS accumulation levels of JP8 treated cancer and normal cell lines. Cells were treated with JP8 at indicated concentration for 12h and then stained with DHE. ROS levels were analyzed by flow cytometry.

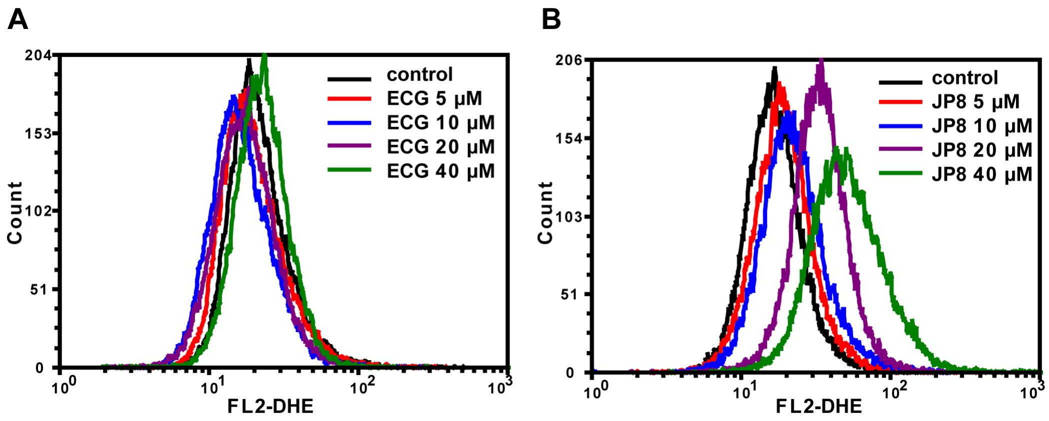

Figure S176. JP8 but not ECG induces ROS accumulation in a does dependent manner. B16-F10 cells were treated with ECG (A) and JP8 (B) at gradient concentrations for 24 h and stained with DHE. ROS levels were analyzed by flow cytometery.

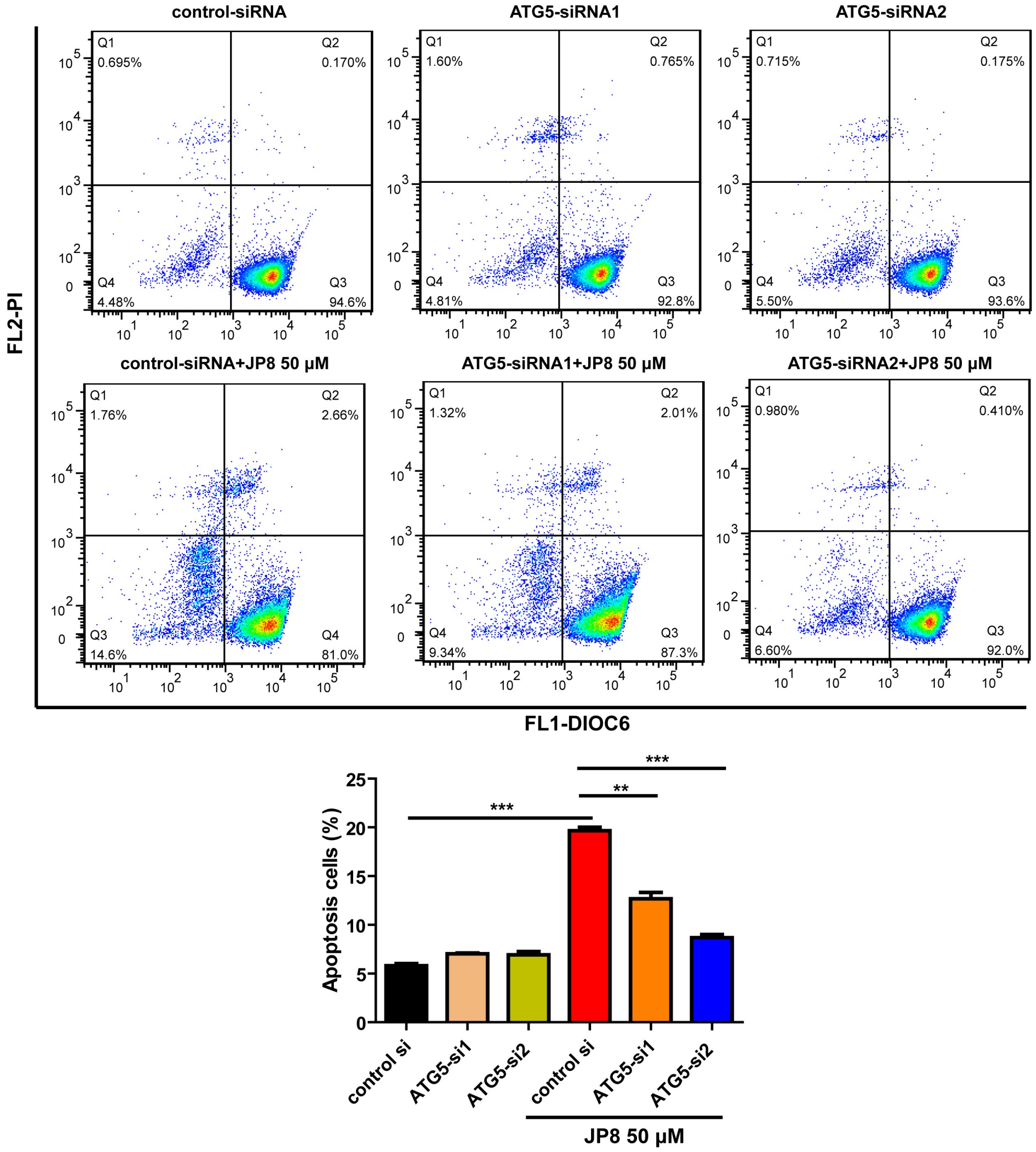

Figure S177. Silencing of ATG5 attenuates JP8 induced cell death in AML-12 cells. Control-siRNA or ATG5-siRNA-transfected cells were treated with 50 μM JP8 for 24h. Cell apoptosis was measured by DIOC6/PI assays.
